# Development of Water-Trapping Pyrrole-2-carboxylic Acids as Broad-Spectrum Metallo-β-lactamase Inhibitors

**DOI:** 10.1021/acs.jmedchem.5c03534

**Published:** 2026-05-14

**Authors:** Monisha Singha, Liam A. Wilson, Elisabete C. C. M. Moura, Maria M. Trush, Karina Calvopina, Gurleen Kaur, Greta Zaborskytė, Toms Kalniņš, Tharindi Panduwawala, Matthew J. Bowen, Matthew J. Beech, Jürgen Brem, Peter J. McHugh, Edgars Suna, Timothy R. Walsh, Christopher J. Schofield, Alistair J. M. Farley

**Affiliations:** † Chemistry Research Laboratory, Department of Chemistry, and the Ineos Oxford Institute for Antimicrobial Research, 6396 University of Oxford, 12 Mansfield Road, Oxford OX1 3TA, United Kingdom; ‡ Sir William Dunn School of Pathology, Department of Biology, and the Ineos Oxford Institute for Antimicrobial Research, University of Oxford, S Parks Rd, Oxford OX1 3RE, United Kingdom; § 187008 Latvian Institute of Organic Synthesis, Riga LV-1006, Latvia; ∥ Department of Oncology, MRC-Weatherall Institute of Molecular Medicine, University of Oxford, Oxford OX3 9DS, United Kingdom

## Abstract

Use of the clinically vital β-lactam antibiotics is increasingly compromised by resistance, commonly mediated by β-lactamases. While clinically used serine-β-lactamase (SBL) inhibitors have long been available, metallo-β-lactamase (MBL) inhibitors are not yet approved for clinical use. We report the structure-guided development of pyrrole-2-carboxylic acid derivatives as potent inhibitors of the clinically important di-Zn­(II) ion containing B1 MBLs (NDM-1, VIM-1, VIM-2, IMP-1). Crystallographic studies reveal the pyrrole-2-carboxylic acids inhibit B1 MBLs via active site Zn­(II)-coordination of the inhibitor carboxylate and trapping of the di-Zn­(II) ion bridging hydroxide, the latter of which reacts with the substrate β-lactam ring during hydrolysis. Appropriately derivatized pyrrole-2-carboxylic acids enhance the activity of carbapenems against MBL producing Gram-negative clinical isolates. The results support further development of metalloenzyme inhibitors that exploit binding to structural or catalytically important water molecules, an approach which may help in achieving selectivity over other metalloenzymes compared to metal-chelation based approaches.

## Graphical abstract

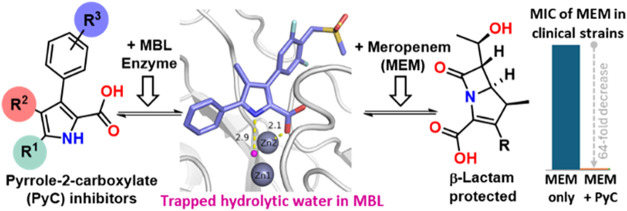

## 1 Introduction

Antimicrobial resistance (AMR) is increasingly compromising the efficacy of all antibiotics, including the clinically vital β-lactams (Figure 1a),^1^ for which an important resistance mechanism involves their β-lactamase catalyzed inactivation.^2^ There are two mechanistic classes of β-lactamases, i.e., the nucleophilic serine-β-lactamases (SBLs, Ambler classes A, C, D) and the metallo-β-lactamases (MBLs, Ambler class B). SBL mediated resistance has been countered clinically by the development of β-lactam antibiotics resistant to SBLs, including the carbapenems, and by the development of focused SBL inhibitors for use in combination with a β-lactam antibiotic. The pioneer SBL inhibitor was clavulanic acid, which is still widely used in combination with amoxicillin. Other established SBL inhibitors include sulbactam and tazobactam, which, like clavulanic acid, contain a β-lactam ring. More recently, enmetazobactam (a tazobactam derivative) and non-β-lactam SBL inhibitors, i.e., avibactam and other related diazabicycloctanes (DBOs) and vaborbactam have been approved for clinical use.^3–10^

**Figure 1:**
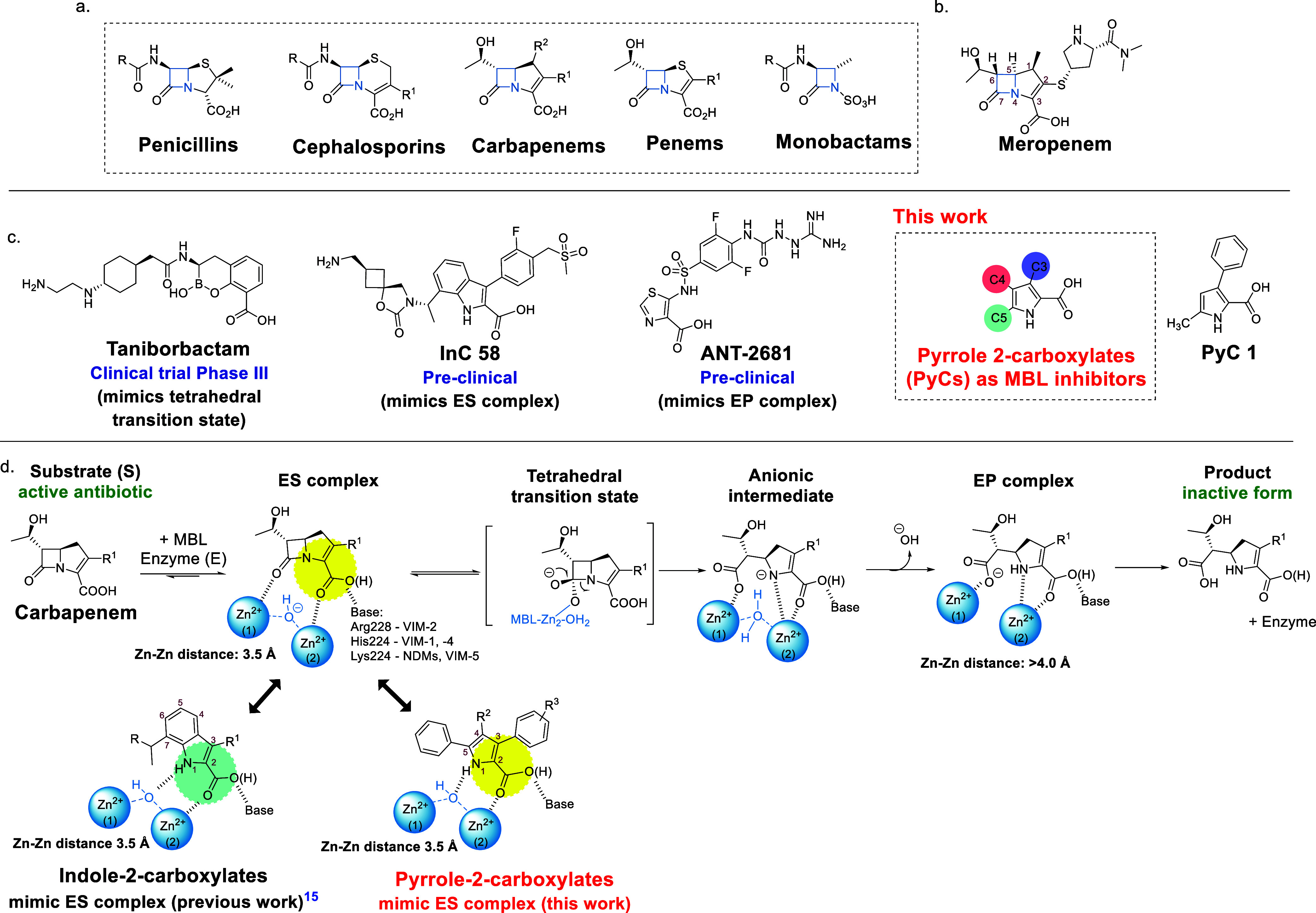
Metallo β-lactamase inhibitors. (a) The major classes of β-lactam antibiotics. (b) Structure of Meropenem, which was used as a partner antibiotic in our studies with MBL inhibitors. (c) Structures of selected MBL inhibitors currently in clinical or preclinical development^13^ and of the pyrrole-2-carboxylic acids (PyC), the subject of our work. (d) Outline mechanism of carbapenem hydrolysis by MBLs, and outline binding modes of MBL inhibition by indole-2-carboxylates and PyCs.

Gram-negative ESKAPE pathogens bearing MBLs pose an increasing threat to global health. Both carbapenems and the β-lactam containing SBL inhibitors are increasingly susceptible to SBL and MBL variants, contributing to growing resistance caused by β-lactamase–producing Gram-negative ESKAPE pathogens, which are a major cause of mortality.^11–13^

Although bicyclic boronates show considerable promise as dual SBL and MBL inhibitors, their inhibitory spectrum against MBLs is non-optimal; taniborbactam has an IC_50_ of ≈2.5 μM against IMP-1 (B1 subclass) and shows little or no inhibition against B2 (CphA) and B3 (L1) MBLs.^14–23^ We and others have thus been interested in developing focused MBL inhibitors (MBLI) aimed at protecting carbapenems which are often regarded as last-resort antibiotics.^24^

MBLs contain either one (Ambler subclass B2) or two Zn­(II) ions (Ambler subclasses B1, B3) in their active site, with B1 MBLs currently being the most clinically relevant subclass, in particular the IMP (imipenemase), VIM (Verona integron-encoded metallo-β-lactamase), and NDM (New Delhi metallo-β-lactamase) B1 subfamilies.^25,26^ Inhibition of MBLs is challenging in part due to the structural diversity of active sites across different clinically relevant MBL variants and the need to avoid off-target inhibition of human MBL fold enzymes and other metalloenzymes with related active site chemistry.^27^

Various classes of MBLI have been reported, including the Zn­(II) ion chelator aspergillomarasmine A^28^ and active site binding compounds, including the thiazole-4-carboxylic acid derivative ANT-2681,^29^ *N*-sulfamoylpyrrole-2-carboxylates,^30^ biphenyl tetrazole derivatives,^31^ dihydro benzo-indole derivatives,^32^ sulfamoylfuran-3-carboxylic acids,^33^ and cyclic boronates^34^ (Figure 1c). Biyclic boronate MBLI include taniborbactam and xeruborbactam;^35,36^ the binding modes of boronates is proposed to mimic that of the β-lactam substrate and/or tetrahedral intermediates during catalysis,^17^ inhibition modes relevant to both SBLs and MBLs.^14^ Most reported bicyclic boronates, including those in clinical development, however, only show limited activity against the different types of B1 MBL variants.^14,37–39^

Most reported active site binding B1 MBLIs currently in development bind to the di-Zn­(II) unit in a manner involving displacement of the catalytically important di-Zn­(II) ion bridging hydroxide (or water), which reacts with the substrate β-lactam ring during catalysis.^40–46^ Exceptions to this mode of inhibition are of interest, including with respect to selectivity over human MBL fold enzymes.^47^ Recently, we reported indole-2-carboxylate (InC) derivatives as broad-spectrum B1 MBLI, the binding mode of which was proposed to mimic initial binding of β-lactam substrates to MBLs in a manner in which the di-Zn­(II) bridging hydroxide is retained (Figure 1d).^15,48^ The InCs restore carbapenem activity against multiple multidrug-resistant Enterobacterales and have *in vivo* efficacy in murine infection models.^15^

Building on the InC binding mode where the indole NH interacts with the di-Zn­(II) bridging hydroxide/water molecule, we hypothesized that alternative heterocycles could adopt a similar binding mode within the MBL active site while maintaining selectivity over human MBL-fold enzymes. Here we report on the identification and structure guided development of pyrrole-2-carboxylic acids (PyC)^49^ derivatives as potent inhibitors of clinically relevant B1 subfamily MBLs (NDM, VIM, and IMP) that potentiate carbapenem efficacy against multiple B1 MBL producing Gram-negative clinical isolates.

## 2 Results

### 2.1 Synthesis of Pyrrole-2-carboxylic Acid Derivatives

To investigate proof of concept for PyCs as MBLIs, the probe **PyC** **1** was initially prepared via β-keto-enamine intermediate **21**, which was synthesized from ethyl glycine and acetyl acetophenone (Figure 2).^50^ Following base mediated condensation of the β-keto-enamine **21** to give the pyrrole core (**22**), ester hydrolysis gave the 2,3,5-substituted **PyC 1** in 8% overall yield. We then tested **PyC 1** for inhibition against a panel of clinically relevant B1 MBLs (NDM-1, VIM-1, VIM-2, and IMP-1) using a reported fluorogenic assay.^51^ While the observed inhibition was modest (NDM-1 pIC_50_ 4.9, Table 1, PyC no. 1), this result supported the potential for PyCs as MBLI and a small set of 2,3,5-substituted and 2,3,4,5-substituted PyCs was thus subsequently prepared.

**Figure 2:**
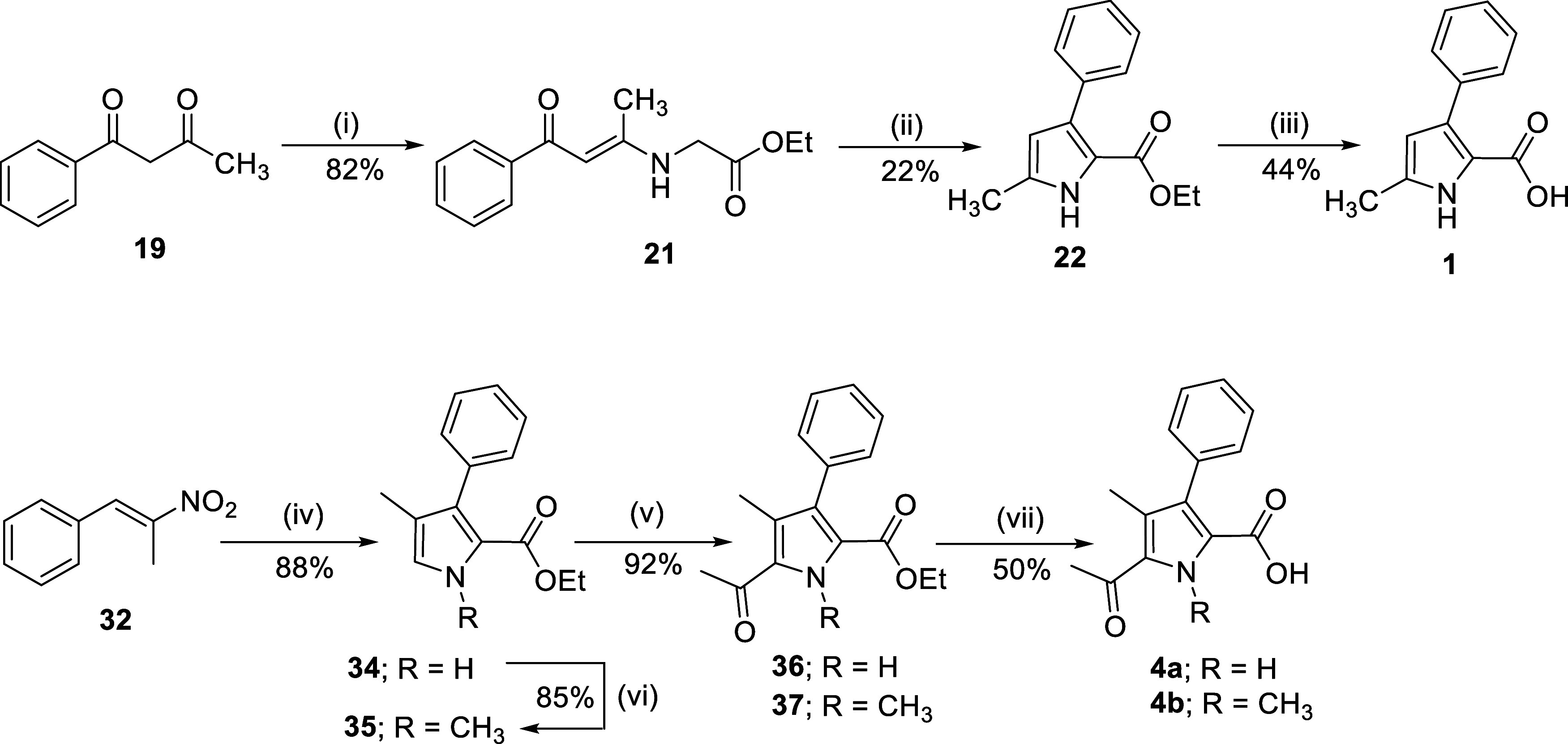
Synthesis of 4,5-substituted 3-phenyl pyrrole-2-carboxylic acids **1**, **4a–b.** Reagents and conditions: (i) GlyOEt·HCl (**20**), triethylamine, EtOH, rt, 48 h; (ii) NaOEt, EtOH, reflux, 2 h; (iii) 2.5 M NaOH, THF/EtOH, rt, 16 h; (iv) ethyl 2-isocyanoacetate (**33**), DBU, THF, ^*i*^PrOH, 10 °C to rt, 4 h; (v) Ac_2_O, BF_3_·OEt_2_, CH_2_Cl_2_, 0–20 °C, 2 h; (vi) CH_3_I, K_2_CO_3_, DMF/1,4-dioxane (1:1), 90 °C, 7 h; (vii) KOH, THF/H_2_O, 50 °C, 24 h.

**Table 1:**
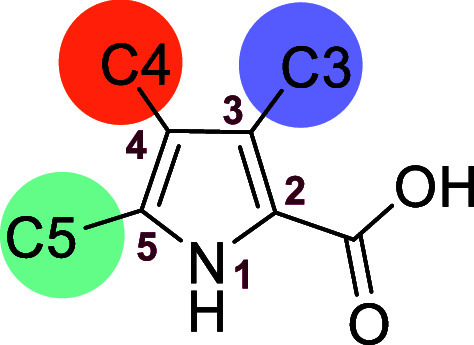

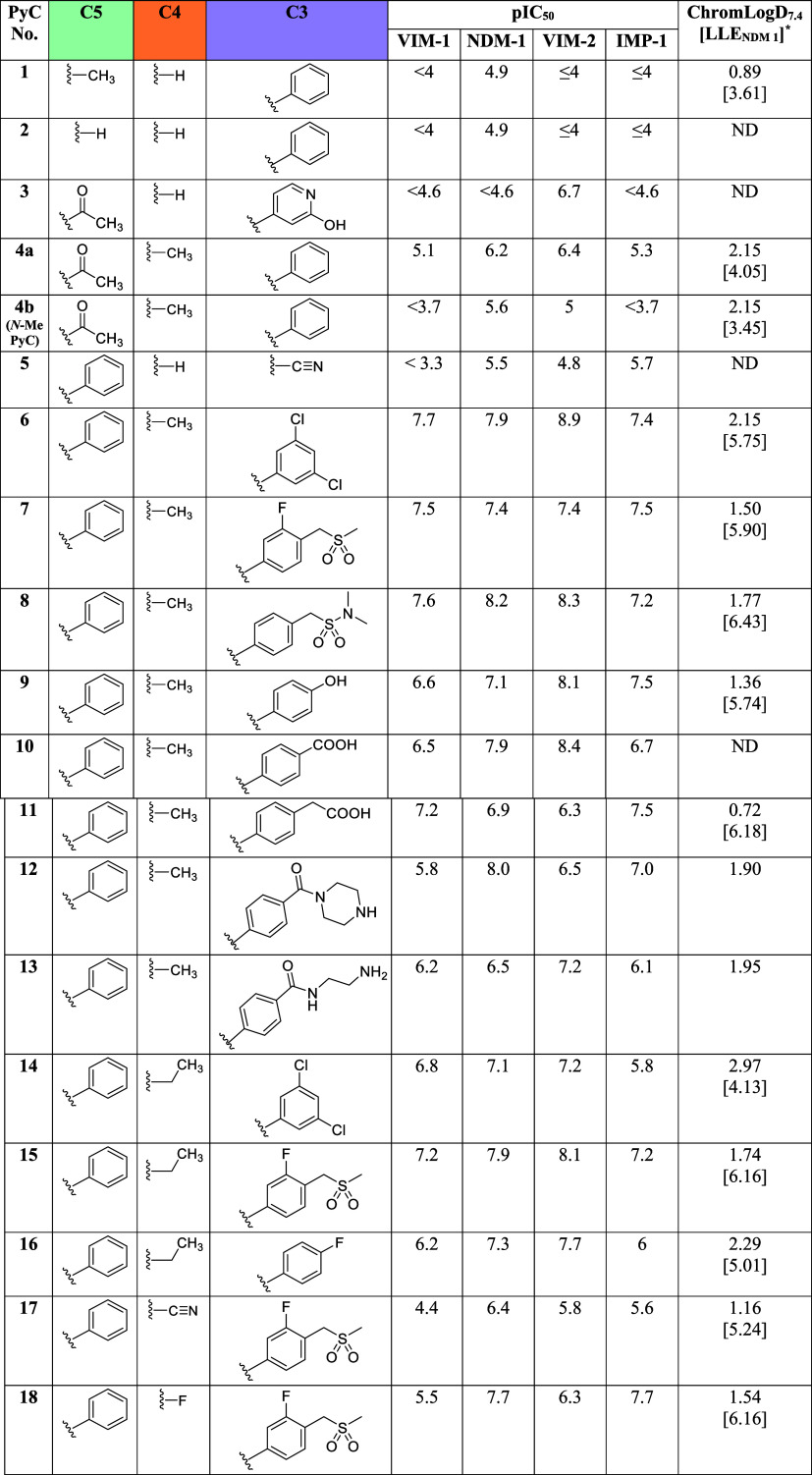
Inhibitory Activities of PyCs against VIM-1, NDM-1, VIM-2, and IMP-1 MBLs, with ChromLogD and Ligand-Lipophilicity Efficiency (LLE) Values[Table-fn t1fn2]

* ChromLogD_7.4_: ChromLogD at pH 7.4 was experimentally measured using reverse-phase HPLC calibrated with standards;^57,58^ Calculated LLE_NDM‑1_: (pIC_50_NDM-1 – ChromLogD_7.4_).

a Final enzyme concentrations: NDM-1 (20 pM), VIM-1 (500 pM), VIM-2 (100 pM), and IMP-1 (20 pM). Final FC5 concentration: 5 μM. Inhibitor concentrations ranged from 50 pM to 100 μM. All pIC_50_ experiments were run at pH 7.2 and are the mean of four replicates.

**PyC 2** and **3** were prepared from commercial 3-bromo-1*H*-pyrrole-2-carboxylate, over two steps and four steps, respectively (see Supporting Information for details). Compared to **PyC 1**, there was no significant improvement in the observed inhibition of NDM-1, VIM-1, and IMP-1 by both these compounds, however, **PyC 3** showed a slight increase in pIC_50_ against VIM-2 (Table 1, PyC no. 3). The 5-acetyl-4,3-substituted **PyC 4a** was then conveniently prepared via Barton–Zard reaction of *trans*-β-methyl-β-nitrostyrene and ethyl 2-isocyanoacetate to form the pyrrole core (**34**, Figure 2) in a single step in 88% yield.^52^ Acylation of the pyrrole ring at the C5 position using Friedel–Crafts conditions^53^ followed by ester hydrolysis gave **PyC 4a**. The intermediate **34** was *N*-methylated to form **35**, which similarly afforded **PyC 4b** over two steps involving acylation and ester hydrolysis (Figure 2).

We envisaged that substituting the C5 position of the PyC with an aromatic ring could enhance potency, as this modification may more closely mimic the binding of InCs to B1 MBLs, where structure activity relationship (SAR) studies and crystallographic evidence imply that the presence of a C7-group of the InC derivatives stabilizes binding of the di-Zn­(II) ion bridging hydroxide.^15^ Building on reported SAR at the C3 position of the InCs,^15^ we anticipated that a conserved active site pocket could be targeted for enhanced potency with an appropriately substituted aryl group at the C3 position of the PyC derivatives. This pocket is formed by Ser205, Thr206, Ser207, and Gly209 in the case of the B1 MBL VIM-1 (Figure 4). Gly209 is fully conserved among all the B1 MBLs tested in this study (VIM-1, VIM-2, NDM-1, and IMP-1), Ser207 is conserved among all but IMP-1, Thr206 is conserved in both VIM enzymes, while Tyr and Lys residues are present at residue-206 in IMP-1 and NDM-1, respectively. SAR and crystallographic analysis of the InCs shows that C3 substituents bind to the peptide backbone in this pocket enabling a conserved binding mode in the presence of variations of residues 205, 206, and 207.^15^

To conveniently synthesize PyCs with aryl substituents at C3, we prepared the 5-phenyl 3-iodo pyrroles **41a–c** in two steps by reacting α-substituted cinnamaldehydes **38a–b** with 2-azidoacetate esters,^49^ followed by electrophilic iodination using *N*-iodosuccinimide (NIS) for reaction at the pyrrole C3 position. The resulting iodinated-intermediates are suited for late-stage cross-coupling to enable efficient diversification (Figure 3). The 4-nitrile or 4-fluoro substituted pyrroles (**43** & **44**) were obtained via the reaction of 2-azidoacetate esters with cinnamaldehyde **38c** to afford a diene-azide intermediate, which was subsequently treated with a Lewis acid (ZnI_2_) to form the pyrrole (**40d**).^54^ The resulting pyrrole **40d** was subjected to electrophilic bromination, followed by palladium-catalyzed nitrilation^55^ to furnish **43**; electrophilic fluorination^56^ of **40d** yielded **44**. Electrophilic iodination of **43** and **44** with NIS afforded the corresponding 3-iodo-pyrroles **45** and **46**, respectively. Suzuki–Miyaura cross coupling of these iodo-derivatives (**41a**–**c**, **45**–**46**) with commercially available or readily prepared boronic acids or esters, followed by base mediated ester hydrolysis of the C2 carboxylate ester afforded the tetra-substituted pyrroles **PyC 6**–**18** (Figure 3). Preparation of **PyCs 12** and **13** necessitated installation of the C2-benzyl ester and subsequent Pd/C hydrogenation, due to competing base-mediated hydrolysis of the C3 amide during attempted deprotection of **12a** producing **PyC 10** (see Supporting Information for details).

**Figure 3:**
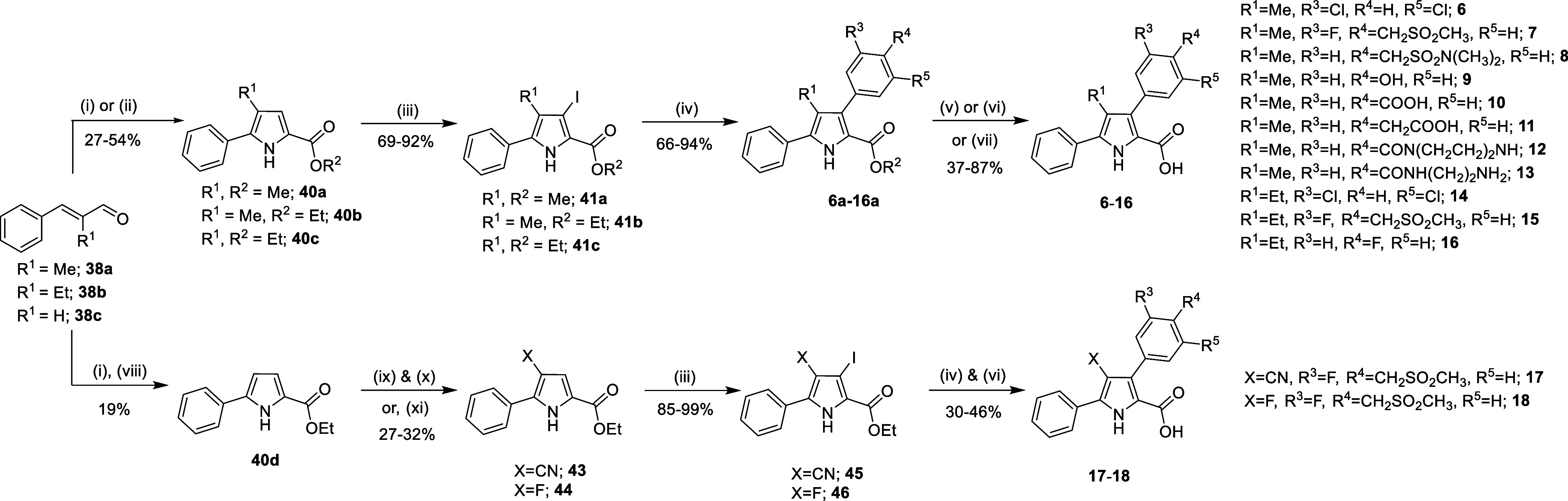
Synthesis of 3,4,5-substituted pyrrole-2-carboxylic acids. Reagents & conditions: (i) methyl 2-azido acetate (**39a**), NaOMe, MeOH, −20 to 0 °C, 4 h; (ii) ethyl 2-azido acetate (**39b**), NaOEt, EtOH, −20 to 0 °C, 4 h then rt, 1 h; (iii) NIS, DMF, rt, 3 h, (iv) ArB­(OR’)_2_ (**42a–h**), Pd­(dppf)­Cl_2_, Na_2_CO_3_ (or K_2_CO_3_), 1,4-dioxane, reflux, 4 h; For **PyC 2**, **6**, **7**, **8**, **9**, **14**: (v) LiOH·H_2_O, THF, H_2_O, EtOH (or MeOH), rt, 48 h; For **PyC 5**, **10**, **11**, **15**, **16**, **17**, **18**: (vi) 4 M KOH, THF, EtOH, rt, 12 h; For **PyC 12**, **13**: (vii) (a) Ti­(OEt)_4_, BnOH, 110 °C, 15 h, (b) TFA, CH_2_Cl_2_, rt, 2–3 h, (c) Pd/C, H_2_, rt, 2–6 h; (viii) ZnI_2_ (5 mol %), CH_2_Cl_2_, rt, 15 h; For **43**: (ix) NBS, CH_2_Cl_2_, −40 to 0 °C, 1 h, (x) K_4_[Fe­(CN)_6_]· 3H_2_O, KOAc, *t*-BuXPhos, *t*-BuXPhos-Pd-G3, 1,4-dioxane, H_2_O, 100 °C, 1 h; For **44**: (xi) Selectfluor, MeCN, 150 °C, 10 min, MW.

### 2.2 MBL Inhibition by PyCs

The PyCs were screened for inhibition against the B1 MBLs NDM-1, VIM-1, VIM-2 and IMP-1 using an established fluorogenic cephalosporin-based assay (Table 1).^51^ **PyC 6**–**18** with aryl substituents both at C3 and at C5 exhibited enhanced potency compared to **PyC 1**, for all the tested MBLs. In contrast, replacement of the PyC C5 aryl group with small alkyl or polar groups, such as methyl (**PyC 1**) or acetyl (**PyC 4a**) groups, led to reduced activity, consistent with the low activities observed for **PyC 2** which lacks both C4 and C5 substituents. Notably, **PyC 4b**, which has an *N*-methyl group on its pyrrole nitrogen, showed a substantial loss of activity against VIM-1 and IMP-1, highlighting the likely importance of the pyrrole NH group in hydrogen bonding with the di-Zn­(II) bridging hydroxide in the active site. Interestingly, however, moderate activity for **PyC 4b** (pIC_50_ 5.6) was observed with NDM-1. Changing the pyrrole C3 substituent from a phenyl- to a nitrile-group, while maintaining a phenyl group at C5 (**PyC 5**) also diminished activity, suggesting that specific electronic or steric features at C3 are critical for efficient binding (Table 1, PyC no. 5). Among **PyC 6**–**13**, which share a common C5 phenyl group and a C4 methyl group, but which differ in their C3 substituents, **PyC 8** and **6** (bearing C3 *N*,*N*-dimethylsulfonamide- and dichloro-substituents, respectively) exhibited the highest pIC_50_ values across the four MBLs tested, with **PyC 8** also showing the highest LLE_NDM‑1_ values. This result suggests favorable interactions between these C3 groups and a subpocket within the MBL active sites. Altering the pyrrole C4 position also influenced activity. For example, replacing the C4Me substitution in **PyC 6** with an ethyl group in **PyC 14**, slightly reduced potency, while a similar change in **PyC 7** to **PyC 15** had minimal effect (Table 1). This observation suggests that interactions involving the methyl sulfone group of the C3 aryl substituent in the latter pair help compensate for the loss in potency that would otherwise be expected.

We were interested in further investigating the influence of small polar, electron-withdrawing groups, such as nitrile- and fluoro- substituents, at C4 of the PyC derivatives, and therefore synthesized **PyC 17** and **18**. **PyC 17** showed an overall reduced inhibitory activity across all the four MBLs compared to their methyl or ethyl substituted counterparts (**PyC 7** and **15**, Table 1), suggesting that the polar nitrile group at C4 engages in unfavorable interactions that weaken the binding. On the other hand, **PyC 18**, with a C4 fluoro substituent, showed increased activity against NDM-1 and IMP-1, while showing diminished activity toward VIM-1 and VIM-2 relative to **PyC 7**.

To investigate selectivity over a structurally related human metalloenzyme, five representative PyC inhibitors were evaluated for inhibition of the human metallo-β-lactamase-fold nuclease SNM1C.^59^ **PyC 7**, **12**, **17**, and **18** showed no detectable inhibition under the assay conditions (IC_50_ > 100 μM), and the IC_50_ of **PyC 13** was 22 μM against SNM1C. These results show selectivity of at least 20-fold for inhibition of the bacterial MBLs over a human MBL-fold nuclease. ChromLogD values of the MBL inhibitors were measured at pH 7.4 using a reverse-phase HPLC method,^57,58^ and their lipophilic ligand efficiencies (LLEs) for NDM-1 were calculated (Table 1). We measured ChromLogD experimentally, as computational cLogD predictions can be unreliable for ionizable compounds.^60^ The **PyC 6**–**9**, **11**, **15**, and **18** exhibited LLE value > 5.5, which suggest a favorable balance of potency and lipophilicity.^61,62^ A high LLE indicates that a compound may achieve its inhibitory effect (low MIC) efficiently, without excessive lipophilicity.^60^ In further assessment of the drug-like properties of the PyCs, we evaluated **PyC 7** for cytotoxicity and **PyC 7**, **12** for stability in biological media and to liver microsomes (see Supporting Information Table S5). Pleasingly, **PyC 7** was noncytotoxic against the HepG2 cell line (IC_50_ > 150 μM) and both **PyC 7** and **12** showed no degradation after 24 h at 37 °C in pH 7.4 buffer. Furthermore, the *t*_1/2_ in both human and mouse plasma for both **PyC 7** and **12** was > 512 min and the *t*_1/2_ in human and mouse liver microsomes was determined to be > 256 min.

### 2.3 Crystallographic Analysis with VIM-1 Reveals Binding Modes of the PyCs to MBLs

High-resolution crystal structures of VIM-1 complexed with **PyC 6**, **7**, **8**, **11**, **14**, and **15** were obtained to study the binding modes (Figure 4). All the structures were obtained through cocrystallization of VIM-1, using reported conditions^15^ and were solved with one molecule in the asymmetric unit (*P*12_1_1 space group), with resolutions of 1.05–1.15 Å.

**Figure 4:**
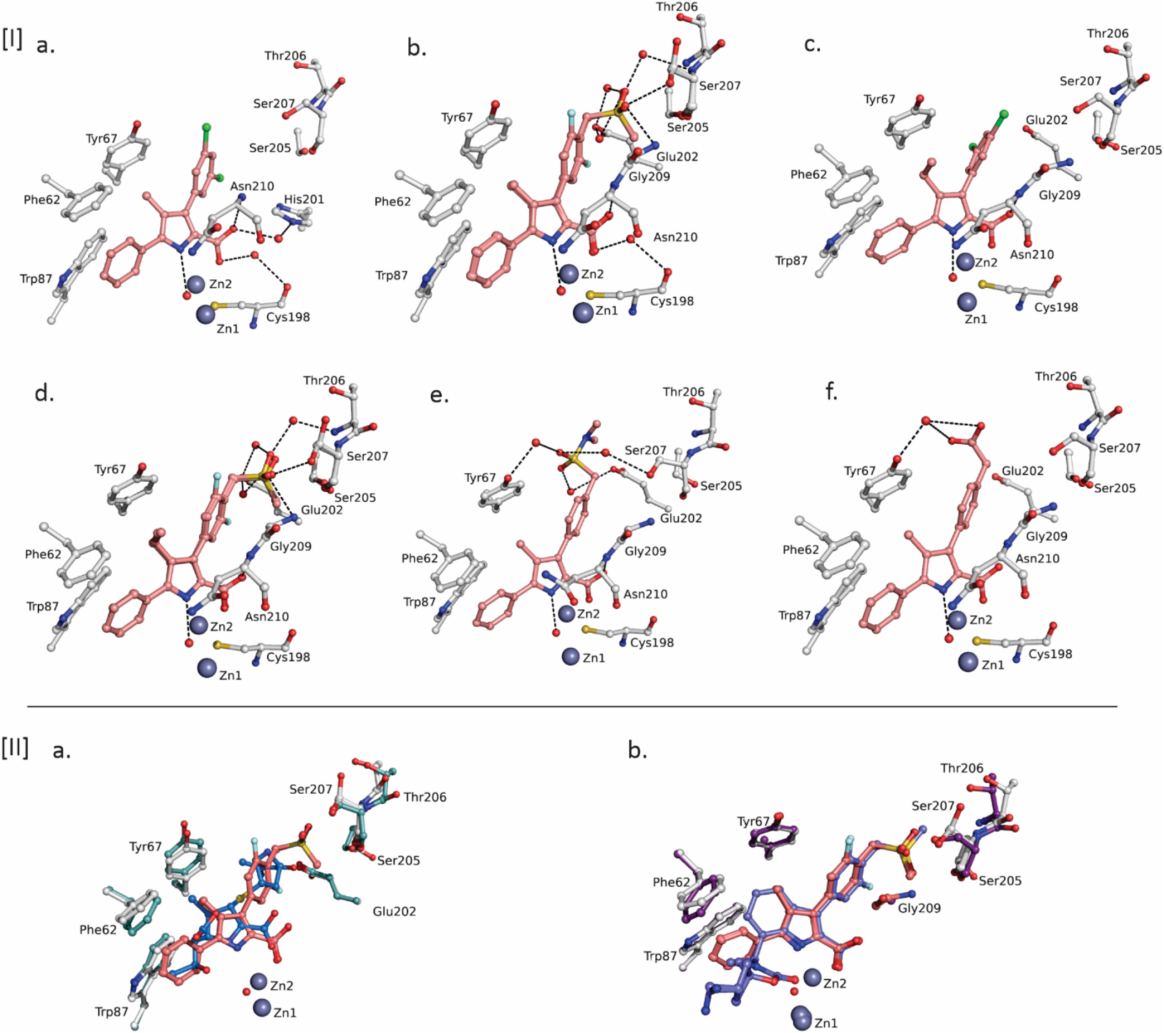
Binding of PyCs to the B1 subfamily MBL VIM-1 involves active site hydroxide trapping. [I] Active site views of the VIM-1 B1 MBL in complex with PyC derivatives: **PyC 6** (a) (PDB 9RFK); **PyC 7** (b) (PDB 9RFM); **PyC 14** (c) (PDB 9RFG); **PyC 15** (d) (PDB 9RFJ); **PyC 8** (e) (PDB 9RFI); and **PyC 11** (f) (PDB 9RFH). Hydrogen bonds and water “bridges” are depicted as dotted lines. For clarity, only the main-chain atoms of residues interacting with the ligand are shown. Of the two refined conformational isomers for Asn210 and His201, only one conformation is shown. **PyC 7** and **15** are displayed in two overlapping conformations showing flipping of the C3 aromatic ring. [II] Superimposition of crystal structure views of compound **PyC 7** (pink) complexed with VIM-1 with (a) hydrolyzed Meropenem (marine blue) (PDB 5N5I) and, (b) indole carboxylate **InC 59** (slate blue) (PDB 8PGE).

Notably, all of the high resolution PyC-VIM-1 complex structures obtained showed retention of the di-Zn­(II) bridging hydroxide (see SI Figure S6 for electron density maps and SI Table S1 for refinement statistics). They also show direct coordination to Zn2 by the PyC carboxylate (O–Zn­(II) distances 2.0–2.1 Å) and the presence of a hydrogen-bonding interaction between the di-Zn­(II) bridging hydroxide and the pyrrole NH (N–O distance 2.8–2.9 Å), replicating the binding mode seen with the InCs.^15^ The PyC carboxylate is further apparently stabilized by a hydrogen bonding interaction with the main chain NH of Asn210 (N–O distance 2.9–3.2 Å) and a water-bridging interaction with the main chain oxygen of Cys198. In all the PyC-bound structures obtained the refined Zn2–O distance (where Zn2 is the cysteine-bound Zn­(II) ion and O is the bridging hydroxide) is substantially longer than the Zn1–O distance, ranging from 2.0–2.1 Å (Zn2–O) and 1.9 Å (Zn1–O), respectively. These distances match those observed in the InC bound structures (Zn2–O and Zn1–O distances of 2.0 Å and 1.9 Å, respectively for PDB 8PGE). Note that, ligand-free VIM-1 has a slightly extended Zn2–O distance (2.2 Å, PDB 5N5G)^63^ compared to the inhibitor-bound structures. A crystal structure of VIM-1 bound to hydrolyzed Meropenem (PDB 5N5I) manifests an extended Zn2–O distance compared to both the inhibitor bound and unbound structures (2.3 Å).^63^ The refined Zn1–O distances are consistent when comparing the PyC-bound structures and those of VIM-1 bound to InC, hydrolyzed Meropenem or unbound VIM-1 (ranging between 1.8–1.9 Å).

The C5 phenyl ring of the PyC in all six of the VIM-1 crystal structures reported here is positioned to make π–stacking interactions with Phe62 (see SI Figure S8a), with the C4 ethyl/methyl group positioned to make hydrophobic interactions with Tyr67 and Phe62. A π–stacking interaction is observed between the C3 aromatic ring of PyC and His240 in all cases, except for **PyC 11** which instead is positioned to form a π–cation interaction with His201 (see SI Figure S8b). The C3 aryl substituents of **PyC 7** and **15** (Figure 4,[I]­b,[I]­d) show hydrogen-bond and water-bridging interactions with Thr206 and Ser207 in the hydrophilic pocket adjacent to the metal binding site. Neither **PyC 7** nor **15** is predicted to form hydrogen bonds with Ser205, despite the hydrophilic sulfone coming into relatively close proximity with the Ser205 hydroxyl group. Both **PyC 7** and **15** form additional (compared to **PyCs 6**, **8**, **11**, and **14**) hydrogen-bond interactions with Gly209 and make two water-bridging interactions with Glu202. The *N*,*N*-dimethyl sulphonamide group of **PyC 8** is flipped, compared to **PyC 7** and **15**, to interact with Tyr67 via water-bridging interactions, while maintaining water-bridge interactions with Ser207 and Glu202 (Figure 4,[I]­e). The carboxylic acid substituent of the C3 aryl group in **PyC 11** is also flipped relative to **PyC 7** and **15**, in a manner enabling formation of a water-bridging interaction with Tyr67 through its C3 group carboxylic oxygen atoms (Figure 4;[I]­f).

**PyC 6** and **14**, both of which have a C3 dichloro-phenyl group, are not positioned to make any additional protein–ligand interactions other than the π–stacking interactions with the C3 and C5 aromatic rings and the hydrogen bonding interaction between the pyrrole nitrogen and the di-Zn­(II) bridging hydroxide. Although additional protein–ligand interactions are apparently formed with **PyC 7**, **15**, **8**, and **11** in the VIM-1 active site compared to **PyC 6** and **14**, this difference does not, however, result in large increases in activity compared to **PyC 6** (Table 1).

Superimposition of the VIM-1 structures with **PyC 7** and the **InC 59** (the C7 epimer of **InC 58**)^15^ shows striking similarities in the binding modes (Figure 4,[II]­b). There are no substantial shifts in the positions of the five membered aromatic ring or the C3 PyC substituent. The methyl group at C4 of **PyC 7** superimposes directly with the indole C4 carbon in the **InC 59** structure, occupying the hydrophobic pocket. The spirocyclic oxazolidinone of **InC 58** and its epimer **InC 59** enable extended hydrophobic interactions with residues in the conserved hydrophobic pocket in MBLs, formed by the L3 loop in VIM-1,^64^ likely contributing to increased potency of the highly optimized InC structure compared with the C5-phenyl PyC analogues. **InC 58** is also positioned to form π–stacking interactions with Phe62, Tyr67, and Trp87, while the PyC compounds maintain hydrophobic interactions with these residues, and π–stacking with Phe62, the loss of interactions with Tyr67 and Trp87 likely decreases binding affinity. Substitution of the C4 methyl group of **PyC 7** with a fluorine (as in **PyC 18**) may results in loss of hydrophobic interactions, leading to reduced activity, whereas the nitrile group of **PyC 17** may make unfavorable interactions within the otherwise hydrophobic C4 group binding pocket. Interestingly, rather than mirroring the indole-phenyl ring binding mode, the C4 ethyl group of **PyC 15** is directed perpendicular to the pyrrole ring, in a position to stack with Tyr67. This interaction likely increases hydrophobic interactions with Phe62, and apparently causes Asn210 to adopt a single crystallographically observed conformation, potentially reflecting the stability of this complex, consistent with the increased activity of **PyC 15** against VIM-1 compared to **PyC 7** and **18** (pIC_50_ values: 8.1, 7.4, and 6.3 for **PyC 15**, **7**, and **18**, respectively).

Superimposition of a structure of VIM-1 in complex with hydrolyzed Meropenem in its dihydropyrrole tautomeric form with the **PyC 7**-bound VIM-1 structure shows closely related binding modes (Figure 4,[I]­b). The C1 side chain methyl group on the hydrolyzed Meropenem occupies a similar position to that of the C4 methyl of **PyC 7**, while the pyrrole ring and carboxylic acid of the hydrolyzed Meropenem are shifted away from the di-Zn­(II) bridging hydroxide (the NH to hydroxide oxygen distances for hydrolyzed Meropenem and **PyC 7** are 3.4 Å and 2.9 Å, respectively). This observation suggests that the stabilization of the bridging hydroxide is stronger with **PyC 7** bound to the active site compared to the hydrolyzed Meropenem product, perhaps reflecting the need for the active site to release the latter during catalysis. Thus, the binding mode of the PyCs may better imitate the enzyme–substrate (ES) complex during MBL hydrolysis rather than the hydrolyzed reaction product.

### 2.4 Minimum Inhibitory Concentration (MIC) Assays

The activity of selected PyCs to potentiate the activity of Meropenem (Figure 1b) was evaluated by determining minimum inhibitory concentration (MIC) values (Table 2) using an isogenic panel of *Escherichia coli* strains overexpressing the NDM-1, VIM-1, and VIM-2 MBLs (see SI Table S4 for details of the strains). To provide a clear genetic background for the assessment of β-lactamase inhibitory activity, we constructed an in-frame deletion of the native chromosomal β-lactamase *ampC* in *E. coli* K12 MG1655. The strain MG1655 Δ*ampC* was transformed with the recombinant plasmids pK18-NDM-1, pK18-VIM-1, pK18-VIM-2 and pK18-KPC-2, as well as with the empty vector pK18 as a control, to obtain strains IP93, IP90, IP41, IP42 and IP38, respectively. Activity against the SBL KPC-2 was tested to investigate selectivity of the PyCs for MBLs over SBLs.

**Table 2:** MIC (mg/L) Values of Meropenem in Combination with Selected Pyrrole 2-Carboxylate Inhibitors against an Isogenic Panel of *E. coli* Strains Overexpressing Various Clinically Relevant MBLs and the SBL KPC-2^a^

			inhibitor 4 mg/L + MEM 32–0.03 mg/L														
strain	β-lactamase	MEM	**4a**	**4b**	**6**	**7**	**8**	**9**	**10**	**11**	**12**	**13**	**14**	**15**	**16**	**17**	**18**
IP38	-	≤0.03	≤0.03	≤0.03	≤0.03	≤0.03	≤0.03	≤0.03	≤0.03	≤0.03	≤0.03	≤0.03	≤0.03	≤0.03	≤0.03	≤0.03	≤0.03
IP42	KPC-2	8	4	4	8	8	8	8	8	8	8	8	8	4	8	4	8
IP93	NDM-1	>32	8	>32	4	4	4	2	8	8	4	8	4	4	8	4	2
IP90	VIM-1	16	8	16	1	1	2	1	2	2	4	1	1	1	1	8	2
IP41	VIM-2	4	0.25	2	0.06	0.25	0.25	0.125	0.125	0.25	0.5	0.125	0.125	0.25	0.25	0.5	0.25

a Results are representative of at least two independent experiments.

The inhibitors **PyC 4a–b**, **6–18**, were tested at a fixed concentration of 4 mg/L, with Meropenem being serially diluted from 32 to 0.03 mg/L. All the evaluated inhibitors, with the exception of **PyC 4b**, clearly potentiated the activity of Meropenem against the NDM-1-producing strain, as evidenced by significant reductions (≥8-fold) in MICs (Table 2). As anticipated, no activity was observed against the KPC-2 producing strain, consistent with the PyC specificity for MBLs over SBLs based on the active site binding of PyCs to metalloenzymes. Among the tested PyCs, **PyC 9** and **18** demonstrated the highest potentiation against the NDM-1 producing strain, reducing the MIC of Meropenem by at least 32-fold compared to Meropenem alone. **PyC 6**, **7**, **8**, **12**, **14**, **15**, and **17** were also strong potentiators, decreasing MIC of Meropenem by 16-fold in the same NDM-1 isogenic strain. **PyC 9**, **13**, and **14** exhibited a 16-fold and 32-fold MIC reduction against VIM-1 and VIM-2 producing strains, respectively. Notably, **PyC 6** was the most potent of the tested PyCs against VIM-2, achieving a 64-fold decrease in Meropenem MIC, while also showing a 16-fold MIC reduction against VIM-1 and NDM-1 strain. Other inhibitors with a potent combined effect with Meropenem against VIM-1 included **PyC 7**, **15**, and **16**, each producing a 16-fold MIC reduction.

Based on the most potent MIC reductions of Meropenem observed in this controlled system, we selected compounds **PyC 6**, **7**, **9**, **10**, **12**, **13**, **14, 15**, and **18** for further evaluation against MBL-producing clinical isolates of different species, including carbapenem-resistant Enterobacterales (CRE), carbapenem-resistant *Pseudomonas aeruginosa* (CRPA), and carbapenem-resistant *Acinetobacter baumannii* (CRAB) (see SI Table S4 for details of the strains).^65^ **InC 58**, a broad-spectrum indole-2-carboxylate MBL inhibitor,^15,66^ was used as comparison with the PyC-Meropenem combinations against the clinical panel. Meropenem was tested against the MBL-producing clinical isolates using a dilution range from 128 to 0.125 mg/L and the MBL inhibitors were evaluated at fixed concentrations of 8 and 16 mg/L (Table 3 and SI Table 2), compared to 4 mg/L with the model strains. Concentrations of 8 and 16 mg/L were chosen for the PyC inhibitors due to their reduced activity versus MBLs compared with **InC 58**; increasing the MBL inhibitor concentration to 16 mg/L did not lead to a substantial improvement in MIC values (Table 3), with at most a 2-fold reduction observed compared to 8 mg/L (see SI Table S2).

**Table 3:** MIC (mg/L) Values of Meropenem in Combination with Selected Pyrrole 2-Carboxylate Inhibitors against MBL-Producing Clinically Derived Isolates^a^

				inhibitor 16 mg/L + MEM 128–0.125 mg/L									
strain	species	carbapenemase profile	MEM	**6**	**7**	**9**	**10**	**12**	**13**	**14**	**15**	**18**	**Inc.58**
ATCC 25922	*E. coli*		0.016	≤0.125	≤0.125	≤0.125	≤0.125	≤0.125	≤0.125	≤0.125	≤0.125	≤0.125	≤0.125
K1N	*K. pneumoniae*	NDM-1, OXA-181	128	4	4	4	2–4	2	4	4	8	2–4	0.5–1
K2N	*K. pneumoniae*	NDM-1	64	4	4	4	2–4	2	8	4	4	4	0.5–1
K8N	*K. pneumoniae*	NDM-7	128	4	8	4	2–4	2	4	4	4–8	2	0.125–0.25
E8N	*E. coli*	NDM-5	64	4	8	4	8	4	8	4	8	2	0.25
E10N	*E. coli*	NDM-5	64	2	2	1	1–2	1	2	2–4	2	1	≤0.125
E11N	*E. coli*	NDM-7	>128	16–32	32	32	32	16	32–64	32	32	16	2
C1A	*E. coli*	NDM-4	64	1	1–2	1	1	0.5–1	2	1	2	0.5	≤0.125
C5A	*C. sedlakii*	NDM-1	64	2	2	1–2	1	1	2	2	1–2	0.5–1	≤0.125
E5A	*E. hormaechei*	VIM-2	16	2	4	4	2–4	8	4	2	8	8	2
S4A	*S. marcescens*	NDM-1	128	4	4	2	2–4	2	4	4–8	2	4	0.25
B3H	*P. aeruginosa*	VIM-2	128	32	64	64	64	128	64	32	64	128	32
P43	*P. aeruginosa*	IMP-1	≥128	≥128	≥128	>128	64	64	64	64	≥128	≥128	≥128
A10K	*A. baumannii*	NDM-1, OXA-98	>128	16	16	16	32	16	64	16	32	8	2
IEC429	*A. baumannii*	IMP-1	128	64	64	64	64	32–64	64	64	64	64	64

a The results are representative of two independent experiments.

Overall, the tested PyCs showed limited activity against *P. aeruginosa* and *A. baumannii*, an observation which may be due to restricted cellular permeability and insufficient intracellular accumulation in these species affecting both the antibiotic and partner MBLI. In contrast, all inhibitors produced notable MIC reductions against clinical isolates of *E. coli*, *Klebsiella pneumoniae*, and *Citrobacter sedlakii*. The PyCs lowered the Meropenem MIC below the EUCAST clinical breakpoint for Enterobacterales strains (resistance defined as >8 mg/L according to EUCAST Clinical Breakpoint Tables v. 15.0), with an exception being the *E. coli* strain E11N. Compounds **12** and **18** showed the highest potency against NDM-producers and were overall the best of the PyC series. Meropenem MICs with the highly optimized **InC 58** were typically 4-fold lower than with **PyC 18**, an observation which likely reflects differences in enzyme potency and compound accumulation.

## 3 Discussion and Conclusions

The combined biochemical, crystallographic, and microbiological results described here reveal PyCs as a new class of B1 subclass MBL inhibitors, with high potency against isolated enzymes and selectivity over a structurally related human-MBL fold nuclease.^67,68^ Many inhibitors of nonheme metalloenzymes bind in a manner involving active site metal-ion chelation, often in a bidentate manner.^69^ Metal-coordination is apparently often important in obtaining sufficiently potent metalloenzyme inhibition, however can raise concerns regarding selectivity over other metalloenzymes, including in the case of MBL inhibitors, inhibition of MBL-fold enzymes which have important roles in human biology.^27^ We have shown the potential for active site binding MBL inhibitors that do not coordinate to the Zn­(II) ions;^47^ presently, however, such compounds do not manifest the sufficient breadth of MBL activity to justify further development. We have thus pursued the PyC/InC series^15,49^ which, as shown here, bind via a largely conserved mode, with coordination to a single Zn­(II) ion (Zn2) in a monodentate manner via their carboxylate and in a manner which does not displace the di-Zn­(II) ion bridging hydroxide (Figure 4). The bridging hydroxide is apparently held in place by a hydrogen bond with the pyrrole/indole NH in a manner stabilized by substitution of the adjacent PyC C5 carbon, as supported SAR studies in both the InC^15,48^ and, as reported here, the PyC series. Although differing in detail, the di-Zn­(II) ion binding mode of the PyC/InC B1 MBL inhibitors is somewhat reminiscent of that observed for C2 carboxamide indole inhibitors of the di-Mn­(II) ion dependent methionine aminopeptidase-2 (MetAP-2), where the C2 carboxamide oxygen coordinates to only one of the two active site Mn­(II) ions.^70^ There is scope for developing further classes of inhibitors of metal ion containing enzymes, which bind via interactions with metal ion coordinating waters/hydroxides or which coordinate in a monodentate manner, interaction modes that may help to achieve selective inhibition.

The PyC compounds engage conserved active site residues similar to that of previously reported indole carboxylate inhibitors^15^ (Figure 4). The crystallographic observations not only confirm the binding mode which does not involve displacement of the di-Zn­(II) bridging hydroxide, but also provide a structural rationale for the observed SAR. In particular, the C3 substituents on the pyrrole ring were shown to make a significant contribution to activity, likely by increasing the pIC_50_ through hydrogen-bond and water-bridging interactions with polar residues conserved in B1 subfamily MBLs (Figure 4). This is reflected in the pIC_50_ range of 7.4–8.4 for **PyC 7**–**10** and **15** against VIM-2 (Table 1).

An overall correlation was observed between the *in vitro* potency of the PyCs against isolated MBLs and their activity in live bacterial cells against the isogenic strains expressing NDM-1, VIM-1, and VIM-2 (Tables 1 and 2). In cell-based assays using the isogenic panel, many of the inhibitors (**PyC 7**, **8**, **12**, **14**, **15**, **17**), tested at 4 mg/L, restored Meropenem efficacy, resulted in an average 16-fold reduction in MIC values. Notably, **PyC 9** and **18** achieved at least 32-fold reductions in Meropenem MICs. These results demonstrate that the PyCs can permeate the bacterial outer membrane and reach their intracellular target.

In studies with clinical isolates, higher inhibitor concentrations (8–16 mg/L) were required to achieve significant MIC reductions, with **PyC 12** and **18** showing up to a 64-fold decrease in Meropenem MICs across most NDM-producing strains (Table 3). Despite good pIC_50_ values against isolated MBLs, the combinations of Meropenem with PyCs exhibited limited or no activity against clinical isolates of *P. aeruginosa* and *A. baumannii*, where the InCs demonstrate moderate potentiation (Table 3). This discrepancy between potency in biochemical assays and whole-cell activity highlights the well-documented^71–75^ challenges of translating enzyme inhibition into microbiological efficacy, particularly in pathogens with complex resistance mechanisms, including reduced permeability, and active efflux systems. The clinical strains used in this study include Gram-negative pathogens from the ESKAPE group, which are associated with high mortality rates and pose significant challenges in antimicrobial therapy.^13^ The inhibition of these strains by **PyC 12** and **18**, in combination with Meropenem, as well as their promising in vitro ADMET properties, opens up scope for the future development of the PyCs as clinically useful MBL inhibitors.

## 4 Experimental Section

### 4.1 General Methods, Reagents, and Materials

All reagents were from commercial sources (Sigma-Aldrich, Inc.; Fluorochem Ltd.; Tokyo Chemical Industries) and were used as received. Anhydrous solvents (Sigma-Aldrich, Inc.) were kept under a nitrogen atmosphere. Chromatographic purifications were performed using a Biotage Isolera One or Biotage Selekt purification machines (wavelengths monitored: 254 and 280 nm) equipped with prepacked silica gel or C18 Biotage Sfär Duo cartridges for flash column chromatography (FCC). HPLC grade solvents (Sigma-Aldrich Inc.) were used for purifications, reaction work-ups, and extractions. Microwave reactions were carried out using Biotage Initiator^+^ Microwave Synthesizer. Thin layer chromatography (TLC) employed Merck silica gel 60 F254 TLC plates that were visualized using UV light. A Waters UPLC-MS machine equipped with QDa and PDA detectors was used for monitoring reactions. A ScanVac Basic Freeze-Dryer was used for lyophilization of the solvent fractions from reverse-phase column chromatography. High-resolution mass spectrometry (HRMS) was performed using electrospray ionization (ESI) in the positive or negative ionization modes employing a Waters RDa benchtop TOF machine linked to an Acquity LC system in the direct infusion (loop injection) mode or a Thermo Exactive High-Resolution Orbitrap FTMS instrument. HRMS data are presented as mass-to-charge (*m*/*z*) ratios. HRMS (APCI^+^): High-Resolution Mass Spectrometry (HRMS) coupled with Atmospheric Pressure Chemical Ionization (APCI). Nuclear magnetic resonance (NMR) spectroscopy was performed using Bruker AVIII 700, AVIII 600, NEO 600, AVIIIHD 500, NEO 400 Nanobay, and AVIIIHD 400 Nanobay machines. Chemical shifts for ^1^H NMR are reported in parts per million (ppm) downfield from tetramethylsilane and are referenced to residual proton in the NMR solvent (CDCl_3_: δ = 7.26 ppm; DMSO-*d*_6_: δ = 2.50 ppm; MeOH-*d*_4_: 3.31 ppm). For ^13^C NMR, chemical shifts are reported in parts per million (ppm) in the scale relative to the NMR solvent (CDCl_3_: δ = 77.2 ppm; DMSO-*d*_6_: δ = 39.5 ppm; MeOH-*d*_4_: 49.0 ppm). NMR data are reported as follows: chemical shift, multiplicity (s: singlet, d: doublet, t: triplet, q: quartet, m: multiplet, br: broad signal, dd: doublet of doublets, tt: triplet of triplets, qd: quartet of doublets), coupling constant (*J*, Hz; accurate to 0.5 Hz), and integration. Multiplicities are reported as observed where appropriate, and ‘’ denotes an apparent splitting pattern. ^19^F NMR spectra were acquired without ^1^H decoupling and chemical shifts are reported in parts per million (ppm). 2D NMR experiments (COSY, HSQC, and HMBC) were used to elucidate structures when appropriate. NMR spectra are shown at the end of the Supporting Information. All final compounds were >95% pure; and the HPLC chromatograms are shown at the end of the Supporting Information. Melting points of crystalline compounds were determined using a Stuart SMP40 Automatic melting point apparatus.

### 4.2 General Procedure A for Electrophilic Iodination

To a solution of the requisite 4,5-substituted pyrrole-2-carboxylate ester derivative (1.0 equiv) in dry DMF (3.0 mL/mmol) under an inert atmosphere was added *N*-iodosuccinimide (3.0 equiv) at room temperature. The reaction mixture was stirred at room temperature for 3 h, diluted with EtOAc, then washed with brine. The organic layer was dried over Na_2_SO_4_, filtered, and concentrated *in vacuo*. The residue was purified by silica gel FCC to afford the desired iodo-compound.

### 4.3 General Procedure B for Suzuki–Miyaura Cross Coupling

In a 5 mL microwave vial, the requisite 3-iodo-1*H*-pyrrole-2-carboxylate ester derivative (1.0 equiv) and aryl boronic acid or pinacol ester (1.5 equiv), Pd­(dppf)­Cl_2_ (0.1 equiv) were placed under vacuum; the vial was then backfilled with N_2_. N_2_-purged 1,4-dioxane (8.0 mL/mmol) and 2 M aq Na_2_CO_3_ (2.0 mL/mmol, N_2_-purged with sonication) were then added to the vial. The sealed vial was heated at 90 °C in an oil bath for 4 h with stirring. After cooling to room temperature, the reaction mixture was filtered through a pad of Celite, eluting with EtOAc. The organic layer was then washed with 1 N aq. HCl (20 mL/mmol) and brine (×2), dried over Na_2_SO_4_, filtered, and concentrated *in vacuo*. The residue was purified by silica gel FCC to give the desired compound.

### 4.4 General Procedure C for Suzuki–Miyaura Cross Coupling

In a 5 mL microwave vial, the requisite 3-iodo-1*H*-pyrrole-2-carboxylate ester derivative (1.0 equiv) and aryl boronic acid or pinacol ester (1.5 equiv), Pd­(dppf)­Cl_2_ (0.1 equiv), and K_2_CO_3_ (4.0 equiv) were placed under a vacuum; the vial was then backfilled with N_2_. N_2_-purged 1,4-dioxane (6.0 mL/mmol) and water (1.5 mL/mmol, N_2_-purged with sonication) were then added to the vial. The sealed vial was heated at 90 °C in an oil bath for 4 h with stirring. After cooling to room temperature, the reaction mixture was filtered through a pad of Celite, eluting with EtOAc. The organic layer was then washed with 1 N aq HCl (20 mL/mmol) and brine (twice), dried over Na_2_SO_4_, filtered, and concentrated *in vacuo*. The residue was purified by silica gel FCC to give the desired compound.

### 4.5 General Procedure D for Ester Hydrolysis

A mixture of the requisite carboxylate ester (1.0 equiv), LiOH·H_2_O (5.0 equiv), THF (8.5 mL/mmol), EtOH (1.4 mL/mmol), and water (2.8 mL/mmol) was stirred at room temperature for 48 h. Upon completion of the reaction, the mixture was acidified to pH 2 with 2 N HCl and twice extracted with EtOAc. The combined organic extracts were dried over Na_2_SO_4_, filtered, concentrated *in vacuo*. The crude mixture was purified either by crystallization or reverse phase (C18 column) FCC (acetonitrile in H_2_O with 0.1% v/v formic acid, 0–100%), then lyophilized to give the purified product.

### 4.6 General Procedure E for Ester Hydrolysis

To a solution of the requisite carboxylate ester (1.0 equiv) in EtOH (7 mL/mmol) and THF (minimum volume to dissolve the ester), was added 4 M KOH (13 mL/mmol). The resultant mixture was stirred at room temperature for 12 h. Upon completion of reaction, the mixture was acidified to pH 2 with 4 N HCl and twice extracted with EtOAc. The combined organic extracts were dried over Na_2_SO_4_, filtered, and concentrated *in vacuo*. The crude mixture was purified by reverse phase (C18 column) FCC (acetonitrile in H_2_O with 0.1% v/v formic acid, 0–100%), and then lyophilized to give the purified product.

### 4.7 Synthesis of 5-Methyl-3-phenyl-1*H*-pyrrole-2-carboxylic Acid (**PyC 1**)

#### 4.7.1 Ethyl (4-Oxo-4-phenylbut-2-en-2-yl)­glycinate (**21**)

To a solution of 1-phenyl-1,3-butanedione (**19**) (1.0 g, 6.16 mmol, 1.0 equiv) in EtOH (9.2 mL) were added glycine ethyl ester hydrochloride (**20**) (1.29 g, 9.24 mmol, 1.5 equiv) and triethylamine (1.3 mL, 9.24 mmol, 1.5 equiv); the reaction mixture was stirred at room temperature for 48 h. The reaction mixture was concentrated *in vacuo* and the crude residue was diluted with water (15 mL). The formed precipitate was isolated by suction filtration, washed with water, then dried under vacuum to afford the desired β-keto-enamine **21** in 82% yield (1.25 g, 5.05 mmol), as an off-white amorphous solid.

^1^H NMR (400 MHz, CDCl_3_) δ 11.49 (br t, *J* = 6.2 Hz, 1H), 7.88–7.85 (m, 2H), 7.42–7.36 (m, 3H), 5.76 (s, 1H), 4.23 (q, *J* = 7.1 Hz, 2H), 4.07 (d, *J* = 6.1 Hz, 2H), 2.03 (s, 3H), 1.29 (t, *J* = 7.1 Hz, 3H). ^13^C NMR (101 MHz, CDCl_3_) δ 188.7, 169.1, 164.0, 140.2, 130.8, 128.2, 127.1, 93.4, 61.8, 45.0, 19.5, 14.3. HRMS (TOF, ESI^+^) *m*/*z*: [M + H]^+^ calcd. for C_14_H_18_NO_3_, 248.1281; found, 248.1282.

#### 4.7.2 Ethyl 5-Methyl-3-phenyl-1*H*-pyrrole-2-carboxylate (**22**)

To a solution of 21% w/w NaOEt in EtOH (3.8 mL, 10.12 mmol, 1.0 equiv) at room temperature, was added the β-keto-enamine **21** (2.5 g, 10.12 mmol, 1.0 equiv); the reaction mixture was heated to reflux for 2 h. After cooling to rt, the reaction mixture was slowly poured into water (50 mL). The aqueous phase was extracted with EtOAc (3 × 50 mL), and the combined organic extracts were dried over Na_2_SO_4_, filtered, and concentrated *in vacuo*. The crude residue was purified by silica gel FCC (EtOAc in cyclohexane, 0–30%) to afford the desired compound **22** in 22% yield (510 mg, 2.22 mmol) as a white amorphous solid.

^1^H NMR (400 MHz, CDCl_3_) δ 9.15 (br s, 1H), 7.57–7.54 (m, 2H), 7.38–7.34 (m, 2H), 7.31– 7.27 (m, 1H), 6.07 (‘dd’, *J* = 3.0, 0.8 Hz, 1H), 4.25 (q, *J* = 7.1 Hz, 2H), 2.34 (s, 3H), 1.25 (t, *J* = 7.1 Hz, 3H). ^13^C NMR (101 MHz, CDCl_3_) δ 161.3, 135.5, 133.0, 132.7, 129.6, 127.7, 126.9, 116.7, 111.2, 60.2, 14.4, 13.2. HRMS (TOF, ESI^+^) *m*/*z*: [M + Na]^+^ calcd. for C_14_H_15_NO_2_Na, 252.0995; found, 252.0996.

#### 4.7.3 5-Methyl-3-phenyl-1*H*-pyrrole-2-carboxylic Acid (**PyC 1**)

To a solution of the ethyl ester **22** (150 mg, 0.66 mmol, 1.0 equiv) in THF (4.4 mL) and EtOH (2.2 mL) was added 2.5 M NaOH (2.1 mL, 5.24 mmol, 8.0 equiv) at room temperature. The reaction mixture was stirred at room temperature for 16 h, then acidified with 2 M HCl and extracted with EtOAc (2 × 30 mL). The organic fractions were combined, dried over Na_2_SO_4_, filtered, then concentrated *in vacuo*. The residue was suspended in CH_2_Cl_2_ and the resulting precipitate was collected under suction filtration. The precipitate was washed with CH_2_Cl_2_ to afford the desired compound **PyC 1** in 44% yield (58 mg, 0.29 mmol), as a white crystalline solid.

M.p.: 145–146 °C. ^1^H NMR (500 MHz, DMSO-*d*_6_) δ 11.43 (br s, 1H), 7.50–7.48 (m, 2H), 7.32–7.28 (m, 2H), 7.23–7.20 (m, 1H), 5.98 (d, *J* = 2.6 Hz, 1H), 2.21 (s, 3H); ^13^C NMR (126 MHz, DMSO-*d*_6_) δ 161.9, 135.9, 132.5, 131.4, 129.2, 127.5, 126.2, 116.2, 110.2, 12.5. HRMS (TOF, ESI^–^) *m*/*z*: [M – H]^−^ calcd. for C_12_H_10_NO_2_, 200.0717; found, 200.0717.

### 4.8 Synthesis of 3-Phenyl-1*H*-pyrrole-2-carboxylic Acid (**PyC 2**)

#### 4.8.1 Methyl 3-Phenyl-1*H*-pyrrole-2-carboxylate (**25**)

Methyl 3-bromo-1*H*-pyrrole-2-carboxylate (**23**) (100 mg, 0.49 mmol, 1.0 equiv), phenyl boronic acid (**24**) (90 mg, 0.74 mmol, 1.5 equiv), potassium carbonate (271 mg, 1.96 mmol, 4.0 equiv) and Pd­(dppf)­Cl_2_·CH_2_Cl_2_ (40 mg, 0.05 mmol, 0.1 equiv) were mixed in a vial under an inert atmosphere. To this mixture were added 1,4-dioxane (2.0 mL, N_2_-purged) and water (0.5 mL, N_2_-purged with sonication). The sealed microwave vial containing the reaction mixture was heated at 100 °C in an oil bath for 12 h with stirring. After cooling to room temperature, the reaction mixture was filtered through a pad of Celite eluting with EtOAc. The organic layer was then washed with brine (20 mL × 2), dried over Na_2_SO_4_, filtered, and concentrated *in vacuo*. The residue was purified by silica gel FCC to afford the desired compound **25** in 84% yield (83 mg, 0.41 mmol) as a white crystalline solid. The spectral data are in accord with those reported in the literature.^76^

#### 4.8.2 3-Phenyl-1*H*-pyrrole-2-carboxylic Acid (**PyC 2**)

**General Procedure D** for ester hydrolysis was followed using methyl pyrrole ester **25** (150 mg, 0.75 mmol, 1.0 equiv) to afford the desired pyrrole carboxylic acid **PyC 2** in 43% yield (60 mg, 0.32 mmol) as a white crystalline solid.

M.p.: 166–167 °C (melted and charred). ^1^H NMR (600 MHz, DMSO*-d*_6_) δ 12.26 (s, 1H), 11.67 (s, 1H), 7.58–7.46 (m, 2H), 7.35–7.28 (m, 2H), 7.26–7.19 (m, 1H), 6.96 (t, *J* = 2.8 Hz, 1H), 6.25 (t, *J* = 2.5 Hz, 1H). ^13^C NMR (151 MHz, DMSO*-d*_6_) δ 162.0, 135.7, 130.6, 129.2, 127.5, 126.2, 122.3, 118.0, 111.4.^77^ HRMS (TOF, ESI^+^) *m*/*z*: [M + Na]^+^ calcd. for C_11_H_9_NO_2_Na, 210.0526; found, 210.0531.

### 4.9 Synthesis of 5-Acetyl-3-(2-hydroxypyridin-4-yl)-1*H*-pyrrole-2-carboxylic Acid (**PyC 3**)

#### 4.9.1 1-(*tert*-Butyl) 2-Methyl 3-(2-(benzyloxy)­pyridin-4-yl)-1*H*-pyrrole-1,2-dicarboxylate (**27**)

To solution of methyl 3-bromo-1*H*-pyrrole-2-carboxylate (**23**) (200 mg, 0.98 mmol, 1.0 equiv) and DMAP (6 mg, 0.050 mmol, 0.05 equiv) in anhyd CH_2_Cl_2_ (5.0 mL) was added triethylamine (205 μL, 1.47 mmol, 1.5 equiv) and Boc_2_O (300 mg, 1.37 mmol, 1.4 equiv). The clear yellow solution was stirred at rt for 18 h. The solution was diluted with EtOAc and washed with 1 M aq HCl (×2) and brine. The organic layer was dried over MgSO_4_, filtered and evaporated to dryness under reduced pressure. To the remaining yellow oil was added 2-(benzyloxy)-4-(4,4,5,5-tetramethyl-1,3,2-dioxaborolan-2-yl)­pyridine (**26**) (366 mg, 1.18 mmol, 1.2 equiv), Pd­(dppf)­Cl_2_·CH_2_Cl_2_ (80 mg, 0.098 mmol, 0.1 equiv) and Na_2_CO_3_ (312 mg, 1.18 mmol, 1.2 equiv) and the vial was purged with argon. 1,4-Dioxane and water (4:1, 5.0 mL) were added to the solids and argon was bubbled through the suspension for 10 min. The vial was placed in a preheated oil bath at 85 °C and stirred for 3 h. After cooling to rt, to the brown suspension were added aq saturated NH_4_Cl and EtOAc. Layers were separated and the organic layer was washed with water (×2) and brine, dried over MgSO_4_, filtered and evaporated under reduced pressure. The brown residue was purified by silica gel FCC (EtOAc in cyclohexane, 0–40%) to afford the desired compound **27** in 84% yield (338 mg, 0.83 mmol) as a yellow oil.

^1^H NMR (400 MHz, CDCl_3_) δ 8.15 (dd, *J* = 5.4, 0.8 Hz, 1H), 7.50–7.43 (m, 2H), 7.42–7.35 (m, 2H), 7.35–7.31 (m, 1H), 7.29 (d, *J* = 3.3 Hz, 1H), 6.97 (dd, *J* = 5.4, 1.5 Hz, 1H), 6.91–6.86 (m, 1H), 6.36 (d, *J* = 3.3 Hz, 1H), 5.40 (s, 2H), 3.85 (s, 3H), 1.59 (s, 9H). ^13^C NMR (101 MHz, CDCl_3_) δ 164.2, 163.4, 148.0, 147.0, 144.2, 137.5, 128.6, 128.1, 127.9, 127.9, 123.2, 122.3, 116.4, 111.2, 109.8, 85.6, 67.8, 52.8, 27.9. HRMS (ESI^+^) *m*/*z* [M + H]^+^ calculated for C_23_H_25_N_2_O_5_, 409.1763; found, 409.1761.

#### 4.9.2 1-(*tert*-Butyl) 2-Methyl 3-(2-(benzyloxy)­pyridin-4-yl)-5-bromo-1*H*-pyrrole-1,2-dicarboxylate (**29**)

To a solution of *N*-Boc-pyrrole **27** (60 mg, 0.15 mmol, 1.0 equiv) in anhyd THF (1.0 mL) at −78 °C, was added freshly prepared solution of LiTMP in THF (0.33 M, 0.57 mL, 1.3 equiv). The dark brown solution was stirred at −78 °C for 30 min; a solution of 1,2-dibromotetrachloroethane (**28**) (96 mg, 0.29 mmol, 2.0 equiv) in anhyd THF (0.5 mL) was then added dropwise. Color of the solution changed from brown to orange and it was stirred at −78 °C for 1 h. After warming up to −20 °C, the reaction was quenched with AcOH (100 μL) and warmed to rt. To the solution were added EtOAc and H_2_O; the layers were separated and the organic layer was washed with water (×1) and brine, dried over MgSO_4_ and evaporated under reduced pressure. The resulting residue was purified by silica gel FCC (EtOAc in cyclohexane, 0–40%) to afford the desired compound **29** in 61% yield (44 mg, 0.09 mmol) as a colorless oil.

^1^H NMR (400 MHz, CDCl_3_) δ 8.16 (dd, *J* = 5.3, 0.7 Hz, 1H), 7.51–7.44 (m, 2H), 7.41–7.35 (m, 2H), 7.35–7.28 (m, 1H), 6.95 (dd, *J* = 5.3, 1.5 Hz, 1H), 6.89–6.85 (m, 1H), 6.35 (s, 1H), 5.40 (s, 2H), 3.75 (s, 3H), 1.64 (s, 9H). ^13^C NMR (101 MHz, CDCl_3_) δ 163.9, 160.6, 147.8, 146.5, 144.1, 137.5, 130.3, 128.6, 128.1, 128.0, 122.3, 117.7, 115.2, 111.2, 106.3, 87.0, 67.8, 52.2, 27.7. HRMS (ESI^+^) *m*/*z* [M + H]^+^ calculated for C_23_H_24_^79^BrN_2_O_5_, 487.0869; found, 487.0876.

#### 4.9.3 Methyl 5-Acetyl-3-(2-hydroxypyridin-4-yl)-1*H*-pyrrole-2-carboxylate (**31**)

To a solution of bromo-pyrrole **29** (42 mg, 0.086 mmol, 1.0 equiv) and Pd­(PPh_3_)­Cl_2_ (6.0 mg, 0.009 mmol, 0.1 equiv) in anhydrous 1,4-dioxane (1.0 mL) was added 1-ethoxyvinyltributyltin (**30**) (38 μL, 0.11 mmol, 1.3 equiv) under argon atmosphere. The vial was placed in a preheated oil bath and the clear yellow solution was stirred at 100 °C for 2 h. The vial was then removed from the bath; to the resultant brown solution were added water (1 mL) and 36% aq. HCl (36 μL, 0.43 mmol, 5.0 equiv); the vial was then placed back in oil bath at 100 °C and the mixture was stirred for 2 h. The yellow solution was cooled to rt and evaporated to dryness under reduced pressure. The residue was purified by reverse phase (C18 column) FCC (acetonitrile in H_2_O, 5–80%), then lyophilized to afford the desired compound **31** in 80% yield (18 mg, 0.07 mmol) as a white amorphous solid.

^1^H NMR (400 MHz, DMSO-*d*_6_) δ 12.54 (s, 1H), 11.62 (br s, 1H), 7.36 (dd, *J* = 6.8, 0.7 Hz, 1H), 7.22 (d, *J* = 2.6 Hz, 1H), 6.48 (dd, *J* = 1.8, 0.7 Hz, 1H), 6.35 (dd, *J* = 6.8, 1.8 Hz, 1H), 3.76 (s, 3H), 2.47 (s, 3H). ^13^C NMR (101 MHz, DMSO-*d*_6_) δ 188.5, 162.3, 160.4, 146.7, 134.2, 133.6, 127.6, 123.2, 118.2, 117.2, 107.2, 51.8, 26.5. HRMS (ESI^+^) *m*/*z* [M + H]^+^ calculated for C_13_H_13_N_2_O_4_, 261.0875; found, 261.0879.

#### 4.9.4 5-Acetyl-3-(2-hydroxypyridin-4-yl)-1*H*-pyrrole-2-carboxylic Acid (**PyC 3**)

To a suspension of methyl ester **31** (18 mg, 0.07 mmol, 1.0 equiv) in a mixture of 1,4-dioxane and water (1:1, 1.0 mL) was added anhydrous LiOH (17 mg, 0.35 mmol, 10 equiv); the resultant turbid solution was stirred at rt for 24 h. The reaction mixture was then diluted with water, acidified with 1 M aq HCl to pH 3 and evaporated to dryness. The resulting white residue was dissolved in DMSO and subjected to purification by reverse phase (C18 column) FCC (acetonitrile in H_2_O, 5–80%), and then lyophilized to afford the desired carboxylic acid **PyC 3** in 47% yield (8 mg, 0.03 mmol) as a white amorphous solid.

^1^H NMR (400 MHz, DMSO-*d*_6_) δ 7.35 (dd, *J* = 6.8, 0.7 Hz, 1H), 7.17 (d, *J* = 2.6 Hz, 1H), 6.49 (dd, *J* = 1.8, 0.7 Hz, 1H), 6.36 (dd, *J* = 6.8, 1.8 Hz, 1H), 2.46 (s, 3H) (the exchangeable hydrogens are not observed in the δ 0–11 ppm region). ^13^C NMR (101 MHz, DMSO-*d*_6_) δ 188.4, 162.3, 161.5, 147.0, 134.1, 133.2, 127.1, 124.4, 118.3, 117.1, 107.3, 26.5. HRMS (ESI^+^) *m*/*z* [M + H]^+^ calculated for C_12_H_11_N_2_O_4_, 247.0719; found, 247.0727.

### 4.10 Synthesis of 5-Acetyl-4-methyl-3-phenyl-1*H*-pyrrole-2-carboxylic Acid (**PyC 4a**)

#### 4.10.1 Ethyl 4-Methyl-3-phenyl-1*H*-pyrrole-2-carboxylate (**34**)

To a stirred solution of ethyl 2-isocyanoacetate (**33**) (0.74 mL, 6.74 mmol, 1.1 equiv) and *trans*-β-methyl-β-nitrostyrene (**32**) (1.0 g, 6.13 mmol, 1.0 equiv) in THF (7.4 mL) and ^*i*^PrOH (2.5 mL) was added DBU (1.8 mL, 12.26 mmol, 2.0 equiv) dropwise, while maintaining the reaction temperature between 10–20 °C using a mixture of ice in water. The reaction mixture was then left to stir at room temperature for 4 h. The resultant mixture was concentrated *in vacuo* and the crude was treated with water and extracted with Et_2_O (3 × 50 mL). The combined organic extracts were dried over Na_2_SO_4_, filtered and concentrated *in vacuo*. The crude residue was purified by silica gel FCC (EtOAc in cyclohexane, 0–20%) to afford the desired compound **34** in 88% yield (1.2 g, 5.39 mmol) as a white amorphous solid.

^1^H NMR (400 MHz, CDCl_3_) δ 9.11 (br s, 1H), 7.41–7.29 (m, 5H), 6.79–6.78 (m, 1H), 4.17 (q, *J* = 7.1 Hz, 2H), 2.01 (d, *J* = 0.9 Hz, 3H), 1.14 (t, *J* = 7.1 Hz, 3H); ^13^C NMR (101 MHz, CDCl_3_) δ 161.4, 134.9, 131.2, 130.4, 127.6, 126.8, 120.5, 120.5, 119.0, 60.1, 14.2, 10.7. HRMS (APCI^+^) *m*/*z*: [M + H]^+^ calcd. for C_14_H_15_NO_2_, 229.1097; found, 229.1092.

#### 4.10.2 Ethyl 5-Acetyl-4-methyl-3-phenyl-1*H*-pyrrole-2-carboxylate (**36**)

To a solution of acetic anhydride (0.17 mL, 1.74 mmol, 2.0 equiv) in CH_2_Cl_2_ (5.0 mL) at 0 °C, boron trifluoride etherate (BF_3_·OEt_2_) (0.16 mL, 1.31 mmol, 1.5 equiv) was added dropwise and stirred at 0 °C for 10 min. To the resultant mixture, pyrrole **34** (200 mg, 0.87 mmol, 1.0 equiv) was added portionwise; the mixture stirred at 0 °C for 30 min, then at room temperature for 2 h. The reaction mixture was poured into water and extracted with CH_2_Cl_2_ (3 × 30 mL). The organic fractions were washed with saturated NaHCO_3_, dried over Na_2_SO_4_, filtered and concentrated *in vacuo*. The residue obtained was purified by silica gel FCC (EtOAc in cyclohexane, 0–20%) to afford the desired compound **36** in 92% yield (217 mg, 0.80 mmol) as an orange amorphous solid.

^1^H NMR (400 MHz, CDCl_3_) δ 9.81 (br s, 1H), 7.42–7.32 (m, 3H), 7.27–7.25 (m, 2H), 4.18 (q, *J* = 7.1 Hz, 2H), 2.54 (s, 3H), 2.24 (s, 3H), 1.14 (t, *J* = 7.2 Hz, 3H); ^13^C NMR (101 MHz, CDCl_3_) δ 188.9, 160.5, 133.6, 132.4, 130.9, 130.6, 127.8, 127.4, 125.4, 122.3, 60.9, 28.9, 14.1, 12.1. HRMS (TOF, ESI^+^) *m*/*z*: [M + H]^+^ calcd. for C_16_H_18_NO_3_, 272.1281; found, 272.1279.

#### 4.10.3 5-Acetyl-4-methyl-3-phenyl-1*H*-pyrrole-2-carboxylic acid (**PyC 4a**)

To a stirred solution of the ethyl ester **36** (200 mg, 0.74 mmol, 1.0 equiv) in THF/H_2_O (1:2, 7 mL) was added KOH (160 mg, 2.95 mmol, 4.0 equiv); the resultant mixture was heated at 50 °C for 24 h. Upon completion of the reaction, the mixture was cooled to room temperature, then acidified to pH 1 with 2 N HCl and the resultant mixture was extracted with EtOAc (3 × 15 mL); the combined organic extracts were dried over Na_2_SO_4_, filtered, and concentrated *in vacuo*. The crude residue was purified by silica gel FCC (MeOH in CH_2_Cl_2_, 0–8%) to afford the desired compound **PyC 4a** in 50% yield (89 mg, 0.37 mmol) as an off-white crystalline solid.

M.p.: 186–189 °C (melted and charred). ^1^H NMR (600 MHz, DMSO-*d*_6_) δ 12.69 (br s, 1H), 11.86 (br s, 1H), 7.36 (t, *J* = 7.4 Hz, 2H), 7.30 (t, *J* = 7.2 Hz, 1H), 7.23 (d, *J* = 7.3 Hz, 2H), 2.53 (s, 3H), 2.09 (s, 3H); ^13^C NMR (151 MHz, DMSO-*d*_6_) δ 189.9, 161.7, 134.1, 130.8, 130.4, 130.4 127.6, 126.7, 125.3, 122.5, 28.5, 11.4. HRMS (TOF, ESI^+^) *m*/*z*: [M + H]^+^ calcd. for C_14_H_14_NO_3_, 244.0968; found, 244.0969.

### 4.11 Synthesis of 5-Acetyl-1,4-dimethyl-3-phenyl-1*H*-pyrrole-2-carboxylic Acid (**PyC 4b**)

#### 4.11.1 Ethyl 1,4-Dimethyl-3-phenyl-1*H*-pyrrole-2-carboxylate (**35**)

To a stirred suspension of the pyrrole **34** (250 mg, 1.09 mmol, 1.0 equiv) and K_2_CO_3_ (300 mg, 2.18 mmol, 2.0 equiv) in DMF/1,4-dioxane (1:1, 4 mL) was added CH_3_I (0.48 mL, 7.64 mmol, 7.0 equiv); the resultant mixture was heated at 90 °C for 7 h. The reaction mixture was cooled to room temperature, treated with water and extracted with EtOAc (3 × 30 mL). The combined organic extracts were dried over Na_2_SO_4_, filtered and concentrated *in vacuo*. The obtained residue was purified by silica gel FCC (EtOAc in cyclohexane, 0–10%) to afford the desired compound **35** in 85% yield (225 mg, 0.93 mmol), as a colorless oil.

^1^H NMR (400 MHz, CDCl_3_) δ 7.40–7.35 (m, 2H), 7.33–7.26 (m, 3H), 6.65 (s, 1H), 4.06 (q, *J* = 7.1 Hz, 2H), 3.93 (s, 3H), 1.94 (d, *J* = 0.8 Hz, 3H), 0.97 (t, *J* = 7.1 Hz, 3H); ^13^C NMR (101 MHz, CDCl_3_) δ 161.9, 136.5, 133.0, 130.3, 127.4, 127.3, 126.4, 119.6, 117.9, 59.5, 37.2, 13.8, 10.3; HRMS (TOF, ESI^+^) *m*/*z*: [M + H]^+^ calcd. for C_15_H_18_NO_2_ 244.1332; found, 244.1334.

#### 4.11.2 Ethyl 5-Acetyl-1,4-dimethyl-3-phenyl-1*H*-pyrrole-2-carboxylate (**37**)

To a solution of acetic anhydride (0.16 mL, 1.65 mmol, 2.0 equiv) in CH_2_Cl_2_ (5.0 mL) at 0 °C was added BF_3_·OEt_2_ (0.15 mL, 1.23 mmol, 1.5 equiv) dropwise; the resultant mixture was stirred at 0 °C for 10 min. Pyrrole **35** (200 mg, 0.82 mmol, 1.0 equiv) was then added portionwise, and the reaction was stirred at 0 °C for 30 min and at room temperature for 2 h. The reaction was quenched with water and extracted with CH_2_Cl_2_ (3 × 30 mL). The combined organic extracts were washed with saturated NaHCO_3_, dried over Na_2_SO_4_, filtered, and concentrated *in vacuo*. The residue obtained was purified by silica gel FCC (EtOAc in cyclohexane, 0–20%) to afford the desired compound **37** in 96% yield (226 mg, 0.79 mmol), as a white crystalline solid.

M.p.: 87–89 °C. ^1^H NMR (400 MHz, CDCl_3_) δ 7.38–7.29 (m, 3H), 7.17–7.14 (m, 2H), 4.08 (s, 3H), 4.01 (q, *J* = 7.1 Hz, 2H), 2.54 (s, 3H), 2.15 (s, 3H), 0.86 (t, *J* = 7.1 Hz, 3H); ^13^C NMR (101 MHz, CDCl_3_) δ 191.5, 161.8, 135.3, 133.3, 131.6, 130.3, 127.8, 127.0, 126.2, 125.3, 60.6, 35.7, 31.7, 13.5, 13.1. HRMS (APCI^+^) *m*/*z*: [M + H]^+^ calcd. for C_17_H_20_NO_3_, 286.1438; found, 286.1438.

#### 4.11.3 5-Acetyl-1,4-dimethyl-3-phenyl-1*H*-pyrrole-2-carboxylic Acid (**PyC 4b**)

To a stirred solution of ethyl ester **37** (200 mg, 0.70 mmol, 1.0 equiv) in THF/H_2_O (1:2, 9.0 mL) was added KOH (160 mg, 2.8 mmol, 4.0 equiv); the resultant mixture was heated at 50 °C for 24 h. Upon completion of the reaction, the mixture was cooled to room temperature and acidified to pH 1 with 2 N HCl. The resulting mixture was extracted with EtOAc (3 × 30 mL), and the combined organic layers were dried over Na_2_SO_4_, filtered, and concentrated *in vacuo*. The crude residue was purified by silica gel FCC (MeOH in CH_2_Cl_2_, 0–10%) to afford the desired compound **PyC 4b** in 79% yield (143 mg, 0.55 mmol) as a yellow crystalline solid.

M.p.: 152–153 °C. ^1^H NMR (400 MHz, DMSO-*d*_6_) δ 12.82 (br s, 1H), 7.40–7.35 (m, 2H), 7.33–7.28 (m, 1H), 7.18–7.15 (m, 2H), 3.93 (s, 3H), 2.50 (s, 3H, obscured by DMSO signal; confirmed by HSQC), 2.08 (s, 3H); ^13^C NMR (126 MHz, DMSO-*d*_6_) δ 191.0, 162.6, 134.7, 132.5, 130.1, 129.6, 127.8, 126.7, 126.6, 124.4, 35.3, 31.3, 12.4. HRMS (TOF, ESI^–^) *m*/*z*: [M – H]^−^ calcd. for C_15_H_14_NO_3_, 256.0979; found, 256.0978.

### 4.12 Synthesis of 3-(3,5-Dichlorophenyl)-4-methyl-5-phenyl-1*H*-pyrrole-2-carboxylic Acid (**PyC 6**)

#### 4.12.1 Methyl 4-Methyl-5-phenyl-1*H*-pyrrole-2-carboxylate (**40a**)

α-Methyl-*trans*-cinnamaldehyde (**38a**) (0.50 mL, 3.6 mmol, 1.0 equiv) was added dropwise to a solution of NaOMe (0.5 N in MeOH, 10.8 mL, 5.4 mmol, 1.5 equiv) at −20 °C. Methyl 2-azidoacetate (**39a**) (1.4 mL, 14.3 mmol, 4.0 equiv) was then added dropwise at −20 °C over several minutes; the reaction mixture was warmed to 0 °C and stirred for 4 h at 0 °C. The reaction mixture was concentrated *in vacuo*, then H_2_O (200 mL) followed by Et_2_O (200 mL) were added. The organic phase was separated and the aqueous phase was extracted with Et_2_O (2 × 200 mL). The combined organic layers were washed with brine, dried over MgSO_4_, filtered, and concentrated *in vacuo*. The crude mixture was purified by silica gel FCC (CH_2_Cl_2_ in cyclohexane with 1% v/v NEt_3_, 0–20%). The product, obtained as a mixture with the starting material, was combined, concentrated *in vacuo*, and resubjected to column purification (CH_2_Cl_2_/cyclohexane, 0–25%) to afford the desired compound **40a** in 37% yield (287 mg, 1.33 mmol) as a white crystalline solid.

M.p.: 114–116 °C. ^1^H NMR (400 MHz, CDCl_3_) δ 9.57 (br s, 1H), 7.53–7.51 (m, 2H), 7.45–7.41 (m, 2H), 7.35–7.30 (m, 1H), 6.82 (d, *J* = 2.6 Hz, 1H), 3.81 (s, 3H), 2.26 (s, 3H); ^13^C NMR (101 MHz, CDCl_3_) δ 162.0, 134.1, 132.4, 128.8, 127.5, 127.2, 121.2, 118.4, 118.3, 51.6, 12.6. HRMS (TOF, ESI^+^) *m*/*z*: [M + H]^+^ calcd. for C_13_H_14_NO_2_, 216.1019; found, 216.1019.

#### 4.12.2 Methyl 3-Iodo-4-methyl-5-phenyl-1*H*-pyrrole-2-carboxylate (**41a**)

Following **General Procedure A**, pyrrole **40a** (720 mg, 3.33 mmol, 1.0 equiv) was subjected to iodination. Purification by silica gel FCC (EtOAc in cyclohexane, 0–10%) afforded the desired compound **41a** in 69% yield (774 mg, 2.27 mmol) as a white crystalline solid.

M.p.: 147–149 °C. ^1^H NMR (400 MHz, CDCl_3_) δ 9.22 (br s, 1H), 7.48–7.43 (m, 4H), 7.39–7.35 (m, 1H), 3.90 (s, 3H), 2.21 (s, 3H); ^13^C NMR (101 MHz, CDCl_3_) δ 160.8, 133.8, 131.8, 129.0, 128.3, 127.6, 123.0, 122.2, 77.6, 51.7, 14.4. HRMS (TOF, ESI^–^) *m*/*z*: [M – H]^−^ calcd. for C_13_H_11_INO_2_, 339.9840; found, 339.9840.

#### 4.12.3 Methyl 3-(3,5-Dichlorophenyl)-4-methyl-5-phenyl-1*H*-pyrrole-2-carboxylate (**6a**)

A mixture of iodo-pyrrole **41a** (750 mg, 2.19 mmol, 1.0 equiv), 3,5-dichlorophenylboronic acid (**42a**) (500 mg, 2.62 mmol, 1.2 equiv), and 2 M aqueous Na_2_CO_3_ (4.4 mL) in 1,4-dioxane (20.0 mL) was purged with argon, and Pd­(dppf)­Cl_2_ (69 mg, 0.09 mmol, 0.04 equiv) was added. After addition of the catalyst, the reaction mixture was again purged with argon, then heated at 80 °C for 4 h. Upon completion of the reaction, the mixture was cooled to room temperature and filtered through a pad of Decalite, eluting with EtOAc and water. The filtrate was partitioned between 1 M HCl and EtOAc, and the organic layer was dried over Na_2_SO_4_, filtered, and concentrated *in vacuo*. The crude residue was purified by silica gel FCC (EtOAc in cyclohexane, 0–15%) to obtain the desired compound **6a** in 94% yield (742 mg, 2.06 mmol) as a white crystalline solid.

M.p.: 164–166 °C. ^1^H NMR (600 MHz, DMSO-*d*_6_) δ 12.02 (s, 1H), 7.60–7.53 (m, 3H), 7.50–7.43 (m, 2H), 7.39–7.33 (m, 3H), 3.65 (s, 3H), 1.99 (s, 3H); ^13^C NMR (151 MHz, DMSO-*d*_6_) δ 160.4, 138.7, 133.6, 133.1, 131.5, 128.9, 128.9, 128.4, 128.2, 127.5, 126.2, 118.0, 116.6, 50.9, 10.6. HRMS (TOF, ESI^+^) *m*/*z*: [M + Na]^+^ calcd. for C_19_H_15_NO_2_^35^Cl_2_Na, 382.03721; found, 382.03717.

#### 4.12.4 3-(3,5-Dichlorophenyl)-4-methyl-5-phenyl-1*H*-pyrrole-2-carboxylic Acid (**PyC 6**)

To a solution of methyl ester **6a** (200 mg, 0.56 mmol, 1.0 equiv) in THF (2.4 mL), MeOH (1.0 mL), and H_2_O (1.0 mL) was added LiOH·H_2_O (117 mg, 2.78 mmol, 5.0 equiv); the reaction mixture was stirred at room temperature for 48 h. Upon completion of the reaction, the mixture was acidified to pH 2 with 2 M HCl and extracted with EtOAc (2 × 30 mL). The combined organic extracts were dried over Na_2_SO_4_, filtered, and concentrated *in vacuo*. The residue was purified by silica gel FCC (MeOH in CH_2_Cl_2_, 0–5%) to obtain the desired compound **PyC 6** in 87% yield (174 mg, 0.48 mmol) as a light pink crystalline solid.

M.p.: 167–169 °C. ^1^H NMR (600 MHz, DMSO-*d*_6_) δ 12.28 (s, 1H), 11.84 (s, 1H), 7.58–7.56 (m, 2H), 7.53 (t, *J* = 1.9 Hz, 1H), 7.46–7.44 (m, 2H), 7.36–7.33 (m, 3H), 1.99 (s, 3H); ^13^C NMR (151 MHz, DMSO-*d*_6_) δ 161.6, 139.1, 133.0, 132.9, 131.7, 129.1, 128.3, 128.2, 128.1, 127.2, 126.0, 119.3, 116.4, 10.7. HRMS (TOF, ESI^–^) *m*/*z*: [M – H]^−^ calcd. for C_18_H_12_NO_2_^35^Cl_2_, 344.0251; found, 344.0246.

### 4.13 2-(3-Fluoro-4-((methylsulfonyl)­methyl)­phenyl)-4,4,5,5-tetramethyl-1,3,2-dioxaborolane (**42b**)

A round-bottom flask containing 4-bromo-2-fluoro-1-((methylsulfonyl)­methyl)­benzene^15^ (50 mg, 0.19 mmol, 1.0 equiv), bis­(pinacolato)­diboron (95 mg, 0.37 mmol, 2.0 equiv), Pd­(PPh_3_)_2_Cl_2_ (8 mg, 0.01 mmol, 0.06 equiv), and KOAc (73 mg, 0.75 mmol, 4.0 equiv) was purged with N_2_. Anhydrous 1,4-dioxane (7.0 mL, N_2_-purged) was then added and the reaction mixture was heated at 90 °C for 12 h. After cooling to room temperature, the reaction mixture was filtered through Celite, eluting with EtOAc. The filtrate was washed with brine and the organic layer was separated; the aqueous phase was extracted with EtOAc (2 × 30 mL). The combined organic extracts were dried over Na_2_SO_4_, filtered, and concentrated *in vacuo*. The crude residue was purified by silica gel FCC (EtOAc in cyclohexane, 10–50%) to afford the product **S1** in 86% yield (51 mg, 0.161 mmol) as a white crystalline solid.

M.p.: 123–125 °C. ^1^H NMR (600 MHz, DMSO-*d*_6_) δ 7.54–7.50 (m, 2H), 7.41 (“d”, *J* = 9.9 Hz, 1H), 4.58 (s, 2H), 3.00 (s, 3H), 1.29 (s, 12H); ^13^C NMR (151 MHz, DMSO-*d*_6_) δ 160.6 (d, *J*_C–F_ = 249.6 Hz), 133.0 (d, *J*_C–F_ = 2.4 Hz), 130.3 (d, *J*_C–F_ = 3.3 Hz), 120.4 (d, *J*_C–F_ = 19.8 Hz), 119.5 (d, *J*_C–F_ = 15.2 Hz), 84.1, 53.2, 40.0, 24.6 (ArCB was not observed).^78^ ^19^F NMR (565 MHz, DMSO-*d*_6_) δ −116.67 (dd, *J*_F–H_ = 9.5, 6.4 Hz). HRMS (TOF, ESI^+^) *m*/*z*: [M + H]^+^ calcd. for C_14_H_21_BFO_4_S, 315.1232; found, 315.1227.

### 4.14 Synthesis of 3-(3-Fluoro-4-((methylsulfonyl)­methyl)­phenyl)-4-methyl-5-phenyl-1*H*-pyrrole-2-carboxylic Acid (**PyC 7**)

#### 4.14.1 Ethyl 4-Methyl-5-phenyl-1*H*-pyrrole-2-carboxylate (**40b**)

A solution of α-methyl-*trans*-cinnamaldehyde (**38a**) (1.43 mL, 10.1 mmol, 1.0 equiv) in anhyd EtOH (5.0 mL) was added dropwise to a cooled solution (−20 °C, acetonitrile/dry ice bath) of 21% w/w NaOEt in EtOH (5.6 mL, 15.1 mmol, 1.5 equiv). 25% w/w ethyl 2-azidoacetate (**39b**) in EtOH (24.0 mL, 40.2 mmol, 4.0 equiv) was then added dropwise over 1 h in 5 min intervals to the mixture. The reaction mixture was warmed to −10 °C and stirred at this temperature for 4 h, then at room temperature for 1 h. The reaction was quenched with water (100 mL) and diluted with EtOAc (125 mL). The organic layer was separated, and the aqueous layer was extracted with EtOAc (2 × 125 mL). The combined organic extracts were dried over Na_2_SO_4_, filtered, and concentrated *in vacuo*. The resulting residue was purified by silica gel FCC (EtOAc in cyclohexane, 0–10%) to give the desired compound **40b** in 54% yield (1.25 g, 5.45 mmol) as a bright yellow crystalline solid.

M.p.: 94–98 °C. ^1^H NMR (400 MHz, CDCl_3_) δ 10.09 (br s, 1H), 7.59–7.56 (m, 2H), 7.46–7.43 (m, 2H), 7.36–7.32 (m, 1H), 6.88 (d, *J* = 2.6 Hz, 1H), 4.27 (q, *J* = 7.1 Hz, 2H), 2.30 (s, 3H), 1.34 (t, *J* = 7.1 Hz, 3H); ^13^C NMR (151 MHz, CDCl_3_) δ 161.4, 133.6, 132.4, 129.0, 127.5, 127.1, 121.6, 118.4, 118.2, 60.4, 14.6, 12.6. HRMS (TOF, ESI^+^) *m*/*z*: [M + H]^+^ calcd. for C_14_H_16_NO_2_, 230.1176; found, 230.1175.

#### 4.14.2 Ethyl 3-Iodo-4-methyl-5-phenyl-1*H*-pyrrole-2-carboxylate (**41b**)

Following **General Procedure A**, compound **40b** (128 mg, 0.56 mmol, 1.0 equiv) was subjected to iodination. Purification by silica gel FCC (EtOAc in cyclohexane, 0–10%) afforded the desired compound **41b** in 92% yield (182 mg, 0.51 mmol) as a beige crystalline solid.

M.p: 182–185 °C. ^1^H NMR (400 MHz, CDCl_3_) δ 9.50 (br s, 1H), 7.45–7.44 (m, 4H), 7.39–7.34 (m, 1H), 4.32 (q, *J* = 7.1 Hz, 2H), 2.21 (s, 3H), 1.39 (t, *J* = 7.1 Hz, 3H); ^13^C NMR (101 MHz, CDCl_3_) δ 160.4, 133.7, 131.9, 129.0, 128.2, 127.6, 122.9, 122.3, 77.5, 60.9, 14.5, 14.4. HRMS (TOF, ESI^+^) *m*/*z*: [M + H]^+^ calcd. for C_14_H_15_INO_2_, 356.0142; found, 356.0139.

#### 4.14.3 Ethyl 3-(3-Fluoro-4-((methylsulfonyl)­methyl)­phenyl)-4-methyl-5-phenyl-1*H*-pyrrole-2-carboxylate (**7a**)

**General Procedure B** for Suzuki–Miyaura cross coupling was followed using iodo-pyrrole **41b** (84 mg, 0.24 mmol, 1.0 equiv) and pinacol-boronate ester **42b** (89 mg, 0.28 mmol, 1.2 equiv). Purification by silica gel FCC (EtOAc in cyclohexane, 0–40%) afforded the desired compound **7a** in 88% yield (86 mg, 0.21 mmol), as a white crystalline solid.

M.p.: 189–191 °C. ^1^H NMR (700 MHz, DMSO-*d*_6_) δ 11.94 (s, 1H), 7.59–7.57 (m, 2H), 7.49–7.45 (m, 3H), 7.37–7.35 (m, 1H), 7.24 (dd, *J* = 10.9, 1.6 Hz, 1H), 7.20 (dd, *J* = 7.8, 1.7 Hz, 1H), 4.59 (s, 2H), 4.09 (q, *J* = 7.1 Hz, 2H), 3.03 (s, 3H), 2.01 (s, 3H), 1.08 (t, *J* = 7.1 Hz, 3H); ^13^C NMR (176 MHz, DMSO-*d*_6_) δ 160.3, 160.2 (d, *J*_C–F_ = 246.4 Hz), 138.0 (d, *J*_C–F_ = 8.8 Hz), 133.5, 132.1 (d, *J*_C–F_ = 3.6 Hz), 131.7, 129.9, 128.4, 128.3, 127.4, 126.6 (d, *J*_C–F_ = 2.8 Hz), 118.3, 117.5 (d, *J*_C–F_ = 21.8 Hz), 116.5, 114.1 (d, *J*_C–F_ = 15.4 Hz), 59.5, 53.0, 40.0, 14.0, 10.9; ^19^F NMR (565 MHz, DMSO-*d*_6_) δ −117.42 to – 117.54 (m). HRMS (TOF, ESI^+^) *m*/*z*: [M + H]^+^ calcd. for C_22_H_23_FNO_4_S, 416.1326; found, 416.1326.

#### 4.14.4 3-(3-Fluoro-4-((methylsulfonyl)­methyl)­phenyl)-4-methyl-5-phenyl-1*H*-pyrrole-2-carboxylic Acid (**PyC 7**)

**General Procedure D** for ester hydrolysis was followed using ethyl ester **7a** (50 mg, 0.12 mmol, 1.0 equiv) to afford the desired carboxylic acid **PyC 7** in 48% yield (24 mg, 0.062 mmol) as a beige crystalline solid.

M.p.: 163–165 °C. ^1^H NMR (600 MHz, DMSO-*d*_6_) δ 12.13 (br s, 1H), 11.76 (br s, 1H), 7.58 (dd, J = 8.2, 1.4 Hz, 2H), 7.47–7.44 (m, 3H), 7.34 (tt, J = 7.2, 1.2 Hz, 1H), 7.23–7.19 (m, 2H), 4.57 (s, 2H), 3.04 (s, 3H), 2.00 (s, 3H); ^13^C NMR (151 MHz, DMSO-*d*_6_) δ 161.6, 160.1 (d, *J*_C–F_ = 246.6 Hz), 138.2, 132.8, 132.0 (d, *J*_C–F_ = 3.8 Hz), 131.8, 129.4, 128.3, 128.1, 127.1, 126.6, 117.4 (d, *J*_C–F_ = 21.6 Hz), 116.3, 113.8 (d, *J*_C–F_ = 15.6 Hz), 53.0, 40.1 (d, *J*_C–F_ = 2.6 Hz), 10.9 (one quaternary carbon was not observed); ^19^F NMR (377 MHz, DMSO-*d*_6_) δ −117.4 (dd, *J*_F–H_ = 12.0, 8.4 Hz). HRMS (TOF, ESI^+^) *m*/*z*: [M + H]^+^ calcd. for C_20_H_19_FNO_4_S, 388.1013; found, 388.1014.

### 4.15 1-(4-Bromophenyl)-*N*,*N*-dimethylmethanesulfonamide (**42c′**)

To a solution of (4-bromophenyl)­methanesulfonyl chloride (**42c″**) (1.00 g, 3.71 mmol, 1.0 equiv) in CH_2_Cl_2_ (20 mL) at 0 °C, were added anhyd pyridine (0.59 mL, 7.42 mmol, 2.0 equiv) and dimethylamine (2 M in THF, 2.23 mL, 4.45 mmol, 1.2 equiv). The reaction mixture was allowed to warm at room temperature and stirred for 16 h. After completion, the reaction was quenched by addition of H_2_O (200 mL), extracted with CH_2_Cl_2_ (2 × 200 mL), dried over Na_2_SO_4_, filtered and concentrated *in vacuo*. The combined extracts were purified by silica gel FCC (EtOAc in cyclohexane, 0–35%) to afford the desired compound **42c′** in 78% yield (800 mg, 2.88 mmol) as a yellow-white crystalline solid.

M.p.: 120–122 °C. ^1^H NMR (400 MHz, CDCl_3_) δ 7.43 (d, *J* = 8.4 Hz, 2H), 7.20 (d, *J* = 8.4 Hz, 2H), 4.08 (s, 2H), 2.68 (s, 6H); ^13^C NMR (101 MHz, CDCl_3_) δ 132.3, 132.0, 128.1, 123.0, 54.9, 37.8. HRMS (TOF, ESI^+^) *m*/*z*: [M + H]^+^ calcd. for C_9_H_13_^79^BrNO_2_S, 277.9845; found, 277.9838.

### 4.16 *N*,*N*-Dimethyl-1-(4-(4,4,5,5-tetramethyl-1,3,2-dioxaborolan-2-yl)­phenyl)­methane Sulfonamide (**42c**)

A round-bottom flask containing aryl bromide **42c′** (500 mg, 1.8 mmol, 1.0 equiv), B_2_Pin_2_ (914 mg, 3.60 mmol, 2.0 equiv), Pd­(PPh_3_)_2_Cl_2_ (76 mg, 0.11 mmol, 0.06 equiv), and KOAc (706 mg, 7.20 mmol, 4.0 equiv) was placed under an inert atmosphere. N_2_-purged anhyd 1,4-dioxane (60 mL) was added, and the reaction mixture was heated at 90 °C for 12 h. After cooling to room temperature, the reaction mixture was filtered through Celite, washing with EtOAc several times. The filtrate was washed with brine, the organic phase was separated, and the aqueous phase was extracted with EtOAc (2 × 100 mL). The combined organic extracts were dried over Na_2_SO_4_, filtered, and concentrated *in vacuo*. The crude residue was purified by silica gel FCC (EtOAc in cyclohexane, 0–50%) to afford the desired product **42c** in 85% yield (495 mg, 1.52 mmol) as a white crystalline solid.

M.p.: 124–126 °C. ^1^H NMR (600 MHz, CDCl_3_) δ 7.81 (d, *J* = 8.0 Hz, 2H), 7.40 (d, *J* = 8.0 Hz, 2H), 4.25 (s, 2H), 2.70 (s, 6H), 1.35 (s, 12H); ^13^C NMR (151 MHz, CDCl_3_) δ 135.3, 132.1, 130.1, 84.2, 56.4, 38.0, 25.0 (ArCB was not observed).^78^ HRMS (TOF, ESI^+^) *m*/*z*: [M + H]^+^ calcd. for C_15_H_25_BNO_4_S, 326.1592; found, 326.1584.

### 4.17 Synthesis of 3-(4-((*N*,*N*-Dimethylsulfamoyl)­methyl)­phenyl)-4-methyl-5-phenyl-1*H*-pyrrole-2-carboxylic Acid (**PyC 8**)

#### 4.17.1 Ethyl 3-(4-((*N*,*N*-Dimethylsulfamoyl)­methyl)­phenyl)-4-methyl-5-phenyl-1*H*-pyrrole-2-carboxylate (**8a**)

**General Procedure C** for Suzuki–Miyaura cross coupling was followed using iodo pyrrole **41b** (300 mg, 0.85 mmol, 1.0 equiv) and pinacol ester **42c** (412 mg, 1.27 mmol, 1.5 equiv). Purification by silica gel FCC (EtOAc in cyclohexane, 0–45%) afforded the desired compound **8a** in 67% yield (241 mg, 0.57 mmol) as a white crystalline solid.

M.p.: 175–178 °C. ^1^H NMR (500 MHz, DMSO-*d*_6_) δ 11.80 (br s, 1H), 7.60–7.58 (m, 2H), 7.47–7.42 (m, 4H), 7.37–7.31 (m, 3H), 4.45 (s, 2H), 4.06 (q, *J* = 7.1 Hz, 2H), 2.73 (s, 6H), 1.97 (s, 3H), 1.08 (t, *J* = 7.1 Hz, 3H); ^13^C NMR (126 MHz, DMSO-*d*_6_) δ 160.4, 135.0, 133.4, 131.8, 131.2, 130.3, 129.9, 128.3, 128.1, 127.6, 127.3, 118.2, 116.4, 59.2, 53.2, 37.4, 14.0, 10.8. HRMS (TOF, ESI^+^) *m*/*z*: [M + H]^+^ calcd. for C_23_H_27_N_2_O_4_S, 427.1686; found, 427.1686.

#### 4.17.2 3-(4-((*N*,*N*-Dimethylsulfamoyl)­methyl)­phenyl)-4-methyl-5-phenyl-1*H*-pyrrole-2-carboxylic Acid (**PyC 8**)

**General Procedure D** for ester hydrolysis was followed using ethyl ester **8a** (150 mg, 0.35 mmol, 1.0 equiv) to afford the desired carboxylic acid **PyC 8** in 44% yield (62 mg, 0.16 mmol) as a white crystalline solid.

M.p.: 148–150 °C. ^1^H NMR (600 MHz, DMSO-*d*_6_) δ 12.05 (br s, 1H), 11.61 (br s, 1H), 7.58 (“dt”, *J* = 8.2, 1.6 Hz, 2H), 7.45–7.43 (m, 2H), 7.40 (‘d’, *J* = 8.2 Hz, 2H), 7.34–7.31 (m, 3H), 4.43 (s, 2H), 2.72 (s, 6H), 1.97 (s, 3H); ^13^C NMR (151 MHz, DMSO-*d*_6_) δ 162.0, 135.3, 132.4, 132.4, 132.0, 130.4, 129.9, 128.3, 127.9, 127.3, 127.0, 116.2, 53.2, 37.4, 11.0 (one ArC quaternary was not observed). HRMS (TOF, ESI^+^) *m*/*z*: [M + H]^+^ calcd. for C_21_H_23_N_2_O_4_S, 399.1373; found, 399.1372.

### 4.18 Synthesis of 3-(4-Hydroxyphenyl)-4-methyl-5-phenyl-1*H*-pyrrole-2-carboxylic Acid (**PyC 9**)

#### 4.18.1 Ethyl 3-(4-Hydroxyphenyl)-4-methyl-5-phenyl-1*H*-pyrrole-2-carboxylate (**9a**)

**General Procedure C** for Suzuki–Miyaura cross coupling was followed using iodo-pyrrole **41b** (300 mg, 0.85 mmol, 1.0 equiv) and 4-hydroxyphenylboronic acid (**42d**) (175 mg, 1.27 mmol, 1.5 equiv). Purification by silica gel FCC (EtOAc in cyclohexane, 0–30%) afforded the desired compound **9a** in 66% yield (180 mg, 0.56 mmol) as a white crystalline solid.

M.p.: 190–192 °C. ^1^H NMR (500 MHz, DMSO-*d*_6_) δ 11.62 (s, 1H), 9.32 (s, 1H), 7.58 (“dd”, *J* = 8.4, 1.3 Hz, 2H), 7.46–7.43 (m, 2H), 7.35–7.32 (m, 1H), 7.10 (d, *J* = 8.5 Hz, 2H), 6.76 (d, *J* = 8.5 Hz, 2H), 4.07 (q, *J* = 7.1 Hz, 2H), 1.97 (s, 3H), 1.10 (t, *J* = 7.1 Hz, 3H); ^13^C NMR (126 MHz, DMSO-*d*_6_) δ 160.5, 156.0, 133.1, 132.0, 132.0, 131.4, 128.3, 128.1, 127.1, 125.3, 118.0, 116.5, 114.2, 59.0, 14.1, 11.0. HRMS (TOF, ESI^+^) *m*/*z*: [M + Na]^+^ calcd. for C_20_H_19_NO_3_Na, 344.1257; found, 344.1250.

#### 4.18.2 3-(4-Hydroxyphenyl)-4-methyl-5-phenyl-1*H*-pyrrole-2-carboxylic Acid (**PyC 9**)

**General Procedure D** for ester hydrolysis was followed using ethyl ester **9a** (80 mg, 0.25 mmol, 1.0 equiv) to afford the desired carboxylic acid **PyC 9** in 45% yield (33 mg, 0.11 mmol) as a white crystalline solid.

M.p.: 164–168 °C. ^1^H NMR (600 MHz, DMSO-*d*_6_) δ 11.85 (br s, 1H), 11.48 (br s, 1H), 9.29 (s, 1H), 7.58–7.57 (m, 2H), 7.44–7.42 (m, 2H), 7.33–7.30 (m, 1H), 7.09 (d, *J* = 8.5 Hz, 2H), 6.75 (d, *J* = 8.5 Hz, 2H), 1.96 (s, 3H); ^13^C NMR (151 MHz, DMSO-*d*_6_) δ 162.0, 155.9, 132.5, 132.1, 131.6, 131.4, 128.3, 127.9, 126.9, 125.6, 118.6, 116.4, 114.2, 11.1. HRMS (TOF, ESI^+^) *m*/*z*: [M + H]^+^ calcd. for C_18_H_16_NO_3_, 294.1125; found, 294.1117.

### 4.19 Synthesis of 3-(4-(Carboxymethyl)­phenyl)-4-methyl-5-phenyl-1*H*-pyrrole-2-carboxylic Acid (**PyC 11**)

#### 4.19.1 2-(4-(2-(Ethoxycarbonyl)-4-methyl-5-phenyl-1*H*-pyrrol-3-yl)­phenyl)­acetic Acid (**11a**)

**General Procedure C** for Suzuki–Miyaura cross coupling was followed using iodo-pyrrole **41b** (300 mg, 0.85 mmol, 1.0 equiv) and 4-(carboxymethyl)­phenyl boronic acid pinacol ester (**42e**) (332 mg, 1.27 mmol, 1.5 equiv). Purification by silica gel FCC (EtOAc in cyclohexane, 0–50%) afforded the desired compound **11a** in 72% yield (221 mg, 0.61 mmol) as a white crystalline solid.

M.p.: 198–200 °C. ^1^H NMR (500 MHz, DMSO-*d*_6_) δ 12.33 (br s, 1H), 11.74 (br s, 1H), 7.59–7.57 (m, 2H), 7.48–7.44 (m, 2H), 7.37–7.33 (m, 1H), 7.28–7.24 (m, 4H), 4.07 (q, *J* = 7.1 Hz, 2H), 3.60 (s, 2H), 1.99 (s, 3H), 1.09 (t, *J* = 7.1 Hz, 3H); ^13^C NMR (126 MHz, DMSO-*d*_6_) δ 172.8, 160.4, 133.2, 133.2, 133.1, 131.9, 131.5, 130.2, 128.4, 128.3, 128.1, 127.2, 118.1, 116.4, 59.2, 40.6, 14.0, 10.9. HRMS (TOF, ESI^+^) *m*/*z*: [M + Na]^+^ calcd. for C_22_H_21_NO_4_Na, 386.1363; found, 386.1361.

#### 4.19.2 3-(4-(Carboxymethyl)­phenyl)-4-methyl-5-phenyl-1*H*-pyrrole-2-carboxylic Acid (**PyC 11**)

**General Procedure E** for ester hydrolysis was followed using ethyl ester **11a** (134 mg, 0.37 mmol, 1.0 equiv) to afford the carboxylic acid **PyC 11** in 60% yield (74 mg, 0.22 mmol) as a white crystalline solid.

M.p.: 137–139 °C. ^1^H NMR (600 MHz, DMSO-*d*_6_) δ 12.16 (br s, 1H), 11.61 (br s, 1H), 7.59–7.57 (m, 2H), 7.45–7.42 (m, 2H), 7.34–7.31 (m, 1H), 7.25 (‘s’, 4H), 3.59 (s, 2H), 1.97 (s, 3H); ^13^C NMR (151 MHz, DMSO-*d*_6_) δ 172.7, 161.9, 133.5, 132.9, 132.6, 132.0, 131.1, 130.3, 128.4, 128.3, 128.0, 127.0, 118.8, 116.4, 40.5, 11.0. HRMS (TOF, ESI^+^) *m*/*z*: [M + H]^+^ calcd. for C_20_H_18_NO_4_, 336.1230; found, 336.1223.

#### 4.19.3 *tert*-Butyl 4-(4-(4,4,5,5-Tetramethyl-1,3,2-dioxaborolan-2-yl)­benzoyl)­piperazine-1-carboxylate (**42f**)

To a solution of 4-(4,4,5,5-tetramethyl-1,3,2-dioxaborolan-2-yl)­benzoic acid^79^ (**42f′**) (1.00 g, 4.03 mmol, 1.0 equiv) in CH_2_Cl_2_ (10.0 mL) was added diisopropylethylamine (2 M in NMP, 8.06 mL, 16.10 mmol, 4.0 equiv), followed by HBTU (1.83 g, 4.84 mmol, 1.2 equiv); the reaction mixture was stirred for 5 min at room temperature. The reaction mixture was cooled to 0 °C, and *N*-Boc-piperazine (826 mg, 4.43 mmol, 1.1 equiv) was then added portionwise to the reaction mixture, after which the mixture was stirred overnight at room temperature. After completion of reaction (as monitored by UPLC) saturated aq NaHCO_3_ and CH_2_Cl_2_ were added. The organic layer was separated, and the aqueous phase was extracted with CH_2_Cl_2_ (×2). The combined organic extracts were dried over Na_2_SO_4_, filtered, and concentrated *in vacuo*. The crude residue (orange gummy) was purified by silica gel FCC (EtOAc in cyclohexane, 0–20%) to afford the desired compound **42f** in 43% yield (714 mg, 1.71 mmol) as a white crystalline solid.

M.p.: 159–161 °C. ^1^H NMR (600 MHz, CDCl_3_) δ 7.85 (d, *J* = 7.9 Hz, 2H), 7.38 (d, *J* = 8.0 Hz, 2H), 3.74 (‘br s’, 2H), 3.51 (‘br s’, 2H), 3.35 (‘br s’, 4H), 1.46 (s, 9H), 1.35 (s, 12H); ^13^C NMR (151 MHz, CDCl_3_) δ 170.7, 154.7, 138.1, 135.1, 126.3, 84.2, 80.5, 47.6 (br), 42.1 (br), 28.5, 25.0 (ArCB was not observed).^78^ HRMS (TOF, ESI^+^) *m*/*z*: [M + Na]^+^ calcd. for C_22_H_33_BN_2_O_5_Na, 439.2375; found, 439.2389.

### 4.20 Synthesis of 4-Methyl-5-phenyl-3-(4-(piperazine-1-carbonyl)­phenyl)-1*H*-pyrrole-2-carboxylic Acid (**PyC 12**)

#### 4.20.1 *tert*-Butyl 4-(4-(2-(Ethoxycarbonyl)-4-methyl-5-phenyl-1*H*-pyrrol-3-yl)­benzoyl)­piperazine-1-carboxylate (**12a**)

**General Procedure C** for Suzuki–Miyaura cross coupling was followed using iodo-pyrrole **41b** (213 mg, 0.60 mmol, 1.0 equiv) and pinacol-ester **42f** (375 mg, 0.90 mmol, 1.5 equiv). Purification by silica gel FCC (EtOAc in cyclohexane, 0–35%) afforded the desired compound **12a** in 77% yield (240 mg, 0.46 mmol) as a white crystalline solid.

M.p.: 207–208 °C. ^1^H NMR (600 MHz, CDCl_3_) δ 9.18 (br s, 1H), 7.54–7.52 (m, 2H), 7.48–7.44 (m, 6H), 7.38–7.35 (m, 1H), 4.17 (q, *J* = 7.1 Hz, 2H), 3.74 (“br s”, 2H), 3.49 (“br s”, 6H), 2.10 (s, 3H), 1.48 (s, 9H), 1.14 (t, *J* = 7.1 Hz, 3H). ^13^C NMR (151 MHz, CDCl_3_) δ 171.0, 161.1, 154.7, 137.0, 133.8, 133.3, 132.2, 131.8, 130.8, 129.0, 127.9, 127.5, 126.6, 118.5, 117.8, 80.5, 60.3, 47.8 (br), 43.9 (br), 42.3 (br), 28.5, 14.3, 11.1. HRMS (TOF, ESI^+^) *m*/*z*: [M + H]^+^ calcd. for C_30_H_36_N_3_O_5_, 518.2649; found, 518.2671.

#### 4.20.2 *tert*-Butyl 4-(4-(2-((Benzyloxy)­carbonyl)-4-methyl-5-phenyl-1*H*-pyrrol-3-yl)­benzoyl)­piperazine-1-carboxylate (**12b**)

To a round-bottom flask containing ethyl ester **12a** (81 mg, 0.16 mmol, 1.0 equiv) under an inert atmosphere, was added anhyd benzyl alcohol (7.0 mL) and titanium­(IV) ethoxide (0.12 mL, 0.56 mmol, 3.6 equiv). The reaction mixture was heated for 20 h at 110 °C until complete consumption of the starting material was observed (as monitored by UPLC). After cooling, the reaction mixture was diluted with EtOAc (15 mL), and washed with water (30 mL). The organic layer was then dried over Na_2_SO_4_ and the volatiles were evaporated under reduced pressure to afford crude mixture with residual benzyl alcohol. The residue was purified by silica gel FCC (EtOAc in cyclohexane, 0–35%) to afford the desired compound **12b** in 59% yield (54 mg, 0.093 mmol) as a white crystalline solid.

M.p.: 194–196 °C. ^1^H NMR (600 MHz, DMSO-*d*_6_) δ 11.97 (s, 1H), 7.60 (“dd”, *J* = 8.2, 1.1 Hz, 2H), 7.48–7.46 (m, 2H), 7.39–7.35 (m, 5H), 7.31–7.29 (m, 2H), 7.27–7.24 (m, 1H), 7.14 (“d”, *J* = 7.0 Hz, 2H), 5.15 (s, 2H), 3.59 (“br s”, 2H), 3.37 (“br s”, 6H) (partly overlapped with H_2_O peak), 1.99 (s, 3H), 1.42 (s, 9H). ^13^C NMR (151 MHz, DMSO-*d*_6_) δ 169.3, 160.2, 153.8, 136.6, 136.3, 133.8, 133.7, 131.7, 131.2, 130.3, 128.4, 128.2, 128.2, 127.5, 127.4, 127.3, 126.4, 117.9, 116.8, 79.2, 64.7, 28.0, 10.8 (Piperazine –CH_2_– did not appear or too broad to detect). HRMS (TOF, ESI^+^) *m*/*z*: [M + H]^+^ calcd. for C_35_H_38_N_3_O_5_, 580.2806; found, 580.2797.

#### 4.20.3 4-Methyl-5-phenyl-3-(4-(piperazine-1-carbonyl)­phenyl)-1*H*-pyrrole-2-carboxylic Acid (**PyC 12**)

To a stirred solution of compound **12b** (21 mg, 0.04 mmol, 1.0 equiv) in CH_2_Cl_2_ (0.3 mL) was added trifluoracetic acid (0.3 mL); the reaction mixture was stirred at room temperature for 2 h. The reaction mixture was concentrated under reduced pressure, diluted with EtOAc, and washed once with saturated aqueous NaHCO_3_. The organic layer was separated, dried over Na_2_SO_4_, and concentrated *in vacuo*. The resulting residue was dissolved in MeOH (2.0 mL), and NH_3_ (7 M in MeOH, 0.1 mL) was added, followed by 10% Pd/C (4 mg, 0.004 mmol, 0.1 equiv) under stirring. The reaction vessel was purged with N_2_ and placed under a H_2_ atmosphere. After 4 h, the reaction mixture was filtered through Whatman filter paper and concentrated to form a yellow solid. The solid was purified by reverse-phase (C18 column) FCC (acetonitrile in H_2_O with 0.1% _v/v_ formic acid, 0–100%), and lyophilized to afford the desired carboxylic acid **PyC 12** in 75% yield over two steps (11 mg, 0.03 mmol) as a white crystalline solid containing 0.5 equiv.alent formic acid.

M.p.: 177–180 °C. ^1^H NMR (600 MHz, DMSO-*d*_6_, 373 K) δ 7.61–7.60 (m, 2H), 7.45–7.43 (m, 2H), 7.39–7.35 (m, 4H), 7.34–7.31­(m, 1H), 3.48–3.46 (m, 4H), 2.77–2.75 (m, 4H), 2.02 (s, 3H). ^13^C NMR (151 MHz, DMSO-*d*_6_, 373 K) δ 168.9, 161.2, 136.4, 133.6, 131.8, 131.6, 129.8, 129.6, 127.7, 127.3, 126.4, 125.4, 119.5, 115.7, 45.3, 45.3, 10.2. HRMS (TOF, ESI^+^) *m*/*z*: [M + H]^+^ calcd. for C_23_H_24_N_3_O_3_, 390.1812; found, 390.1825.

### 4.21 3-(4-Carboxyphenyl)-4-methyl-5-phenyl-1*H*-pyrrole-2-carboxylic Acid (**PyC 10**)

To a stirred solution of ethyl ester **12a** (100 mg, 0.19 mmol, 1.0 equiv) in dichloromethane (1.4 mL) was added trifluoroacetic acid (1.4 mL); the reaction mixture was stirred at room temperature for 2 h. The reaction mixture was concentrated under reduced pressure, diluted with ethyl acetate and washed once with saturated aqueous NaHCO_3_ solution. The organic layer was dried over Na_2_SO_4_ and concentrated under reduced pressure. With the residue, **General Procedure E** for ester hydrolysis was followed (stirring for 24 h at 25 °C) to afford the desired carboxylic acid **PyC 10** in 65% yield over two steps (40 mg, 0.124 mmol) as a white crystalline solid.

M.p.: >210 °C. ^1^H NMR (600 MHz, DMSO-*d*_6_) δ 11.75 (s, 1H), 7.94–7.92 (m, 2H), 7.60–7.58 (m, 2H), 7.46–7.42 (m, 4H), 7.35–7.32 (m, 1H), 1.98 (s, 3H); ^13^C NMR (151 MHz, DMSO-*d*_6_) δ 167.4, 161.8, 140.1, 132.9, 131.8, 130.6, 130.3, 128.8, 128.4, 128.3, 128.0, 127.1, 119.1, 116.3, 10.9. HRMS (TOF, ESI^+^) *m*/*z*: [M + H]^+^ calcd. for C_19_H_16_NO_4,_ 322.1074; found, 322.1066.

### 4.22 Synthesis of 3-(4-((2-Aminoethyl)­carbamoyl)­phenyl)-4-methyl-5-phenyl-1*H*-pyrrole-2-carboxylic Acid (**PyC 13**)

#### 4.22.1 *tert*-Butyl (2-(4-(4,4,5,5-Tetramethyl-1,3,2-dioxaborolan-2-yl)­benzamido)­ethyl)­carbamate (**42g**)

To a solution of 4-(4,4,5,5-tetramethyl-1,3,2-dioxaborolan-2-yl)­benzoic acid^79^ (**42f′**) (395 mg, 1.59 mmol, 1.0 equiv) in CH_2_Cl_2_ (3.0 mL) was added diisopropylethylamine (2 M in NMP, 3.18 mL, 6.37 mmol, 4.0 equiv), followed by HBTU (0.725 g,, 1.91 mmol, 1.2 equiv); the reaction mixture was stirred for 5 min at room temperature. The reaction mixture was cooled to 0 °C, and *N*-Boc-ethylenediamine (0.3 mL, 1.75 mmol, 1.1 equiv) was added portionwise, the mixture was then warmed to room temperature and stirred overnight. After completion of the reaction (as monitored by UPLC) saturated aqueous NaHCO_3_ and CH_2_Cl_2_ were added to the reaction mixture. The organic phase was separated and the aqueous phase was extracted with CH_2_Cl_2_ (×2). The combined organic extracts were dried over Na_2_SO_4_, filtered, then concentrated *in vacuo*. The resultant orange gum was purified by silica gel FCC (EtOAc in cyclohexane, 0–40%) to afford the desired compound **42g** in 61% yield (377 mg, 1.59 mmol) as a white crystalline solid.

M.p.: 143–145 °C. ^1^H NMR (600 MHz, CDCl_3_) δ 7.83 (d, *J* = 8.1 Hz, 2H), 7.79 (d, *J* = 8.1 Hz, 2H), 7.36 (br s, 1H), 5.16 (br s, 1H), 3.54–3.52 (m, 2H), 3.39–3.37 (m, 2H), 1.41 (s, 9H), 1.34 (s, 12H); ^13^C NMR (151 MHz, CDCl_3_) δ 167.9, 157.6, 136.5, 135.0, 126.3, 84.2, 80.1, 42.2, 40.1, 28.5, 25.0 (ArCB was not observed).^78^ HRMS (TOF, ESI^+^) *m*/*z*: [M + Na]^+^ calcd. for C_20_H_31_BN_2_O_5_Na, 413.2218; found, 413.2235.

#### 4.22.2 Ethyl 3-(4-((2-((*tert*-Butoxycarbonyl)­amino)­ethyl)­carbamoyl)­phenyl)-4-methyl-5-phenyl-1*H*-pyrrole-2-carboxylate (**13a**)

**General Procedure C** for Suzuki–Miyaura cross coupling was followed using iodo-pyrrole **41b** (215 mg, 0.61 mmol, 1.0 equiv) and pinacol-ester **42g** (354 mg, 0.91 mmol, 1.5 equiv). Purification by silica gel FCC (EtOAc in cyclohexane, 0–55%), followed by crystallization from chloroform gave the desired product **13a** in 75% yield (222 mg, 0.45 mmol) as a white crystalline solid.

M.p.: 166–168 °C. ^1^H NMR (600 MHz, DMSO*-d*_6_) δ 11.86 (br s, 1H), 8.45 (br t, *J* = 5.5 Hz, 1H), 7.85 (d, *J* = 8.2 Hz, 2H), 7.59–7.58 (m, 2H), 7.48–7.45 (m, 2H), 7.40–7.34 (m, 3H), 6.92 (br t, *J* = 5.7 Hz, 1H), 4.08 (q, *J* = 7.1 Hz, 2H), 3.33–3.30 (“br m”, 2H) (overlapped with H_2_O peak), 3.13 (“br q”, *J* = 6.2 Hz, 2H), 1.98 (s, 3H), 1.38 (s, 9H), 1.08 (t, *J* = 7.1 Hz, 3H); ^13^C NMR (151 MHz, DMSO*-d*_6_) δ 166.8, 160.8, 156.2, 138.5, 133.9, 133.0, 132.2, 131.3, 130.7, 128.8, 128.7, 127.8, 126.8, 118.7, 116.9, 78.1, 59.7, 28.7, 14.5, 11.3 (methylene −CH_2_– peaks were obscured by DMSO signal). HRMS (TOF, ESI^+^) *m*/*z*: [M + H]^+^ calcd. for C_28_H_34_N_3_O_5_, 492.2493; found, 492.2506.

#### 4.22.3 Benzyl 3-(4-((2-((*tert*-Butoxycarbonyl)­amino)­ethyl)­carbamoyl)­phenyl)-4-methyl-5-phenyl-1*H*-pyrrole-2-carboxylate (**13b**)

To a round-bottom flask containing ethyl ester **13a** (193 mg, 0.39 mmol, 1.0 equiv) under inert atmosphere, was added anhydrous benzyl alcohol (17.5 mL) and titanium­(IV) ethoxide (0.30 mL, 1.4 mmol, 3.6 equiv). The reaction mixture was heated for 20 h at 110 °C until the complete consumption of the starting material was observed (as monitored by UPLC). After cooling, the reaction mixture was diluted with EtOAc (30 mL), and washed once with water (50 mL). The organic layer was then dried over Na_2_SO_4_, and the volatiles were evaporated under reduced pressure to afford crude mixture with residual benzyl alcohol. The residue was purified by silica gel FCC (EtOAc in cyclohexane, 0–40%) to afford the desired compound **13b** in 56% yield (122 mg, 0.22 mmol) as a white crystalline solid.

M.p.: 192–194 °C (charred). ^1^H NMR (600 MHz, DMSO*-d*_6_) δ 11.97 (br s, 1H), 8.45 (br t, *J* = 5.5 Hz, 1H), 7.83 (d, *J* = 8.2 Hz, 2H), 7.60–7.58 (m, 2H), 7.48–7.45 (m, 2H), 7.39–7.34 (m, 3H), 7.26–7.23 (m, 3H), 7.09–7.07 (m, 2H), 6.92 (br t, *J* = 5.6 Hz, 1H), 5.13 (s, 2H), 3.35–3.32 (m, 2H, overlapped with H_2_O peak), 3.14 (‘br q’, *J* = 6.3 Hz, 2H), 1.97 (s, 3H), 1.38 (s, 9H); ^13^C NMR (151 MHz, DMSO*-d*_6_) δ 166.7, 160.6, 156.2, 138.7, 136.7, 134.3, 133.1, 132.2, 131.8, 130.6, 128.8, 128.7, 128.6, 128.0, 127.9, 127.7, 127.0, 118.4, 117.2, 78.2, 65.3, 28.7, 11.2 (methylene −CH_2_– peaks obscured by DMSO signal). HRMS (TOF, ESI^+^) *m*/*z*: [M + H]^+^ calcd. for C_33_H_36_N_3_O_5_, 554.2649; found, 554.2662.

#### 4.22.4 3-(4-((2-Aminoethyl)­carbamoyl)­phenyl)-4-methyl-5-phenyl-1*H*-pyrrole-2-carboxylic Acid (**PyC 13**)

To a stirred solution of compound **13b** (50 mg, 0.09 mmol, 1.0 equiv) in CH_2_Cl_2_ (0.7 mL) was added trifluoroacetic acid (0.7 mL); the reaction mixture was stirred at room temperature for 3 h. The reaction mixture was concentrated under reduced pressure, diluted with EtOAc and washed once with saturated aqueous NaHCO_3_ solution. The organic layer was separated, dried over Na_2_SO_4_, and concentrated *in vacuo*. The residue was dissolved in MeOH (5.1 mL), and NH_3_ (7 M in MeOH, 0.3 mL) was added, followed by addition of 10% Pd/C (10 mg, 0.01 mmol, 0.1 equiv) under stirring. The reaction vessel was purged with N_2_ and placed under a H_2_ atmosphere. After 4 h, the reaction mixture was filtered through Whatman filter paper and concentrated to form a yellow solid. The solid was purified by reverse-phase (C18 column) FCC (acetonitrile in H_2_O with 0.1% _v/v_ formic acid, 0–100%) and lyophilized to afford the desired carboxylic acid **PyC 13** in 52% yield over two steps (17 mg, 0.05 mmol) as white crystalline solid containing 0.5 equiv.alent formic acid.

M.p.: 155–157 °C. ^1^H NMR (600 MHz, DMSO*-d*_6_) δ 11.05 (br s, 1H), 7.93 (d, *J* = 8.3 Hz, 2H), 7.56 (d, *J* = 7.2 Hz, 2H), 7.47 (d, *J* = 8.3 Hz, 2H), 7.41 (t, *J* = 7.8 Hz, 2H), 7.27 (t, *J* = 7.4 Hz, 1H), 3.50 (“br q”, *J* = 5.5 Hz, 2H), 2.96 (“br q”, *J* = 5.2 Hz, 2H), 2.00 (s, 3H); ^13^C NMR (151 MHz, DMSO*-d*_6_) δ 166.5, 160.3, 139.4, 132.7, 131.3, 130.5, 128.3, 127.6, 126.3, 126.2, 115.3, 38.6, 38.6, 11.3. HRMS (TOF, ESI^+^) *m*/*z*: [M + H]^+^ calcd. for C_21_H_22_N_3_O_3_, 364.1656; found, 364.1661.

### 4.23 Synthesis of 3-(3,5-Dichlorophenyl)-4-ethyl-5-phenyl-1*H*-pyrrole-2-carboxylic Acid (**PyC 14**)

#### 4.23.1 Ethyl 4-Ethyl-5-phenyl-1*H*-pyrrole-2-carboxylate (**40c**)

A solution of (*E*)-2-benzylidenebutanal (**38b**) (1.4 mL, 9.36 mmol, 1.0 equiv) in EtOH (5.0 mL) was added dropwise to a cooled solution (−20 °C, acetonitrile/dry ice bath) of 21% w/w NaOEt in EtOH (5.2 mL, 14 mmol, 1.5 equiv). 25% w/w ethyl 2-azidoacetate (**39b**) in EtOH (22.0 mL, 37.4 mmol, 4.0 equiv) was then added dropwise over 1 h in 5 min intervals to the mixture. The reaction mixture was warmed to −10 °C and stirred at this temperature for 4 h, then at room temperature for 1 h. The reaction was quenched with water (50 mL) and diluted with EtOAc (125 mL). The organic layer was separated, and the aqueous layer was extracted with EtOAc (2 × 125 mL). The combined organic extracts were dried over Na_2_SO_4_, filtered, and concentrated *in vacuo*. The resulting residue was purified by silica gel FCC (EtOAc in cyclohexane, 0–20%) to afford the desired product **40c** in 27% yield (680 mg, 2.51 mmol) as a yellow-white amorphous solid.

^1^H NMR (400 MHz, CDCl_3_) δ 9.31 (br s, 1H), 7.49–7.40 (m, 4H), 7.35–7.31 (m, 1H), 6.90 (d, *J* = 2.7 Hz, 1H), 4.29 (q, *J* = 7.1 Hz, 2H), 2.65 (q, *J* = 7.5 Hz, 2H), 1.35 (t, *J* = 7.1 Hz, 3H), 1.24 (t, *J* = 7.5 Hz, 3H); ^13^C NMR (101 MHz, CDCl_3_) δ 161.5, 133.4, 132.5, 128.9, 127.6, 127.5, 125.4, 121.9, 116.1, 60.4, 19.7, 15.3, 14.6. HRMS (TOF, ESI^+^) *m*/*z*: [M + H]^+^ calcd. for C_15_H_18_NO_2_, 244.1332; found, 244.1325.

#### 4.23.2 Ethyl 4-Ethyl-3-iodo-5-phenyl-1*H*-pyrrole-2-carboxylate (**41c**)

Following **General Procedure A**, pyrrole **40c** (435 mg, 1.79 mmol, 1.0 equiv) was subjected to iodination. Purification by silica gel FCC (EtOAc in cyclohexane, 0–20%) afforded the desired product **41c** in 89% yield (587 mg, 1.59 mmol) as a beige crystalline solid.

M.p.: 105–107 °C. ^1^H NMR (400 MHz, CDCl_3_) δ 9.42 (br s, 1H), 7.46–7.43 (m, 4H), 7.41–7.35 (m, 1H), 4.33 (q, *J* = 7.1 Hz, 2H), 2.60 (q, *J* = 7.5 Hz, 2H), 1.39 (t, *J* = 7.1 Hz, 3H), 1.18 (t, *J* = 7.5 Hz, 3H); ^13^C NMR (101 MHz, CDCl_3_) δ 160.4, 133.6, 131.9, 129.0, 128.8, 128.3, 127.6, 122.4, 75.9, 60.8, 21.1, 15.3, 14.5. HRMS (TOF, ESI^+^) *m*/*z*: [M + H]^+^ calcd. for C_15_H_17_INO_2_, 370.0299; found, 370.0293.

#### 4.23.3 Ethyl 3-(3,5-Dichlorophenyl)-4-ethyl-5-phenyl-1*H*-pyrrole-2-carboxylate (**14a**)

**General Procedure C** for Suzuki–Miyaura cross coupling was followed using iodo-pyrrole **41c** (300 mg, 0.81 mmol, 1.0 equiv) and 3,5-dichlorophenylboronic acid (**42a**) (233 mg, 1.22 mmol, 1.5 equiv). Purification by silica gel FCC (EtOAc in cyclohexane, 0–6%) afforded the desired compound **14a** in 83% yield (289 mg, 0.68 mmol) as a white crystalline solid.

M.p.: 134–136 °C. ^1^H NMR (500 MHz, DMSO-*d*_6_) δ 11.98 (br s, 1H), 7.57–7.53 (m, 3H), 7.46 (“t”, *J* = 7.6 Hz, 2H), 7.39–7.35 (m, 3H), 4.07 (q, *J* = 7.1 Hz, 2H), 2.44 (q, *J* = 7.5 Hz, 2H), 1.06 (t, *J* = 7.1 Hz, 3H), 0.81 (t, *J* = 7.5 Hz, 3H); ^13^C NMR (126 MHz, DMSO-*d*_6_) δ 160.1, 139.3, 133.3, 133.1, 131.7, 128.9, 128.5, 128.3, 128.1, 127.6, 126.2, 123.1, 118.7, 59.4, 17.0, 15.5, 13.9. HRMS (TOF, ESI^+^) *m*/*z*: [M + H]^+^ calcd. for C_21_H_20_^35^Cl_2_NO_2_, 388.0866; found, 388.0859.

#### 4.23.4 3-(3,5-Dichlorophenyl)-4-ethyl-5-phenyl-1*H*-pyrrole-2-carboxylic Acid (**PyC 14**)

**General Procedure D** for ester hydrolysis was followed using the ethyl ester **14a** (189 mg, 0.49 mmol, 1.0 equiv) to afford the desired carboxylic acid **PyC 14** in 42% yield (74 mg, 0.21 mmol) as a white crystalline solid.

M.p.: 156–158 °C. ^1^H NMR (600 MHz, DMSO-*d*_6_) δ 12.19 (br s, 1H), 11.81 (br s, 1H), 7.55–7.53 (m, 3H), 7.46–7.44 (m, 2H), 7.37–7.34 (m, 3H), 2.42 (q, *J* = 7.5 Hz, 2H), 0.81 (t, *J* = 7.5 Hz, 3H); ^13^C NMR (151 MHz, DMSO-*d*_6_) δ 161.5, 139.6, 133.0, 132.6, 131.8, 128.8, 128.4, 128.1, 127.8, 127.4, 126.1, 123.0, 119.4, 17.0, 15.5. HRMS (TOF, ESI^+^) *m*/*z*: [M + H]^+^ calcd. for C_19_H_16_^35^Cl_2_NO_2_, 360.0553; found, 360.0544.

### 4.24 Synthesis of 4-Ethyl-3-(3-fluoro-4-((methylsulfonyl)­methyl)­phenyl)-5-phenyl-1*H*-pyrrole-2-carboxylic Acid (**PyC 15**)

#### 4.24.1 Ethyl 4-Ethyl-3-(3-fluoro-4-((methylsulfonyl)­methyl)­phenyl)-5-phenyl-1*H*-pyrrole-2-carboxylate (**15a**)

**General Procedure C** for Suzuki–Miyaura cross coupling was followed using iodo-pyrrole **41c** (300 mg, 0.81 mmol, 1.0 equiv) and pinacol-ester **42b** (383 mg, 1.22 mmol, 1.5 equiv). Purification by silica gel FCC (EtOAc in cyclohexane, 0–25%) afforded the desired compound **15a** in 76% yield (256 mg, 0.62 mmol) as a beige crystalline solid.

M.p.: 162–164 °C. ^1^H NMR (500 MHz, DMSO-*d*_6_) δ 11.89 (br s, 1H), 7.56–7.54 (m, 2H), 7.49–7.45 (m, 3H), 7.39–7.35 (m, 1H), 7.22–7.17 (m, 2H), 4.58 (s, 2H), 4.05 (q, *J* = 7.1 Hz, 2H), 3.02 (s, 3H), 2.45 (q, *J* = 7.4 Hz, 2H), 1.04 (t, *J* = 7.1 Hz, 3H), 0.81 (t, *J* = 7.4 Hz, 3H); ^13^C NMR (126 MHz, DMSO-*d*_6_) δ 161.2 (d, *J*_C–F_ = 247.0 Hz), 160.2, 138.6 (d, *J*_C–F_ = 8.7 Hz), 133.2, 132.1 (d, *J*_C–F_ = 3.9 Hz), 131.9, 129.4, 128.4, 128.3, 127.6, 126.3 (d, *J*_C–F_ = 3.0 Hz), 123.1, 118.5, 117.2 (d, *J*_C–F_ = 21.7 Hz), 114.2 (d, *J*_C–F_ = 15.4 Hz), 59.3, 53.0, 39.9, 17.1, 15.5, 13.9; ^19^F NMR (377 MHz, CDCl_3_) δ −112.64 (dd, *J*_F–H_ = 10.5, 8.2 Hz). HRMS (TOF, ESI^+^) *m*/*z*: [M + H]^+^ calcd. for C_23_H_25_FNO_4_S, 430.1483; found, 430.1480.

#### 4.24.2 4-Ethyl-3-(3-fluoro-4-((methylsulfonyl)­methyl)­phenyl)-5-phenyl-1*H*-pyrrole-2-carboxylic Acid (**PyC 15**)

**General Procedure E** for ester hydrolysis was followed using ethyl ester **15a** (286 mg, 0.67 mmol, 1.0 equiv) to afford the desired carboxylic acid **PyC 15** in 41% yield (110 mg, 0.27 mmol) as a white crystalline solid.

M.p.: 185–187 °C. ^1^H NMR (600 MHz, DMSO-*d*_6_) δ 12.08 (br s, 1H), 11.73 (br s, 1H), 7.55–7.54 (m, 2H), 7.47–7.44 (m, 3H), 7.37–7.34 (m, 1H), 7.21–7.17 (m, 2H), 4.57 (s, 2H), 3.03 (s, 3H), 2.44 (q, *J* = 7.4 Hz, 2H), 0.80 (t, *J* = 7.4 Hz, 3H); ^13^C NMR (151 MHz, DMSO-*d*_6_) δ 161.6, 160.1 (d, *J*_C–F_ = 246.9 Hz), 138.7 (d, *J*_C–F_ = 8.2 Hz), 132.6, 132.1, 132.0, 129.0, 128.4, 128.1, 127.3, 126.4, 123.1, 119.1, 117.2 (d, *J*_C–F_ = 21.5 Hz), 113.9 (d, *J*_C–F_ = 15.2 Hz), 53.0, 40.1, 17.1, 15.5; ^19^F NMR (565 MHz, DMSO-*d*_6_) δ −117.35 to −117.43 (m). HRMS (TOF, ESI^+^) *m*/*z*: [M + H]^+^ calcd. for C_21_H_21_FNO_4_S, 402.1170, found, 402.1176.

### 4.25 Synthesis of 4-Ethyl-3-(4-fluorophenyl)-5-phenyl-1*H*-pyrrole-2-carboxylic Acid (**PyC 16**)

#### 4.25.1 Ethyl 4-Ethyl-3-(4-fluorophenyl)-5-phenyl-1*H*-pyrrole-2-carboxylate (**16a**)

**General Procedure C** for Suzuki–Miyaura cross coupling was followed using iodo-pyrrole **41c** (300 mg, 0.81 mmol, 1.0 equiv) and (4-fluorophenyl)­boronic acid (**42h**) (171 mg, 1.22 mmol, 1.5 equiv). Purification by silica gel FCC (EtOAc in cyclohexane, 0–7%) afforded the desired compound **16a** in 81% yield (212 mg, 0.66 mmol) as a white crystalline solid.

M.p.: 118–120 °C. ^1^H NMR (400 MHz, DMSO-*d*_6_) δ 11.79 (s, 1H), 7.57–7.54 (m, 2H), 7.47–7.43 (m, 2H), 7.38–7.29 (m, 3H), 7.21–7.16 (m, 2H), 4.03 (q, *J* = 7.1 Hz, 2H), 2.42 (q, *J* = 7.4 Hz, 2H), 1.03 (t, *J* = 7.1 Hz, 3H), 0.80 (t, *J* = 7.4 Hz, 3H); ^13^C NMR (101 MHz, DMSO-*d*_6_) δ 162.4 (d, *J*_C–F_ = 243.4 Hz), 160.4, 133.0, 132.0, 131.9 (d, *J*_C–F_ = 8.1 Hz), 131.8 (d, *J*_C–F_ = 3.0 Hz), 130.2, 128.4, 128.2, 127.4, 123.3, 118.5, 114.2 (d, *J*_C–F_ = 21.2 Hz), 59.1, 17.1, 15.5, 13.9; ^19^F NMR (376 MHz, DMSO-*d*_6_) δ −116.48 (tt, *J*_F–H_ = 9.1, 5.7 Hz). HRMS (TOF, ESI^+^) *m*/*z*: [M + H]^+^ calcd. for C_21_H_21_FNO_2_, 338.1551; found, 338.1552.

#### 4.25.2 4-Ethyl-3-(4-fluorophenyl)-5-phenyl-1*H*-pyrrole-2-carboxylic Acid (**PyC 16**)

**General Procedure E** for ester hydrolysis was followed using ethyl ester **16a** (195 mg, 0.58 mmol, 1.0 equiv) to afford the desired carboxylic acid **PyC 16** in 37% yield (67 mg, 0.22 mmol) as a white crystalline solid.

M.p.: 135–139 °C. ^1^H NMR (600 MHz, DMSO-*d*_6_) δ 11.98 (br s, 1H), 11.62 (s, 1H), 7.56–7.54 (m, 2H), 7.45–7.43 (m, 2H), 7.36–7.30 (m, 3H), 7.19–7.16 (m, 2H), 2.41 (q, *J* = 7.4 Hz, 2H), 0.79 (t, *J* = 7.5 Hz, 3H); ^13^C NMR (151 MHz, DMSO-*d*_6_) δ 161.8, 161.1 (d, *J*_C–F_ = 241.6 Hz), 132.4, 132.1, 132.0 (d, *J*_C–F_ = 3.0 Hz), 131.9 (d, *J*_C–F_ = 7.6 Hz), 129.8, 128.4, 128.0, 127.2, 123.2, 119.1, 114.2 (d, *J*_C–F_ = 19.6 Hz), 17.1, 15.5; ^19^F NMR (565 MHz, DMSO-*d*_6_) δ −116.67 to −116.72 (m). HRMS (TOF, ESI^+^) *m*/*z*: [M + Na]^+^ calcd. for C_19_H_16_FNO_2_Na, 332.1057; found, 332.1048.

### 4.26 Synthesis of 4-Cyano-3-(3-fluoro-4-((methylsulfonyl)­methyl)­phenyl)-5-phenyl-1*H*-pyrrole-2-carboxylic Acid (**PyC 17**)

#### 4.26.1 Ethyl 2-Azido-5-phenylpenta-2,4-dienoate (**39c**)

*trans*-Cinnamaldehyde (**38c**) (0.45 mL, 3.6 mmol, 1.0 equiv) was added dropwise to a solution of NaOEt (367 mg, 5.40 mmol, 1.5 equiv) in dry EtOH (7.0 mL) at −20 °C, followed by dropwise addition over several minutes of a solution of ethyl 2-azidoacetate (**39b**) (1.7 mL, 14.4 mmol, 4.0 equiv) dissolved in anhyd EtOH (4.0 mL). The reaction mixture was stirred at −20 °C for 1 h, warmed to −10 °C for 3 h, and then stirred at room temperature for 1 h. The reaction mixture was then concentrated *in vacuo*, diluted with EtOAc (50 mL) and treated with ice-cold water (50 mL). The organic layer was separated and the aqueous layer was extracted with EtOAc (2 × 50 mL). The organic fractions were combined, dried over Na_2_SO_4_, filtered, then concentrated *in vacuo* with the water bath maintained at ∼30 °C in a fume cupboard. The resulting residue was purified by silica gel FCC (EtOAc in cyclohexane, 0–20%) to afford the desired compound **39c** in 35% yield (240 mg, 0.97 mmol) as a yellow oil which solidified on freezing.

^1^H NMR (500 MHz, CDCl_3_) δ 7.50–7.48 (m, 2H), 7.36 (dd, *J* = 8.3, 6.5 Hz, 2H), 7.32–7.29 (m, 1H), 7.18 (dd, *J* = 15.7, 11.3 Hz, 1H), 6.81 (d, *J* = 15.7 Hz, 1H), 6.76 (dd, *J* = 11.3, 0.9 Hz, 1H), 4.34 (q, *J* = 7.1 Hz, 2H), 1.39 (t, *J* = 7.2 Hz, 3H); ^13^C NMR (126 MHz, CDCl_3_) δ 163.1, 138.9, 136.4, 128.9, 128.8, 127.3, 126.8, 125.7, 122.2, 62.0, 14.2. HRMS (TOF, ESI^+^) *m*/*z*: [M + Na]^+^ calcd. for C_13_H_13_N_3_O_2_Na, 266.0900; found, 266.0892.

#### 4.26.2 Ethyl 5-Phenyl-1*H*-pyrrole-2-carboxylate (**40d**)

To a round-bottom flask containing compound **39c** (655 mg, 2.86 mmol, 1.0 equiv) in anhyd CH_2_Cl_2_ (3.0 mL) under an inert atmosphere, was added ZnI_2_ (45 mg, 0.14 mmol, 0.05 equiv); the reaction mixture was stirred at room temperature for 15 h. The reaction mixture was concentrated *in vacuo* and the crude mixture was purified using silica gel FCC (EtOAc in cyclohexane, 0–20%) to afford the desired product **40d** in 53% yield (325 mg, 1.51 mmol) as a yellow-white crystalline solid.

M.p.: 114–116 °C. ^1^H NMR (600 MHz, CDCl_3_) δ 9.46 (br s, 1H), 7.59–7.57 (m, 2H), 7.41 (dd, *J* = 8.2, 7.4 Hz, 2H), 7.32–7.29 (m, 1H), 6.97 (dd, *J* = 3.9, 2.4 Hz, 1H), 6.54 (dd, *J* = 3.9, 2.4 Hz, 1H), 4.35 (q, *J* = 7.1 Hz, 2H), 1.38 (t, *J* = 7.1 Hz, 3H); ^13^C NMR (151 MHz, CDCl_3_) δ 161.4, 136.9, 131.5, 129.1, 127.9, 124.9, 123.5, 116.8, 108.1, 60.6, 14.6. HRMS (TOF, ESI^+^) *m*/*z*: [M + H]^+^ calcd. for C_13_H_14_NO_2_, 216.1019; found, 216.1017.

#### 4.26.3 Ethyl 4-Bromo-5-phenyl-1*H*-pyrrole-2-carboxylate (**43′**)

To a solution of pyrrole **40d** (150 mg, 0.70 mmol, 1.0 equiv) in CH_2_Cl_2_ (20 mL) at −40 °C, was added *N*-bromosuccinimide (99 mg, 0.56 mmol, 0.8 equiv) portionwise under stirring; the resulting mixture was stirred for 3 h at −40 °C, then 1 h at room temperature. The reaction mixture was washed with brine (2 × 20 mL), organic layer was dried over Na_2_SO_4_, filtered, and concentrated *in vacuo*. The residue was purified by silica gel FCC (CH_2_Cl_2_ in cyclohexane, 0–20%) to give the monobrominated product **43′** in 38% yield (78 mg, 0.27 mmol) as a beige amorphous solid.

^1^H NMR (600 MHz, CDCl_3_) δ 9.79 (br s, 1H), 7.72–7.70 (m, 2H), 7.46–7.43 (m, 2H), 7.40–7.35 (m, 1H), 6.99 (d, *J* = 2.7 Hz, 1H), 4.26 (q, *J* = 7.1 Hz, 2H), 1.32 (t, *J* = 7.1 Hz, 3H); ^13^C NMR (151 MHz, CDCl_3_) δ 160.9, 134.1, 130.5, 128. 8, 128.5, 127.6, 122.6, 119.1, 96.3, 61.0, 14.5. HRMS (TOF, ESI^+^) *m*/*z*: [M + H]^+^ calcd. for C_13_H_13_^79^BrNO_2_, 294.0124; found, 294.0119.

#### 4.26.4 Ethyl 4-Cyano-5-phenyl-1*H*-pyrrole-2-carboxylate (**43**)

A 2 mL microwave vial containing bromo-pyrrole **43′** (100 mg, 0.34 mmol, 1.0 equiv), K_4_Fe­(CN)_6_·3H_2_O (72 mg, 0.17 mmol, 0.5 equiv), *t*BuXPhos (2 mg, 0.005 mmol, 0.014 equiv), and *t*BuXPhos-Pd-G3 (4 mg, 0.005 mmol, 0.014 equiv) was subjected to vacuum then filled with N_2_ three times. Anhydrous 1,4-dioxane (0.5 mL, N_2_-purged) and 0.05 M aqueous KOAc (0.5 mL, N_2_-purged with sonication) were then added to the vial. The reaction mixture was heated at 100 °C for 1.5 h, cooled to room temperature, diluted with EtOAc, and washed with brine. The aqueous layer turned Prussian blue in color. The organic phase was separated and the aqueous phase was twice extracted with EtOAc. The combined organic extracts were washed with brine, dried over Na_2_SO_4_, filtered, and concentrated in *vacuo*. The crude residue was purified by silica gel FCC (EtOAc in cyclohexane, 0–20%) to afford the desired product **43** in 72% yield (59 mg, 0.25 mmol) as a white amorphous solid.

^1^H NMR (600 MHz, CDCl_3_) δ 9.91 (br s, 1H), 7.79–7.78 (m, 2H), 7.52–7.49 (m, 2H), 7.47–7.45 (m, 1H), 7.19 (d, *J* = 2.6 Hz, 1H), 4.33 (q, *J* = 7.2 Hz, 2H), 1.36 (t, *J* = 7.1 Hz, 3H); ^13^C NMR (151 MHz, CDCl_3_) δ 160.5, 142.2, 130.1, 129.5, 128.8, 126.7, 123.8, 119.7, 116.2, 92.5, 61.6, 14.4. HRMS (TOF, ESI^–^) *m*/*z*: [M – H]^−^ calcd. for C_14_H_11_N_2_O_2_, 239.0826; found, 239.0827.

#### 4.26.5 Ethyl 4-Cyano-3-iodo-5-phenyl-1*H*-pyrrole-2-carboxylate (**45**)

Following **General Procedure A**, cyano-pyrrole **43** (38 mg, 0.16 mmol, 1.0 equiv) was subjected to iodination. After workup, the crude residue afforded the desired compound **45** in quantitative yield and was used directly in the subsequent Suzuki–Miyaura coupling reaction without further purification.

^1^H NMR (700 MHz, DMSO-*d*_6_) δ 7.79–7.76 (m, 2H), 7.56–7.53 (m, 2H), 7.52–7.49 (m, 1H), 4.33 (q, *J* = 7.1 Hz, 2H), 1.34 (t, *J* = 7.1 Hz, 3H); ^13^C NMR (176 MHz, DMSO-*d*_6_) δ 158.9, 143.9, 130.0, 128.9, 128.1, 127.7, 125.0, 116.7, 100.4, 77.5, 60.9, 14.2. HRMS (TOF, ESI^+^) *m*/*z*: [M + H]^+^ calcd. for C_14_H_12_IN_2_O_2_, 366.9938; found, 366.9933.

#### 4.26.6 Ethyl 4-Cyano-3-(3-fluoro-4-((methylsulfonyl)­methyl)­phenyl)-5-phenyl-1*H*-pyrrole-2-carboxylate (**17a**)

**General Procedure B** for Suzuki–Miyaura cross coupling was followed using 4-cyano-3-iodo-pyrrole (**45**) (50 mg, 0.14 mmol, 1.0 equiv) and pinacol ester **42b** (64 mg, 0.20 mmol, 1.5 equiv). Purification by silica gel FCC (EtOAc in cyclohexane, 0–40%) afforded the desired product **17a** in 74% yield (43 mg, 0.10 mmol), as a white amorphous solid.

^1^H NMR (600 MHz, DMSO-*d*_6_) δ 13.14 (s, 1H), 7.86–7.84 (m, 2H), 7.58–7.51 (m, 4H), 7.46 (dd, *J* = 10.7, 1.7 Hz, 1H), 7.39 (dd, *J* = 7.9, 1.7 Hz, 1H), 4.62 (s, 2H), 4.18 (q, *J* = 7.1 Hz, 2H), 3.05 (s, 3H), 1.13 (t, *J* = 7.1 Hz, 3H); ^13^C NMR (151 MHz, DMSO-*d*_6_) δ 161.8 (d, *J*_C–F_ = 247.6 Hz), 159.4, 141.9, 134.4 (d, *J*_C–F_ = 8.9 Hz), 132.5 (d, *J*_C–F_ = 3.7 Hz), 132.1 (d, *J*_C–F_ = 2.0 Hz), 129.8, 128.9, 128.4, 127.7, 126.0 (d, *J*_C–F_ = 3.1 Hz), 120.4, 117.2 (d, *J*_C–F_ = 23.0 Hz), 116.0, 115.8 (d, *J*_C–F_ = 15.3 Hz), 92.7, 60.5, 53.0, 40.1, 13.7; ^19^F NMR (565 MHz, DMSO-*d*_6_) δ −116.60 to −116.64 (m). HRMS (TOF, ESI^+^) *m*/*z*: [M + H]^+^ calcd. for C_22_H_20_FN_2_O_4_S, 427.1122; found, 427.1129.

#### 4.26.7 4-Cyano-3-(3-fluoro-4-((methylsulfonyl)­methyl)­phenyl)-5-phenyl-1*H*-pyrrole-2-carboxylic Acid (**PyC 17**)

To a solution of ethyl ester **17a** (18 mg, 0.04 mmol, 1.0 equiv) in EtOH (0.5 mL) was added 4 M aqueous KOH (1 mL) at room temperature; the resultant mixture was stirred at 40 °C for 12 h. Upon completion of the reaction, the mixture was cooled to room temperature and acidified to pH 2 with 4 N HCl and the aqueous layer was extracted with EtOAc (×2). The combined organic extracts were dried over Na_2_SO_4_, filtered, and concentrated *in vacuo*. The resulting residue was purified by reverse phase (C18 column) FCC (acetonitrile in H_2_O with 0.1% v/v formic acid, 0–100%) followed by lyophilization to afford the desired product **PyC 17** in 42% yield (7 mg, 0.0176 mmol) as a yellow-white crystalline solid.

M.p.: 178–180 °C; ^1^H NMR (600 MHz, MeOH-*d*_4_) δ 7.85–7.83 (m, 2H), 7.59–7.48 (m, 4H), 7.44–7.40 (m, 2H), 4.55 (s, 2H), 2.98 (s, 3H); ^13^C NMR (151 MHz, MeOH-*d*_4_) δ 162.8, 162.0 (d, *J*_C–F_ = 247.6 Hz), 143.4, 136.8 (d, *J*_C–F_ = 8.8 Hz), 133.8, 133.5 (d, *J*_C–F_ = 3.4 Hz), 130.8, 130.3, 130.1, 128.5, 127.5 (d, *J*_C–F_ = 3.2 Hz), 122.6, 118.6 (d, *J*_C–F_ = 23.3 Hz), 117.1, 117.1 (d, *J*_C–F_ = 15.2 Hz), 94.0, 54.6 (d, *J*_C–F_ = 2.2 Hz), 40.1 (d, *J*_C–F_ = 1.6 Hz); ^19^F NMR (565 MHz, MeOH-*d*_4_) δ −118.74 to −118.77 (m). HRMS (TOF, ESI^+^) *m*/*z*: [M + H]^+^ calcd. for C_20_H_16_FN_2_O_4_S, 399.0809; found, 399.0801.

### 4.27 Synthesis of 4-Fluoro-3-(3-fluoro-4-((methylsulfonyl)­methyl)­phenyl)-5-phenyl-1*H*-pyrrole-2-carboxylic Acid (**PyC 18**)

#### 4.27.1 Ethyl 4-Fluoro-5-phenyl-1*H*-pyrrole-2-carboxylate (**44**)

To a 5 mL microwave vial containing pyrrole **40d** (70 mg, 0.33 mmol, 1.0 equiv) in acetonitrile (2.0 mL) was added Selectfluor (160 mg, 0.46 mmol, 1.4 equiv). The reaction mixture was heated under microwave irradiation at 100 °C for 5 min. After completion, the reaction mixture was cooled to room temperature, quenched with water, and extracted with CH_2_Cl_2_. The organic layer was separated, and the aqueous layer was extracted with CH_2_Cl_2_ (×2). The combined organic fractions were dried over Na_2_SO_4_, filtered, and concentrated in *vacuo*. Purification of the crude residue by silica gel FCC (EtOAc in cyclohexane, 0–8%) afforded a mixture of product **44** with the starting material **40d**. Further purification by reverse-phase (C18 column) FCC (acetonitrile in H_2_O with 0.1% v/v formic acid, 0–100%), followed by lyophilization, afforded the desired product **44** in 32% yield (24 mg, 0.103 mmol) as a white amorphous solid.

^1^H NMR (400 MHz, CDCl_3_) δ 9.27 (br s, 1H), 7.65–7.63 (m, 2H), 7.43 (td, *J* = 7.5, 1.2 Hz, 2H), 7.33–7.29 (m, 1H), 6.69 (d, *J* = 2.9 Hz, 1H), 4.33 (qd, *J* = 7.2, 0.8 Hz, 2H), 1.36 (td, *J* = 7.1, 0.8 Hz, 3H); ^13^C NMR (101 MHz, CDCl_3_) δ 161.3, 149.0 (d, *J*_C–F_ = 246.7 Hz), 129.2 (d, *J*_C–F_ = 4.2 Hz), 129.1, 127.8, 125.2 (d, *J*_C–F_ = 4.7 Hz), 120.6 (d, *J*_C–F_ = 19.4 Hz), 118.2 (d, *J*_C–F_ = 7.0 Hz), 103.5 (d, *J*_C–F_ = 16.0 Hz), 61.0, 14.5; ^19^F NMR (376 MHz, CDCl_3_) δ −158.82 (d, *J* = 2.1 Hz). HRMS (TOF, ESI^+^) *m*/*z*: [M + H]^+^ calcd. for C_13_H_13_FNO_2_, 234.0925; found, 234.0925.

#### 4.27.2 Ethyl 4-Fluoro-3-iodo-5-phenyl-1*H*-pyrrole-2-carboxylate (**46**)

Following **General Procedure A**, fluoro-pyrrole **44** (12 mg, 0.05 mmol, 1.0 equiv) was subjected to iodination. Purification of the crude residue by silica gel FCC (EtOAc in cyclohexane, 0–8%) afforded the desired compound **46** in 85% yield (38 mg, 0.11 mmol) as a white amorphous solid.

^1^H NMR (600 MHz, CDCl_3_) δ 9.36 (br s, 1H), 7.61 (d, *J* = 7.6 Hz, 2H), 7.46–7.44 (m, 2H), 7.34 (t, *J* = 7.4 Hz, 1H), 4.39 (q, *J* = 7.1 Hz, 2H), 1.42 (t, *J* = 7.1 Hz, 3H); ^13^C NMR (151 MHz, CDCl_3_) δ 160.3, 150.6 (d, *J*_C–F_ = 245.6 Hz), 129.3, 128.4, 128.3 (d, *J*_C–F_ = 4.4 Hz), 125.3 (d, *J*_C–F_ = 4.3 Hz), 120.4 (d, *J*_C–F_ = 19.9 Hz), 118.9 (d, *J*_C–F_ = 3.6 Hz), 61.4, 58.9 (d, *J*_C–F_ = 21.0 Hz), 14.5; ^19^F NMR (565 MHz, CDCl_3_) δ −152.16 (s,). HRMS (TOF, ESI^+^) *m*/*z*: [M + H]^+^ calcd. for C_13_H_12_FINO_2_, 359.9891; found, 359.9886.

#### 4.27.3 Ethyl 4-Fluoro-3-(3-fluoro-4-((methylsulfonyl)­methyl)­phenyl)-5-phenyl-1*H*-pyrrole-2-carboxylate (**18a**)

**General Procedure B** for Suzuki–Miyaura cross coupling was followed using 4-fluoro-3-iodo-pyrrole (**46**) (16 mg, 0.05 mmol, 1.0 equiv) and pinacol ester **42b** (17 mg, 0.05 mmol, 1.2 equiv). Purification by silica gel FCC (EtOAc in cyclohexane, 0–40%) afforded the desired product **18a** in 96% yield (18 mg, 0.04 mmol) as a white amorphous solid.

^1^H NMR (700 MHz, DMSO-*d*_6_) δ 12.27 (br s, 1H), 7.83–7.81 (m, 2H), 7.52– 7.47 (m, 3H), 7.43–7.41 (m, 1H), 7.37–7.34 (m, 2H), 4.60 (s, 2H), 4.19 (q, *J* = 7.1 Hz, 2H), 3.04 (s, 3H), 1.15 (t, *J* = 7.1 Hz, 3H); ^13^C NMR (176 MHz, DMSO-*d*_6_) δ 160.3 (d, *J*_C–F_ = 246.4 Hz), 160.1 (d, *J*_C–F_ = 2.6 Hz), 145.9 (d, *J*_C–F_ = 246.5 Hz), 133.3 (d, *J*_C–F_ = 7.4 Hz), 132.4 (d, *J*_C–F_ = 3.5 Hz), 128.9, 128.5 (d, *J*_C–F_ = 4.1 Hz), 127.8, 126.4, 126.1 (d, *J*_C–F_ = 4.3 Hz), 119.9 (d, *J*_C–F_ = 17.6 Hz), 117.4 (d, *J*_C–F_ = 22.7 Hz), 116.2 (d, *J*_C–F_ = 10.0 Hz), 115.0 (d, *J*_C–F_ = 7.1 Hz), 115.0 (d, *J*_C–F_ = 3.9 Hz), 60.2, 53.0, 40.1, 13.9. ^19^F NMR (376 MHz, DMSO-*d*_6_) δ −116.96 (dd, J = 10.9, 8.0 Hz), −159.35 to −167.01 (m, 1F). HRMS (TOF, ESI^+^) *m*/*z*: [M + H]^+^ calcd. for C_21_H_20_F_2_NO_4_S, 420.1076; found, 420.1069.

#### 4.27.4 4-Fluoro-3-(3-fluoro-4-((methylsulfonyl)­methyl)­phenyl)-5-phenyl-1*H*-pyrrole-2-carboxylic Acid (**PyC 18**)

**General Procedure E** for ester hydrolysis was followed using ethyl ester **18a** (18 mg, 0.04 mmol, 1.0 equiv) to afford the desired carboxylic acid **PyC 18** in 48% yield (8 mg, 0.0204 mmol) as a white crystalline solid.

M.p.: 171–173 °C. ^1^H NMR (600 MHz, MeOH-*d*_4_) δ 7.76–7.75 (m, 2H), 7.52 (t, *J* = 7.8 Hz, 1H), 7.47–7.40 (m, 4H), 7.35–7.32 (m, 1H), 4.53 (s, 2H), 2.97 (s, 3H); ^13^C NMR (151 MHz, MeOH-*d*_4_) δ 163.5, 162.1 (d, *J*_C–F_ = 246.5 Hz), 147.8 (d, *J*_C–F_ = 246.3 Hz), 135.9 (d, *J*_C–F_ = 8.8 Hz), 133.3 (d, *J*_C–F_ = 3.4 Hz), 130.4 (d, *J*_C–F_ = 4.1 Hz), 129.9, 128.8, 127.8 (d, *J*_C–F_ = 2.4 Hz), 127.0 (d, *J*_C–F_ = 4.4 Hz), 121.3 (d, *J*_C–F_ = 18.4 Hz), 118.6 (d, *J*_C–F_ = 23.1 Hz), 118.2 (d, *J*_C–F_ = 11.1 Hz), 116.6, 116.1 (d, *J*_C–F_ = 15.2 Hz), 54.6 (d, *J*_C–F_ = 2.4 Hz), 40.0; ^19^F NMR (565 MHz, MeOH-*d*_4_) δ −119.28 to −119.31 (m), −166.02 (s). HRMS (TOF, ESI^+^) *m*/*z*: [M + H]^+^ calcd. for C_19_H_16_F_2_NO_4_S, 392.0763; found, 392.0764.

### 4.28 Synthesis of 3-Cyano-5-phenyl-1*H*-pyrrole-2-carboxylic Acid (**PyC 5**)

#### 4.28.1 Ethyl 3-Cyano-5-phenyl-1*H*-pyrrole-2-carboxylate (**5a**)

To a round-bottom flask containing pyrrole **40d** (168 mg, 0.78 mmol, 1.0 equiv) in anhyd DMF (0.64 mL) and acetonitrile (8 mL) at 0 °C, was added chlorosulfonyl isocyanate (80 μL, 0.92 mmol, 1.2 equiv); the reaction mixture was stirred at room temperature for 15 h. The reaction mixture was quenched with saturated aqueous Na_2_CO_3_ and extracted with EtOAc. The organic layer was separated and the aqueous layer was extracted with EtOAc (×2). The combined organic extracts were dried over Na_2_SO_4_, filtered, and concentrated *in vacuo*. The crude residue was purified by silica gel FCC (EtOAc in cyclohexane, 0–15%) to afford the desired compound **5a** in 37% yield (69 mg, 0.29 mmol) as a white crystalline solid.

M.p.: 150–152 °C. ^1^H NMR (500 MHz, CDCl_3_) δ 9.67 (br s, 1H), 7.78–7.76 (m, 2H), 7.53–7.45 (m, 3H), 7.19 (d, *J* = 2.6 Hz, 1H), 4.35 (q, *J* = 7.1 Hz, 2H), 1.38 (t, *J* = 7.2 Hz, 3H); ^13^C NMR (126 MHz, CDCl_3_) δ 160.4, 142.0, 130.1, 129.6, 128.7, 126.5, 123.8, 119.6, 116.2, 92.5, 61.5, 14.5. HRMS (TOF, ESI^+^) *m*/*z*: [M + H]^+^ calcd. for C_14_H_13_N_2_O_2_, 241.0972; found, 241.0968.

#### 4.28.2 3-Cyano-5-phenyl-1*H*-pyrrole-2-carboxylic Acid (**PyC 5**)

**General Procedure E** for ester hydrolysis was followed using ethyl ester **5a** (56 mg, 0.23 mmol, 1.0 equiv) to afford the desired carboxylic acid **PyC 5** in 55% yield (27 mg, 0.13 mmol) as a white crystalline solid.

M.p.: >208 °C. ^1^H NMR (600 MHz, MeOH-*d*_4_) δ 7.80–7.79 (m, 2H), 7.52–7.49 (m, 2H), 7.47–7.44 (m, 1H), 7.15 (s, 1H); ^13^C NMR (151 MHz, MeOH-*d*_4_) δ 163.8, 143.4, 130.7, 130.5, 130.1, 129.0, 128.1, 119.8, 117.8, 92.0. HRMS (TOF, ESI^+^) *m*/*z*: [M + Na]^+^ calcd. for C_12_H_8_N_2_O_2_Na, 235.0478; found, 235.0475.

### 4.29 Biochemical Assays

Assays were performed using a previously reported fluorogenic method using the synthetic substrate FC5.^1^ Inhibitor concentrations ranged from 50 pM to 100 μM, with metallo-β-lactamases (MBLs) preincubated with inhibitor for 10 min prior to substrate addition. The final enzyme concentrations were: NDM-1 (20 pM), VIM-1 (500 pM), VIM-2 (100 pM), and IMP-1 (20 pM). Final concentration of FC5 was 5 μM. Reactions were carried out in 50 mM HEPES buffer (pH 7.2) containing 1 μM ZnSO_4_, 1 μg mL^–1^ BSA, and 0.01% (v/v) Triton X-100. All assays were run with four replicates.

### 4.30 Crystallography

Recombinant VIM-1 protein (>90% pure by SDS-PAGE and MS analysis) was produced and purified as reported.^15^ VIM-1 was cocrystallized with each inhibitor by the sitting drop vapor diffusion method. 18.3 mg/mL VIM-1 was incubated with 7 mM inhibitor for 20 min before mixing 1.5 μL of protein-inhibitor solution with 3 μL of well solution. The well solution contained 0.1 M tris, pH 8.5, and between 2.5 and 2.7 M ammonium phosphate. After growth over 1–3 days, the crystals were cryoprotected with 20% (v/v) glycerol and flash cooled in liquid nitrogen. Data were collected at 100 K at the Diamond Light Source IO3 beamline. Data were integrated using xia2 with DIALS and scaled using Aimless.^80,81^ All structures were solved in the *P*12_1_1 space group with one molecule in the asymmetric unit. Crystal structures were solved by molecular replacement using Phaser using a previously reported structure (PDB 5NI5).^82^ Model refinement was done using Refmac in CCP4i2 (v. 9.0.003), PHENIX Refine (v. 1.20.1–4487), and Coot (v. 0.9.8.1). Inhibitor restraints were calculated using eLBOW in PHENIX.^83–87^ Ligand-protein interactions were analyzed using Maestro and Protein–Ligand Interaction Profiler (PLIP).^88^

### 4.31 Microbiological Assays

#### 4.31.1 Plasmid Construction

Gene cassettes encoding for the carbapenemase genes *bla*_*KPC‑2*_, *bla*_*VIM‑2*_, *bla*_*NDM‑1*_, *bla*_*VIM‑1*_ and their native promoter were PCR-amplified from the clinical isolates K3K, B2H, K5N, and E4A^66^ respectively using the primer pairs listed in Table S3. The clinical isolates were obtained from the Timothy Walsh laboratory collection and the BARNARDS (Burden of Antibiotic Resistance in Neonates from Developing Societies) group of the Ineos Oxford Institute for Antimicrobial Research. To construct pK18-KPC-2 and pK18-VIM-2, the PCR-amplified fragment was cloned into the multiple-cloning site of the vector pK18, a pMB1-derived high-copy number vector with the gene *aph­(3′)-II* conferring resistance to kanamycin,^89^ by restriction digest and the ligation mix was transformed into *E. coli* NEB 10-β chemically competent cells. The plasmids pK18-NDM-1 and pK18-VIM-1 were constructed using the NEBuilder HiFi DNA Assembly technique and transformed into *E. coli* NEB 5-α chemically competent cells. The transformants were selected at 37 °C on LB (Miller) agar (Sigma-Aldrich) plates supplemented with kanamycin and ampicillin at 50 μg/mL. The cloned DNA regions obtained by PCR were verified by sequencing.

#### 4.31.2 Isogenic Panel Construction

The recombinant plasmids pK18-KPC-2, pK18-VIM-2, pK18-NDM-1, and pK18-VIM-1 were introduced into *E. coli* MG1655 cells with an inactivated chromosomal AmpC β-lactamase by electroporation. The transformants were selected on LB (Miller) agar (Sigma-Aldrich) plates supplemented with kanamycin at 50 μg/mL. As a negative control, the empty vector pK18 was also transformed into MG1655 Δ*ampC*. An in-frame deletion of *ampC* gene was constructed in a *E. coli* MG1655 background strain using a temperature-inducible λ-Red recombineering system on a pSIM5-Tet plasmid^90^ that is derived from the original pSIM5.^91^ The *kan-sacB-T0* cassette derived from the *cat-sacB-T0* ^92^ was amplified by PCR from the DA27219 strain with primers including 40 bp flanking homology regions outside *ampC* using Phusion Plus Green PCR Master Mix (Thermo Fisher Scientific Inc.). Overnight culture of DA24100 (*E. coli* MG1655/pSIM5-Tet) was diluted 100-fold in 50 mL LB with 10 mg/L tetracycline (250 mL E-flask) and grown at 30 °C with shaking (180 rpm) until early exponential phase (OD_600_ ∼ 0.3). The λ-Red system was induced by 15 min incubation in a 42 °C shaking water bath and the culture was cooled on ice for 10 min. Cells were made electrocompetent by washing 4 times (4 min at 4000*g*, 4 °C) in ice-cold sterile 10% _v/v_ aqueous glycerol and were then resuspended in 500–800 μL (final volume). The electrocompetent cells (50 μL) were mixed with ∼500 ng of the purified *kan-sacB-T0* cassette, transferred to an electroporation cuvette (2 mm gap, Bio-Rad) and electroporated in a MicroPulser (Bio-Rad) at 2.5 kV. The cells were recovered in 1 mL LB overnight at 30 °C with shaking. Transformants were selected on LB agar with kanamycin (50 mg/L) at 30 °C and checked for sucrose sensitivity on 5% sucrose plates (10 g/L tryptone, 5 g/L yeast extract, 15 g/L agar, 1 mM NaOH, 50 g/L sucrose) and the carriage of the pSIM5 plasmid on LB agar with tetracycline (10 mg/L). Successful transformants were used for another λ-Red recombineering step with a linear ssDNA fragment containing 40 bp homologous regions directly upstream and 40 bp downstream of *ampC* to delete the *kan-sacB* cassette. Transformants were selected on 5% sucrose agar plates and PCR-verified for the correct deletion. The pSIM5 plasmid was cured from the final construct by growing on LB agar plate without any antibiotics at 42 °C overnight. Oligonucleotides used for the construction can be found in Table S3.

#### 4.31.3 MIC Determination by Broth Microdilution

Minimum inhibitory concentration (MIC) values were determined by broth microdilution according to European Committee on Antimicrobial Susceptibility Testing (EUCAST) guidelines. The MIC was defined as the lowest concentration that inhibits visible growth of the tested bacterial isolate as observed by the naked eye. Cation-adjusted Mueller Hinton II broth (BD BBL) was used and Meropenem trihydrate was purchased from United States Biological Life Sciences. A Meropenem (MEM) aqueous stock solution was prepared and the metallo-β-lactamase (MBL) inhibitors were dissolved in dimethyl sulfoxide (DMSO). The inhibitors were tested against the *E. coli* isogenic strains expressing NDM-1, VIM-1, VIM-2, and KPC-2 at a fixed concentration of 4 mg/L in combination with Meropenem (32–0.03 mg/L). The activity of the MBL inhibitors was also assessed against 14 clinical isolates expressing different β-lactamases. The inhibitors were tested against the clinical panel at 8 mg/L and 16 mg/L in combination with Meropenem at a concentration varying from 128 to 0.125 mg/L. The clinical isolates were obtained from the Timothy Walsh laboratory collection and the BARNARDS collection, at the Ineos Oxford Institute for antimicrobial research.^66^ The description of the whole genome sequencing (WGS) analysis of the bacterial strains is reported (BioProject ID PRJNA984017).^66^ The WGS of the isolates was submitted to ResFinder 4.7.2 for classification of the carbapenemase genes present. A list of all the strains used in MIC assays is given in Table S4. The *E. coli* strain ATCC 25922 was used as a control. MIC data were obtained from at least two independent experiments.

## Supplementary Material























## Notes

Synthetic chemistry schemes, electron density map, ligand interaction diagram and active site view of crystal structures (Figures S1–S8); crystallographic data collection parameters, refinement and Ramachandran statistics, MIC of Meropenem in combination with inhibitors (8 mg/L), details of oligonucleotides used for experiments, details of the strains used in MIC assays, cytotoxicity and ADME data, IC_50_ against Human MBL-fold Nuclease SNM1C (Tables S1–S6); experiments and methods and their associated references, including: assay protocols for cytotoxicity screening, plasma stability assay, metabolic stability test, and chemical stability test, IC_50_ against Human MBL-fold Nuclease SNM1C, and ChromLogD measurement; NMR spectra of novel intermediates and final compounds; UPLC and HRMS traces of final compounds (PDF)
Molecular formula strings for each compound with corresponding IC_50_ assay data (CSV)
MIC data against isogenic strains (CSV)
MIC data (inhibitor concentration: 16 mg/L) against clinical strains (CSV)
MIC data (inhibitor concentration: 8 mg/L) against clinical strains (CSV)
9RFG (PDB)
9RFH (PDB)
9RFI (PDB)
9RFJ (PDB)
9RFK (PDB)
9RFM (PDB)

## ⊥

Enzymology and Applied Biocatalysis Research Center, Faculty of Chemistry and Chemical Engineering, Babes-Bolyai University, Str. Arany Janos, nr. 11, Cluj-Napoca RO-400028, Romania http://chem.ubbcluj.ro/BIO/CENTRU/

## Abbreviations Used

Anhyd: anhydrous
BnOH: benzyl alcohol
calcd.: calculated
EtOAc: ethyl acetate
EtOH: ethanol
IMP: imipenemase
InC: indole-2-carboxylate
iPrOH: isopropanol
KOAc: potassium acetate
MBL: metallo-β-lactamase
MBLI: metallo-β-lactamase inhibitor
MeCN: acetonitrile
MEM: meropenem
MeOH: methanol
MIC: minimum inhibitory concentration
m.p.: melting point
μg: microgram
μM: micromolar
NaOEt: sodium ethoxide
NDM: New Delhi metallo-β-lactamase
pM: picomolar
PyC: pyrrole-2-carboxylic acid
SBL: serine-β-lactamase
TLC: thin layer chromatography
VIM: Verona integron-encoded metallo-β-lactamase
