# Supplementary material for: Development of Water-Trapping Pyrrole-2-carboxylic Acids as Broad-Spectrum Metallo-β-lactamase Inhibitors
Source: J Med Chem. 2026 May 14;69(10):11961–84. doi: 10.1021/acs.jmedchem.5c03534 (PMC13224168; doi:10.1021/acs.jmedchem.5c03534)
Supplement: Supplementary file 1 [file jm5c03534_si_001.pdf]

Supporting Information

Development of Water-Trapping Pyrrole-2-carboxylic Acids as Broad-spectrum Metallo-β-lactamase Inhibitors

Monisha Singha[1], Liam A. Wilson[1‡], Elisabete C. C. M. Moura[2‡], Maria M. Trush[2], Karina Calvopina[1], Gurleen Kaur[1], Greta Zaborskytė[2], Toms Kalniņš[3], Tharindi Panduwawala[1], Matthew J. Bowen,[1] Matthew J. Beech,[1] Jürgen Brem[1], Peter J. McHugh,[4] Edgars Suna,[3] Timothy R. Walsh[2], Christopher J. Schofield[1*], and Alistair J. M. Farley[1*].

[1]Chemistry Research Laboratory, Department of Chemistry and the Ineos Oxford Institute for Antimicrobial Research, University of Oxford, 12 Mansfield Road, Oxford OX1 3TA, United Kingdom.

[2]Sir William Dunn School of Pathology, Department of Biology and the Ineos Oxford Institute for Antimicrobial Research, University of Oxford, S Parks Rd, Oxford OX1 3RE, United Kingdom.

[3]Latvian Institute of Organic Synthesis, Riga LV-1006, Latvia.

[4]Department of Oncology, MRC-Weatherall Institute of Molecular Medicine, University of Oxford, Oxford OX3 9DS, UK

‡ These authors contributed equally.

email: christopher.schofield@chem.ox.ac.uk; alistair.farley@chem.ox.ac.uk

Table of Contents

I. **Supplementary Schemes – Synthetic Chemistry** ……………………………………... S3

II. **Supplementary Figures and Data – Crystallography** ……………...……..………... S6

III. **Supplementary Data – MIC and Details Bacterial of Strains** ………….…..……… S11

IV. **Supplementary Experiments and Methods** ....…………..….. S14

a. Cytotoxicity and ADME Data ………….…………………….....……........… S14

b. IC_{50} Against Human MBL-fold Nuclease SNM1C ………………...………. S17

c. ChromLogD Measurement ……………………..……………………..…… S17

V. **References** ……………………………………………………………….……… S18

VI. **NMR Spectra of Novel Intermediates and Final Compounds** ………..…...…..... S20

VII. **UPLC & HRMS Traces of Final Compounds** ………………...…...……………..…... S82

I. Supplementary Schemes – Synthetic Chemistry

Figure S1. Synthesis of **PyC 1**.

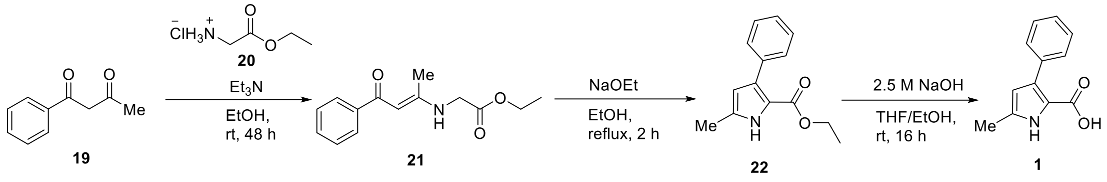

Figure S2. Synthesis of **PyC 2** and **3**.

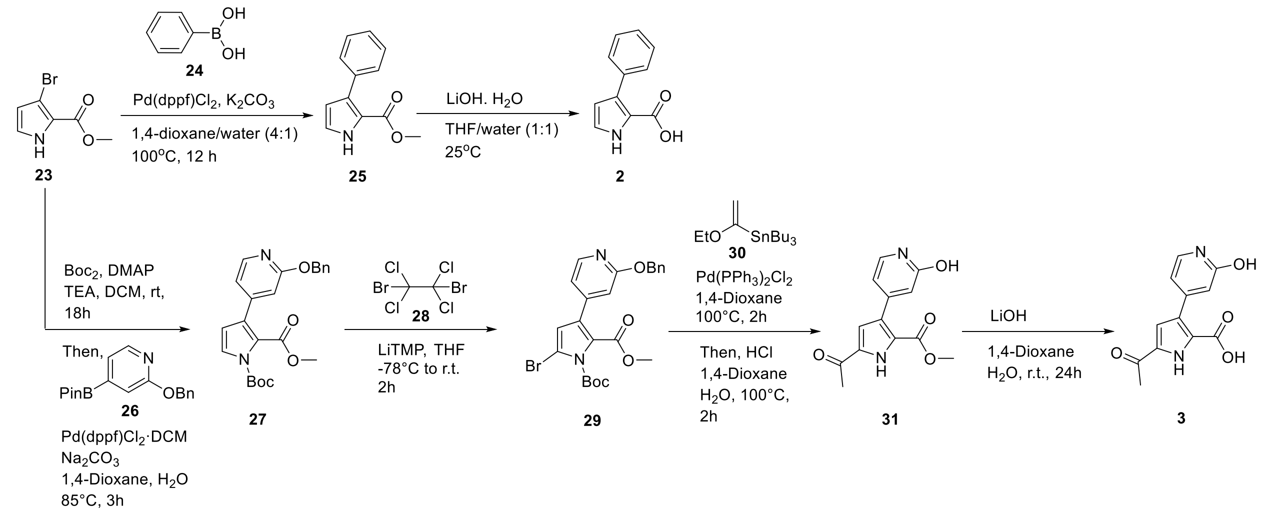

Figure S3. Synthesis of **PyC 4a** and **4b**.

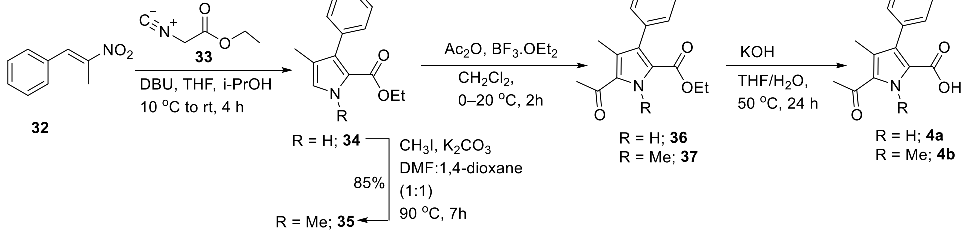

Figure S4: Syntheses of **PyC 6**, **7**, **8**, **9**, **10**, **11**, **12**, **13**, **14**, **15**, and **16**.

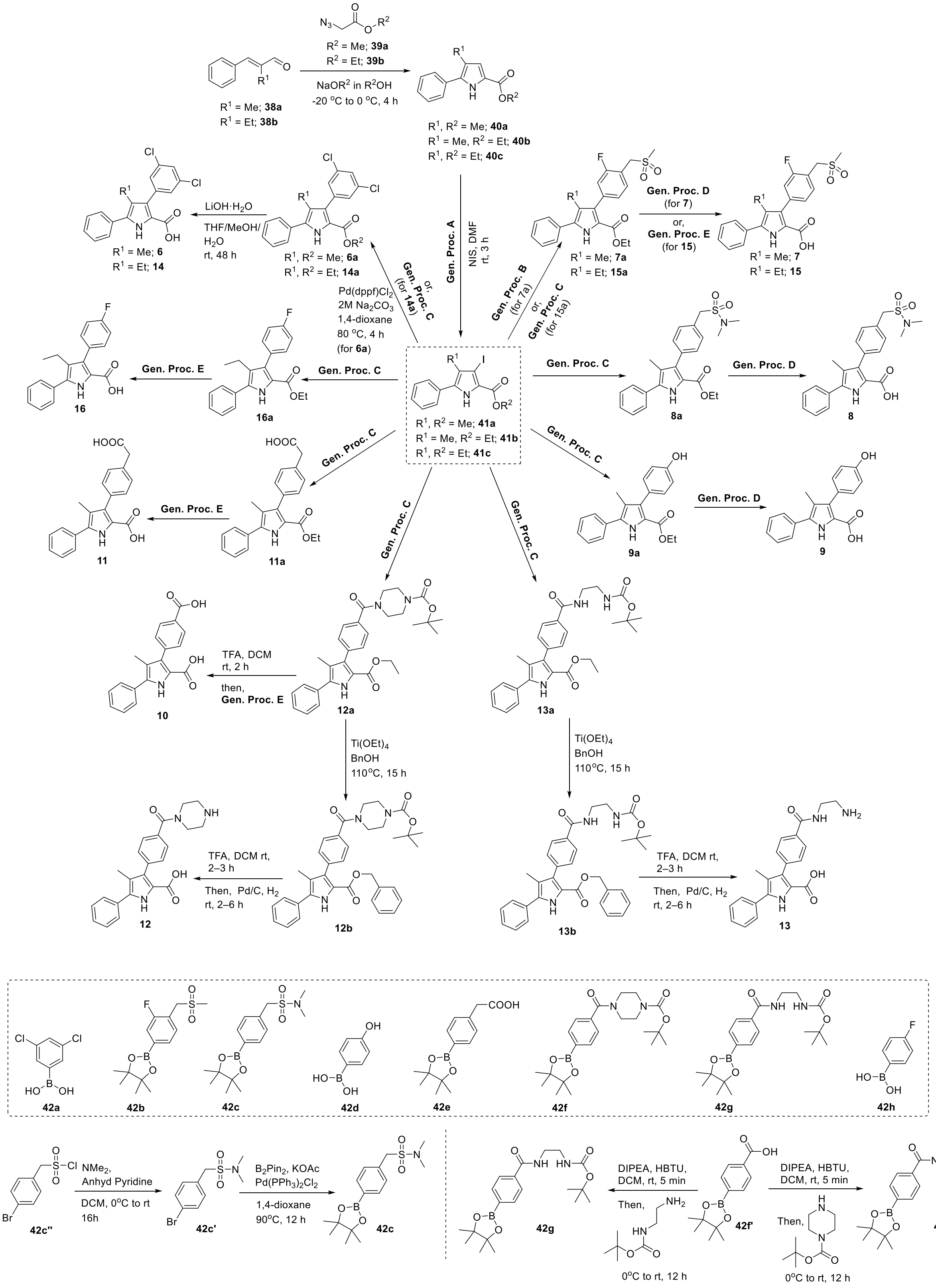

Figure S5: Syntheses of **PyC 5**, **17**, and **18**.

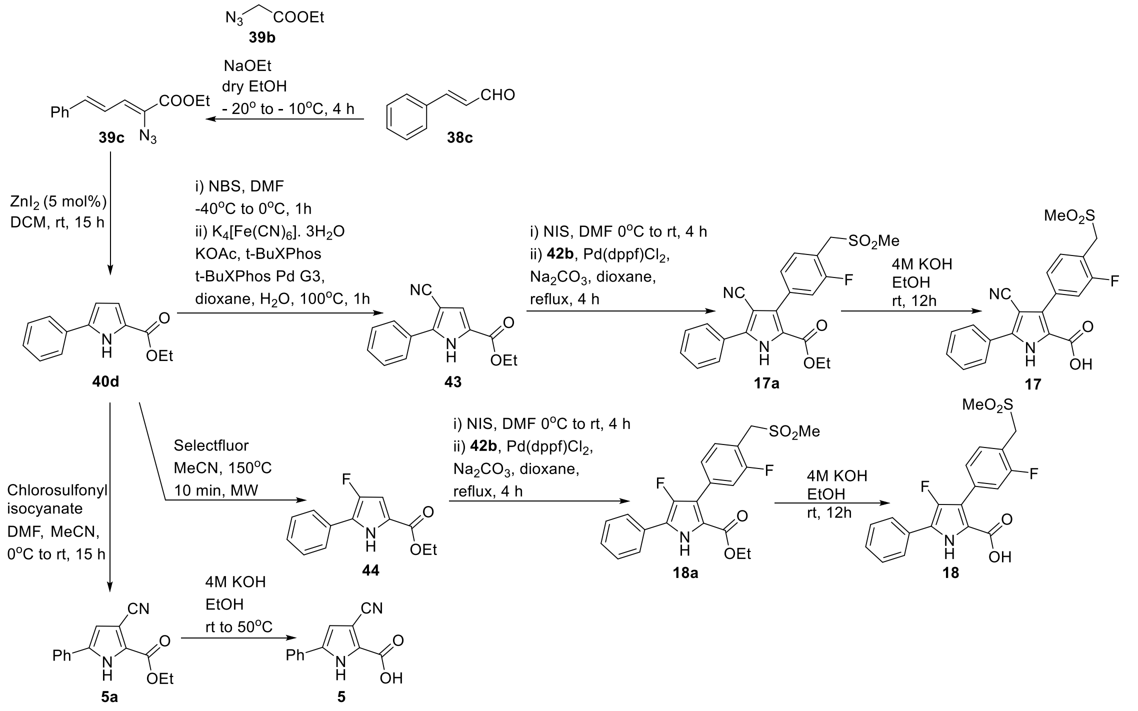

II. Supplementary Figures and Data – Crystallography

Figure S6. Electron density, displayed as polder maps, derived from crystal structures of VIM-1 in complex with: **PyC 6** (a) (PDB 9RFK), **PyC 7** (b) (PDB 9RFM), **PyC 14** (c) (PDB 9RFG), **PyC 15** (d) (PDB 9RFJ), **PyC 8** (e) (PDB 9RFI), and **PyC 11** (f) (PDB 9RFH). All maps are contoured to 3.6 σ.

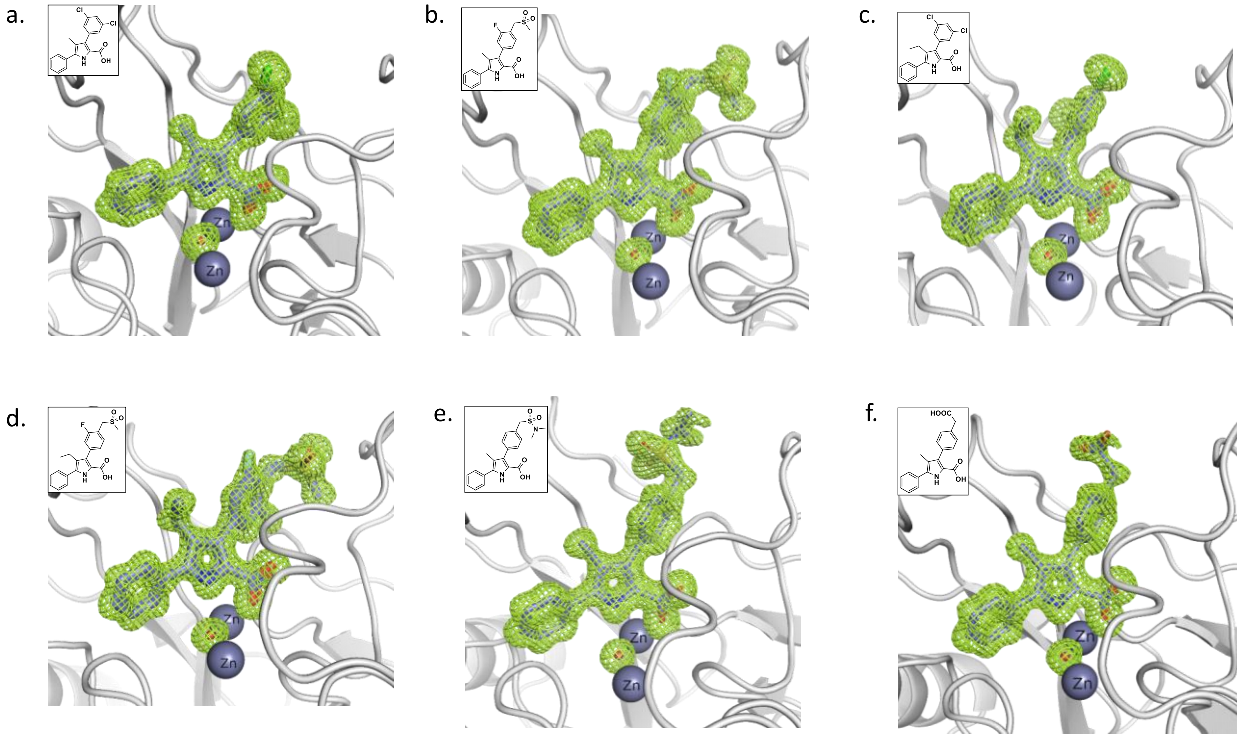

Table S1. Crystallographic data collection parameters, refinement and Ramachandran statistics for all solved VIM-1 complex crystal structures. High resolution shell statistics are in parentheses.

Parameter	Ligand Complexed with VIM-1					
	**PyC 14**	**PyC 6**	**PyC 7**	**PyC 15**	**PyC 8**	**PyC 11**
PDB code	9RFG	9RFK	9RFM	9RFJ	9RFI	9RFH
**Data collection**						
Resolution range (Å)	40.08 - 1.15 (1.191 - 1.15)	40.23 - 1.15 (1.191 - 1.15)	40.06 - 1.05 (1.088 - 1.05)	33.93 - 1.15 (1.191 - 1.15)	34.47 - 1.1 (1.139 - 1.10)	26.64 - 1.15 (1.191 - 1.15)
No. of observations [I>σ(I)]	516361 (48415)	518041 (47265)	670345 (66071)	519437 (48776)	585125 (55358)	517550 (48647)
No. of unique reflections [I>σ(I)]	74387 (7426)	74337 (7411)	96415 (9546)	74784 (7480)	84705 (8392)	74754 (7486)
Completeness (%)	99.99 (100.00)	99.22 (98.93)	98.51 (97.94)	99.99 (99.99)	99.69 (99.02)	99.99 (100.00)
Mean <I/σ(I)>	13.22 (1.57)	15.54 (1.34)	13.81 (1.21)	13.75 (1.76)	11.93 (1.77)	14.51 (1.32)
Rmerge	0.08762 (0.7877)	0.07239 (0.8618)	0.06495 (0.6537)	0.08517 (0.7276)	0.09229 (0.7407)	0.07921 (0.9076)
Rpim	0.03553 (0.3323)	0.02922 (0.3656)	0.026 (0.266)	0.03458 (0.3082)	0.03737 (0.3096)	0.03224 (0.3842)
CC(1/2)	0.999 (0.768)	0.998 (0.714)	0.999 (0.812)	0.999 (0.755)	0.999 (0.754)	0.999 (0.671)
Multiplicity	6.9 (6.5)	7.0 (6.4)	7.0 (6.9)	6.9 (6.5)	6.9 (6.6)	6.9 (6.5)
Space group	P 1 21 1	P 1 21 1	P 1 21 1	P 1 21 1	P 1 21 1	P 1 21 1
Unit cell length (Å)	a=39.3383 b=67.6518 c=40.147	a=39.4158 b= 67.7504 c=40.3003	a=39.3982 b=67.7713 c=40.1177	a=39.4219 b=67.8588 c=40.1493	a=39.3668 b=67.6364 c=40.1264	a=39.4002 b=67.7757 c=40.2104
Unit cell angle (°)	α = λ = 90, β = 93.3459	α = λ = 90, β = 93.4939	α = λ = 90, β = 93.1672	α = λ = 90, β = 93.2797	α = λ = 90, β = 93.2188	α = λ = 90, β = 93.4196

**Refinement statistics**						
Rwork	0.1367 (0.2402)	0.1400 (0.2600)	0.1127 (0.2027)	0.1279 (0.2339)	0.1293 (0.2342)	0.1319 (0.2538)
Rfree	0.1658 (0.2592)	0.1704 (0.2812)	0.1327 (0.2018)	0.1561 (0.2633)	0.1539 (0.2552)	0.1611 (0.2708)
RMSD bond lengths (Å)	0.009	0.007	0.011	0.009	0.006	0.006
RMSD bond angles (°)	1.12	0.97	1.30	1.11	1.06	0.96
Clash score	3.26	2.93	3.66	2.73	4.82	3.47
**Ramachandran plot statistics**						
Favored regions	98.27	98.26	98.26	98.26	98.26	98.26
Outlier regions	0.43	0.00	0.43	0.00	0.43	0.43
Rotamer outliners	1.4	1.35	0.91	0.93	0.47	0.92

Figure S7. Ligand interaction diagrams for **PyC 14** (a), **6** (b), **7** (c), **15** (d), **8** (e), and **11** (f) VIM-1 complex structures showing direct interactions formed by the bound inhibitors with protein residues or solvent. Hydrogen bonding interactions: purple arrows with the arrow pointing in the direction of the H-bond acceptor; pi-pi stacking interactions: green lines; metal coordination: black lines, pi-cation interactions: red lines; halogen bonds: light brown lines. Residues are represented as picks, with the point representing the side-chain direction. Hydrophobic residues: green; polar residues: blue; negatively charged residues: red; glycine: coloured beige: and metals: grey. The border around the ligand represents a protein contact; green border: hydrophobic contact; red and blue: contacts with negatively charged and positively charged residues respectively; beige and grey represent contacts with glycine and metals, respectively. Atoms with a grey halo are solvent exposed. The figure was made using Schrodinger Maestro V14.4.136.[1]

Figure S7 (continued)

a.

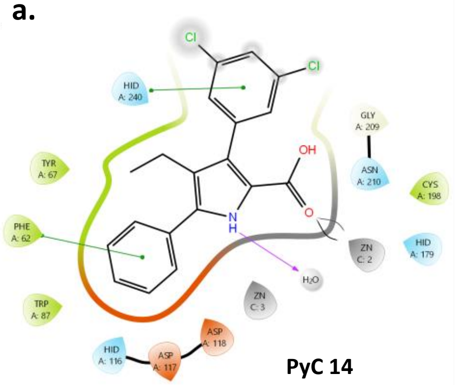

PyC 14

b.

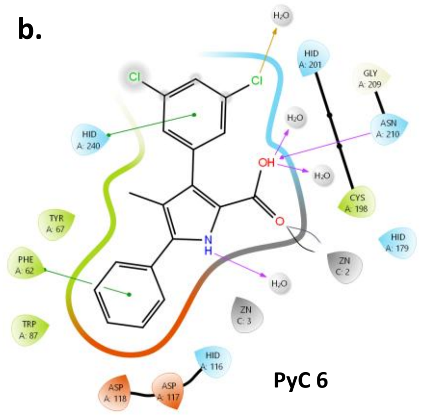

PyC 6

c.

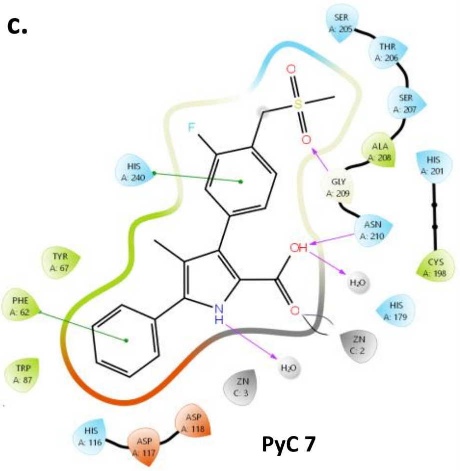

PyC 7

d.

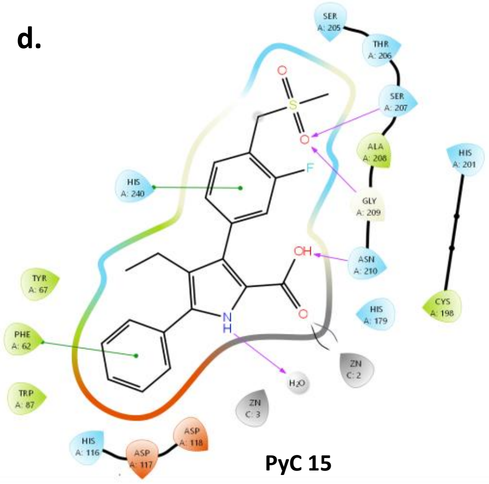

PyC 15

e.

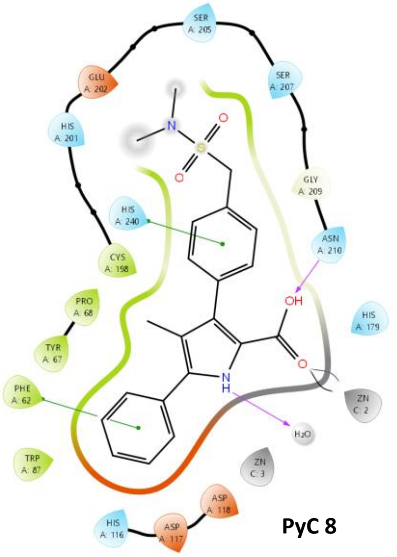

PyC 8

f.

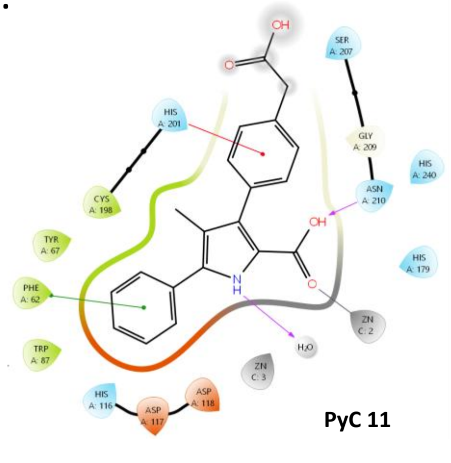

PyC 11

Figure S8. Active site view of the VIM-1 B1 MBL in complex with **PyC 7** (a) and **PyC 11** (b) showing the π-stacking and π-cation interactions between the C3 phenyl ring and His201 and His240. The interactions shown in the **PyC 7** bound VIM-1 complex structure are representative of the interactions seen with **PyC 6**, **14**, **15**, and **8** bound to VIM-1. Note that only **PyC 11** is positioned to interact with His201. In both views, π-interactions and hydrogen-bonding to the catalytic water are shown with black dotted lines. Only the side-chain atoms of the residues are shown for clarity. His201 is modelled as two conformational isomers.

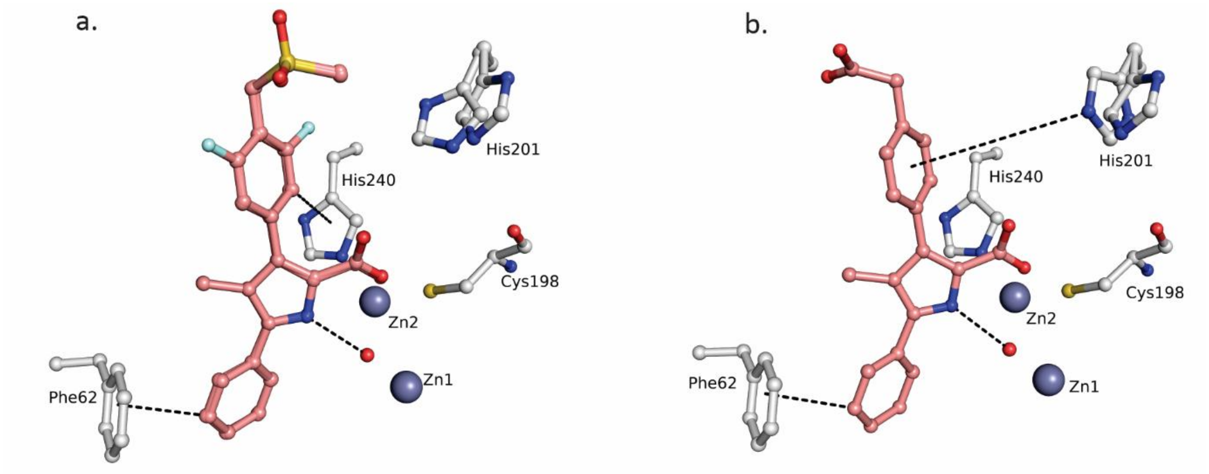

III. Supplementary Data – MIC and Details of Bacterial Strains

Table S2: MIC (mg/L) values of meropenem in combination with selected pyrrole 2-carboxylate inhibitors (8 mg/L) against MBL-producing clinical isolates.

Strain	Species	Carbapenemase profile	MEM	Inhibitor 8 mg/L + MEM 128 - 0.125 mg/L									
				6	7	9	10	12	13	14	15	18	Inc58
**ATCC 25922**	*E. coli*		0.016	≤ 0.125	≤ 0.125	≤ 0.125	≤ 0.125	≤ 0.125	≤ 0.125	≤ 0.125	≤ 0.125	≤ 0.125	≤ 0.125
**K1N**	*K. pneumoniae*	NDM-1, OXA-181	128	8	8	4-8	4	2-4	4-8	8	8	4	0.5
**K2N**	*K. pneumoniae*	NDM-1	64	8	8	4	4	4	8	8	8	4-8	1
**K8N**	*K. pneumoniae*	NDM-7	128	8	8	8	4	4	8	8	8	4	0.25
**E8N**	*E. coli*	NDM-5	64	4-8	8	8	8	4-8	8	8	8	4	0.5
**E10N**	*E. coli*	NDM-5	64	4	4	2	2	1	2	4	4	2	0.125-0.25
**E11N**	*E. coli*	NDM-7	> 128	32	64	32	32	32	64	32	32-64	32	2-4
**C1A**	*E. coli*	NDM-4	64	2	2	1-2	2	1	2	2	2	1	≤ 0.125
**C5A**	*C. sedlakii*	NDM-1	64	2	2	2	2	1-2	2-4	4	2	1	≤ 0.125
**E5A**	*E. hormaechei*	VIM-2	16	2	4-8	4	4	8	4	2	8	8	2
**S4A**	*S. marcescens*	NDM-1	128	8	4	4	4	2-4	8	8	4	4	0.5
**B3H**	*P. aeruginosa*	VIM-2	128	32	128	64	64	128	64	32	64-128	128	32
**P43**	*P. aeruginosa*	IMP-1	≥ 128	≥ 128	≥ 128	≥ 128	64	64	64-128	≥ 128	128	≥ 128	128
**A10K**	*A. baumannii*	NDM-1, OXA-98	> 128	32	32	32	64	32	64	32	32	16	4
**IEC429**	*A. baumannii*	IMP-1	128	64	64	64	64	64	64	64	128	64	64

Table S3: Oligonucleotides used in our work.

Name		Sequence (5’-3’)[a]	Used to make
KPC-2-KpnIFw	Forward	gagaggtaccGAGACAATAACCCTGATAAATGC	pK18-KPC-2
KPC-2-BamHIRv	Reverse	ttgaggatccGTGACAGTGGTTGGTAAT	pK18-KPC-2
VIM-2-KpnIFw	Forward	gagaggtaccGTTCGCCAGCCAGGACAGAAATGCCTCGACTTC	pK18-VIM-2
VIM-2-BamHIRv	Reverse	ttgaggatccATCTAACGCCGAAGTTCAGCCGCCAG	pK18-VIM-2
HiFi_pK18-NDM-1Fw	Forward	tacgaattcgagctcggtaccTCAGCTTGTTGATTATCATATG	pK18-NDM-1
HiFi_pK18-NDM-1Rv	Reverse	tctagaggatccccgggtacATAAAACGCCTCTGTCAC	pK18-NDM-1
HiFi_pK18-VIM-1Fw	Forward	tacgaattcgagctcggtaccAGAAATGCCTCGACTTCG	pK18-VIM-1
HiFi_pK18-VIM-1Rv	Reverse	tctagaggatccccgggtacAAGGTCATGCTCAGTCATG	pK18-VIM-1
sacB_ampC-F	Forward	CCCGCCTATGGCGGGCCGTTTTGTATGGAAACCAGACCCTTGTAGGCTGGAGCTGCTTC	MG1655 Δ*ampC*
sacB_ampC-R	Reverse	AAGCGGAGAAAAGGTCCGAAAATTCGGACCCGATGGAATTCATATGAATATCCTCCTTAGTTCC	MG1655 Δ*ampC*
del_ampC		CCCGCCTATGGCGGGCCGTTTTGTATGGAAACCAGACCCTAATTCCATCGGGTCCGAATTTTCGGACCTTTTCTCCGCTT	MG1655 Δ*ampC*
ampC_int_F	Forward	CGTCACACAGCAAACGTTGT	MG1655 Δ*ampC*
ampC_int_R	Reverse	CAGCATCACGATACCCAGCT	MG1655 Δ*ampC*

[a] Genomic sequence in uppercase; restriction sites in underlined lowercase.

Table S4: Details of the strains used in MIC assays.

Strain	Species	Relevant genotype or description	Source or Reference
**ATCC 25922**	*Escherichia coli*	Quality control strain	ATCC collection
**Isogenic panel**			
**IP38**	*Escherichia coli*	MG1655 Δ*ampC* / pK18	This study
**IP41**	*Escherichia coli*	MG1655 Δ*ampC* / pK18-VIM-2	This study
**IP42**	*Escherichia coli*	MG1655 Δ*ampC* / pK18-KPC-2	This study
**IP90**	*Escherichia coli*	MG1655 Δ*ampC* / pK18-VIM-1	This study
**IP93**	*Escherichia coli*	MG1655 Δ*ampC* / pK18-NDM-1	This study
**Clinical panel**			
**K1N**	*Klebsiella pneumoniae*	bla_{NDM-1}, $bla_{OXA-181}$	Timothy Walsh lab, BARNARDS group
**K2N**	*Klebsiella pneumoniae*	bla_{NDM-1}	Timothy Walsh lab, BARNARDS group
**K8N**	*Klebsiella pneumoniae*	bla_{NDM-7}	Timothy Walsh lab, BARNARDS group
**E8N**	*Escherichia coli*	bla_{NDM-5}	Timothy Walsh lab
**E10N**	*Escherichia coli*	bla_{NDM-5}	Baraniak et al., 2019[2]
**E11N**	*Escherichia coli*	bla_{NDM-7}	Timothy Walsh lab
**C1A**	*Escherichia coli*	bla_{NDM-4}	Timothy Walsh lab
**C5A**	*Citrobacter sedlakii*	bla_{NDM-1}	Timothy Walsh lab
**E5A**	*Enterobacter hormaechei*	bla_{VIM-2}	Izdebski et al., 2018[3]
**S4A**	*Serratia marcescens*	bla_{NDM-1}	Timothy Walsh lab, BARNARDS group
**B3H**	*Pseudomonas aeruginosa*	bla_{VIM-2}	Timothy Walsh lab
**P43**	*Pseudomonas aeruginosa*	bla_{IMP-1}	Cuba et al., 2020[4]
**A10K**	*Acinetobacter baumannii*	bla_{NDM-1}, bla_{OXA-98}	Timothy Walsh lab, BARNARDS group
**IEC429**	*Acinetobacter baumannii*	bla_{IMP-1}	Brasiliense et al., 2019[5]

IV. Supplementary Experiments and Methods

a. Cytotoxicity and ADME Data

Table S5: Cytotoxicity against the HepG2 cells and stability in pH 7.4 buffer, plasma, and liver microsomes for compounds **PyC 7** and **12**.

Experiments	Species	Data	PyC 7	PyC 12	Control Compound[#]
Cytotoxicity Screening in HepG2 cells<br>*Control Compound: Tamoxifen*	–	IC_{50} (µM)	>150	-	18.82
		% vehicle control at highest conc.	97.81	-	0.02
Stability of the Test compounds (5 µM) in Mouse and Human Plasma<br>*Control Compound: Propantheline*	Mouse	$t_{1/2}$ (min)	>511.69	>511.69	41.68 (44.45)
		% Remaining at 120 min	~100	94.47	13.60 (16.32)
	Human	$t_{1/2}$ (min)	>511.69	>511.69	21.99 (36.07)
		% Remaining at 120 min	~100	~100	2.37 (5.60)
Metabolic Stability of the Test compounds (1 µM) in Pooled Male Mouse and Human Liver Microsomes<br>*Control Compound: Verapamil*	Mouse	$t_{1/2}$ (min)	>255.85	>255.85	2.61 (3.02)
		In vitro C_{lint} (µL/min/mg protein)	<5.42	<5.42	530.90 (458.93)
	Human	$t_{1/2}$ (min)	>255.85	>255.85	4.51 (4.34)
		In vitro C_{lint} (µL/min/mg protein)	<5.42	<5.42	307.58 (319.44)
Stability of the Test compounds (5 µM) in pH 7.4 Buffer<br>*Control Compound: Chlorambucil*	–	% Remaining at 0 h	100%	–	100%
		% Remaining at 24 h	~ 100%	–	0%

[#]Repeat Analyses are in parentheses

Assay Protocols

Cytotoxicity Screening

HepG2 cells were maintained in Dulbecco's Modified Eagle's Medium (DMEM) supplemented with 10% FBS, 1 × penicillin-streptomycin mixture, 1 × non-essential amino acids (NEAA) and

1% HEPES at 37 ºC in a humidified atmosphere containing 5% CO_2. Cells were detached using trypsin/EDTA, collected by centrifugation (150g, 5 min), and resuspended in cell culture medium at 8 × 10^4 cells/mL. Cell suspensions (100 µL) were seeded into 96-well plates and incubated overnight at 37 °C, 5% CO_2 atmosphere. The working solution for Test and control compounds were prepared by 3-fold serial dilution (8 concentrations) from 30 mM DMSO stock solutions and diluted in culture medium containing 10% FBS (final DMSO concentration, 0.5%). The medium was replaced with 100 µL of working solution, and DMSO (0.5%) was used as vehicle control. After 48 h incubation, pre-mixed Cell Titer-Glo reagent (50 µL per well) was added, and plates were shaken for 5 min and incubated at room temperature for 10 min. Aliquots (100 µL) were transferred to a white flat bottom opaque 96 well plates and luminescence was measured using a plate reader. All experiments were performed in triplicate and cell viability was expressed as a percentage relative to the DMSO control.

Plasma Stability Assay

The working solutions (1 mM) of the test compounds and the control compound were prepared in DMSO. Propantheline bromide was used as a positive control. Working solutions (2 µL) were spiked into preincubated plasma (398 µL) to afford a final concentration of 5 µM (0.5% DMSO). The assay was performed in duplicate. For time zero, aliquots (50 µL) were immediately quenched with acetonitrile (400 µL) containing 3% FA and internal standards (100 nM Tolbutamide, 500 nM Labetalol and 2 µM Ketoprofen). Remaining aliquots (50 µL) were incubated at 37 °C with shaking (60 rpm) and collected at 15, 30, 60, and 120 min, followed by quenching as described above. Samples were vortexed for 5 min and centrifuged (3220g, 40 min, 4 °C) to precipitate protein. Supernatants (100 µL) were diluted with water (100 µL) and analyzed by LC–MS/MS.

Metabolic Stability Test in Liver Microsomes

A master solution was prepared with liver microsomes (0.556 mg/mL) and phosphate buffer (111.11 mM) in ultra-pure water (30 µL). NADPH solution (10 mM) was prepared in ultra-pure water. Test compound (80 nL of 1 mM) or control compound (80 nL of 1 mM) was added to 72 µL of the master solution and the mixture was pre-warmed at 37 °C for 10 minutes. The reaction was started with addition of NADPH solution (8 µL of 10 mM) and then carried out at 37 °C. The final concentration of NADPH was kept at 1 mM. The negative control samples were

prepared by replacing NADPH solution with 8 μL of ultra-pure water to exclude the misleading factor that may result from instability of the chemical itself. This study was performed in duplicate and negative controls were prepared in singlet. Verapamil was used as a positive control in this study. The final concentration of the test compound or control compound was 1 μM and the DMSO concentration in the incubation system was 0.1% (v/v). The test compound samples (10 μL of each) were transferred from the reaction solution at each time point (0, 15, 30, 45 and 60 minutes) into one well of a new plate containing 120 μL of cold acetonitrile (200 nM labetalol, 100 nM tolbutamide and 100 nM ketoprofen) to quench the reactions. The samples were centrifuged at 3,220 g for 45 minutes to precipitate protein. The supernatant (40 μL) mixed with 40 μL of ultra-pure water was used for LC-MS/MS analysis.

Chemical Stability in pH 7.4 Buffer

The working solutions of the test compounds (500 μM) and control compound chlorambucil (500 μM) were prepared in DMSO. Matrix was prepared with 100 mM Na_2HPO_4/ NaH_2PO_4 solution at pH 7.4. Working solution (2 μL) of each sample was placed in order into their proper 96-well rack. Pre-incubated matrix (198 μL) was added into each vial of sample plate to achieve a final concentration of 5 μM. The assay was performed in duplicate. The sample plate was transferred to a shaker and incubated in a 37 ºC water bath at approximately 60 rpm. Glass vials were removed at designated time points (0, 2, 4, 6, and 24 h). The reaction initiation was staggered so that all samples were quenched simultaneously with 1000 μL of room-temperature quench solution (acetonitrile containing 3% FA and internal standards: 300 nM labetalol, 100 nM tolbutamide and 1 μM ketoplofen). Samples were vortexed for 1 min and centrifuged at 2500 g for 10 min at 4 °C. Then, 100 μL of the supernatant was transferred to a new plate, diluted with 100 μL or 200 μL water according to the LC-MS signal response and peak shape, mixed well and analyzed samples using LC-MS/MS.

b. IC_{50} Against Human MBL-fold Nuclease SNM1C[6,7]

Table S6: IC_{50} values of **PyC 7**, **12**, **13**, **17**, and **18** against human MBL-fold nuclease SNM1C

	IC_{50} (μM)				
**Enzyme**	**PyC 7**	**PyC 12**	**PyC 13**	**PyC 17**	**PyC 18**
**SNM1C**	>100	>100	22 ± 4	>100	>100

Real-time fluorescence assay against SNM1C with four technical replicates. Concentrations used: [SNM1C] = 16 nM and [DNA reporter] = 25 nM. Inhibitor concentrations ranged from 100 μM to 5.08 nM

Assay Protocol: The protocol was carried out according to the previously reported procedure.[8] Briefly, reactions were carried out in black 384-well microplates in a total volume of 25 μL per well. A ssDNA substrate containing a 5′ FITC-conjugated T and an internal BHQ-conjugated T was used. Both FITC (fluorescein isothiocyanate) and BHQ1 (black hole quencher 1) are conjugated to a thymidine nucleotide. The oligonucleotide substrate was purchased from Eurofins Genomics.

Oligo: [FITC]-TAA TTA ATA ATA GAT CAC CT-[BHQ1]

The reaction comprised 25 nM DNA substrate, indicated amounts of protein and reaction buffer: 20 mM HEPES-KOH (pH 7.5), 10 mM $MgCl_2$, 50 mM KCl, 0.5 mM TCEP, 0.05% Triton X-100, and 5% (*v*/*v*) glycerol. Inhibitors were solubilised in DMSO and serially diluted to desired inhibitor concentration with DMSO concentration kept at a constant 1% (*v*/*v*) in the final reaction mixture. Proteins were incubated for 30 min at RT with the stated compound where applicable, before the reaction was started with the addition of the ssDNA oligo substrate. The fluorescence spectra were measured every 150 s, for 35 min, at 37 °C with a PHERAstar FSX (BMG Labtech) (excitation 485 nm, emission 520 nm).

c. ChromLogD Measurement

ChromLogD was measured using a method adapted from Valko *et al.*[9] Briefly, samples were analysed using an ACQUITY H-Class PLUS UPLC instrument (Waters) equipped with a Gemini NX C18 column (50 mm × 2 mm, 3 μM pore size, Phenomenex) using a gradient of 0-100% MeCN in: 0.1 % formic acid (pH 2); 50 mM ammonium acetate (pH 7.4); or 50 mM ammonium

acetate (pH 10.5). Chromatographic Hydrophobicity Index (CHI) were obtained by comparing retention times to a calibration curve generated from 10 compounds with known CHI values[9] and converted to ChromLogD using the formula: ChromlogD = (0.088*CHI) – 2.[10]

V. References

1. Schrödinger Release 2025-3: Maestro, Schrödinger, LLC, New York, NY, 2025.

2. Baraniak, A.; Machulska, M.; Zabicka, D.; Literacka, E.; Izdebski, R.; Urbanowicz, P.; Bojarska, K.; Herda, M.; Kozinska, A.; Hryniewicz, W.; Gniadkowski, M.; Nord-Pas Group. Towards Endemicity: Large-Scale Expansion of the NDM-1-Producing *Klebsiella pneumoniae* ST11 Lineage in Poland, 2015-16. *J. Antimicrob. Chemother.* **2019**, *74*, 3199–3204. https://doi.org/10.1093/jac/dkz315

3. Izdebski, R.; Baraniak, A.; Zabicka, D.; Sekowska, A.; Gospodarek-Komkowska, E.; Hryniewicz, W.; Gniadkowski, M. VIM/IMP Carbapenemase-Producing *Enterobacteriaceae* in Poland: Epidemic *Enterobacter hormaechei* and *Klebsiella oxytoca* Lineages. *J. Antimicrob. Chemother.* **2018**, *73*, 2675–2681. https://doi.org/10.1093/jac/dky257

4. Cuba, G. T.; Rocha-Santos, G.; Cayo, R.; Streling, A. P.; Nodari, C. S.; Gales, A. C.; Pignatari, A. C. C.; Nicolau, D. P.; Kiffer, C. R. V. In Vitro Synergy of Ceftolozane/Tazobactam in Combination with Fosfomycin or Aztreonam against MDR *Pseudomonas aeruginosa*. *J. Antimicrob. Chemother.* **2020**, *75*, 1874–1878. https://doi.org/10.1093/jac/dkaa095

5. Brasiliense, D.; Cayo, R.; Streling, A. P.; Nodari, C. S.; Barata, R. R.; Lemos, P. S.; Massafra, J. M.; Correa, Y.; Magalhaes, I.; Gales, A. C.; Sodre, R. Diversity of Metallo-β-Lactamase-Encoding Genes Found in Distinct Species of *Acinetobacter* Isolated from the Brazilian Amazon Region. *Mem. Inst. Oswaldo Cruz* **2019**, *114*, e190020. https://doi.org/10.1590/0074-02760190020

6. Karim, M. F.; Liu, S.; Laciak, A. R.; Volk, L.; Koszelak-Rosenblum, M.; Lieber, M. R.; Wu, M.; Curtis, R.; Huang, N. N.; Carr, G.; Zhu, G. Structural Analysis of the Catalytic Domain of Artemis Endonuclease/SNM1C Reveals Distinct Structural Features. *J. Biol. Chem.* **2020**, *295*, 12368-12377. https://doi.org/10.1074/jbc.RA120.014136

7. Yosaatmadja, Y.; Baddock, H. T.; Newman, J. A.; Bielinski, M.; Gavard, A. E.; Mukhopadhyay, S. M. M.; Dannerfjord, A. A.; Schofield, C. J.; McHugh, P. J.; Gileadi, O. Structural and Mechanistic Insights into the Artemis Endonuclease and Strategies for Its Inhibition. *Nucleic Acids Res.* **2021**, *49*, 9310–9326. https://doi.org/10.1093/nar/gkab693

8. (i) Bielinski, M.; Henderson, L. R.; Yosaatmadja, Y.; Swift, L. P.; Baddock, H. T.; Bowen, M. J.; Brem, J.; Jones, P. S.; McElroy, S. P.; Morrison, A.; Speake, M.; van Boeckel, S.; van Doornmalen, E.; van Groningen, J.; van den Hurk, H.; Gileadi, O.; Newman, J. A.; McHugh, P. J.; Schofield, C. J. Cell-Active Small Molecule Inhibitors Validate the SNM1A DNA Repair Nuclease as a Cancer Target. *Chem. Sci.* **2024**, 15, 8227–8241. https://doi.org/10.1039/D4SC00367E. (ii) Lee, S. Y.; Brem, J.; Pettinati, I.; Claridge, T. D. W.; Gileadi, O.; Schofield, C. J.; McHugh, P. J. Cephalosporins Inhibit Human Metallo-β-Lactamase Fold DNA Repair Nucleases SNM1A and SNM1B/Apollo. *Chem. Commun.* **2016**, *52* (40), 6727–6730. https://doi.org/10.1039/C6CC00529B

9. Valkó, K.; Bevan, C.; Reynolds, D. Chromatographic Hydrophobicity Index by Fast-Gradient RP-HPLC: A High-Throughput Alternative to log P/log D. *Anal. Chem.* **1997**, *69*, 2022–2029. https://doi.org/10.1021/ac961242d.

10. Young, R. J.; Green, D. V. S.; Luscombe, C. N.; Hill, A. P. Getting Physical in Drug Discovery II: The Impact of Chromatographic Hydrophobicity Measurements and Aromaticity. *Drug Discovery Today* **2011**, *16*, 822–830. https://doi.org/10.1016/j.drudis.2011.06.001

VI. NMR Spectra of Novel Intermediates and Final Compounds

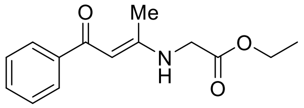

Compound **21**

^{1}H NMR (400 MHz, $CDCl_3$)

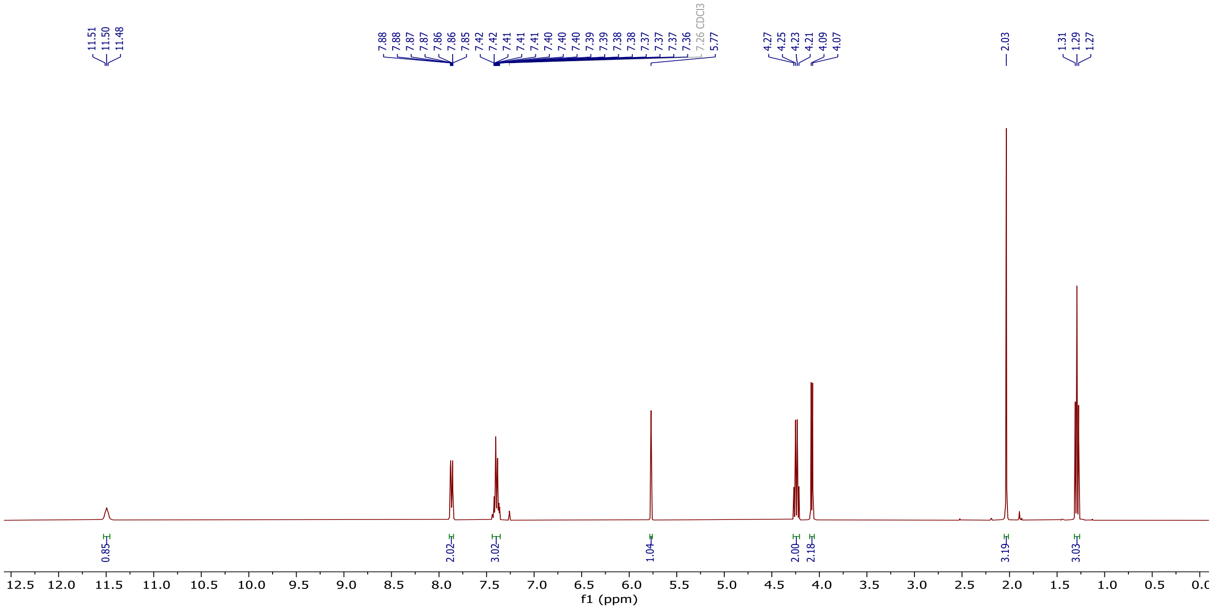

^{13}C NMR (101 MHz, $CDCl_3$)

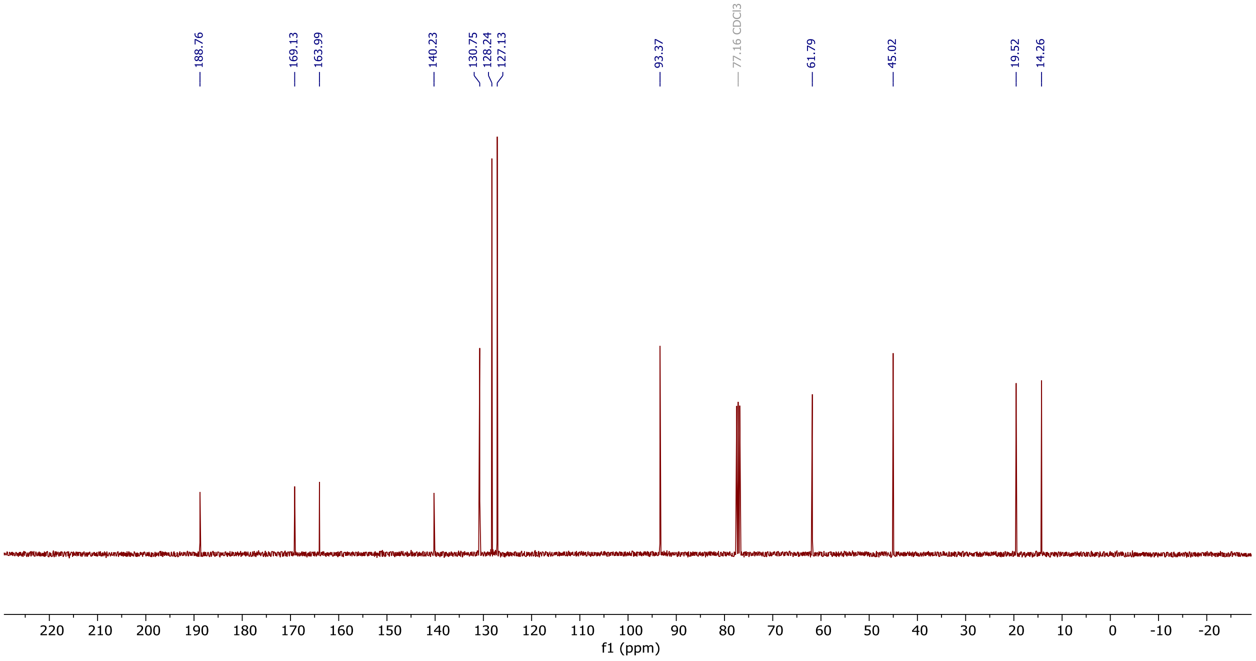

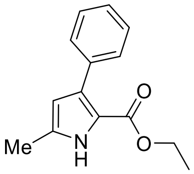

Compound **22**

^1H NMR (400 MHz, $CDCl_3$)

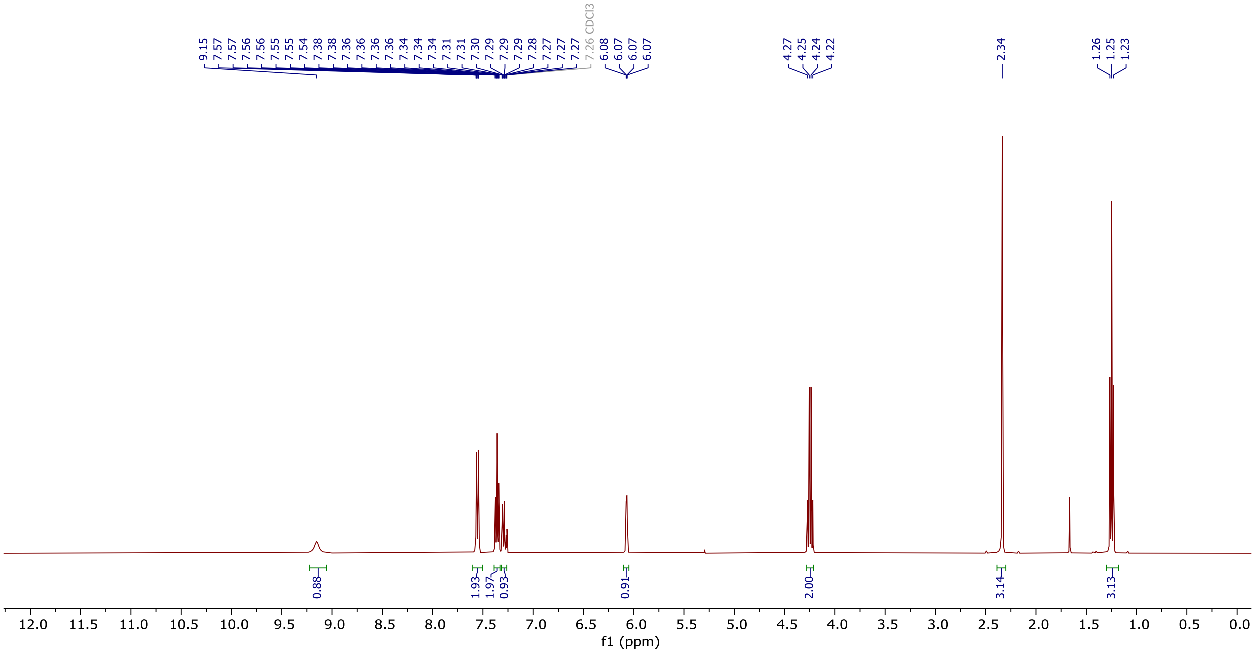

^{13}C NMR (101 MHz, $CDCl_3$)

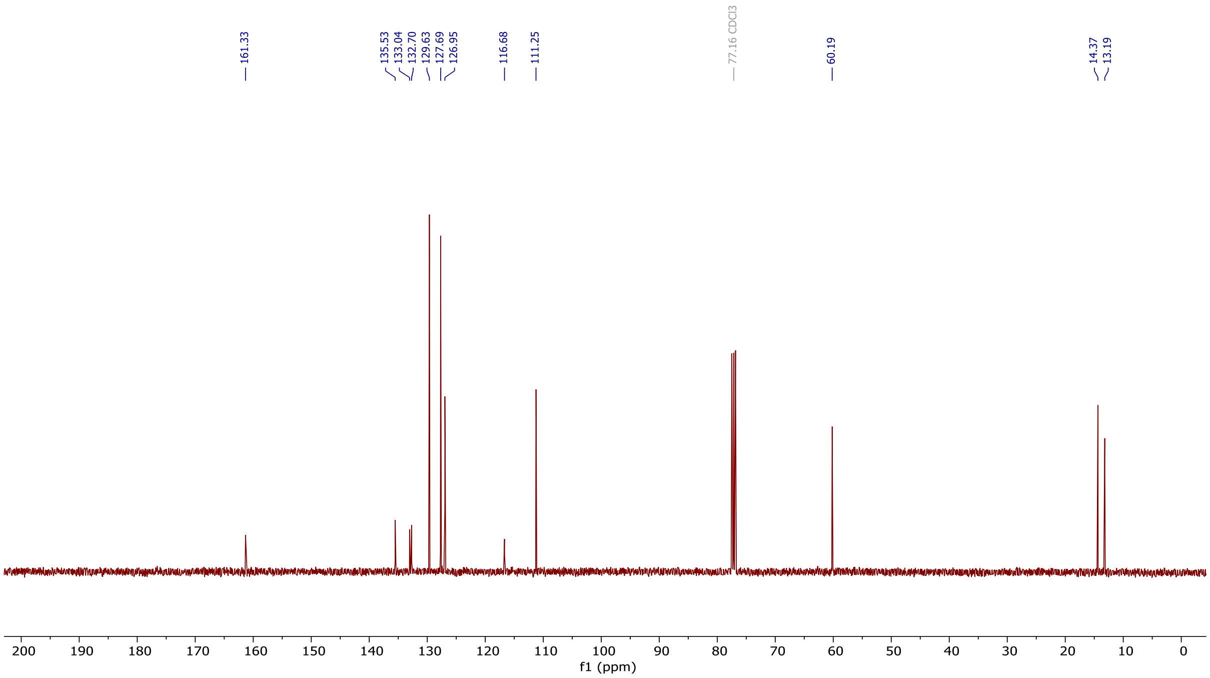

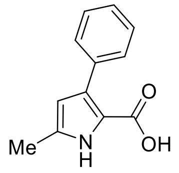

PyC 1

^{1}H NMR (500 MHz, DMSO-d_6)

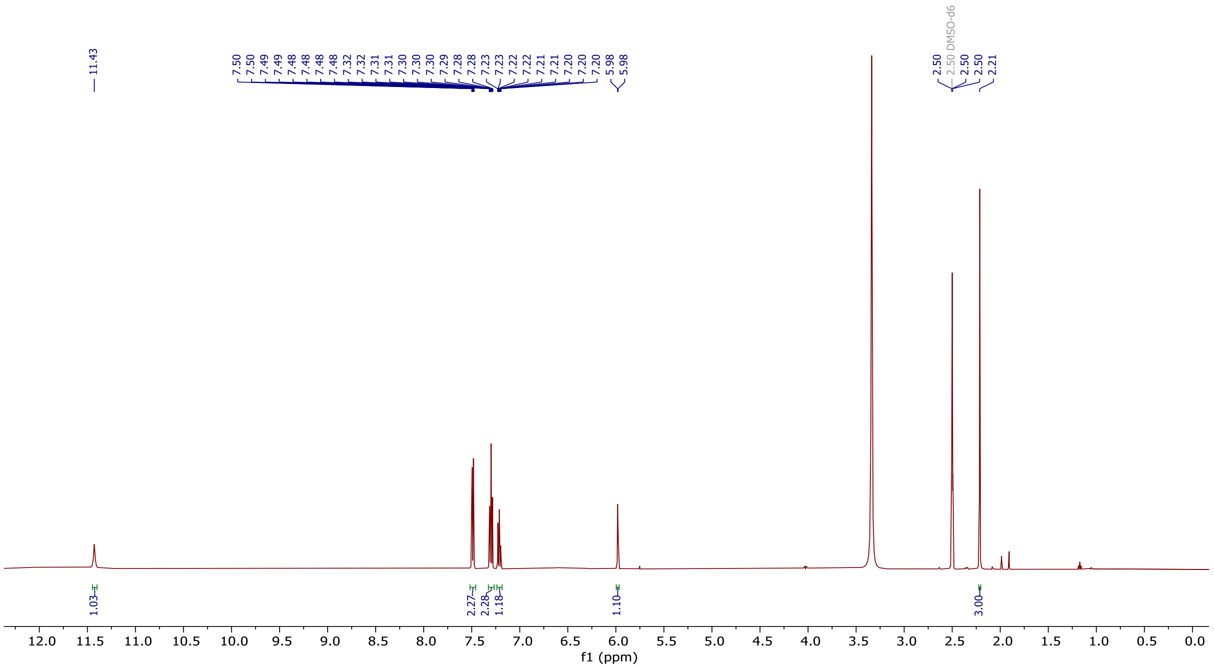

^{13}C NMR (126 MHz, DMSO-d_6)

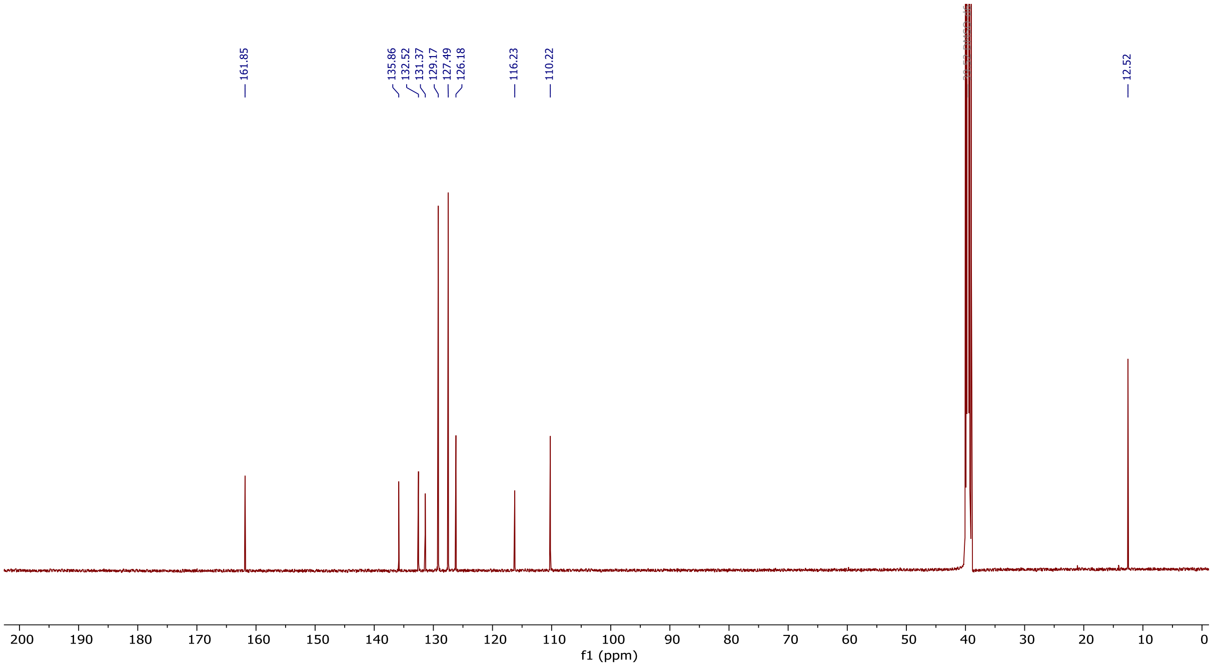

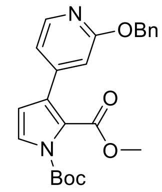

Compound **27**

^{1}H NMR (400 MHz, $CDCl_3$)

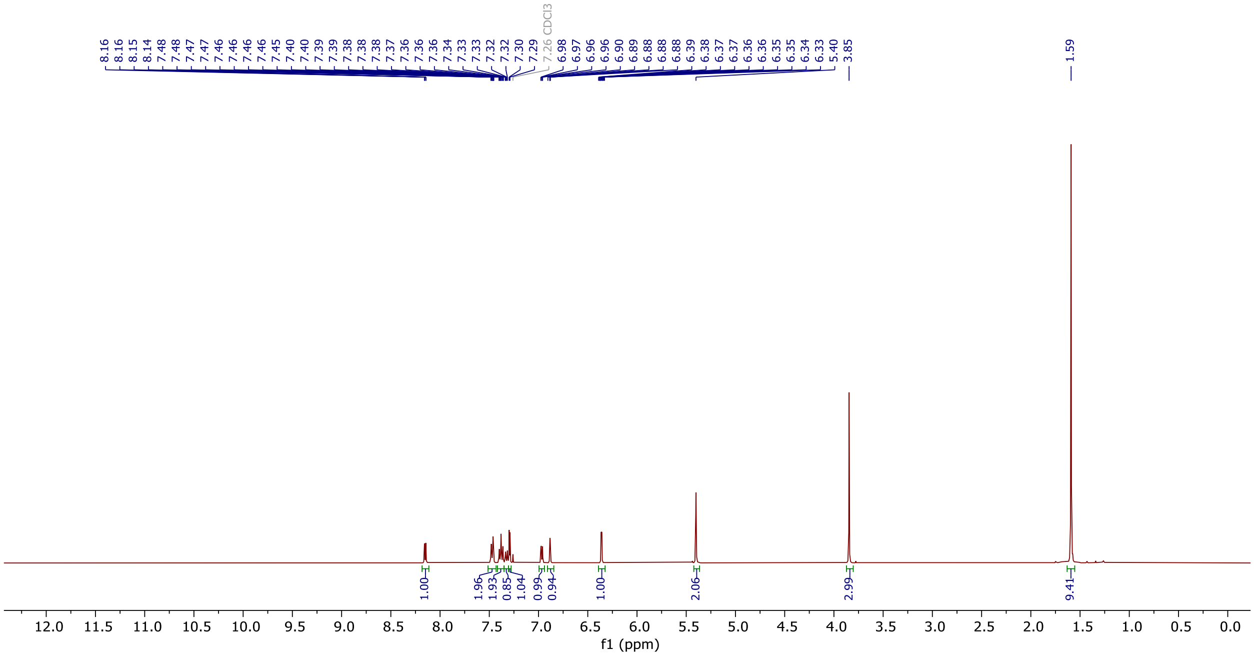

^{13}C NMR (101 MHz, $CDCl_3$)

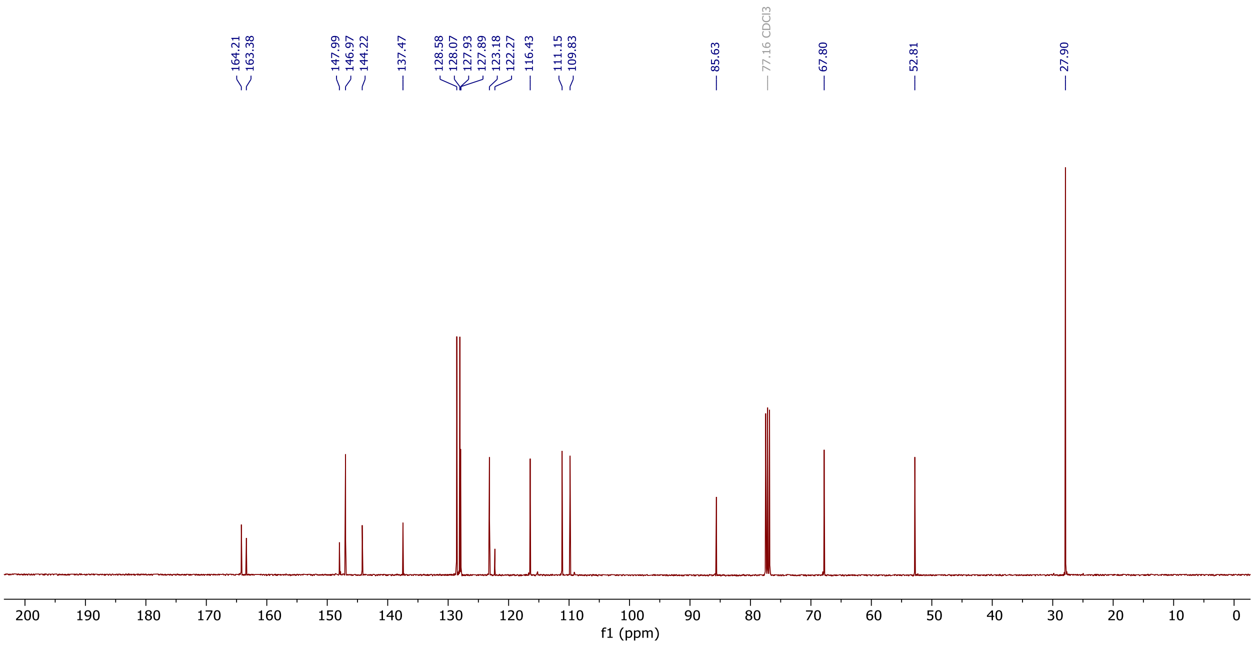

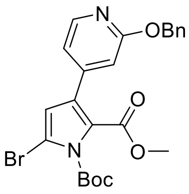

Compound **29**

^{1}H NMR (400 MHz, $CDCl_3$)

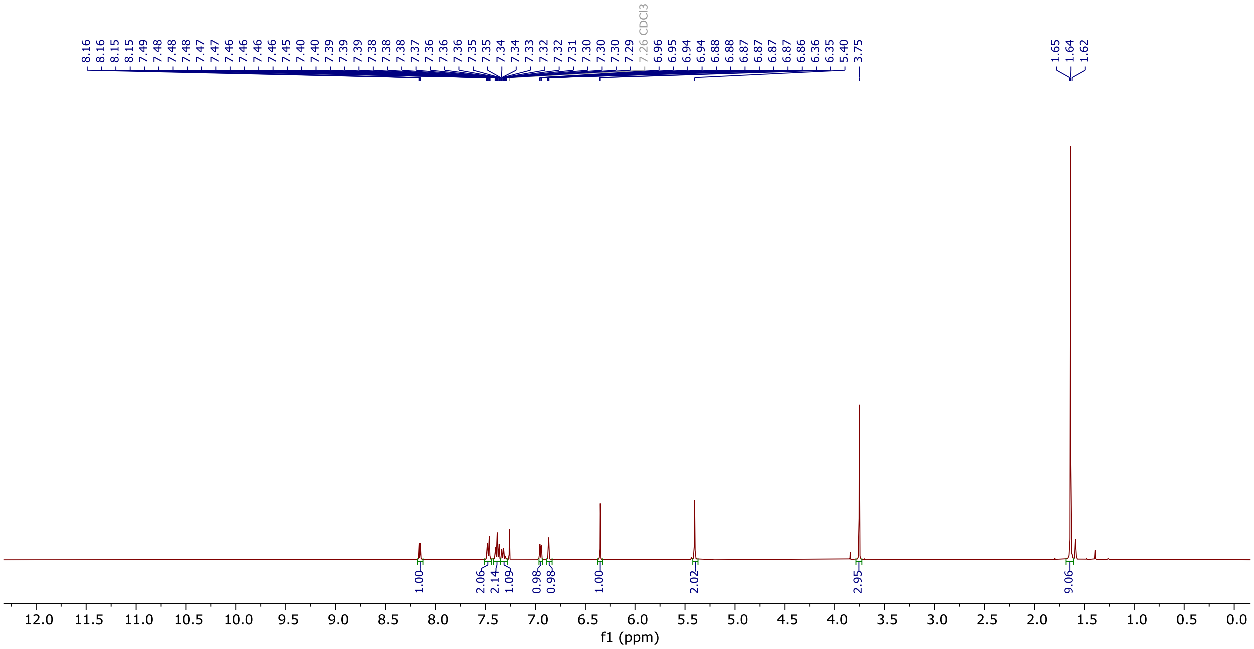

^{13}C NMR (101 MHz, $CDCl_3$)

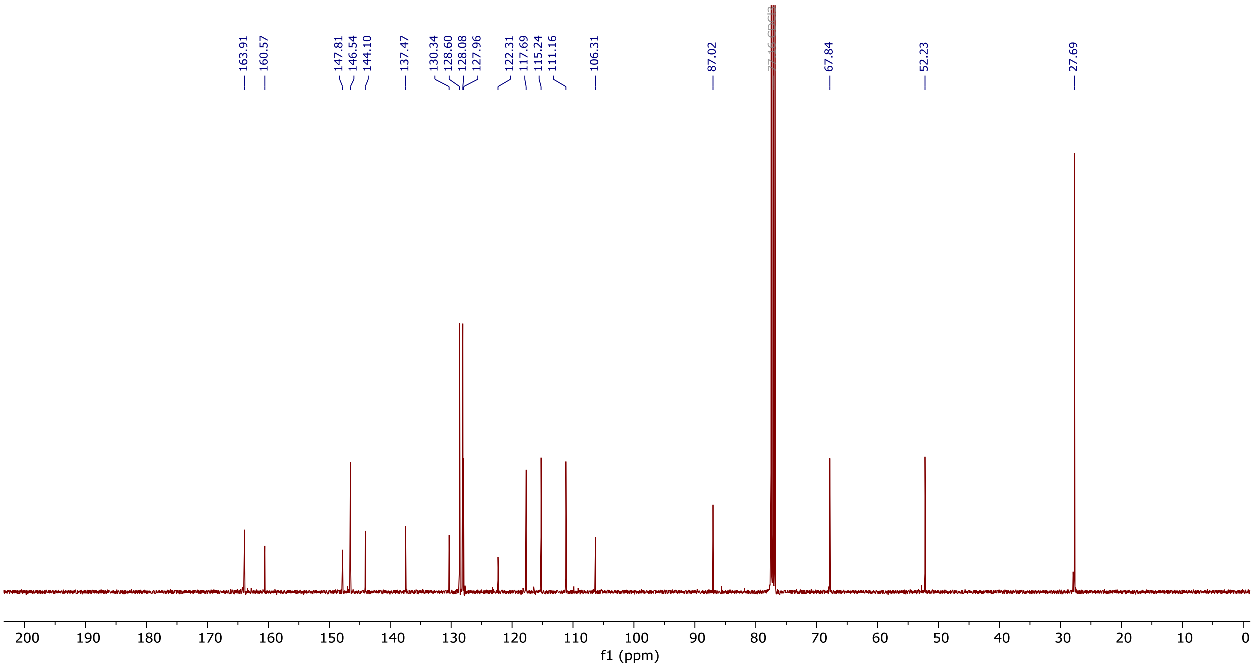

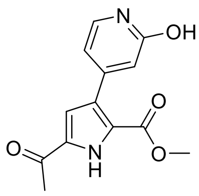

Compound **31**

^{1}H NMR (400 MHz, DMSO-d_6)

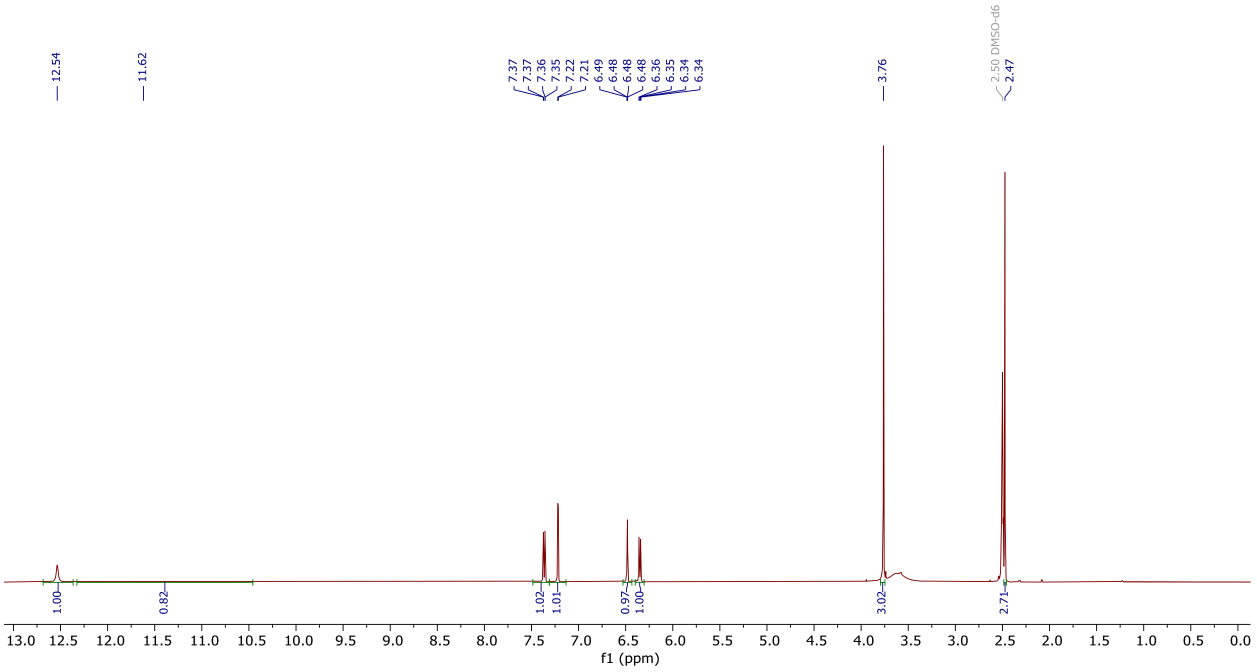

^{13}C NMR (101 MHz, DMSO-d_6)

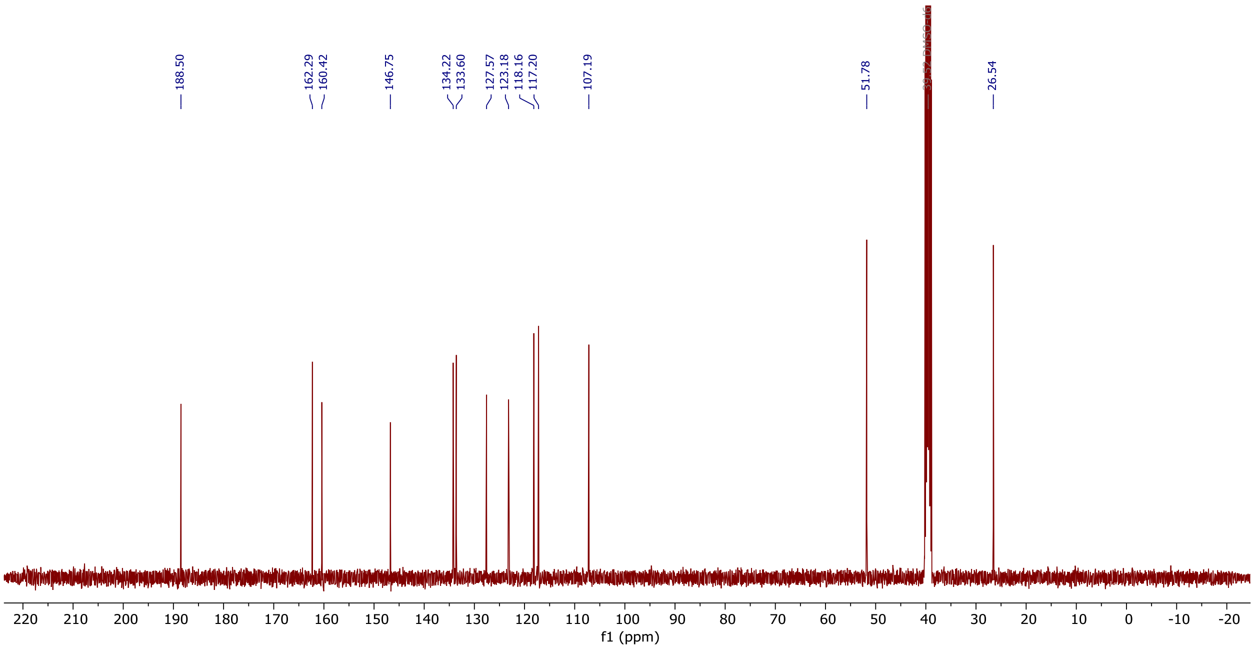

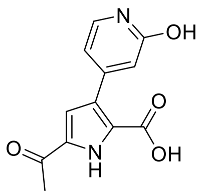

PyC 3

^{1}H NMR (400 MHz, DMSO-d_6)

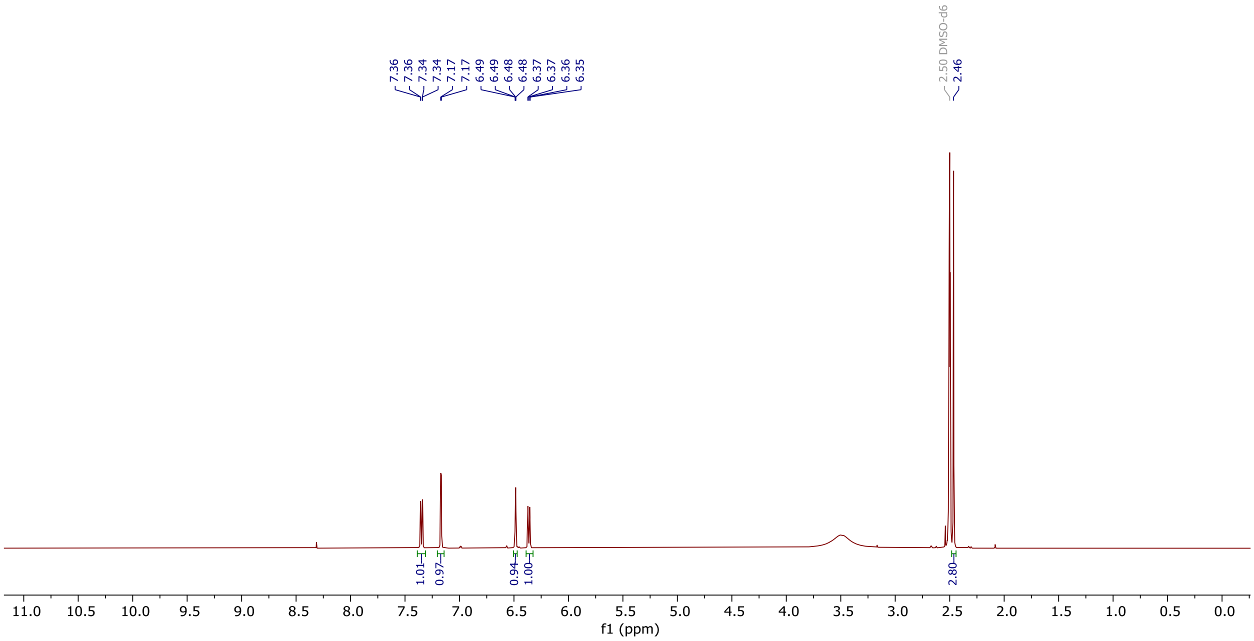

^{13}C NMR (101 MHz, DMSO-d_6)

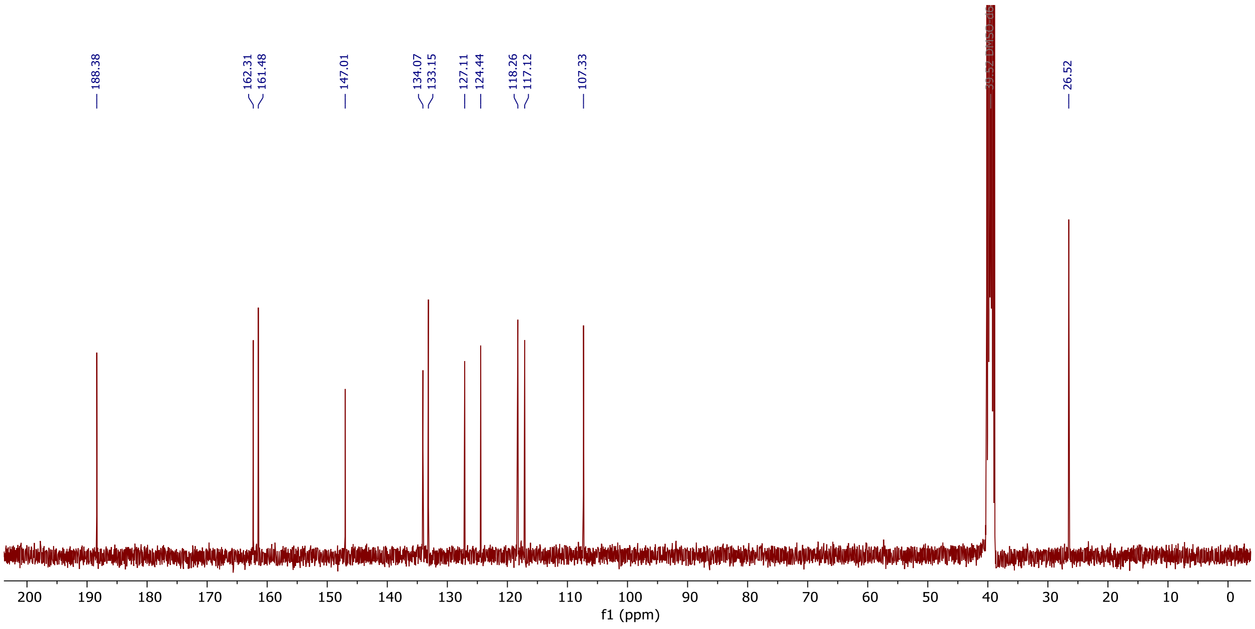

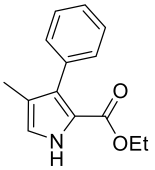

Compound **34**

^{1}H NMR (400 MHz, $CDCl_3$)

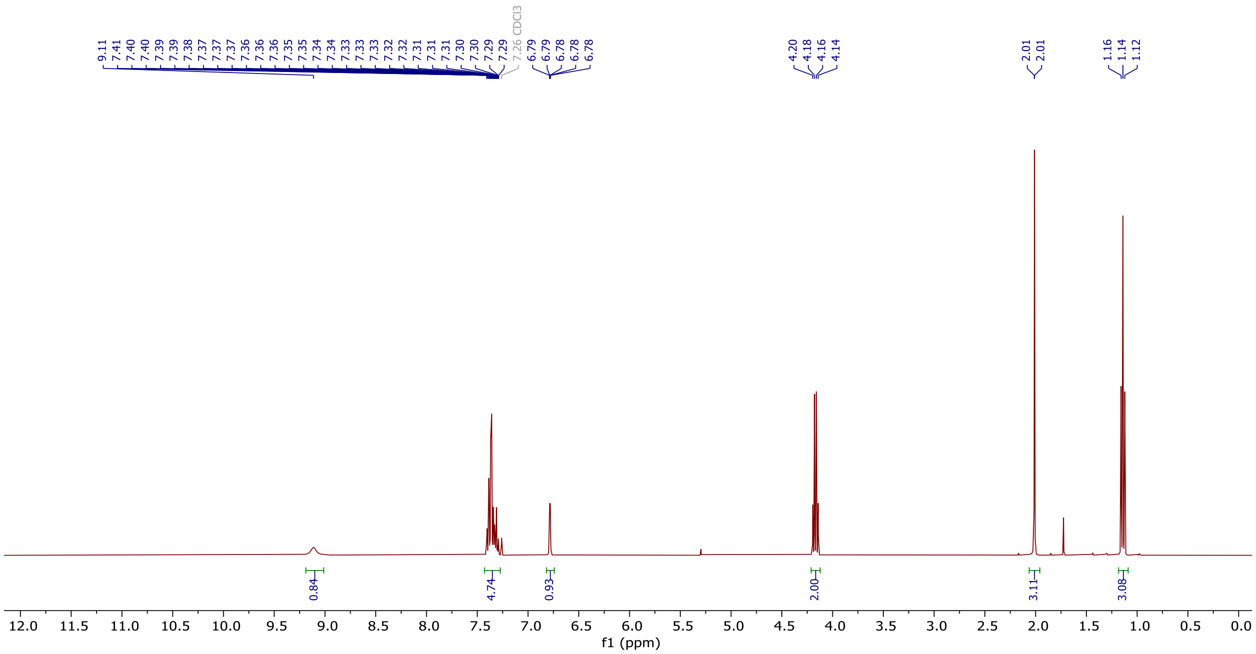

^{13}C NMR (101 MHz, $CDCl_3$)

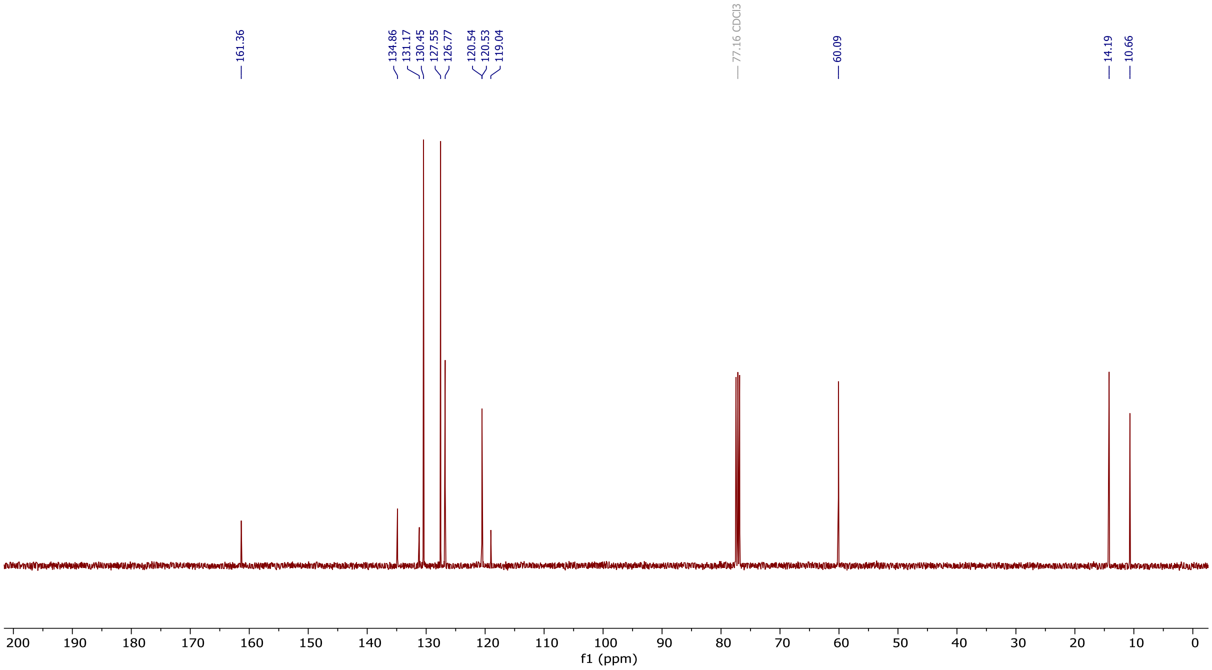

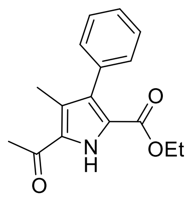

Compound **36**

^{1}H NMR (400 MHz, $CDCl_3$)

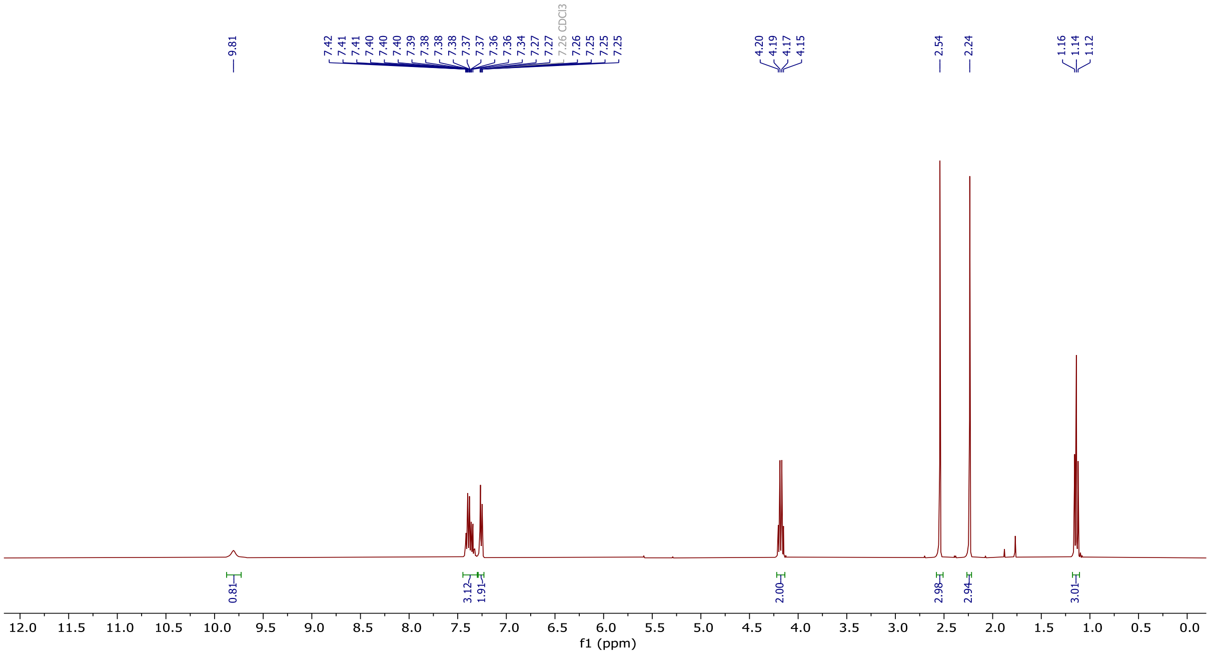

^{13}C NMR (101 MHz, $CDCl_3$)

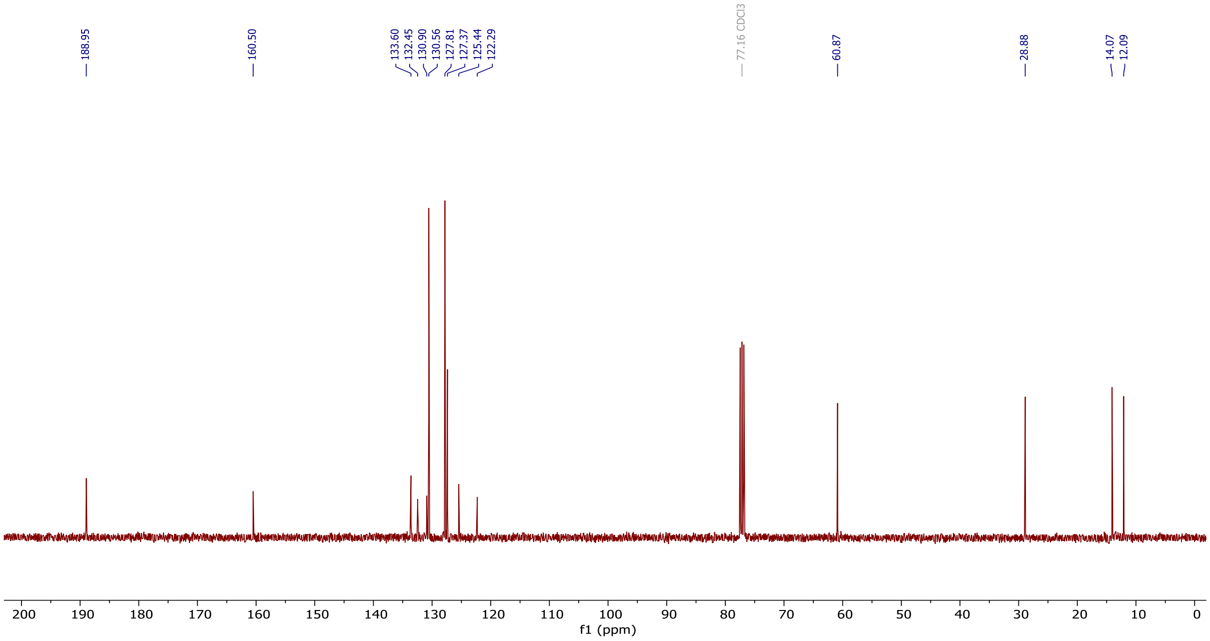

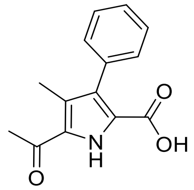

PyC 4a

^{1}H NMR (600 MHz, DMSO-d_6)

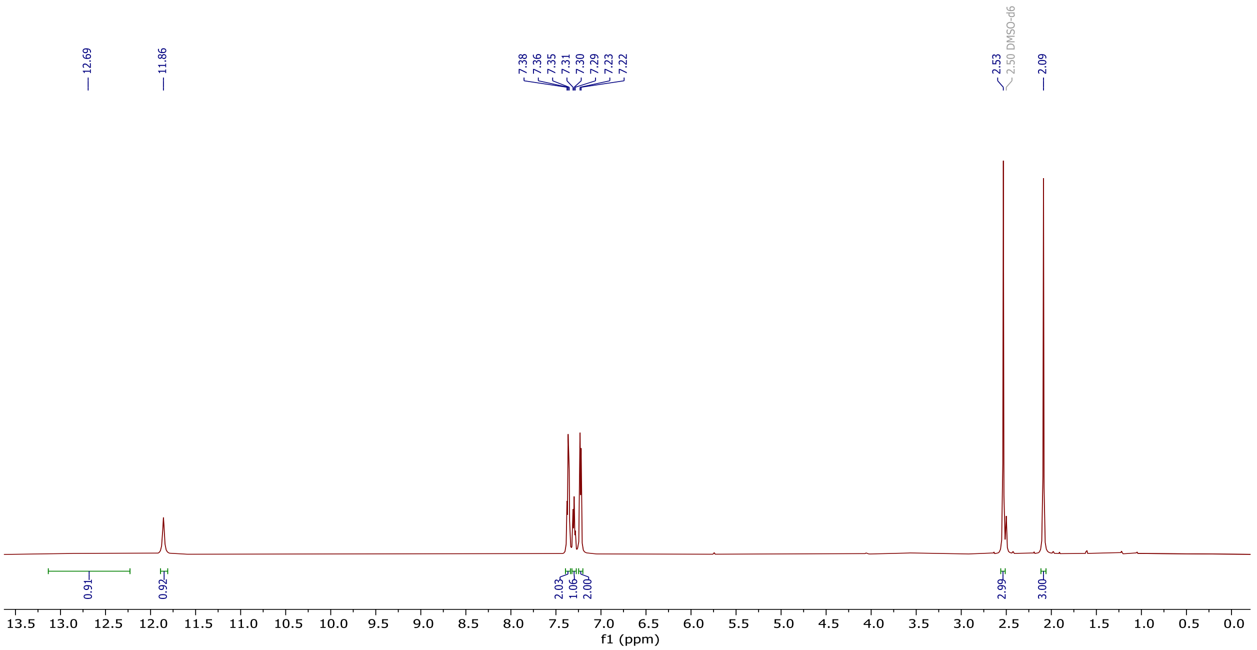

^{13}C NMR (151 MHz, DMSO-d_6)

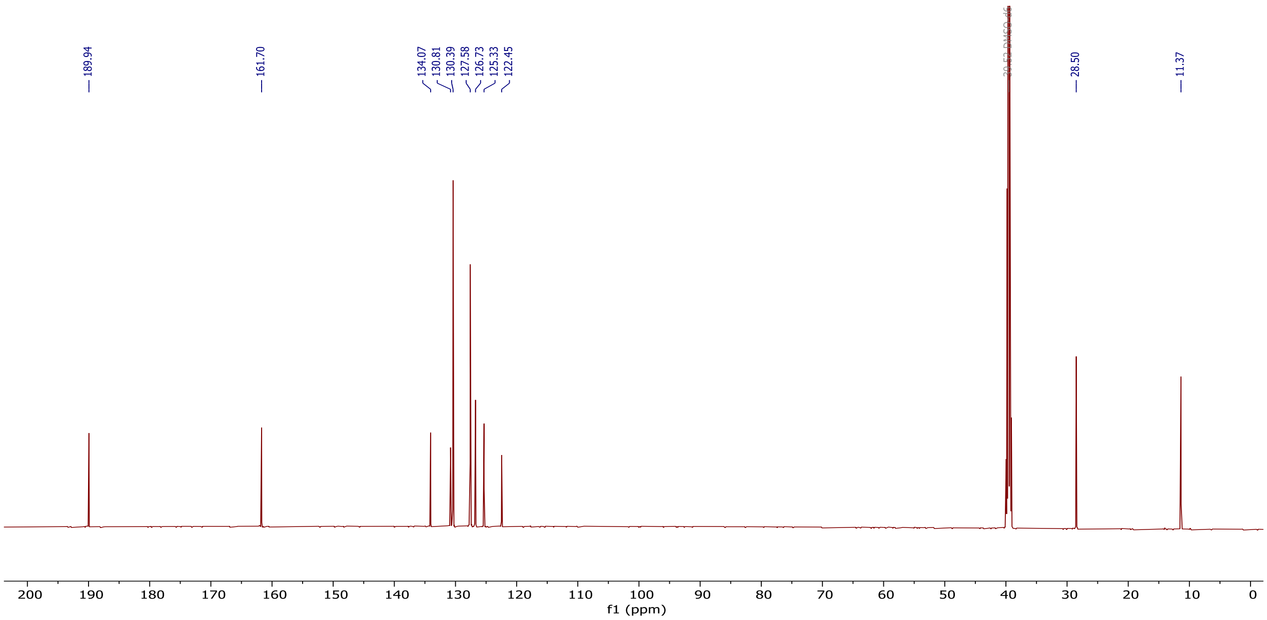

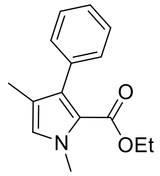

Compound **35**

^{1}H NMR (400 MHz, $CDCl_3$)

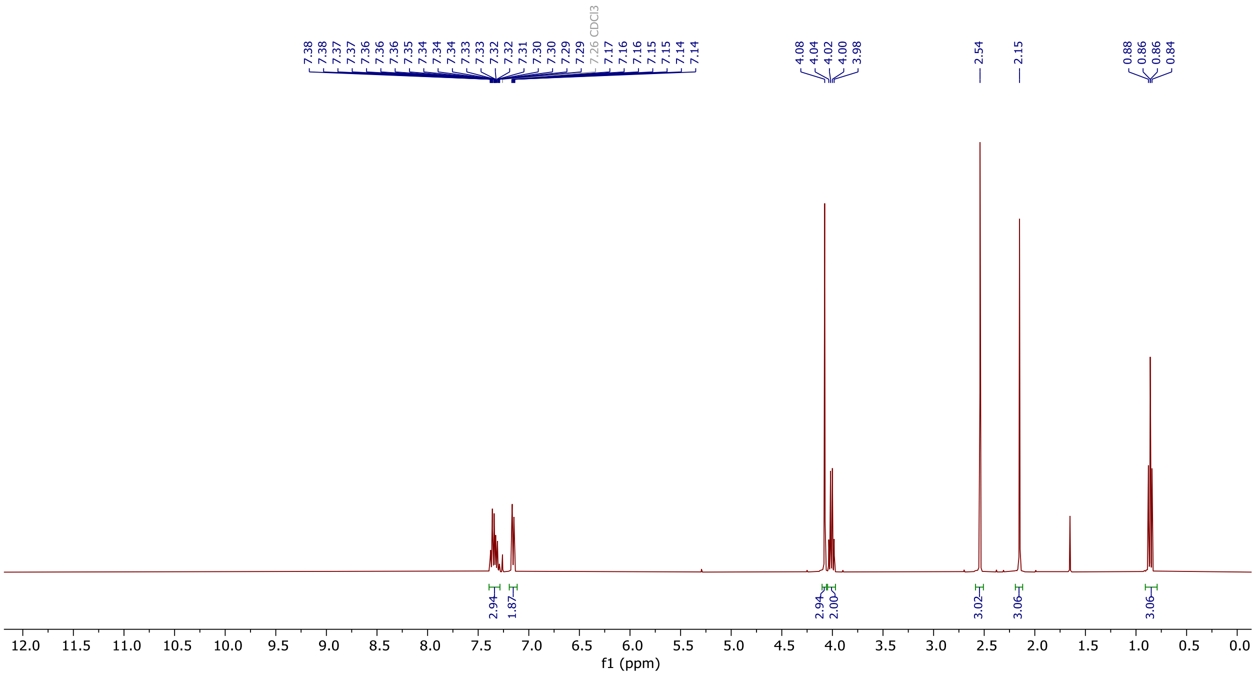

^{13}C NMR (101 MHz, $CDCl_3$)

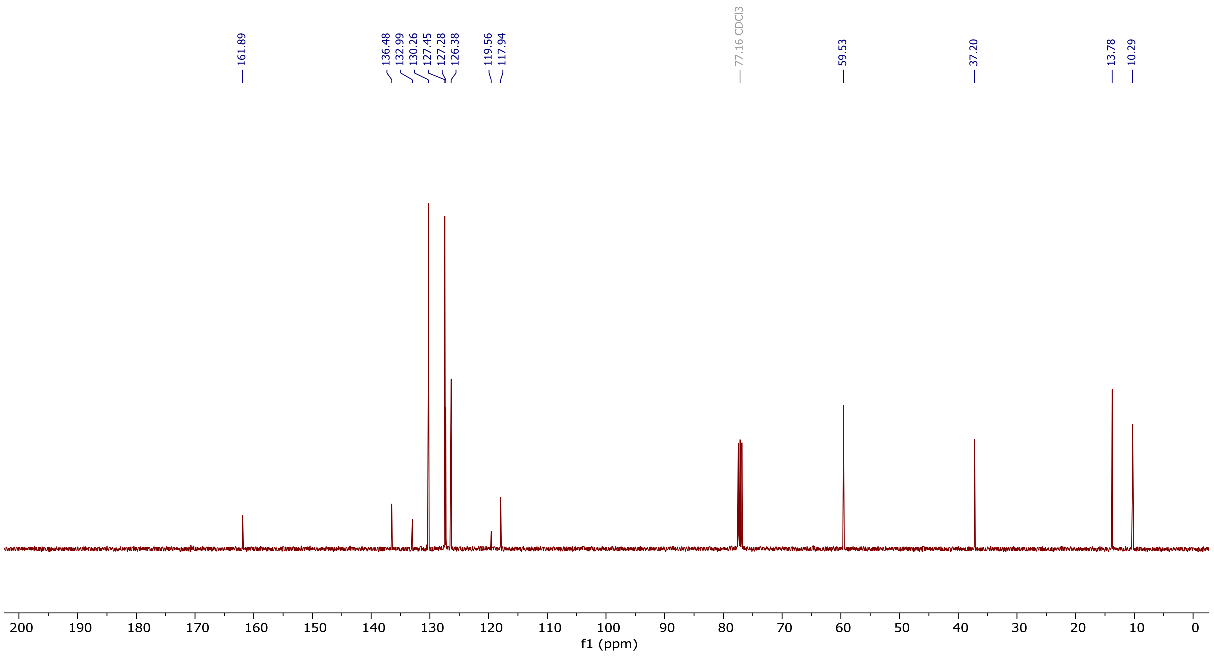

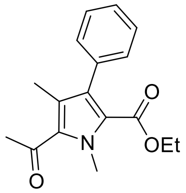

Compound **37**

^{1}H NMR (400 MHz, $CDCl_3$)

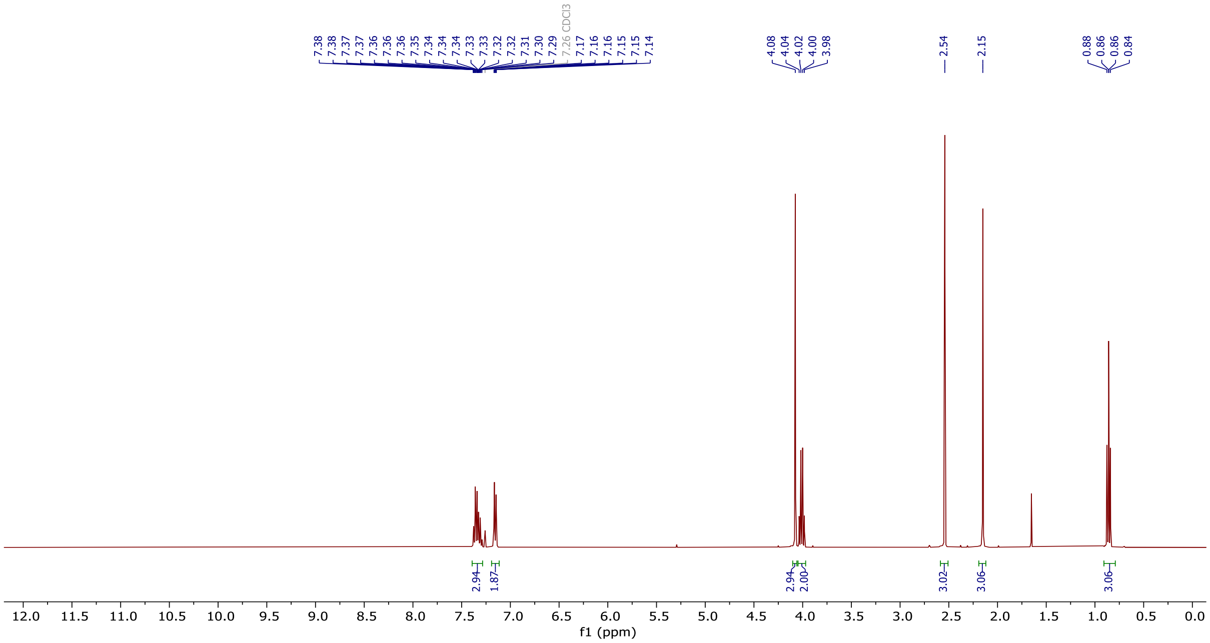

^{13}C NMR (101 MHz, $CDCl_3$)

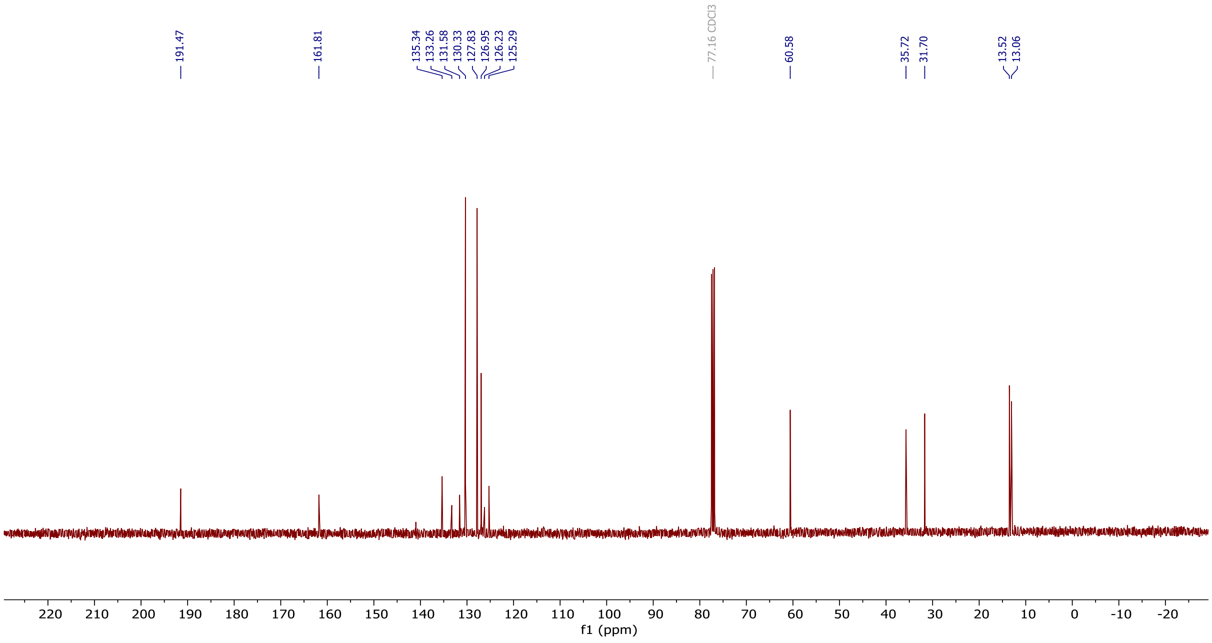

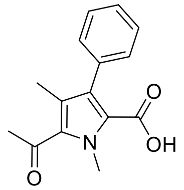

PyC 4b

^{1}H NMR (400 MHz, DMSO-d_6)

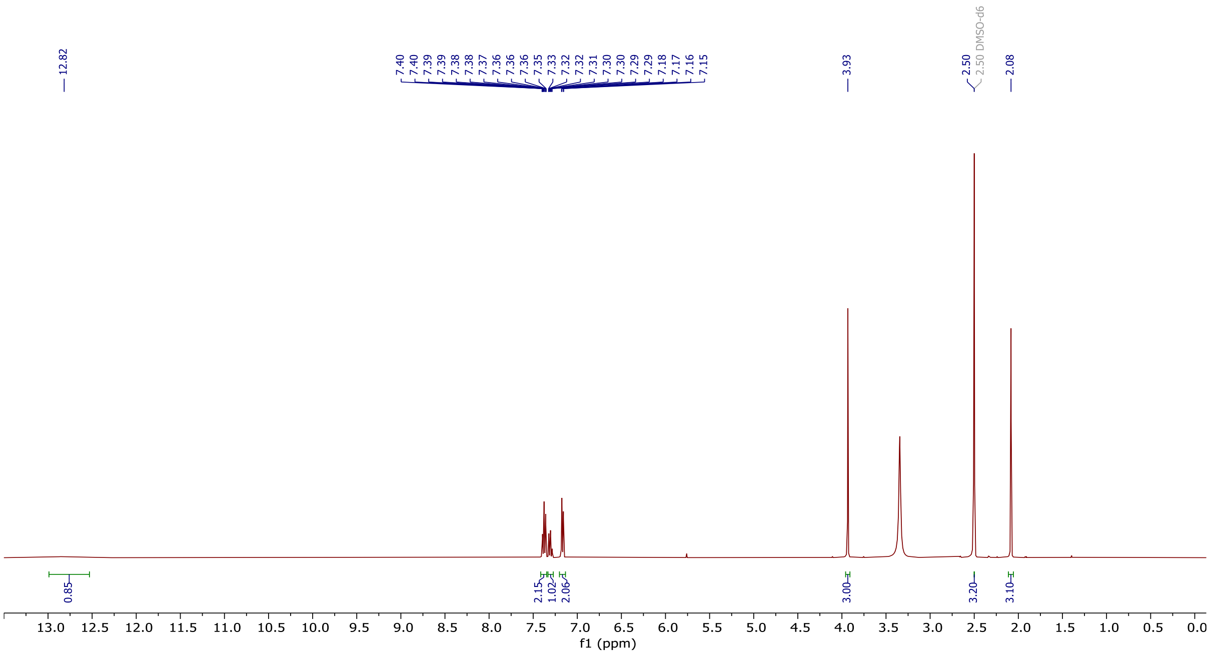

^{13}C NMR (126 MHz, DMSO-d_6)

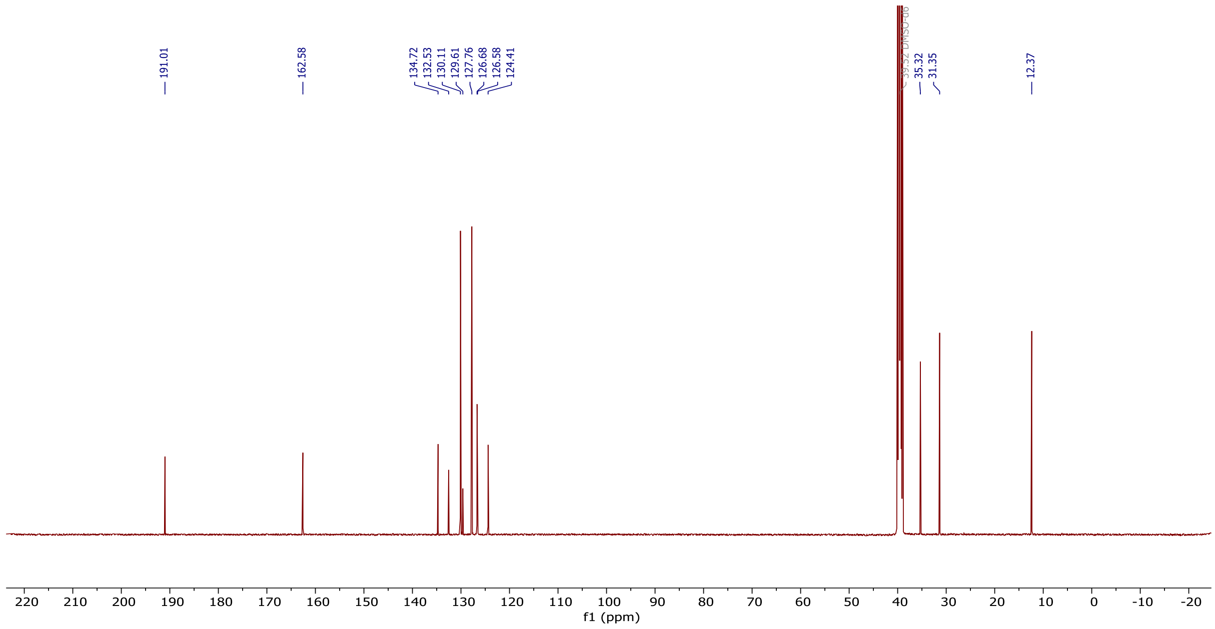

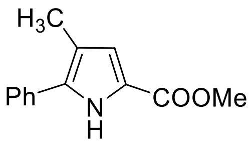

Compound **40a**

^{1}H NMR (400 MHz, $CDCl_3$)

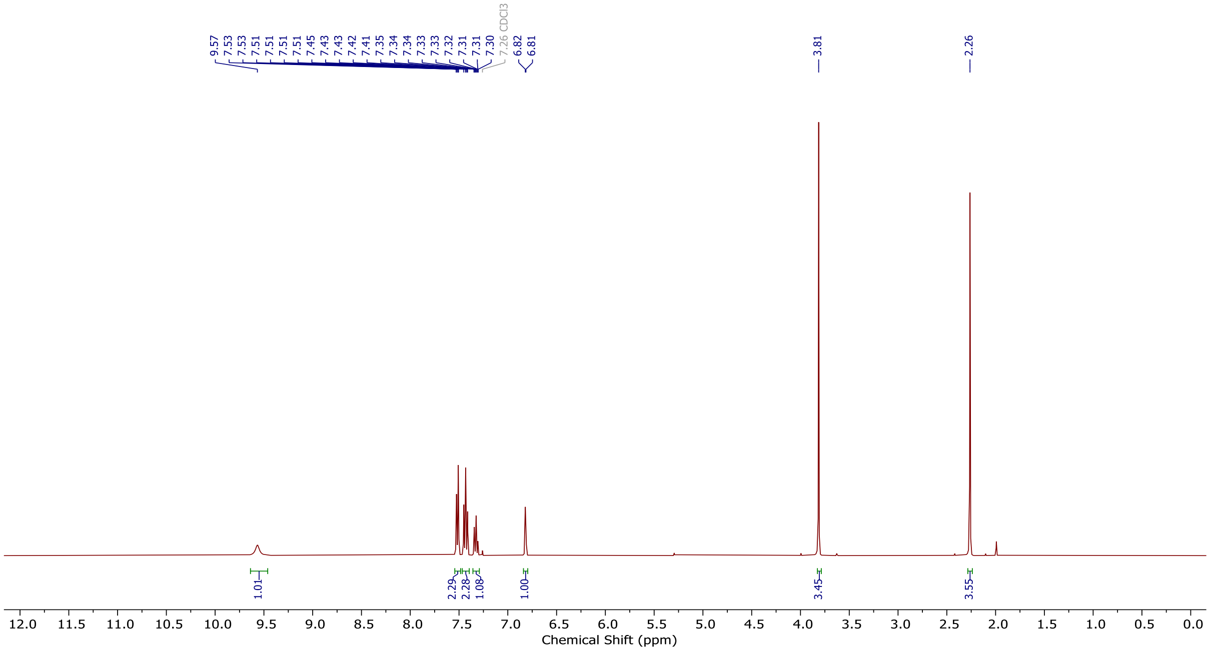

^{13}C NMR (101 MHz, $CDCl_3$)

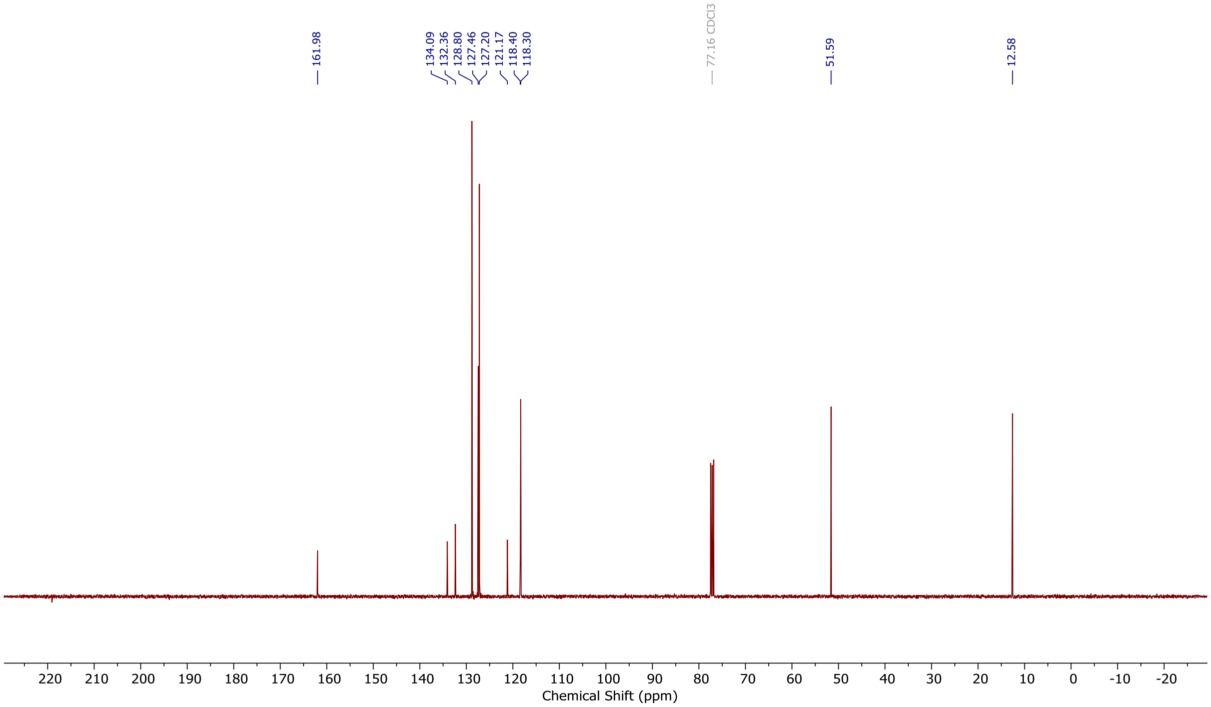

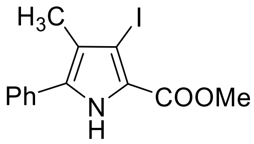

Compound **41a**

^{1}H NMR (400 MHz, $CDCl_3$)

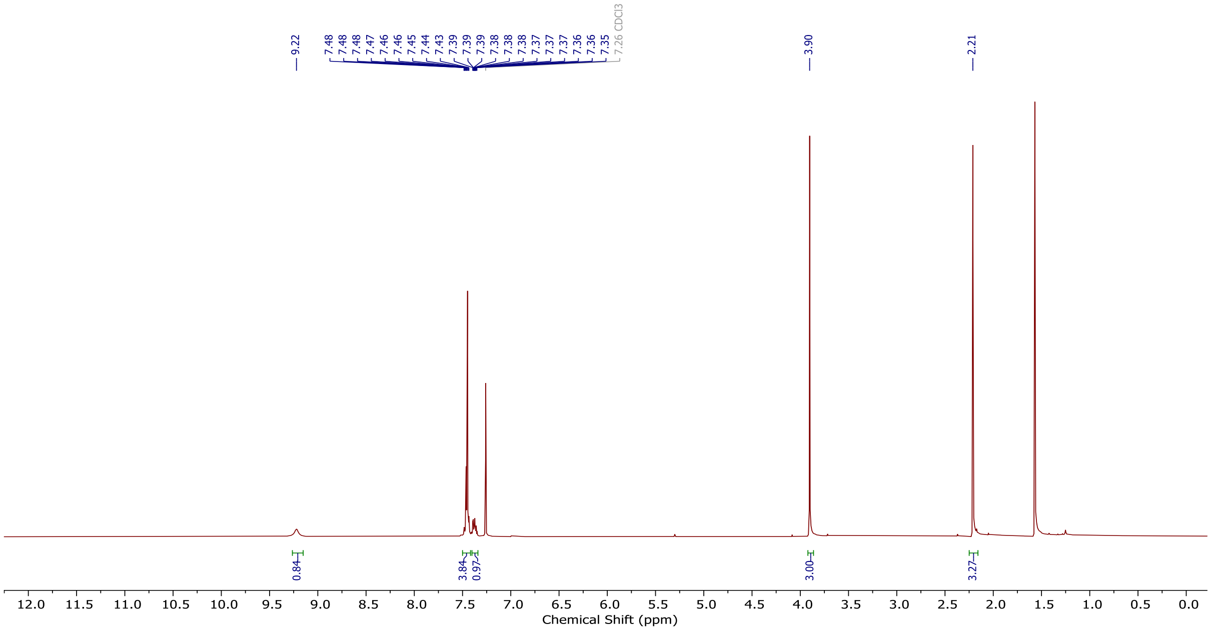

^{13}C NMR (101 MHz, $CDCl_3$)

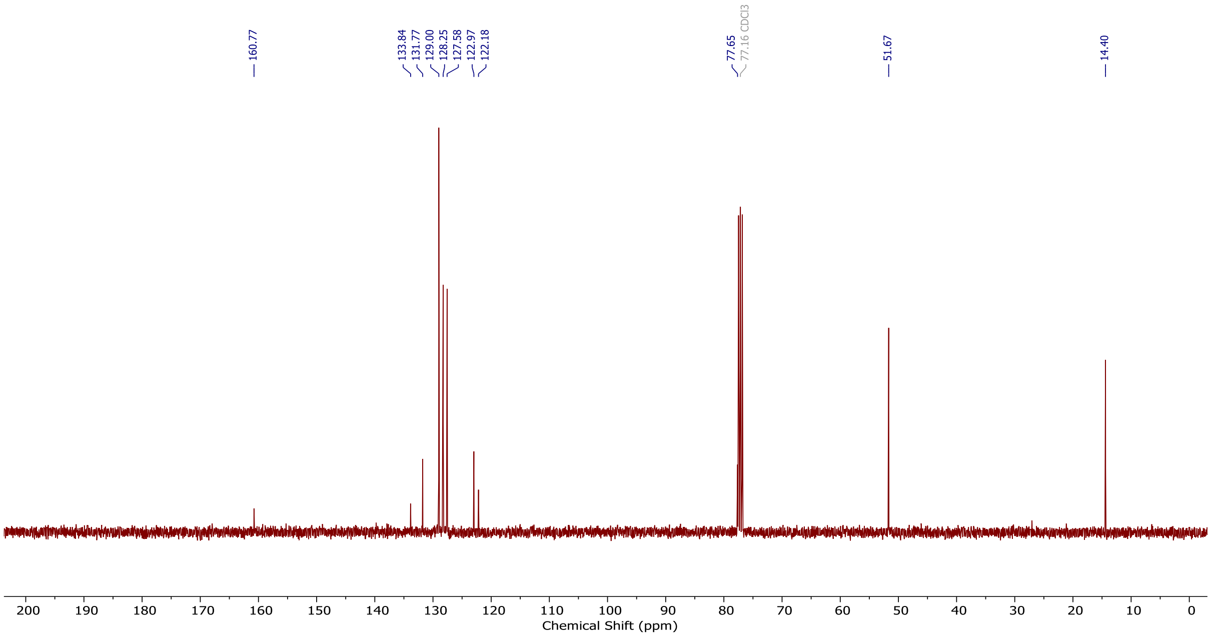

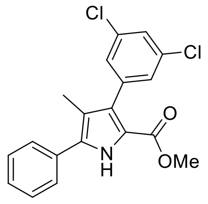

Compound **6a**

^{1}H NMR (600 MHz, DMSO-d_6)

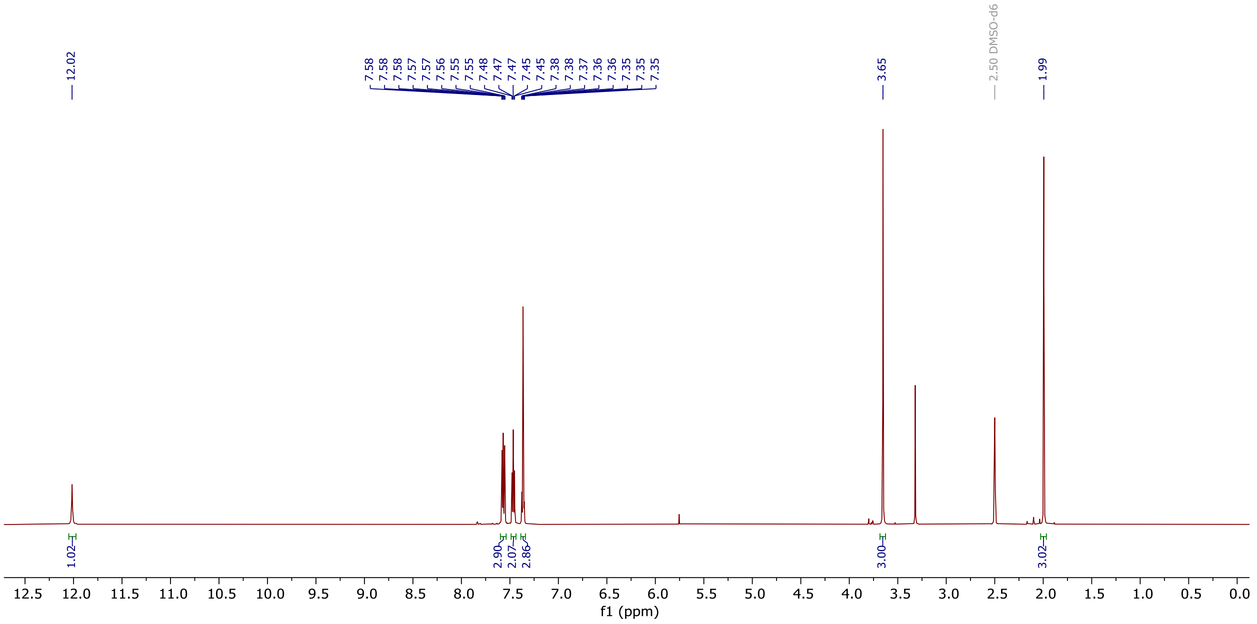

^{13}C NMR (151 MHz, DMSO-d_6)

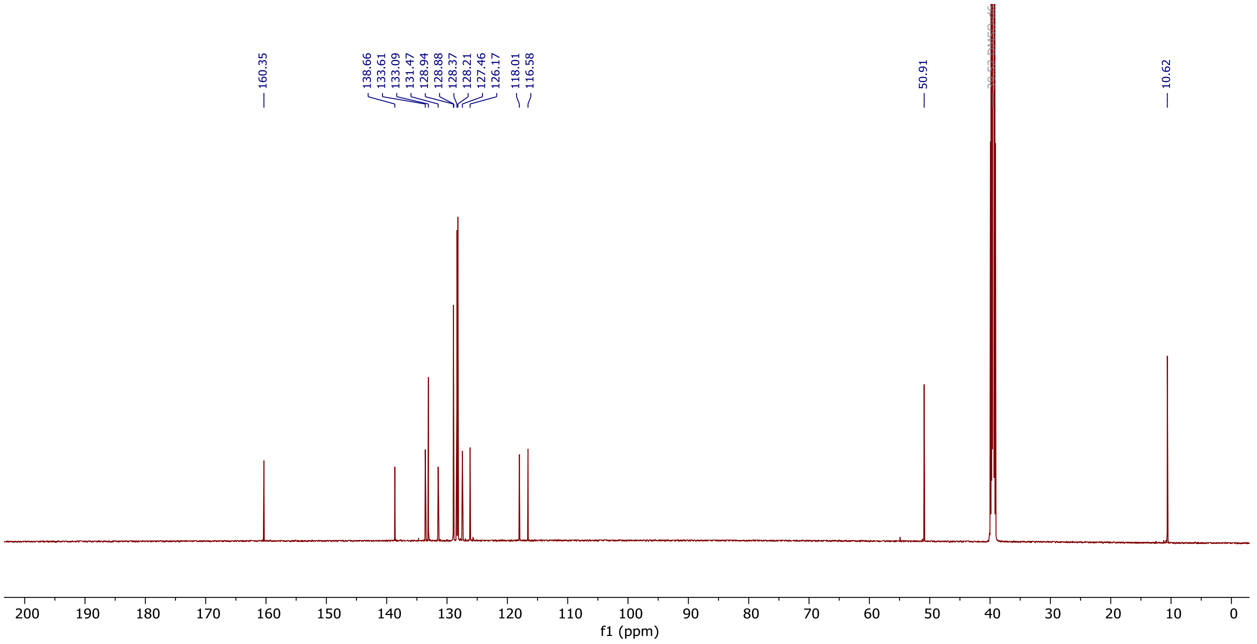

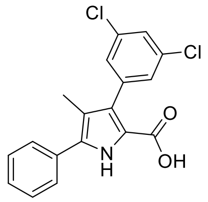

PyC 6

^{1}H NMR (600 MHz, DMSO-d_6)

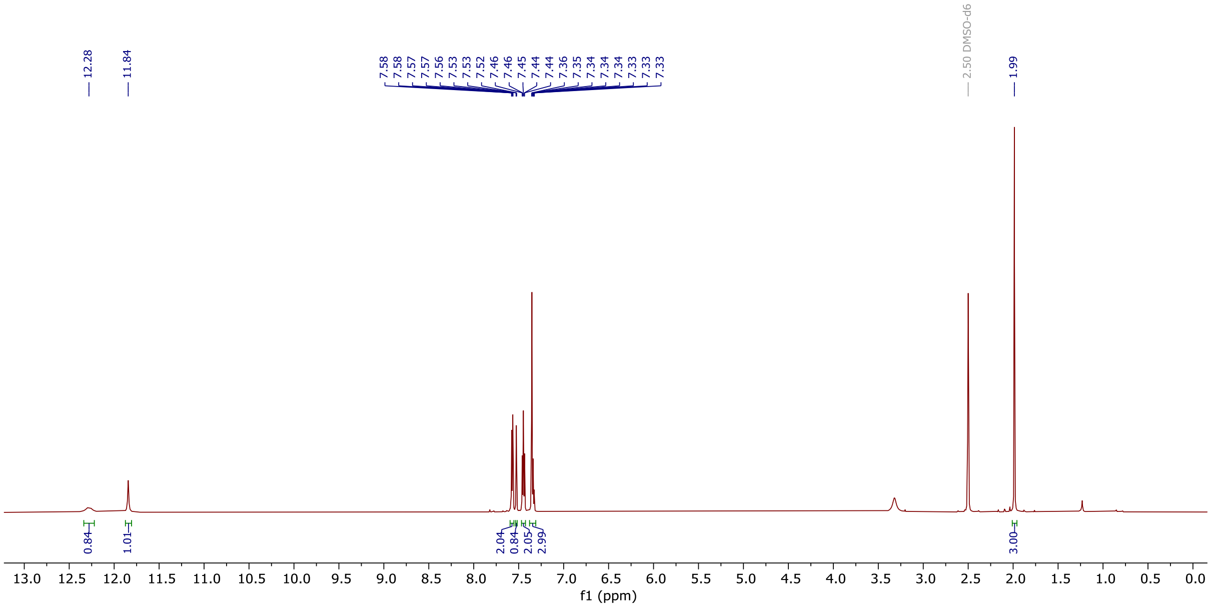

^{13}C NMR (151 MHz, DMSO-d_6)

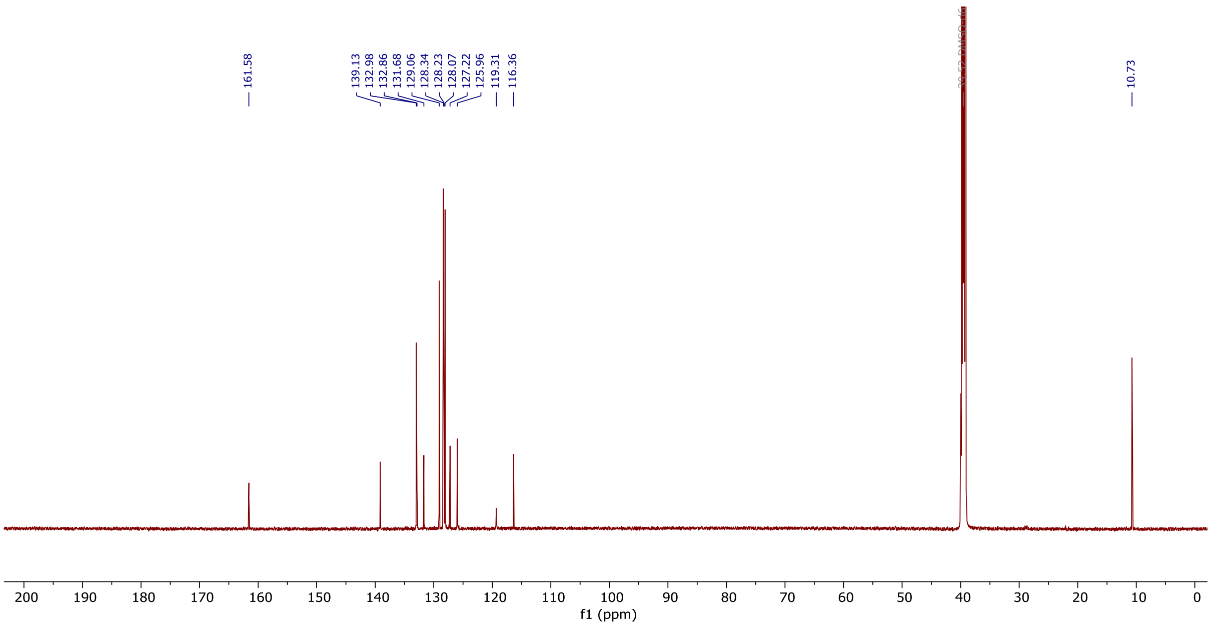

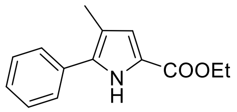

Compound **40b**

^{1}H NMR (400 MHz, $CDCl_3$)

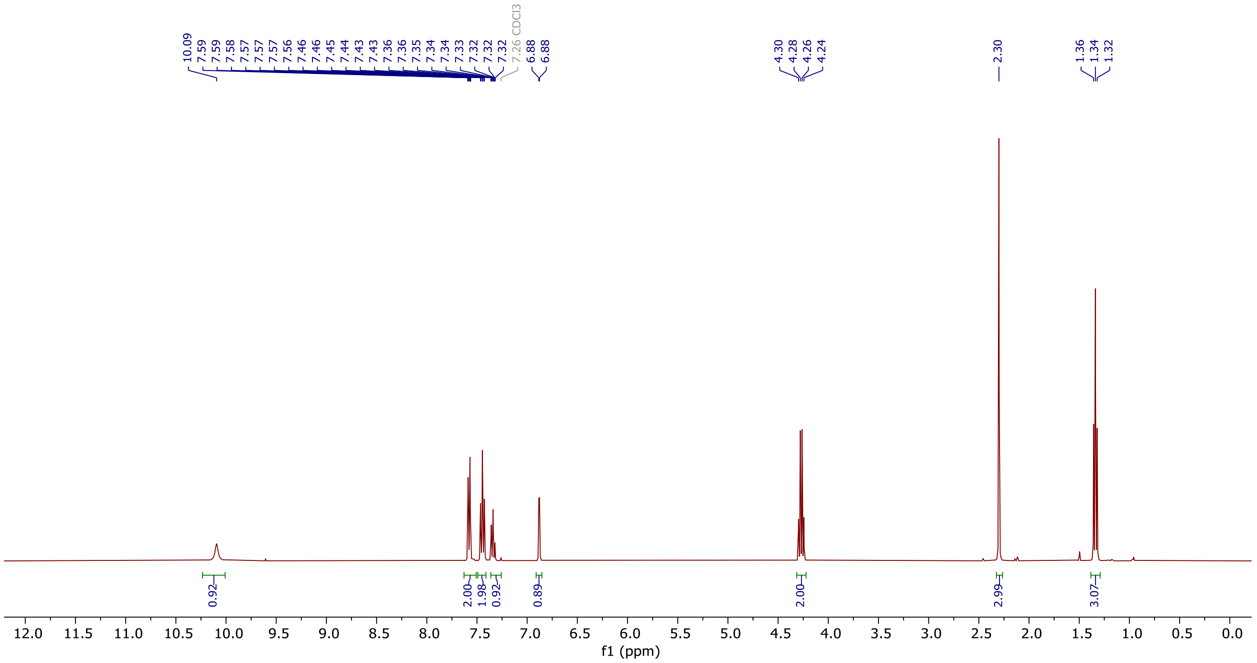

^{13}C NMR (151 MHz, $CDCl_3$)

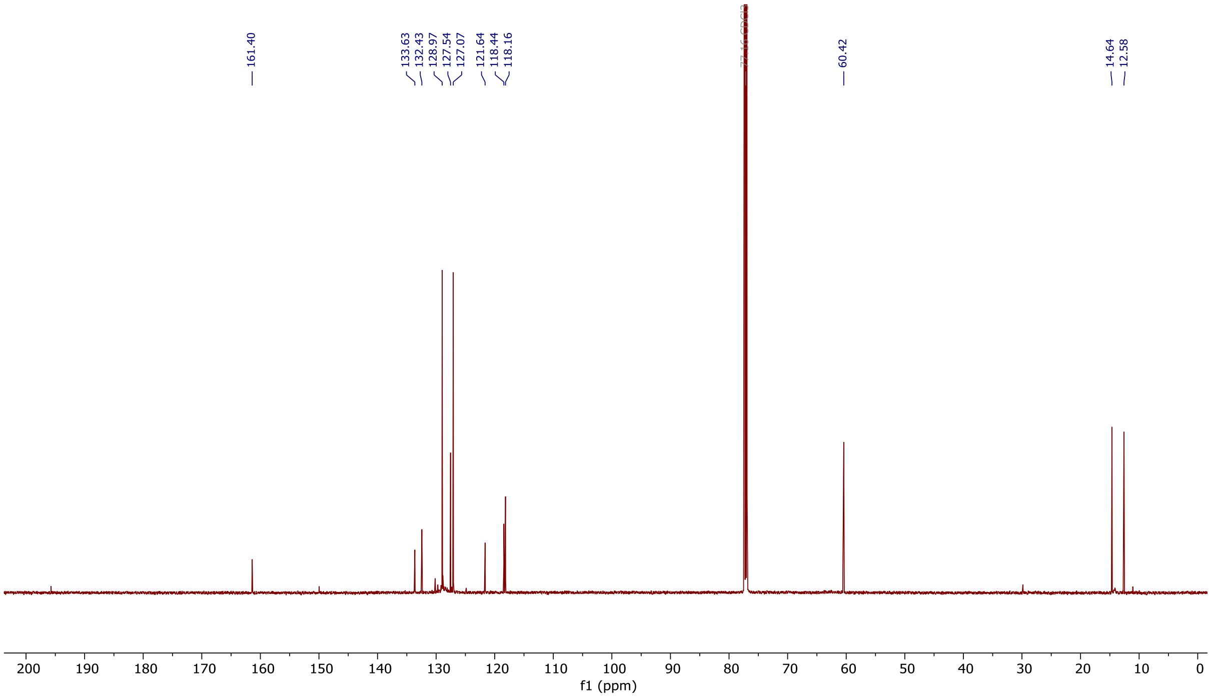

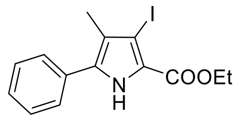

Compound **41b**

^{1}H NMR (400 MHz, $CDCl_3$)

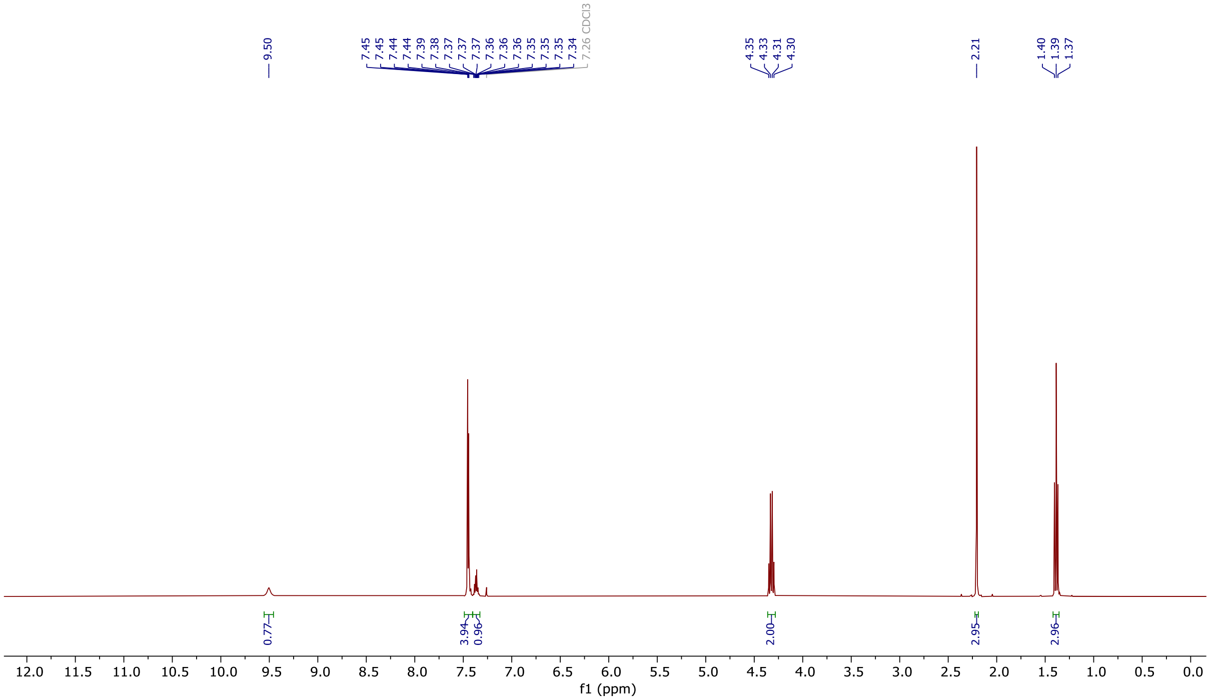

^{13}C NMR (101 MHz, $CDCl_3$)

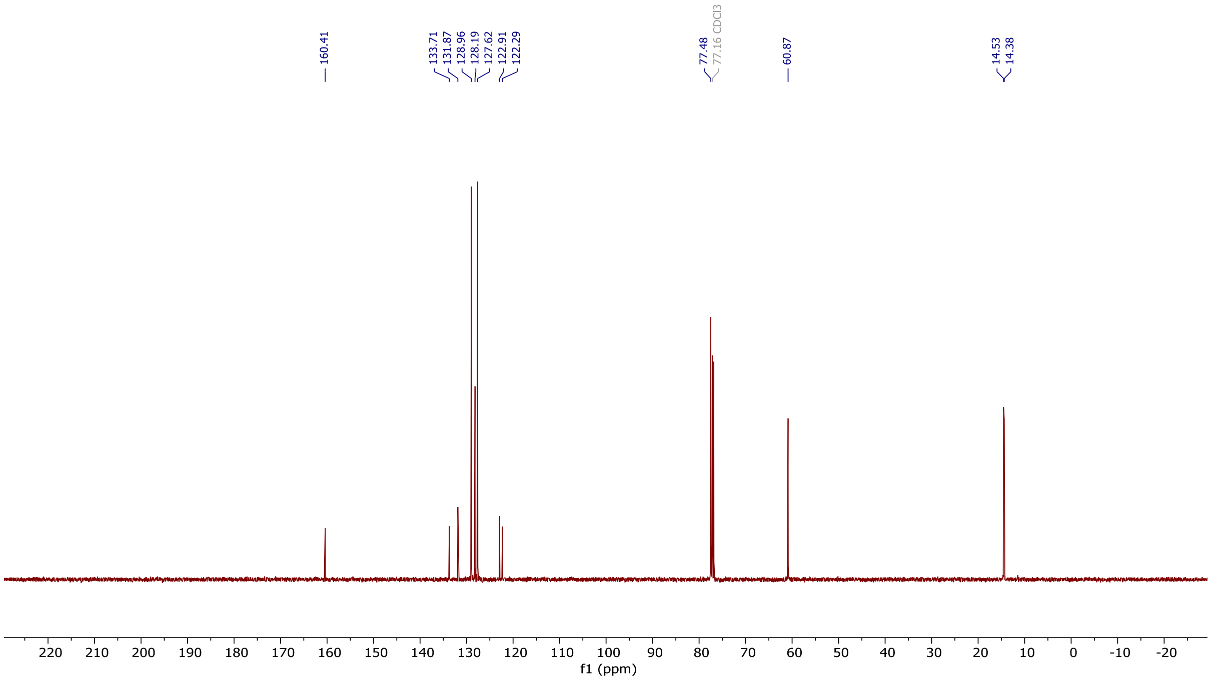

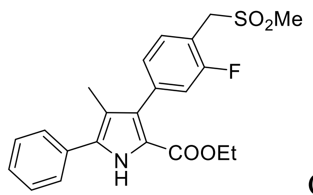

Compound **7a**

^{1}H NMR (700 MHz, DMSO-d_6)

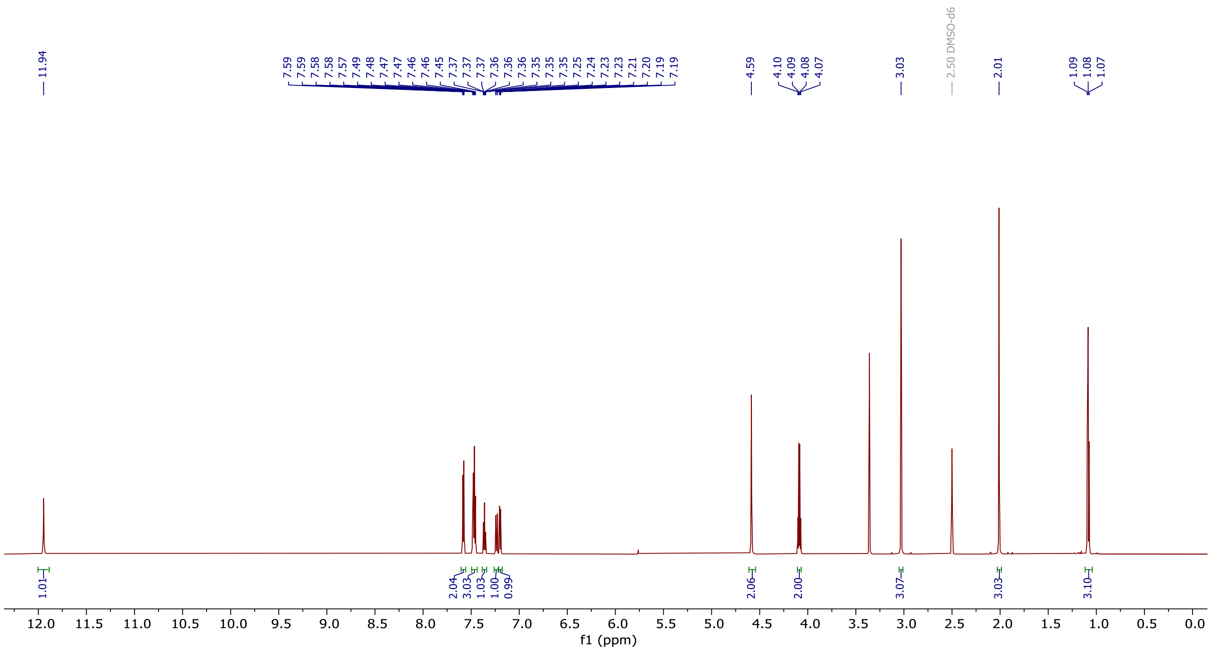

^{13}C NMR (176 MHz, DMSO-d_6)

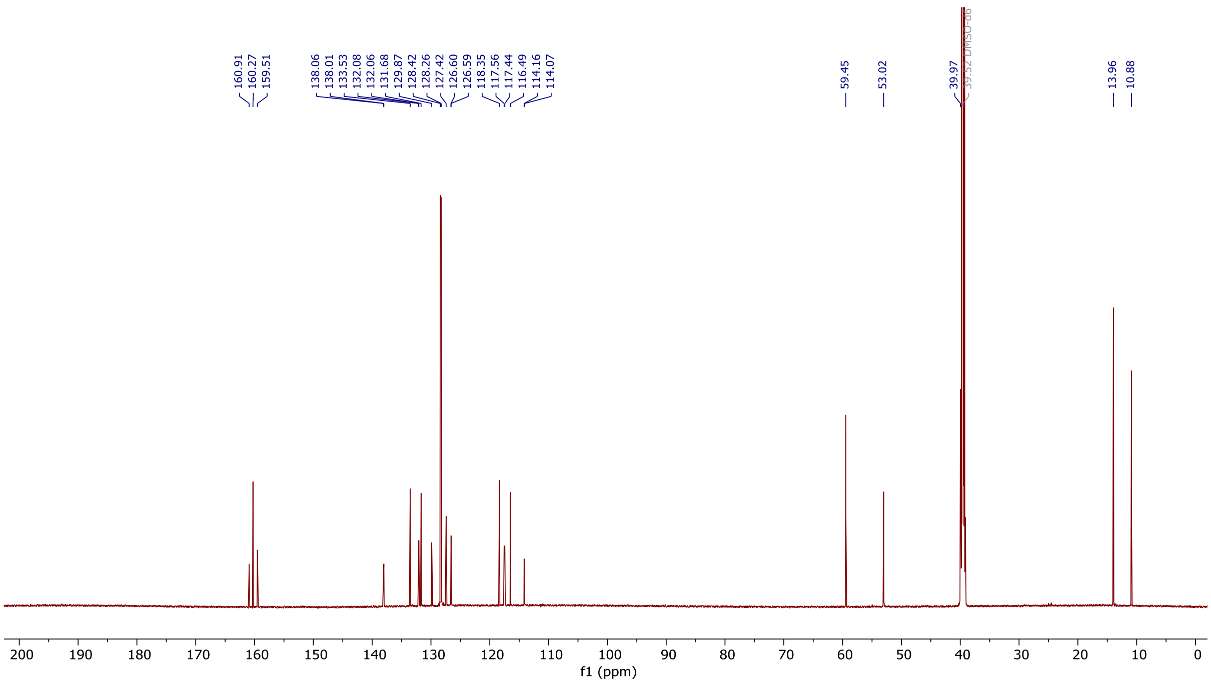

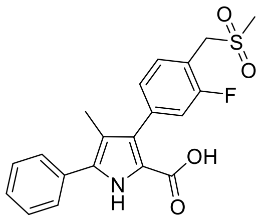

PyC 7

^{1}H NMR (600 MHz, DMSO-d_6)

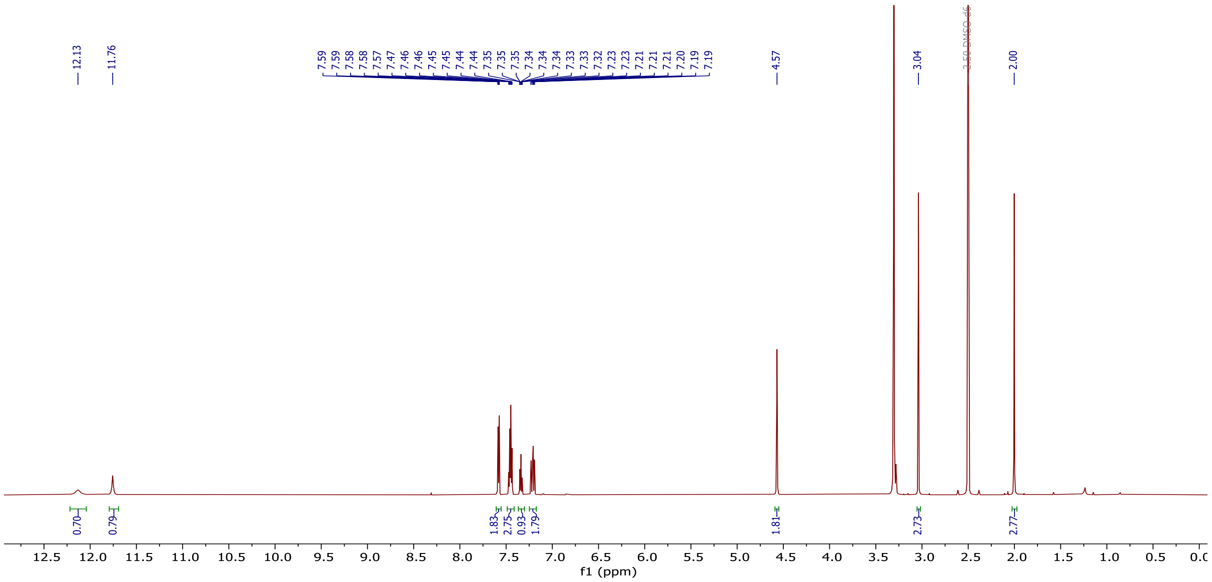

^{13}C NMR (151 MHz, DMSO-d_6)

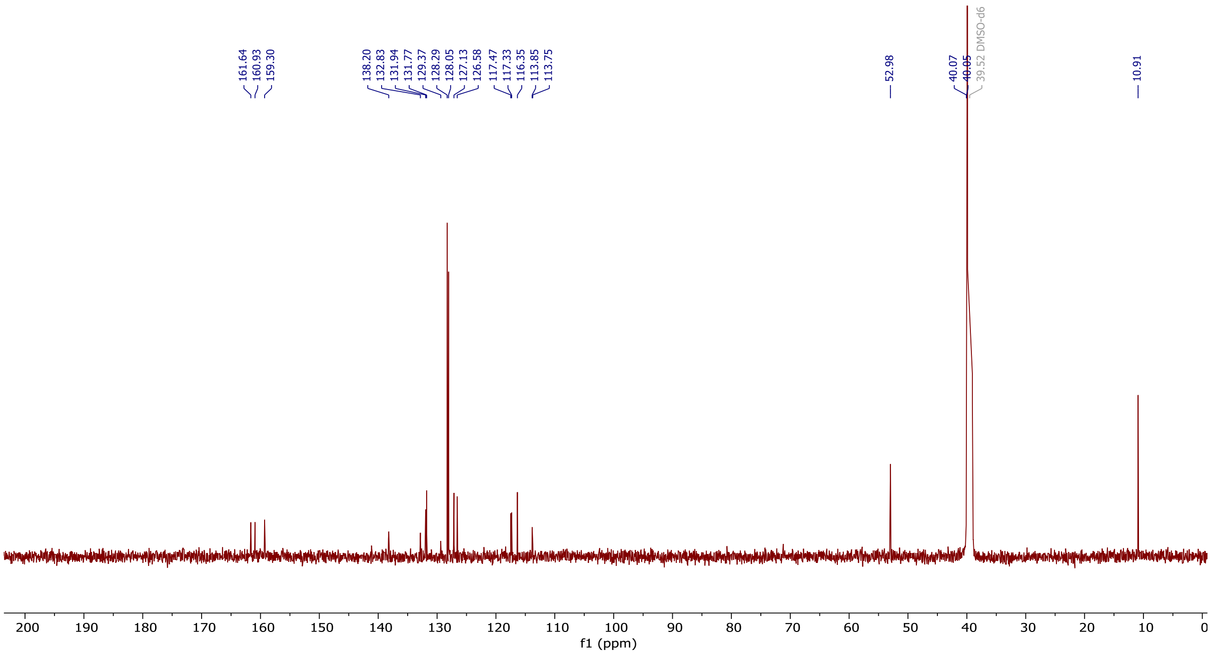

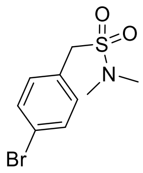

Compound **42c′**

^{1}H NMR (400 MHz, $CDCl_3$)

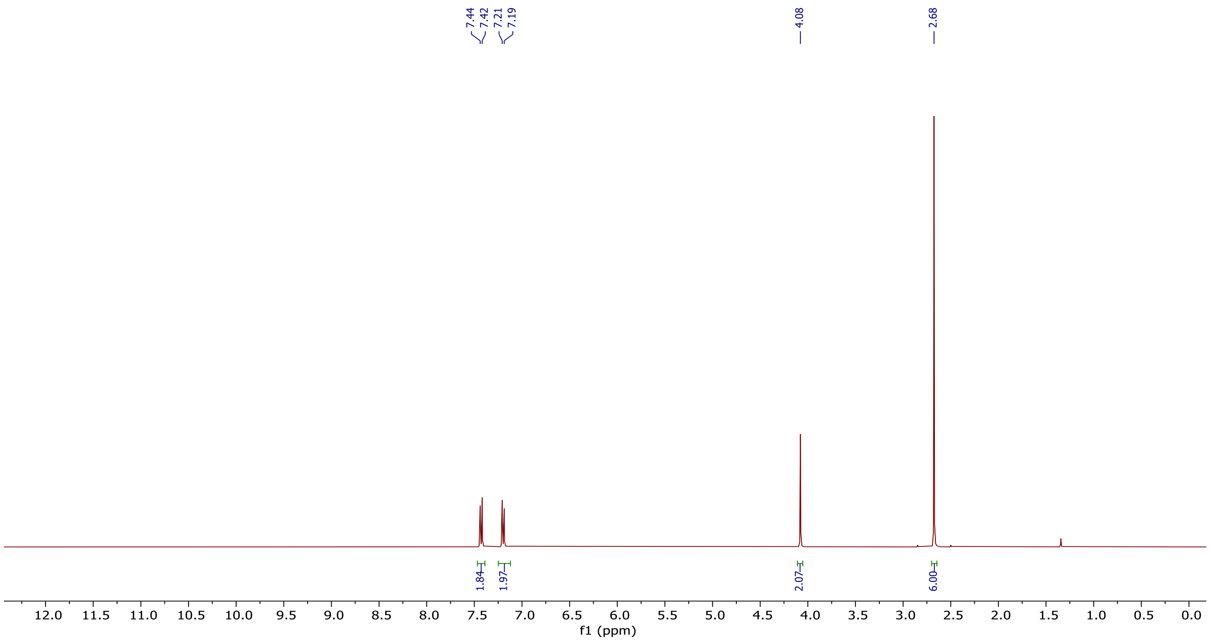

^{13}C NMR (101 MHz, $CDCl_3$)

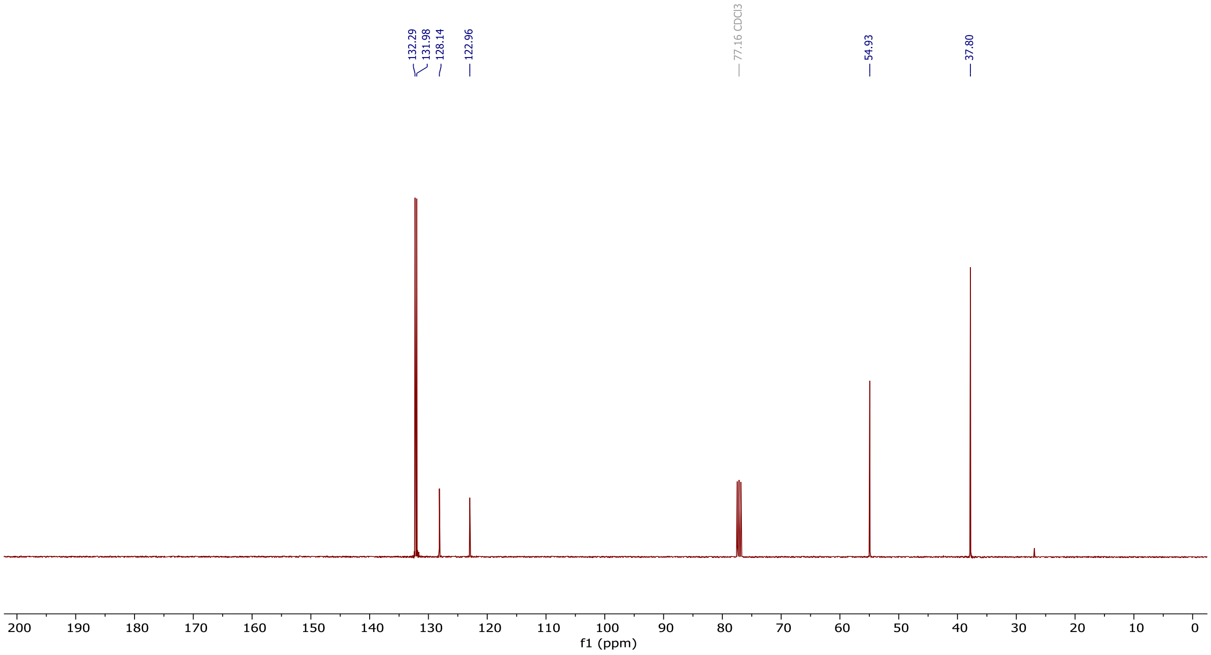

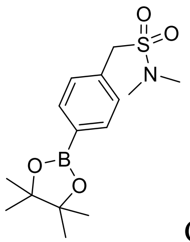

Compound **42c**

^{1}H NMR (600 MHz, $CDCl_3$)

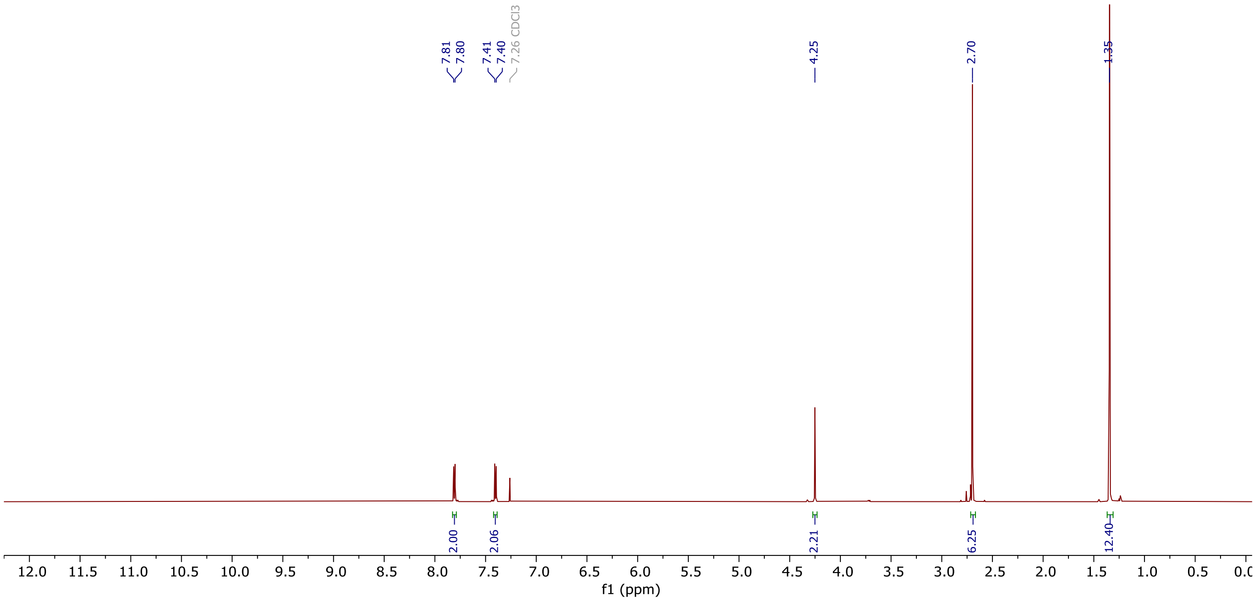

^{13}C NMR (151 MHz, $CDCl_3$)

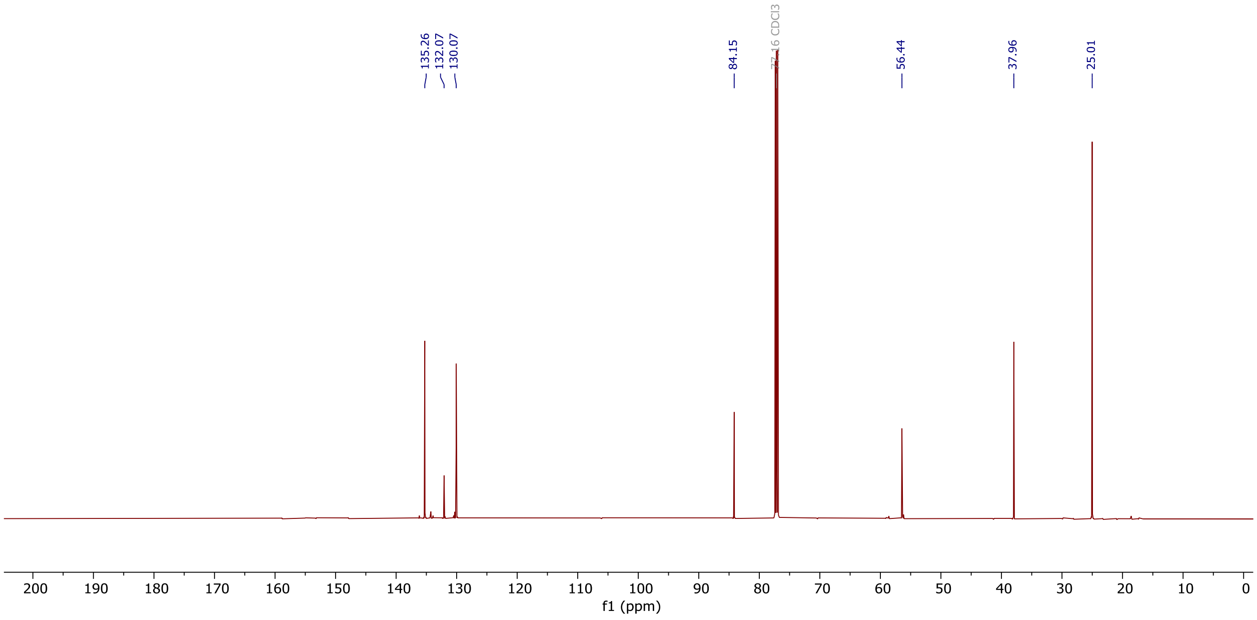

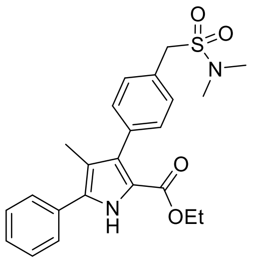

Compound **8a**

^{1}H NMR (500 MHz, DMSO-d_6)

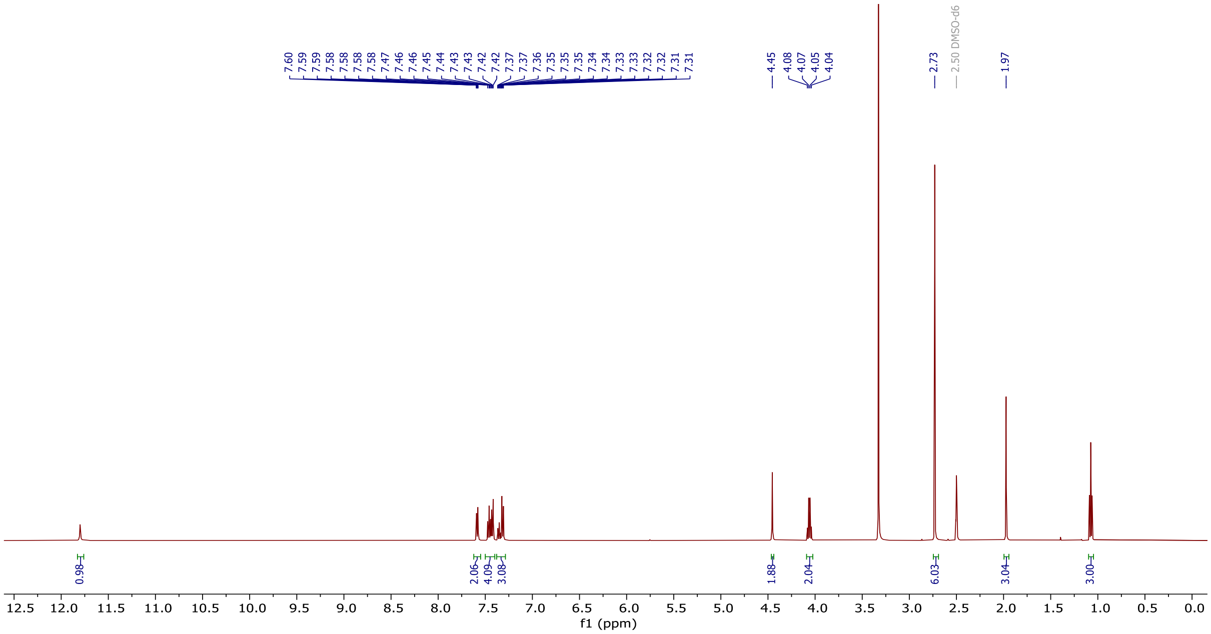

^{13}C NMR (126 MHz, DMSO-d_6)

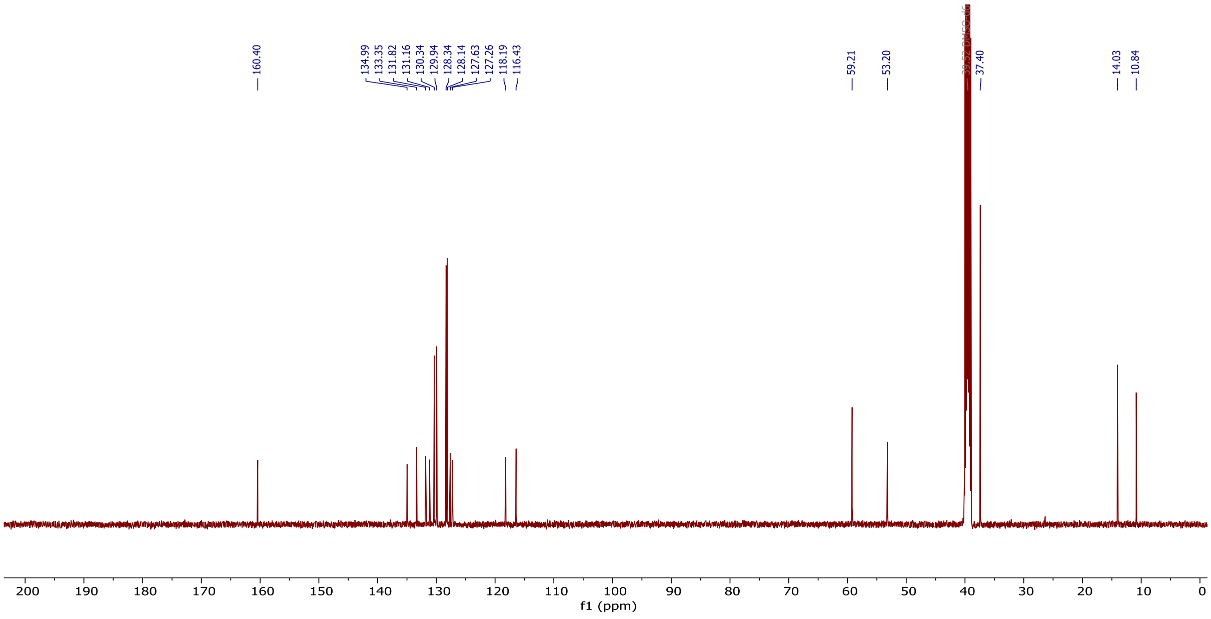

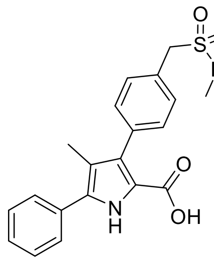

PyC 8

^{1}H NMR (600 MHz, DMSO-d_6)

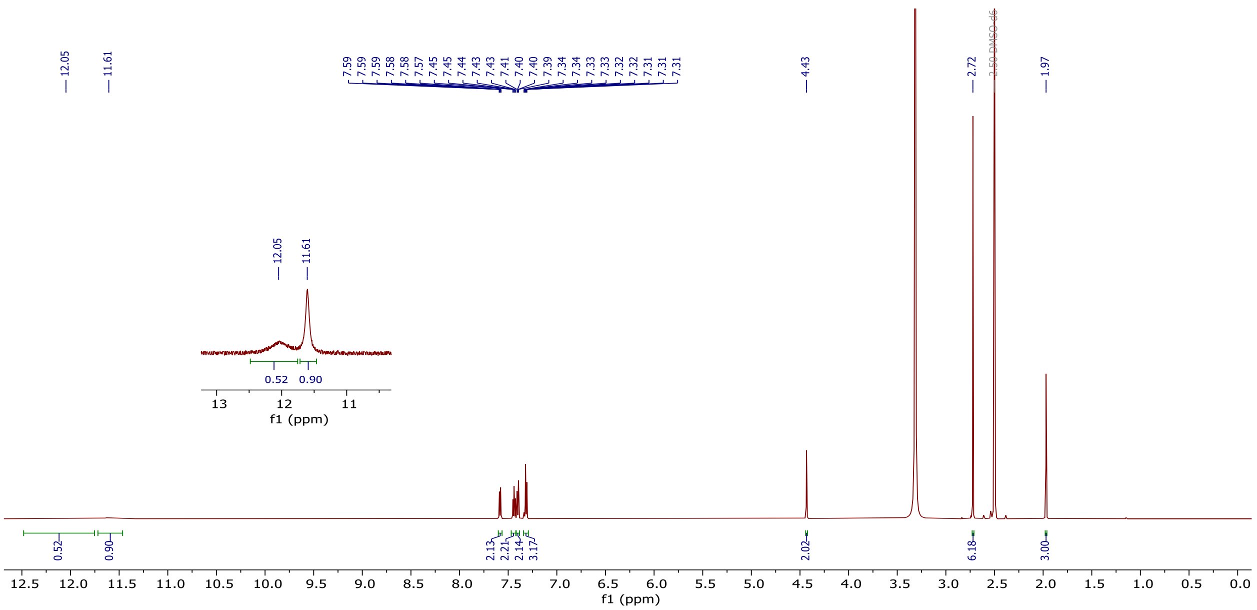

^{13}C NMR (151 MHz, DMSO-d_6)

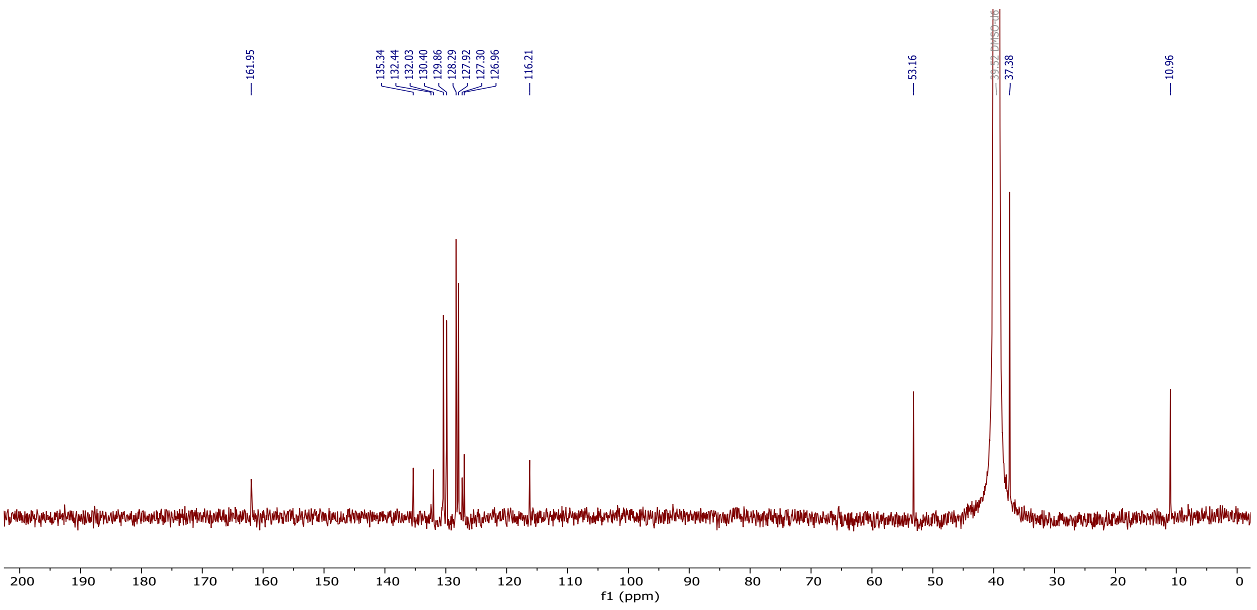

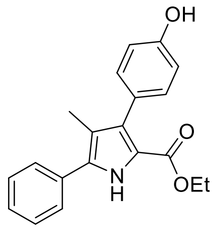

Compound **9a**

^{1}H NMR (500 MHz, DMSO-d_6)

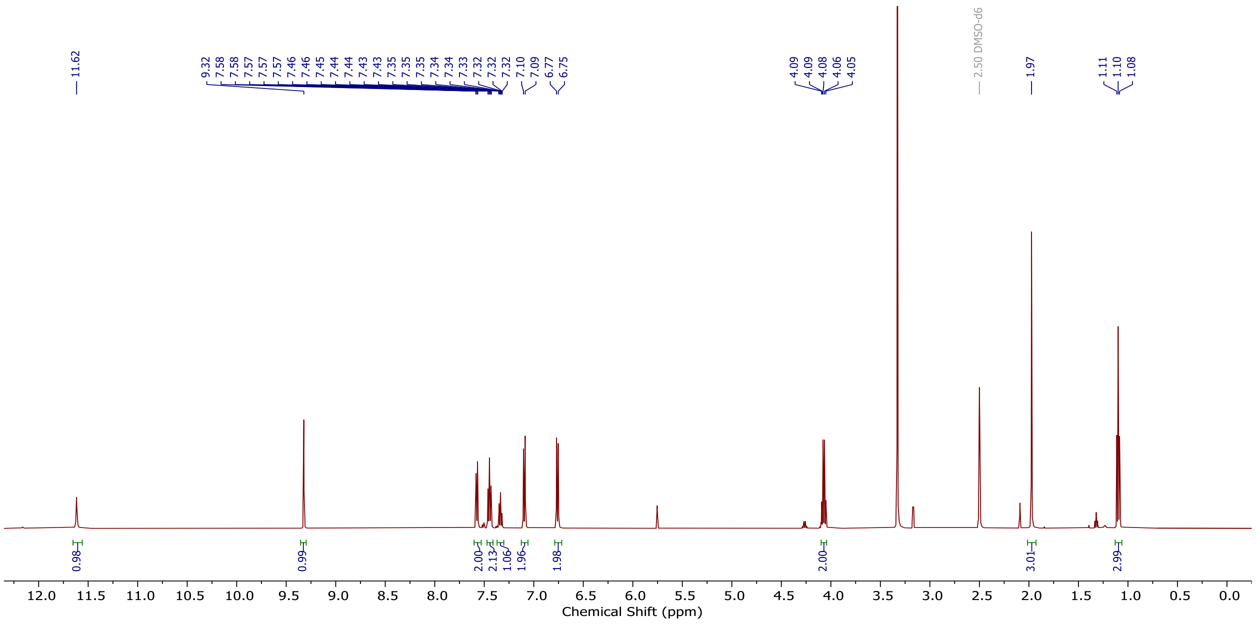

^{13}C NMR (126 MHz, DMSO-d_6)

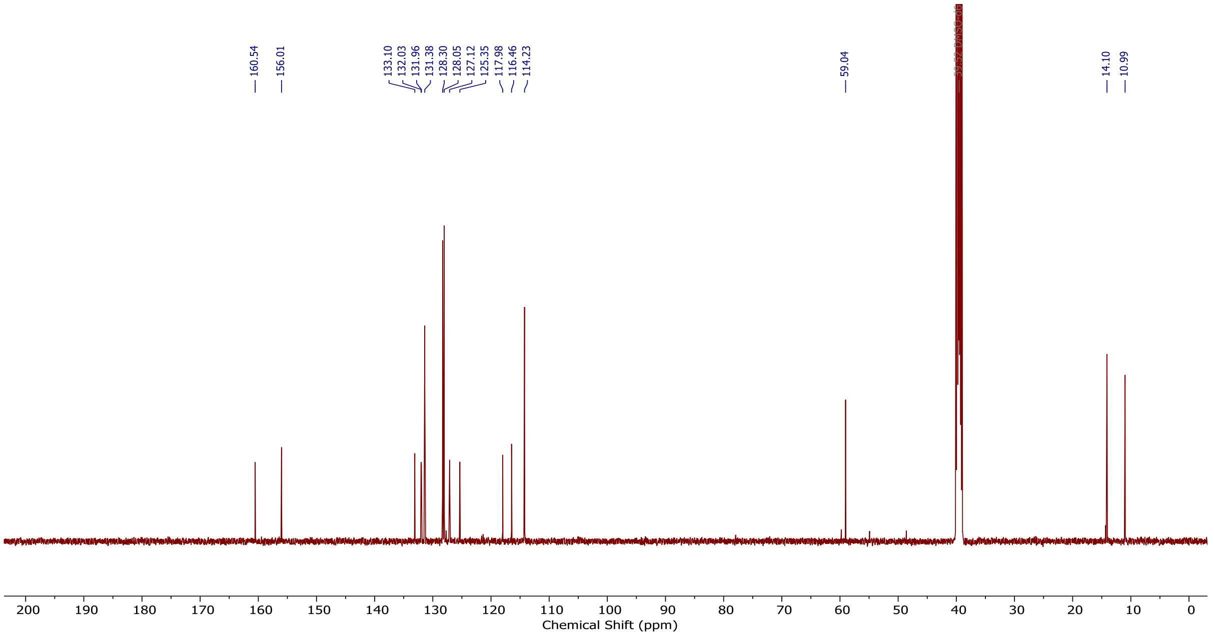

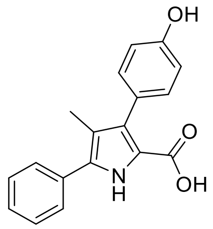

PyC 9

^{1}H NMR (600 MHz, DMSO-d_6)

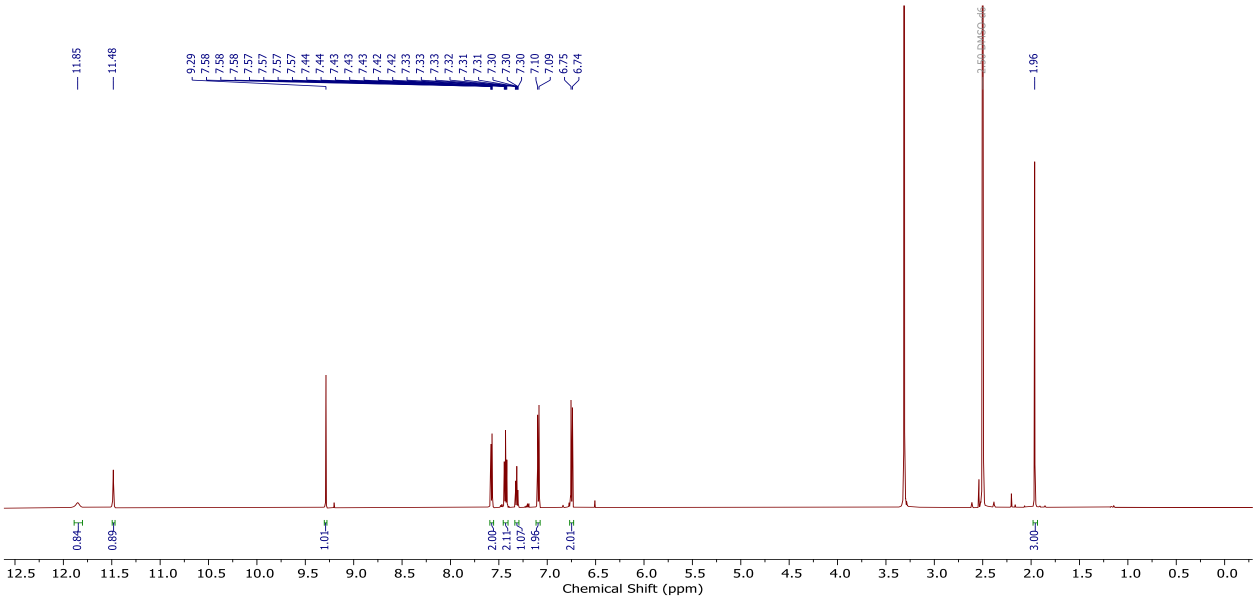

^{13}C NMR (151 MHz, DMSO-d_6)

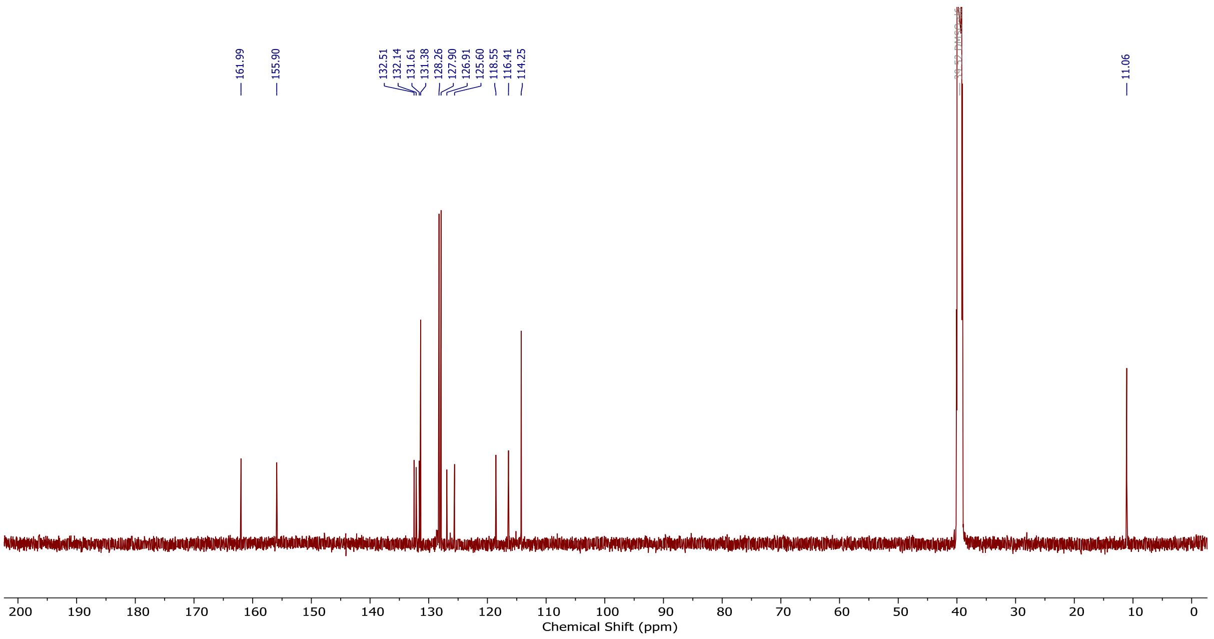

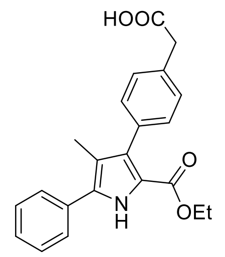

Compound **11a**

^{1}H NMR (500 MHz, DMSO-d_6)

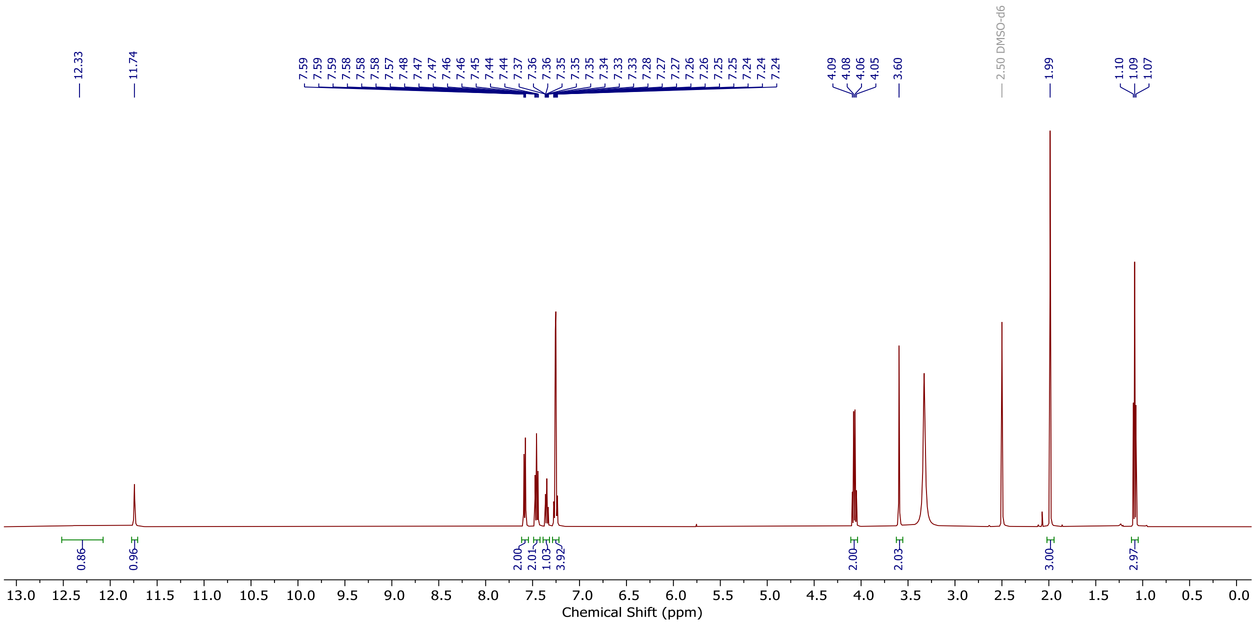

^{13}C NMR (126 MHz, DMSO-d_6)

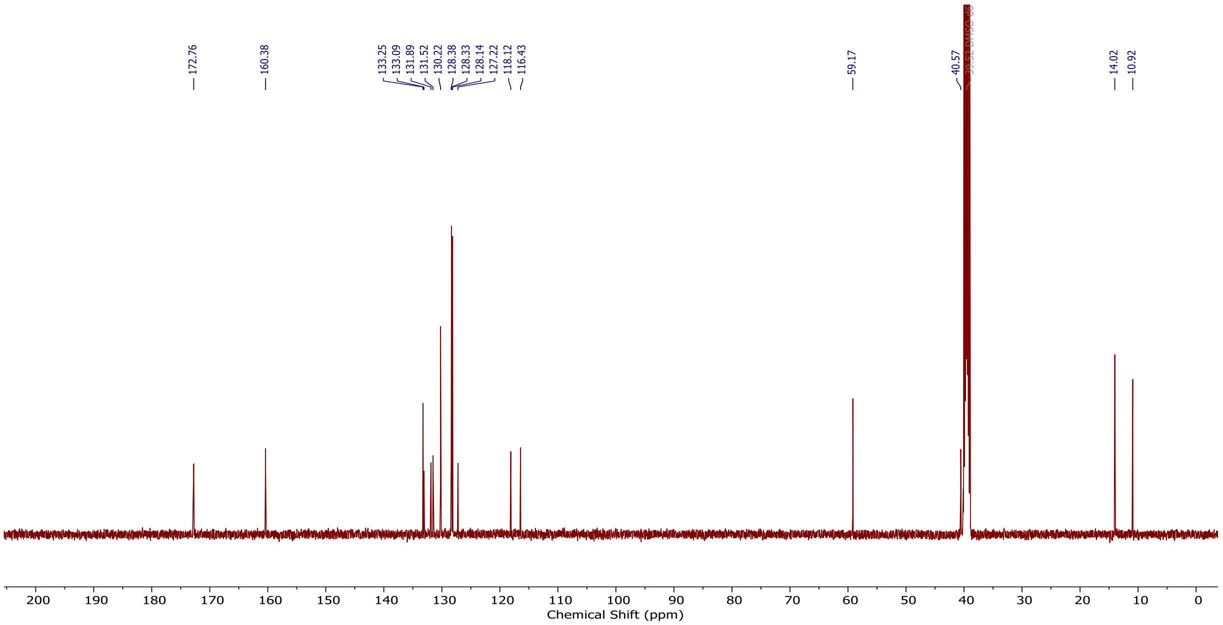

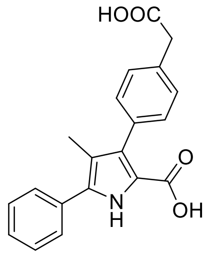

PyC 11

^{1}H NMR (600 MHz, DMSO-d_6)

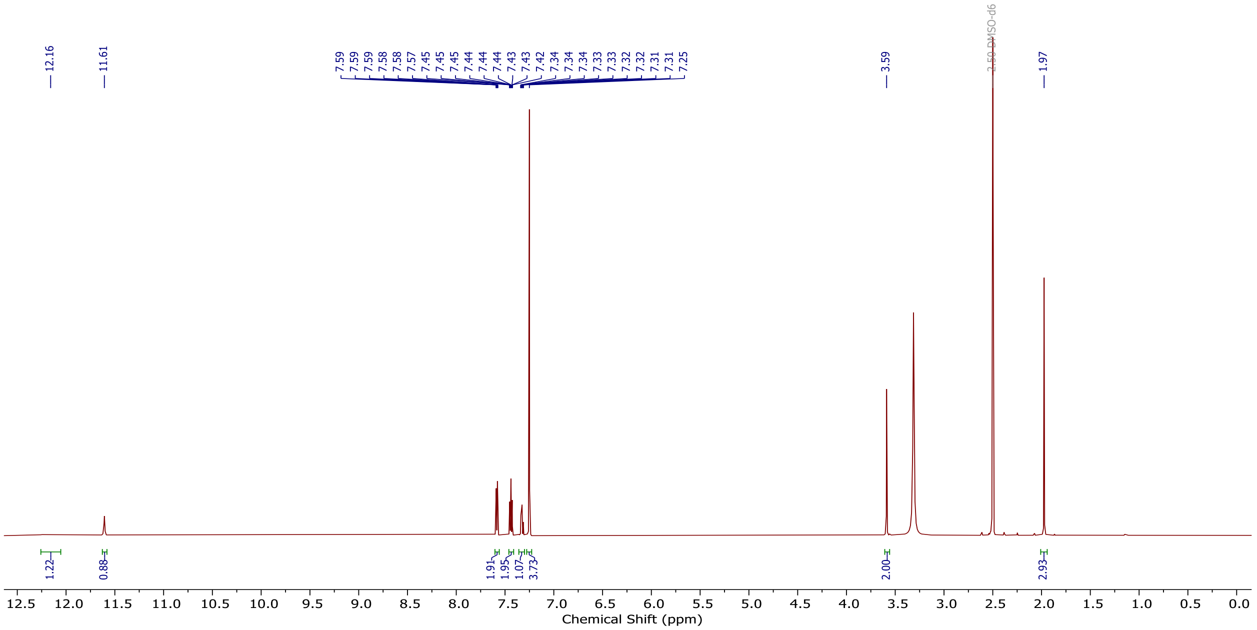

^{13}C NMR (151 MHz, DMSO-d_6)

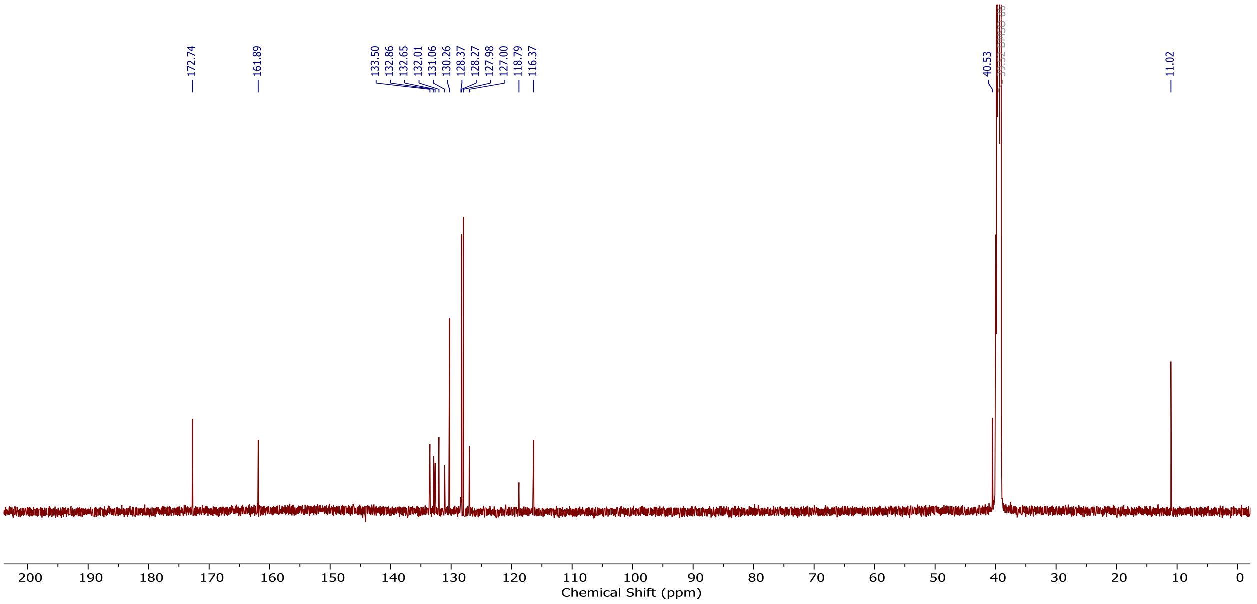

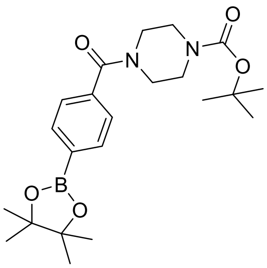

Compound **42f**

^{1}H NMR (600 MHz, $CDCl_3$)

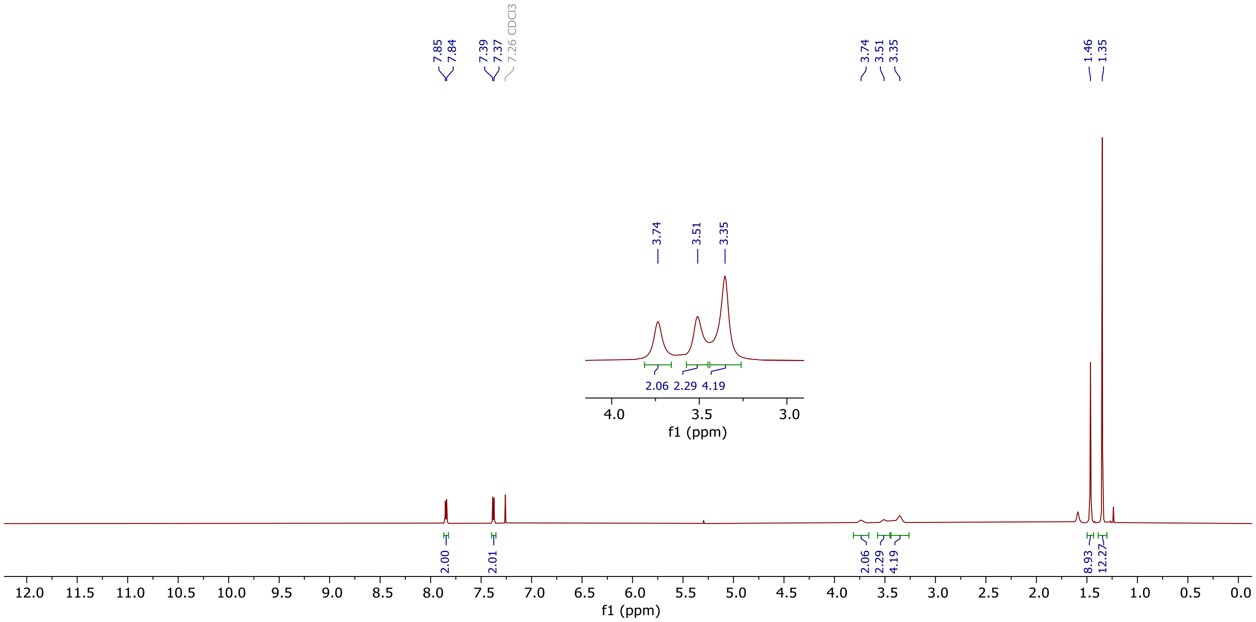

^{13}C NMR (151 MHz, $CDCl_3$)

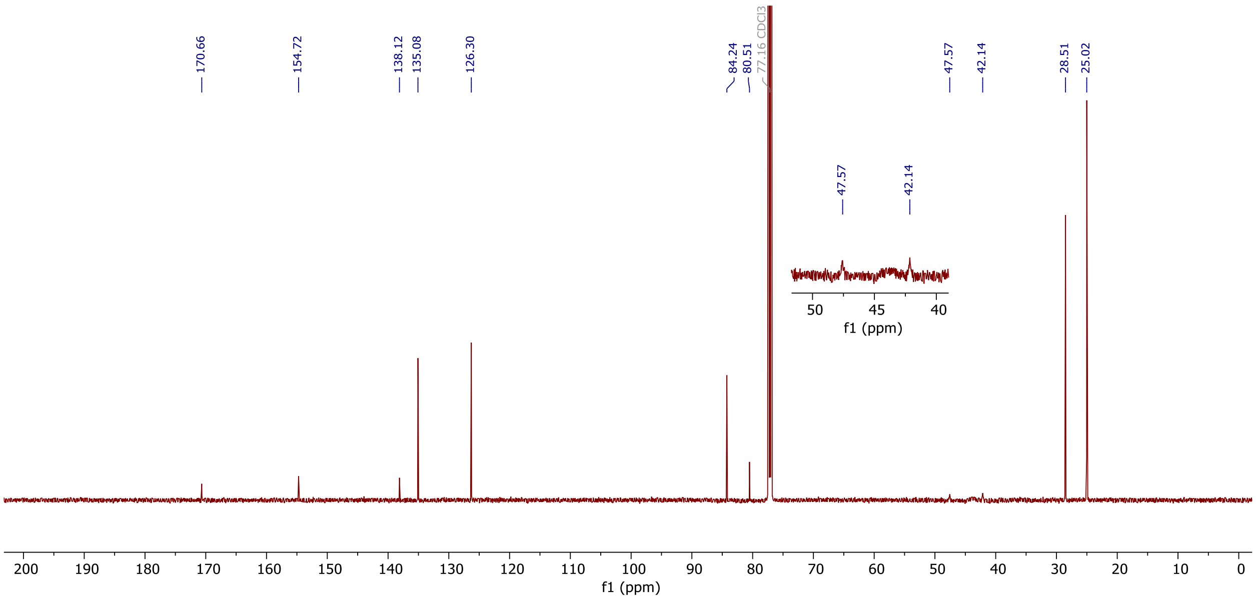

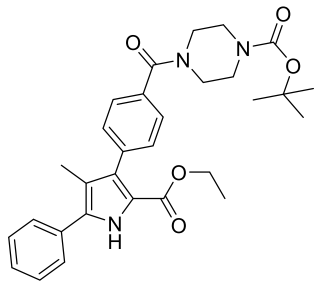

Compound **12a**

^{1}H NMR (600 MHz, $CDCl_3$)

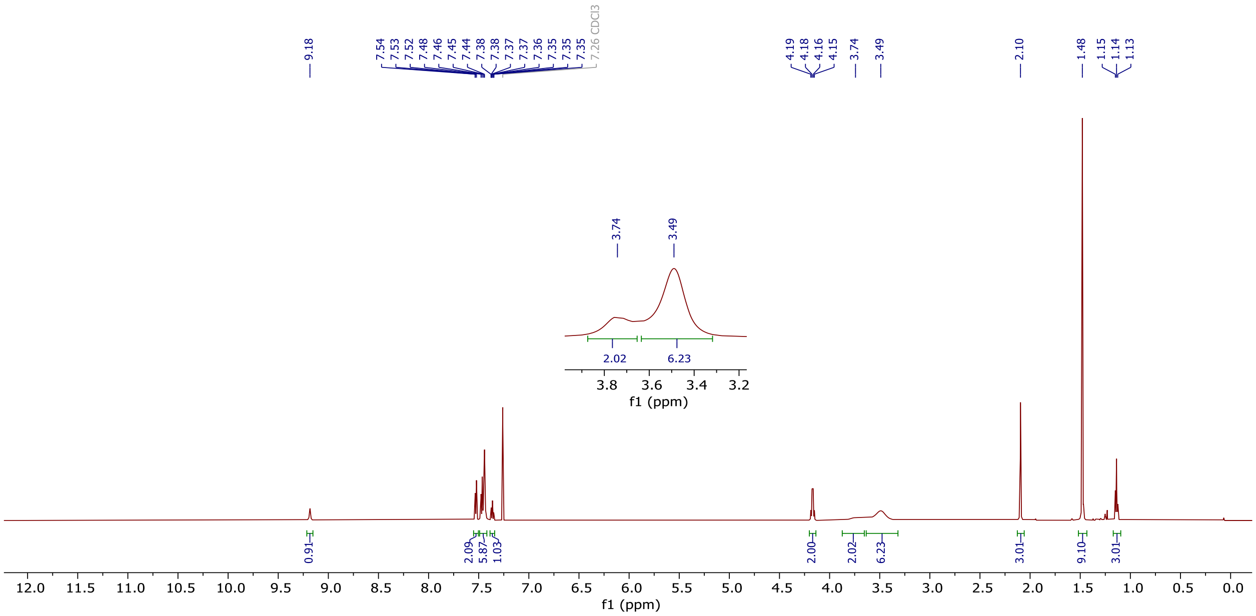

^{13}C NMR (151 MHz, $CDCl_3$)

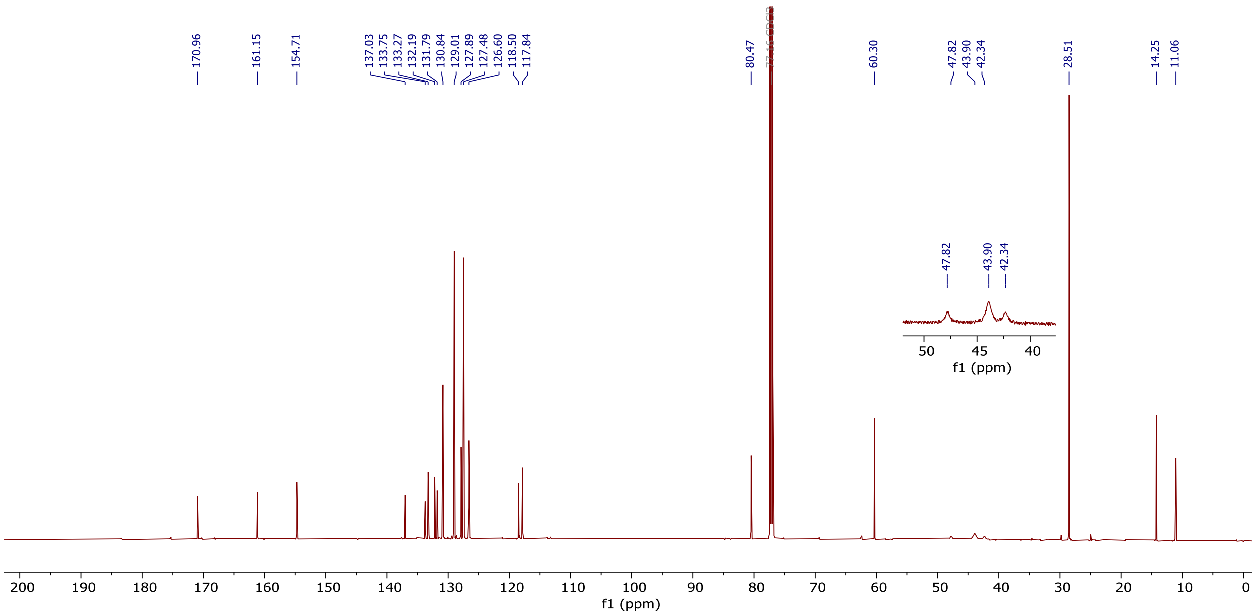

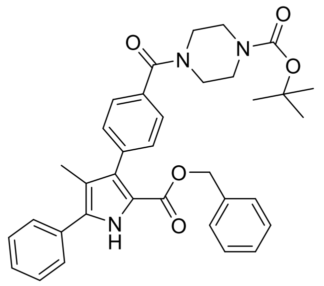

Compound **12b**

^{1}H NMR (600 MHz, DMSO-d_6)

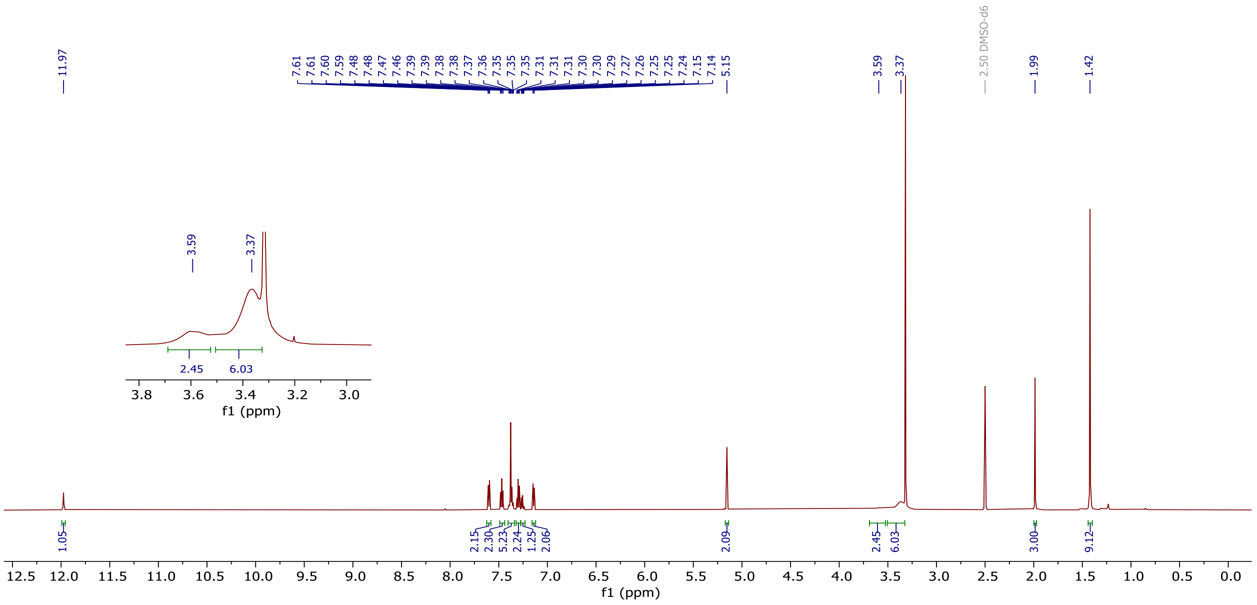

^{13}C NMR (151 MHz, DMSO-d_6)

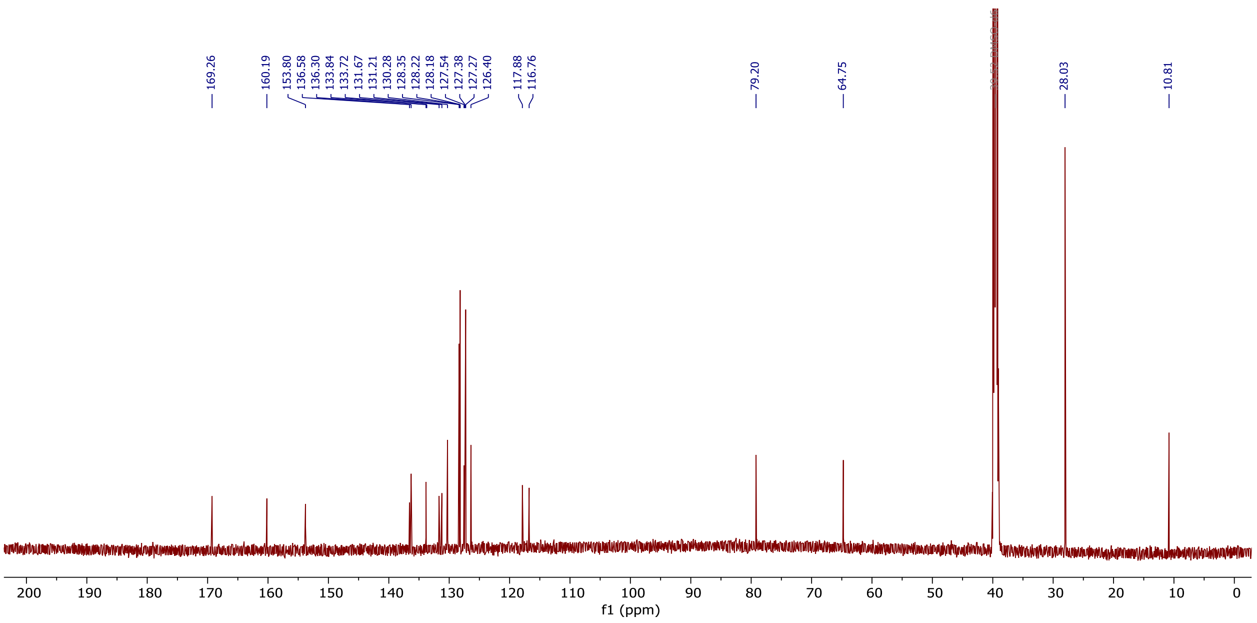

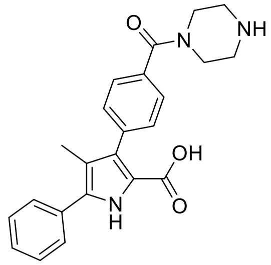

PyC 12

^{1}H NMR (600 MHz, DMSO-d_6, 373 K)

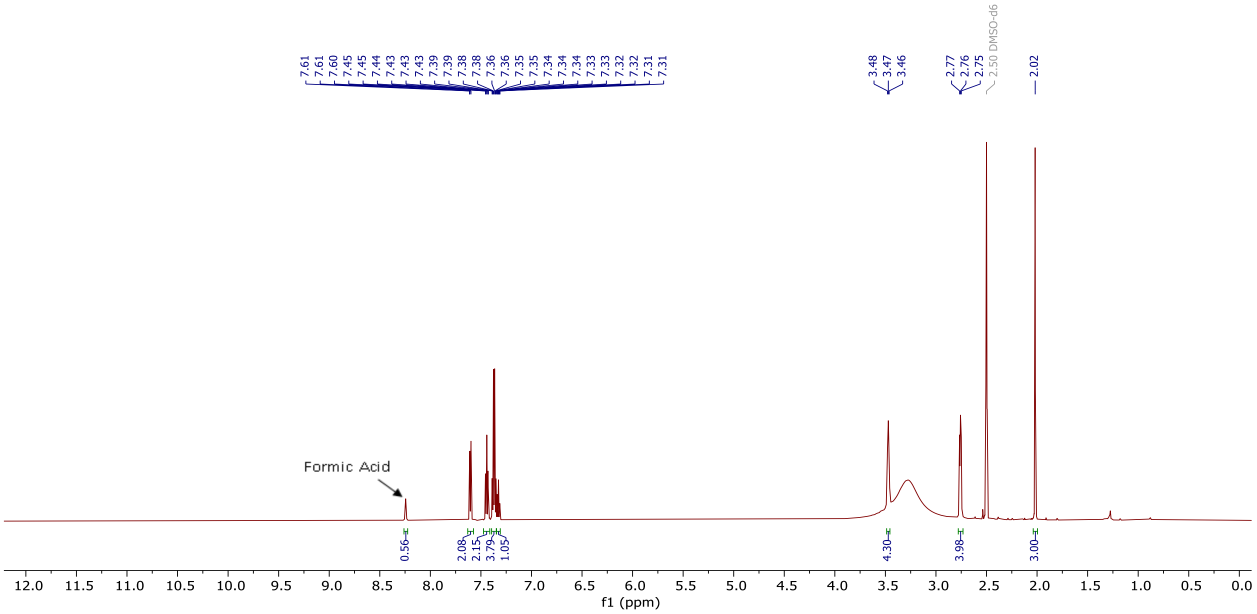

^{13}C NMR (151 MHz, DMSO-d_6, 373 K)

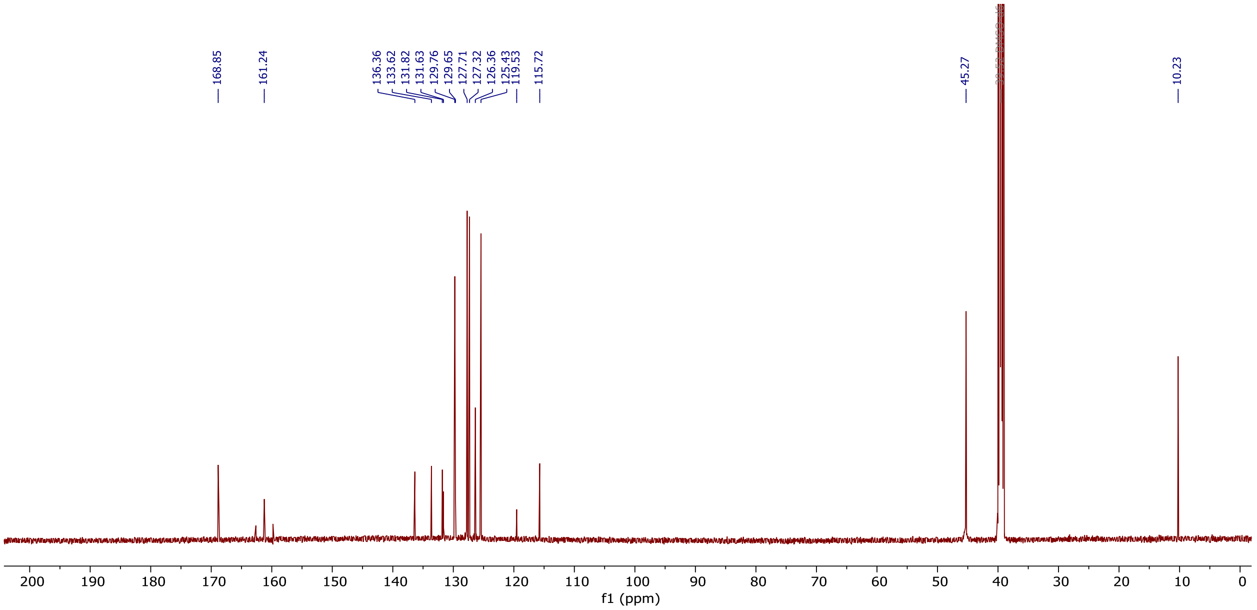

HSQC NMR (373 K)_**PyC 12**

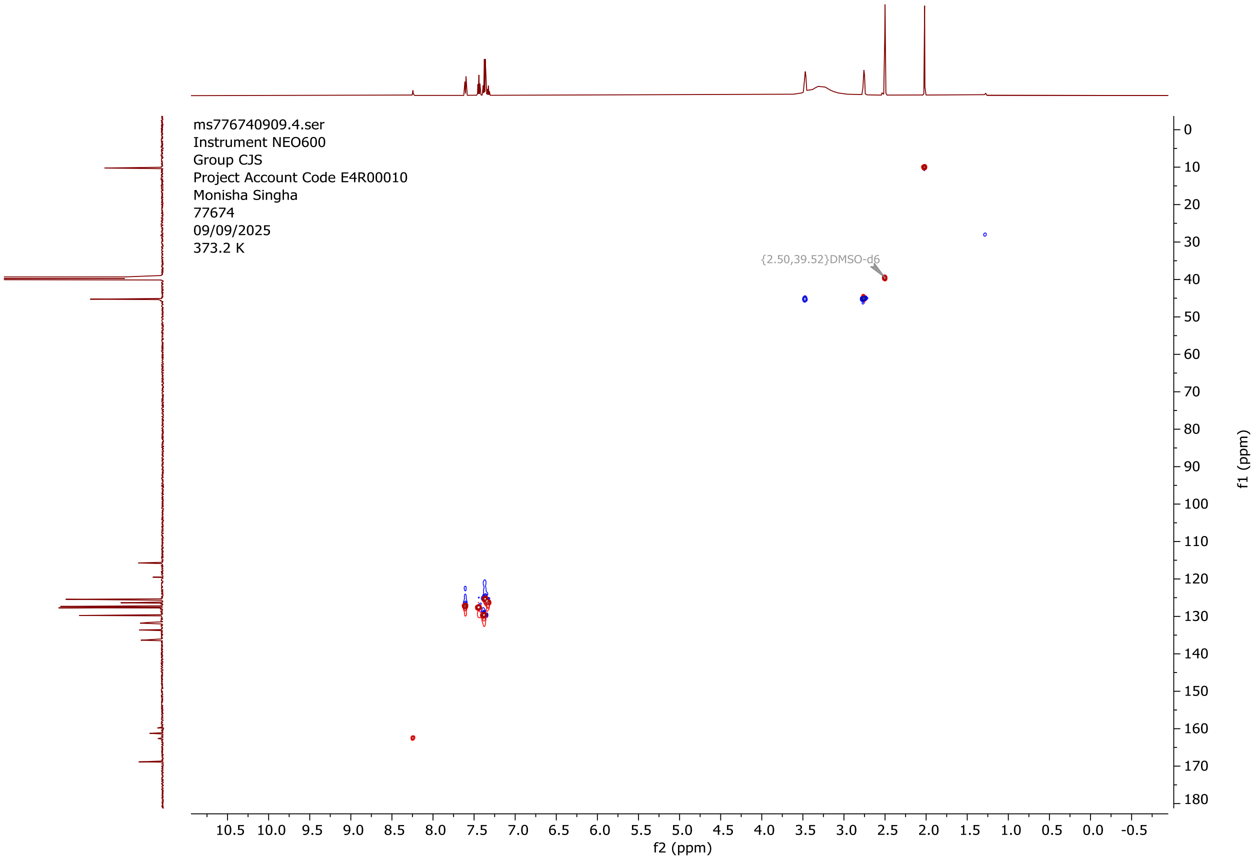

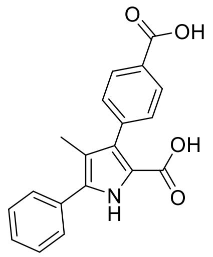

PyC 10

^{1}H NMR (600 MHz, DMSO-d_6)

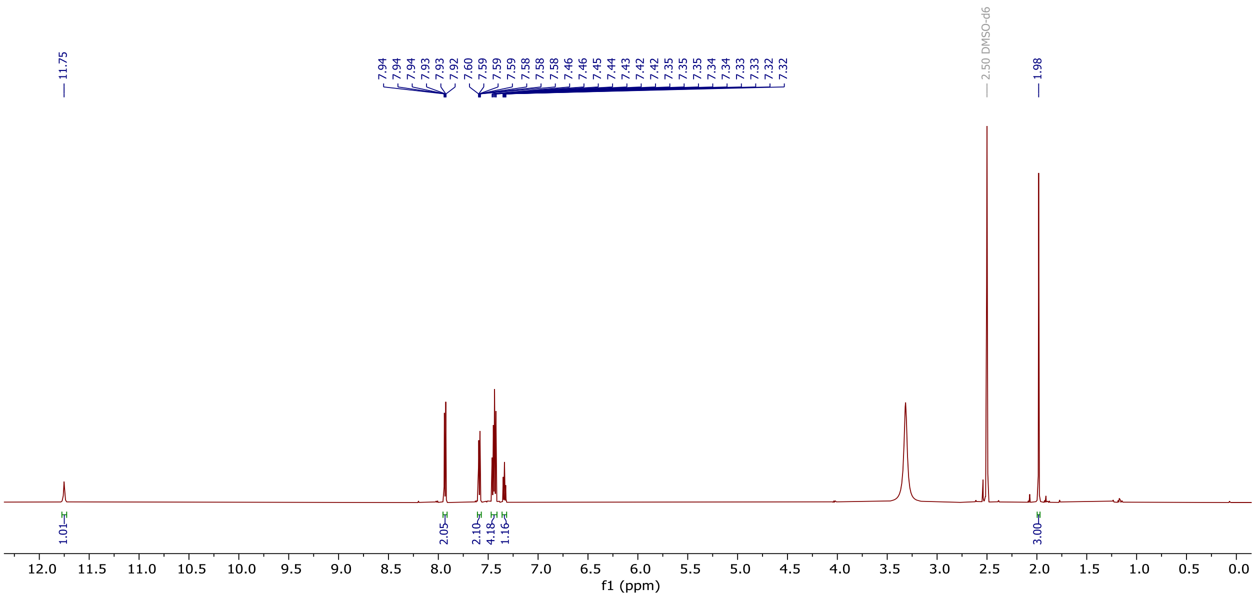

^{13}C NMR (151 MHz, DMSO-d_6)

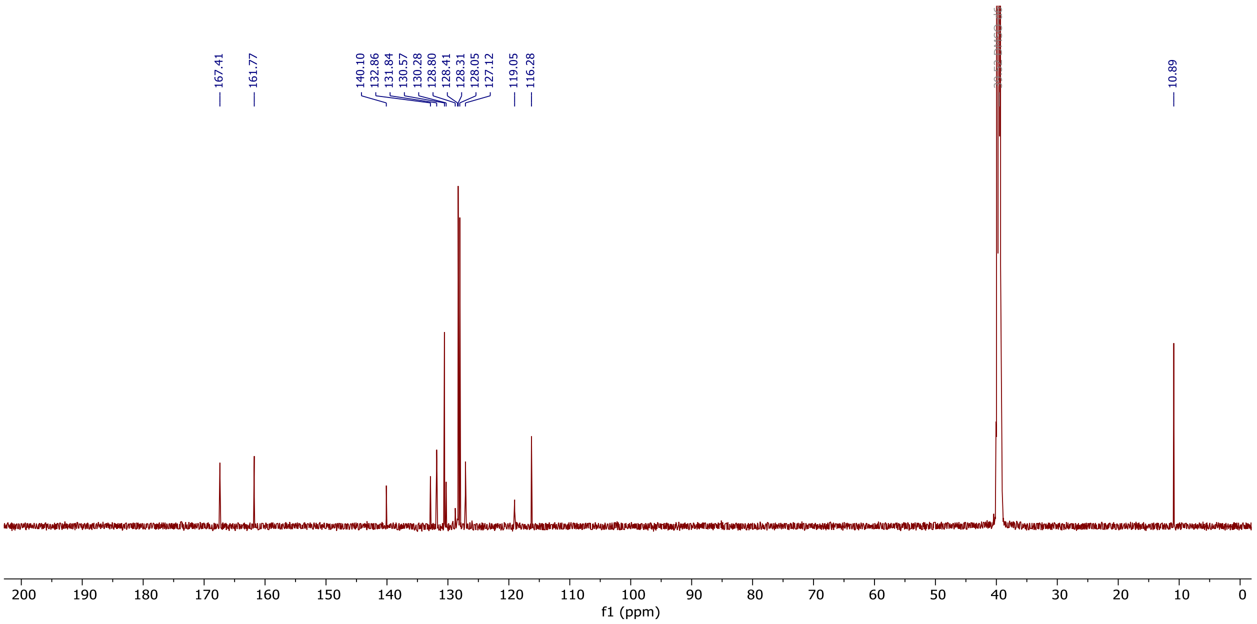

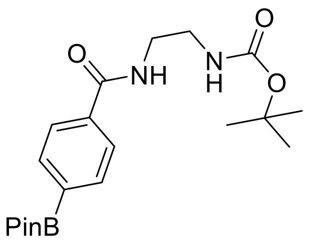

Compound **42g**

^{1}H NMR (600 MHz, $CDCl_3$)

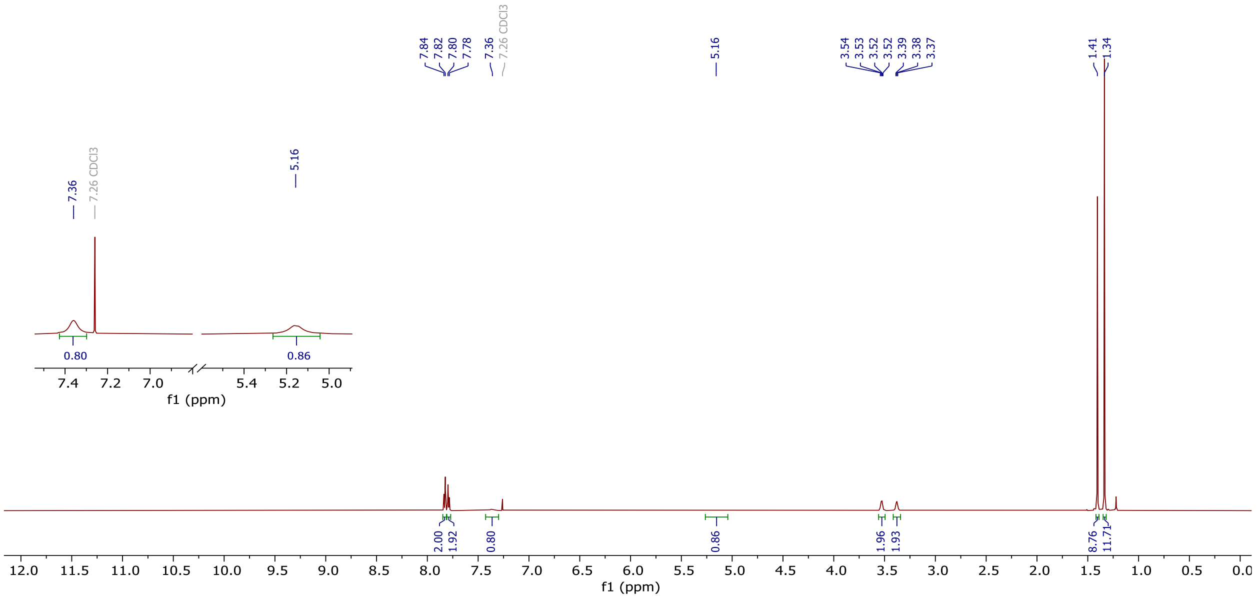

^{13}C NMR (151 MHz, $CDCl_3$)

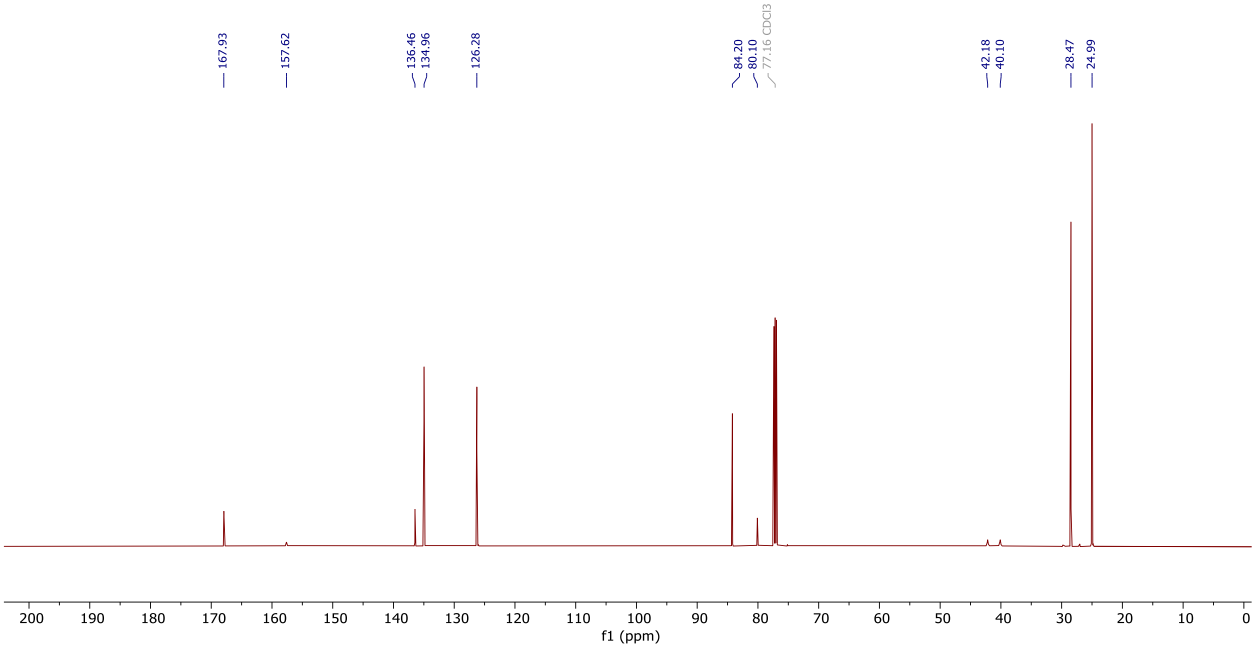

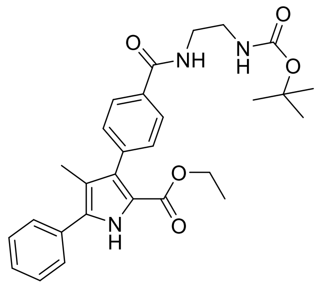

Compound **13a**

^{1}H NMR (600 MHz, DMSO-d_6)

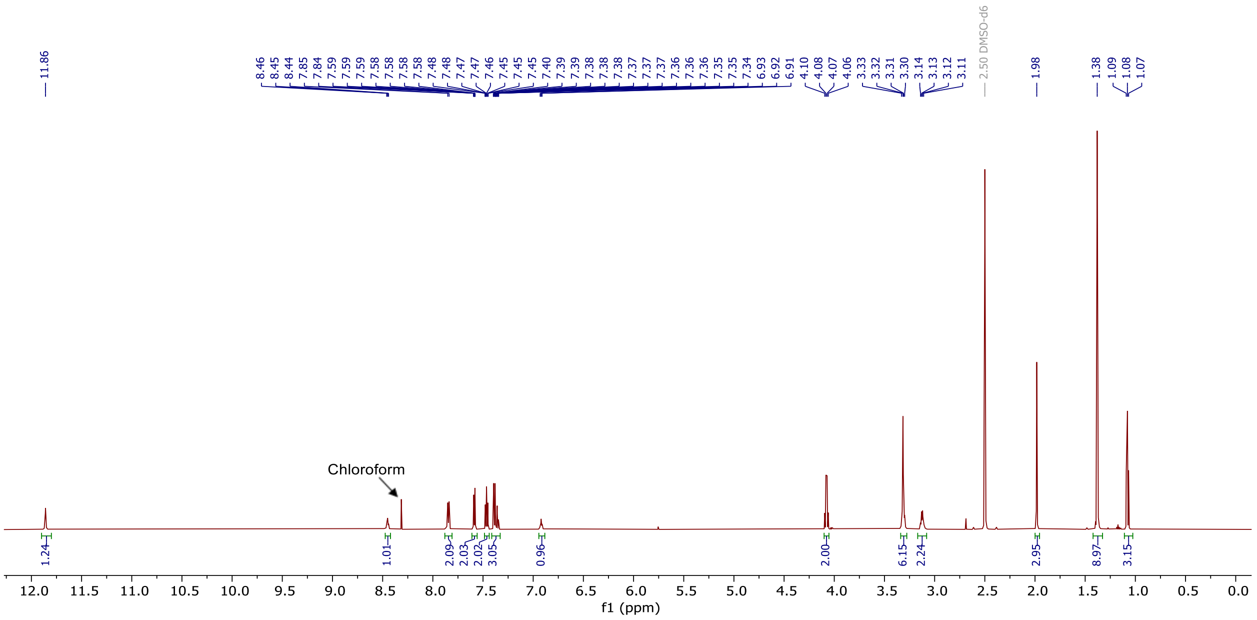

^{13}C NMR (151 MHz, DMSO-d_6)

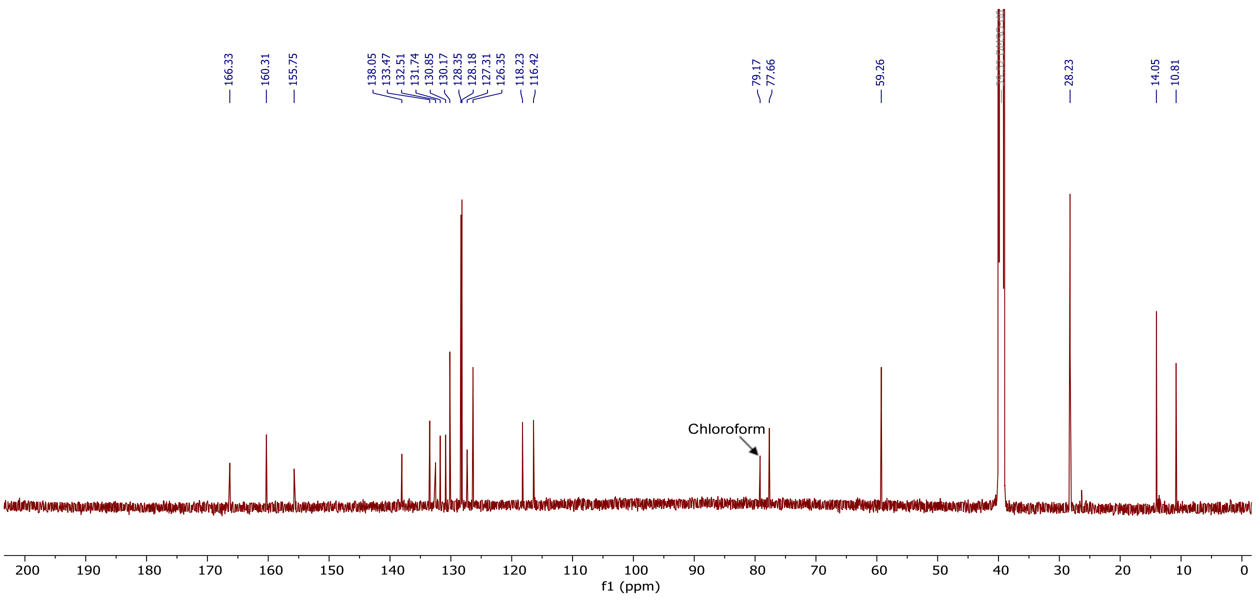

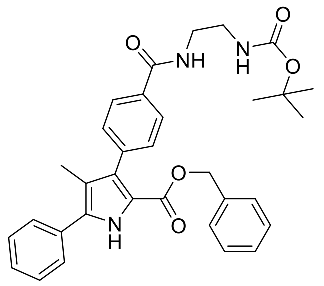

Compound **13b**

^{1}H NMR (600 MHz, DMSO-d_6)

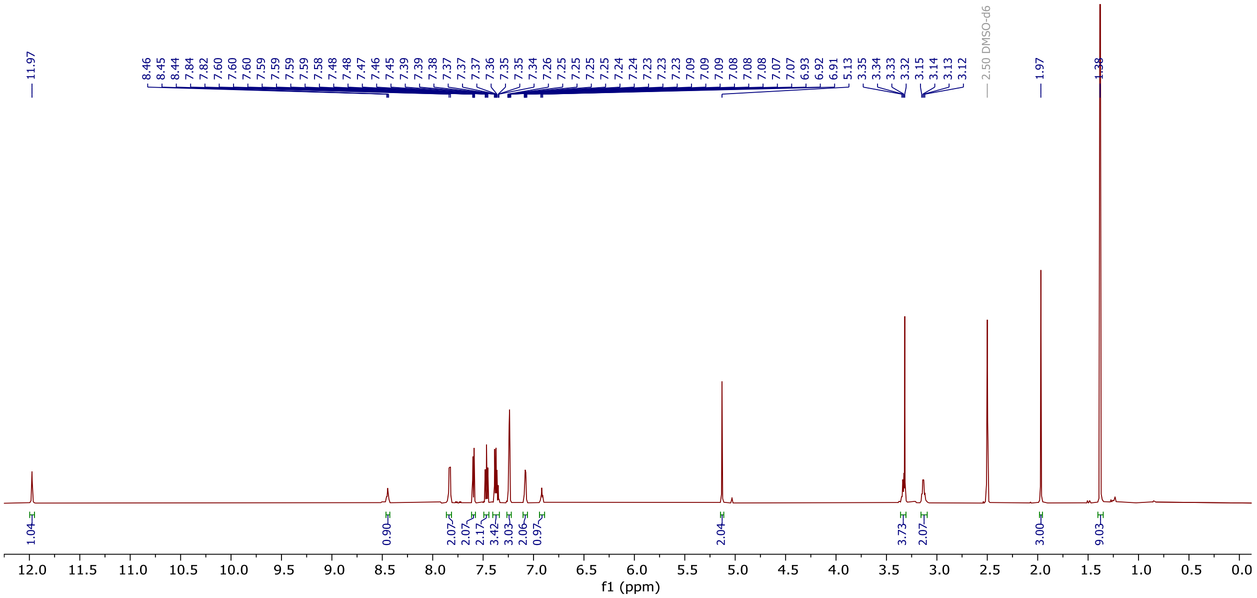

^{13}C NMR (151 MHz, DMSO-d_6)

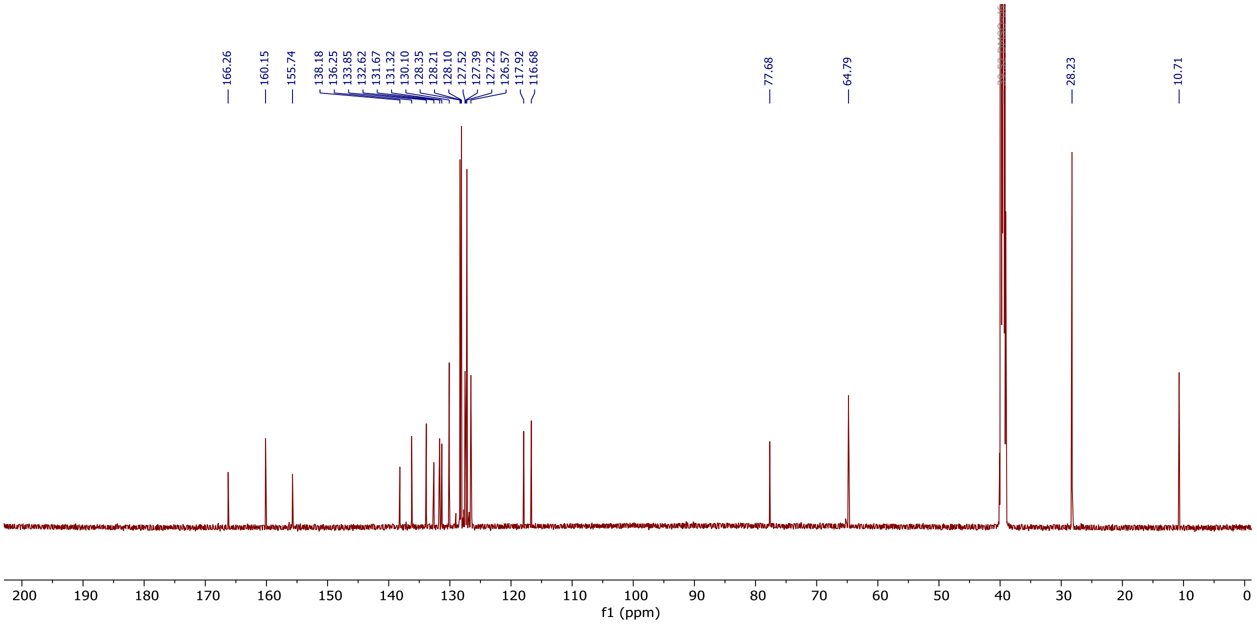

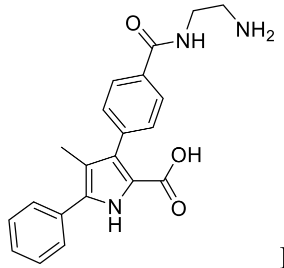

PyC 13

^{1}H NMR (600 MHz, DMSO-d_6)

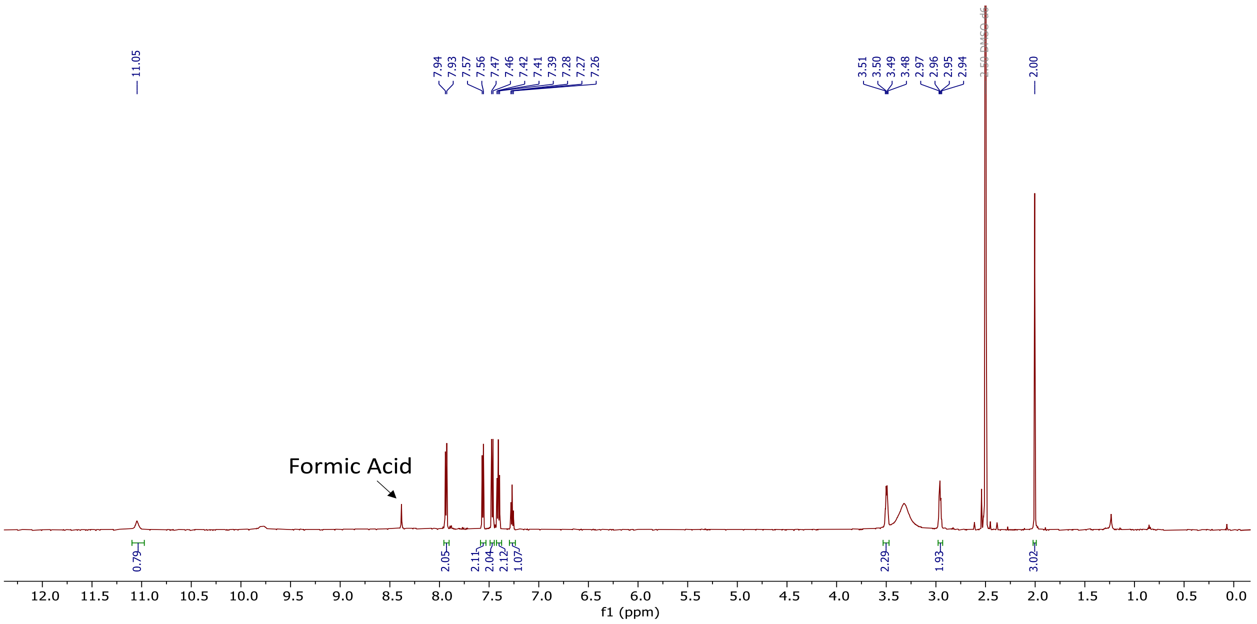

^{13}C NMR (151 MHz, DMSO-d_6)

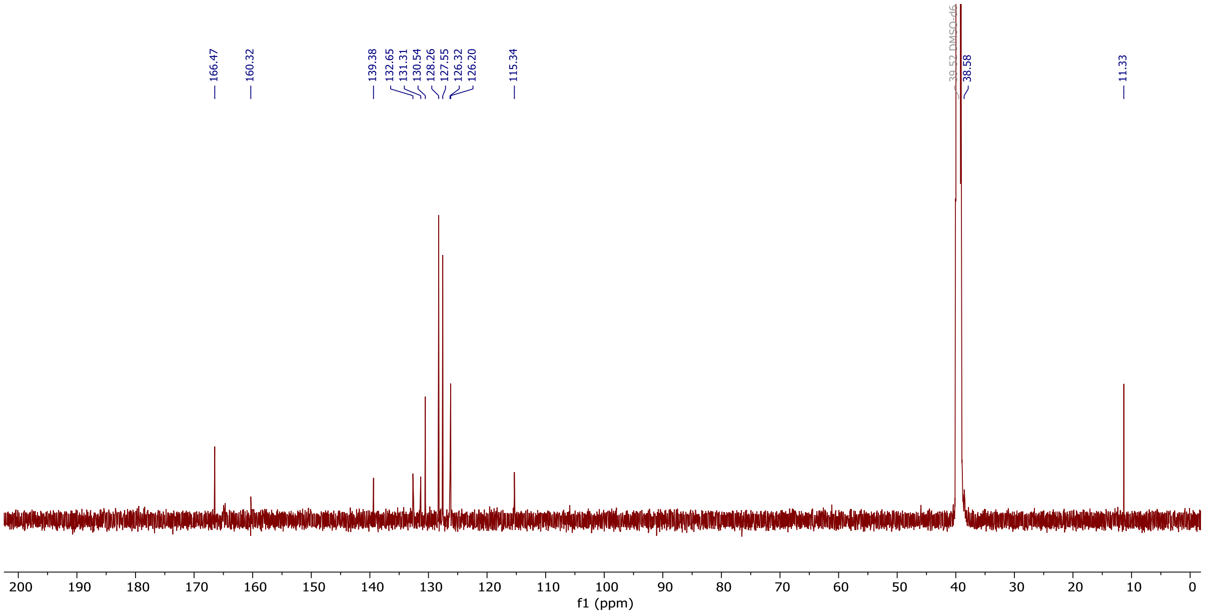

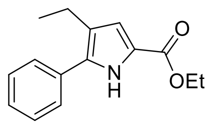

Compound **40c**

^{1}H NMR (400 MHz, $CDCl_3$)

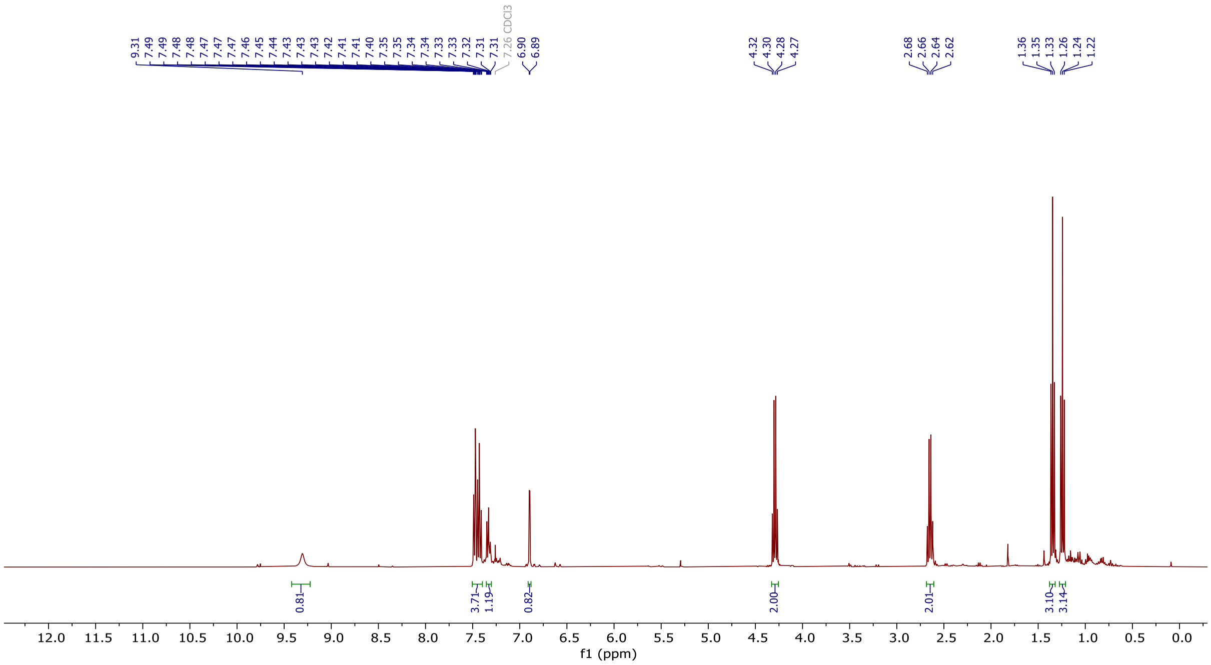

^{13}C NMR (101 MHz, $CDCl_3$)

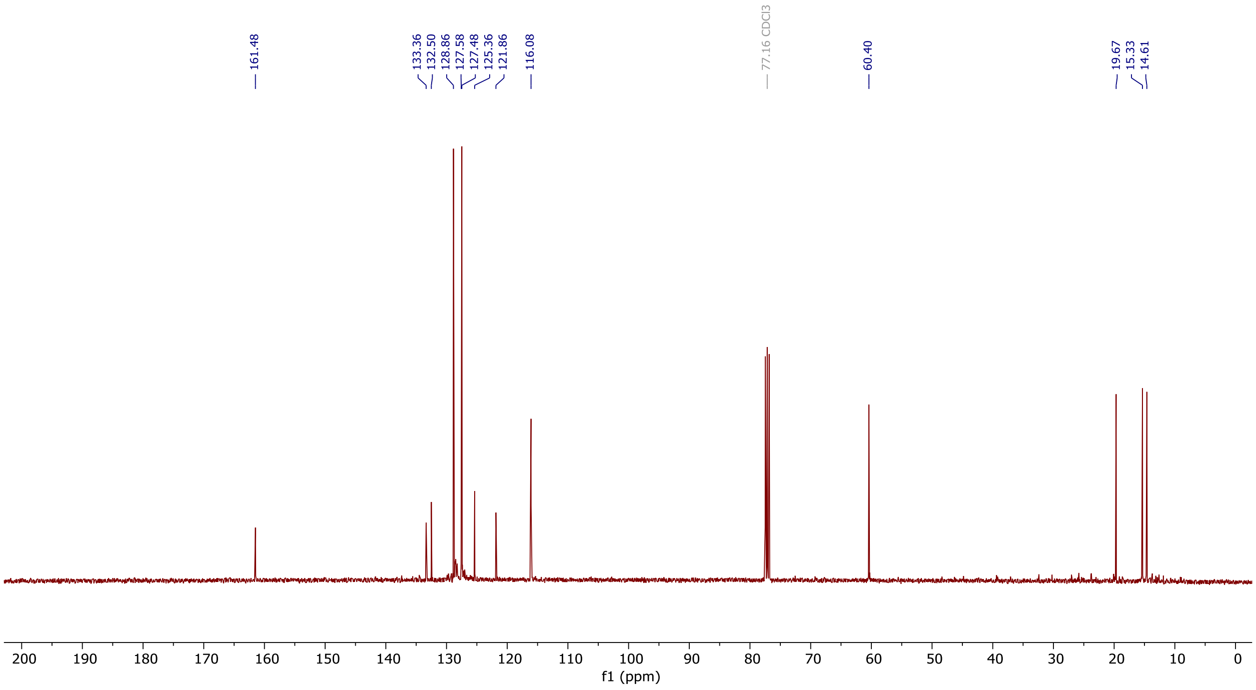

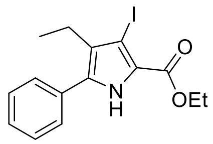

Compound **41c**

^{1}H NMR (400 MHz, CDCl$_3$)

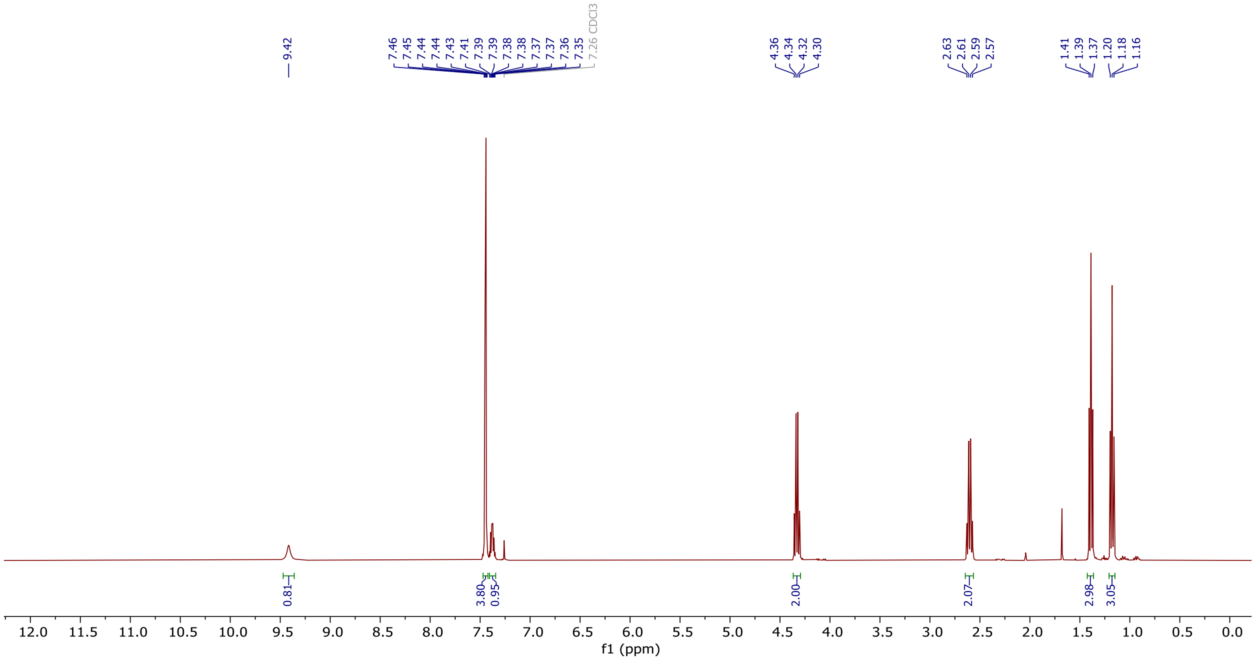

^{13}C NMR (101 MHz, CDCl$_3$)

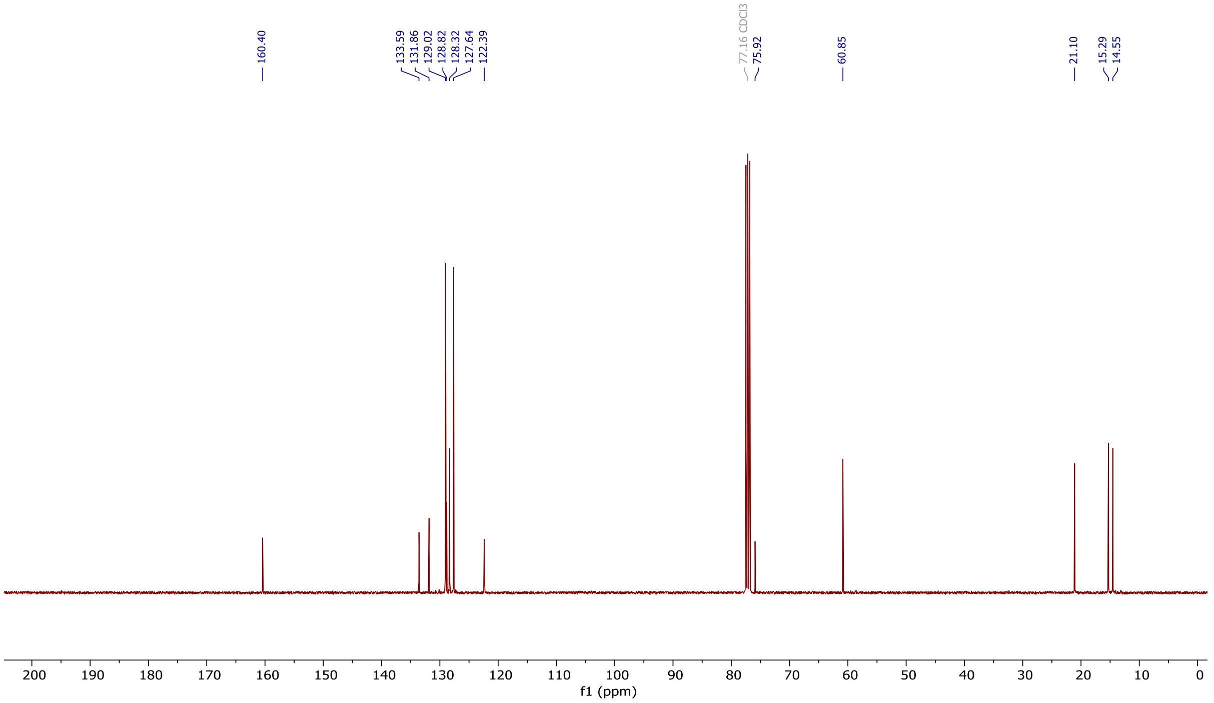

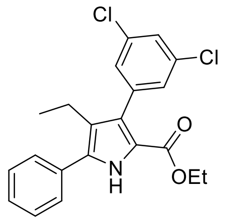

Compound **14a**

^{1}H NMR (500 MHz, DMSO-d_6)

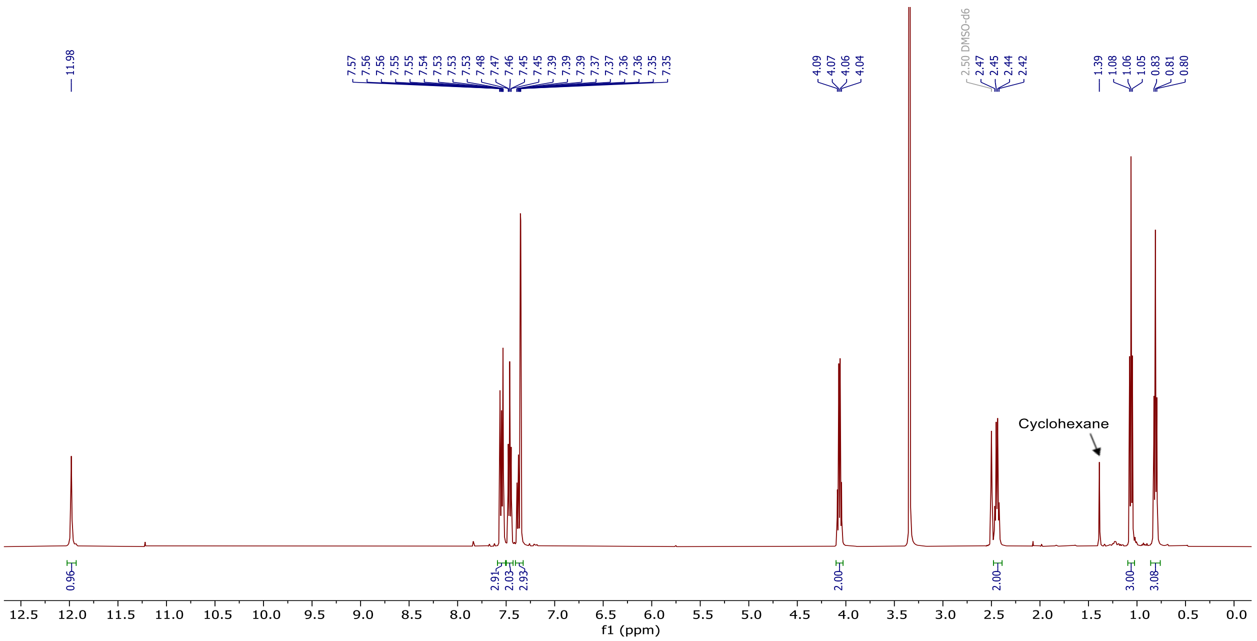

^{13}C NMR (126 MHz, DMSO-d_6)

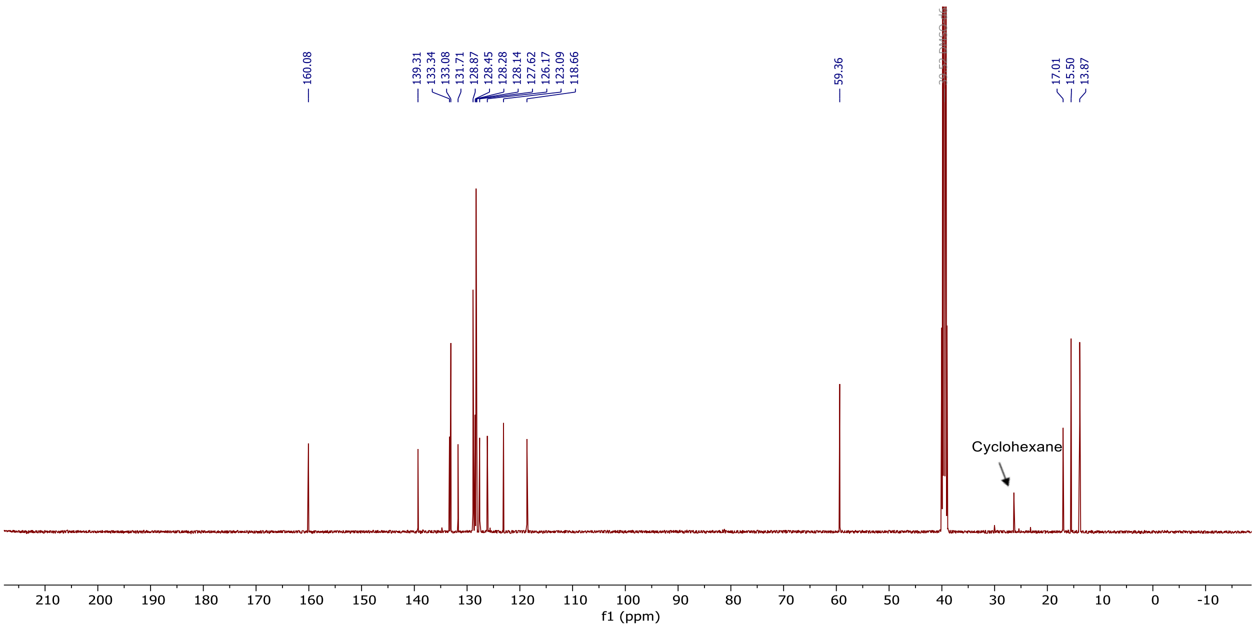

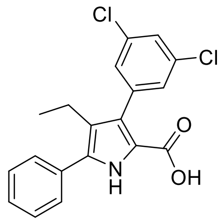

PyC 14

^{1}H NMR (600 MHz, DMSO-d_6)

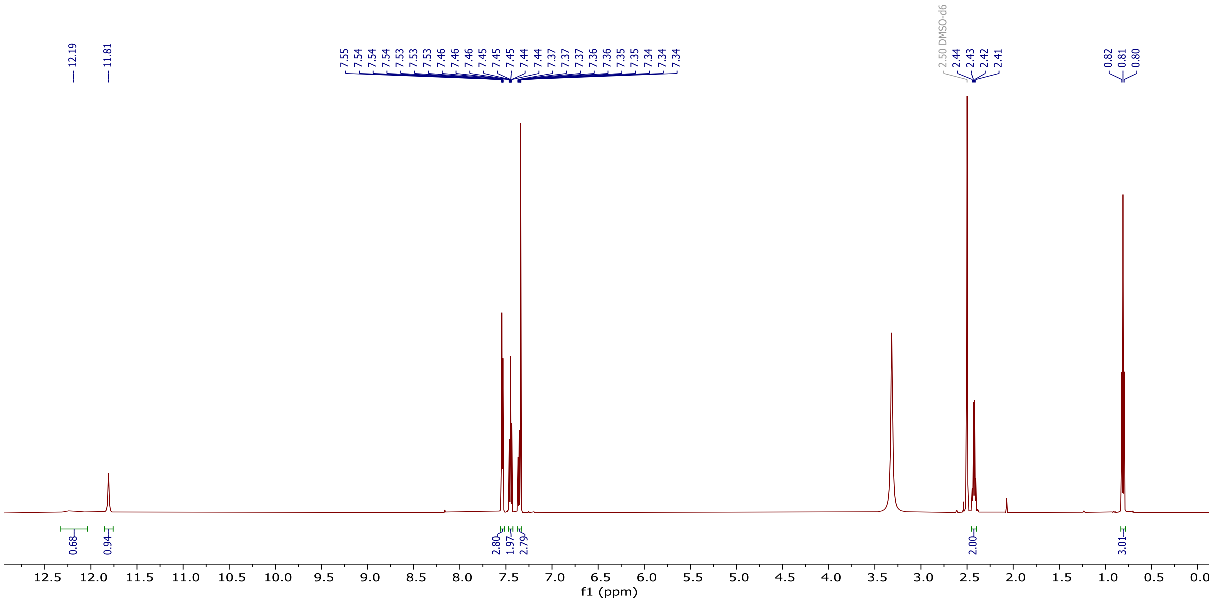

^{13}C NMR (151 MHz, DMSO-d_6)

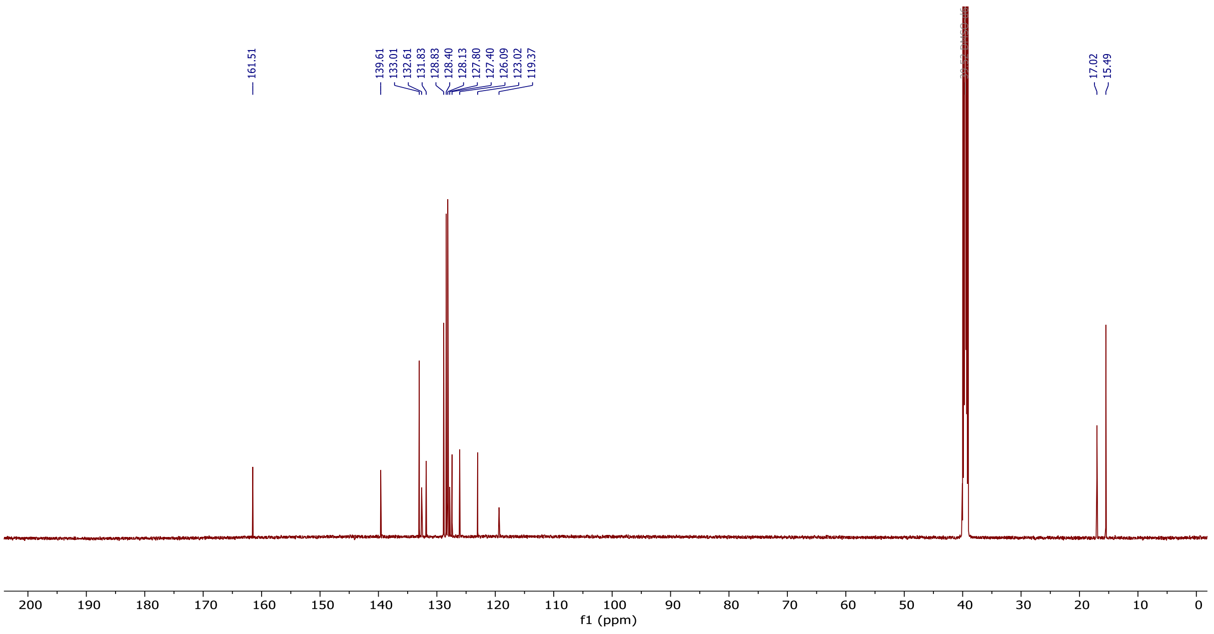

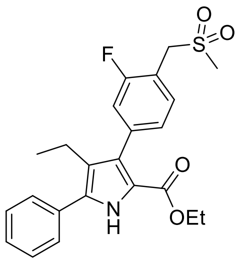

Compound **15a**

^{1}H NMR (500 MHz, DMSO-d_6)

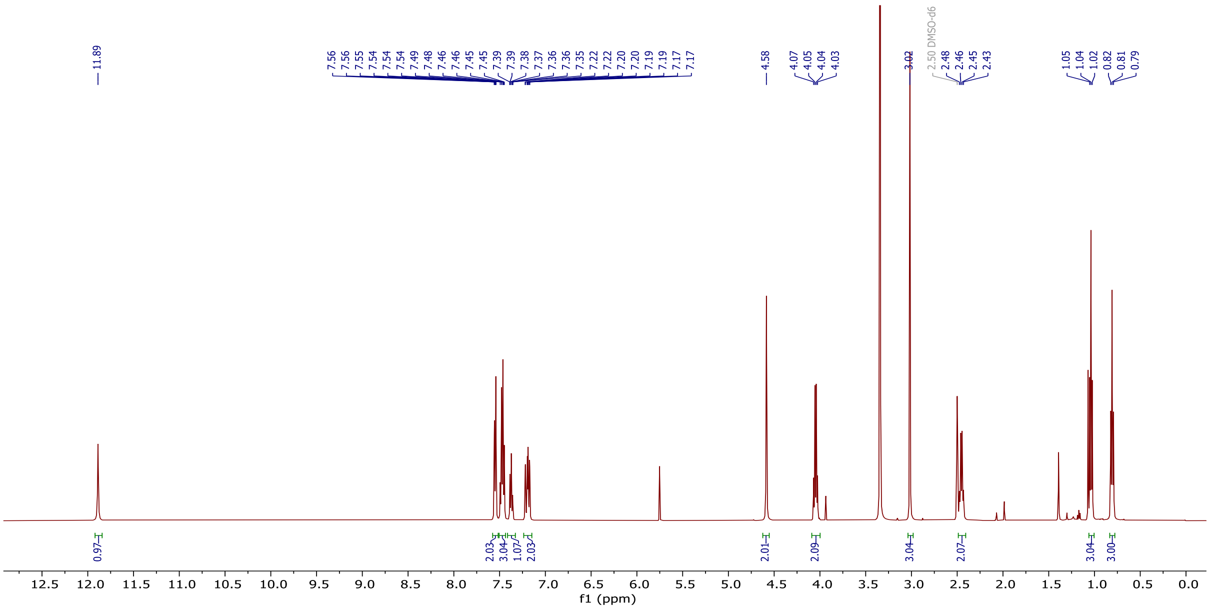

^{13}C NMR (126 MHz, DMSO-d_6)

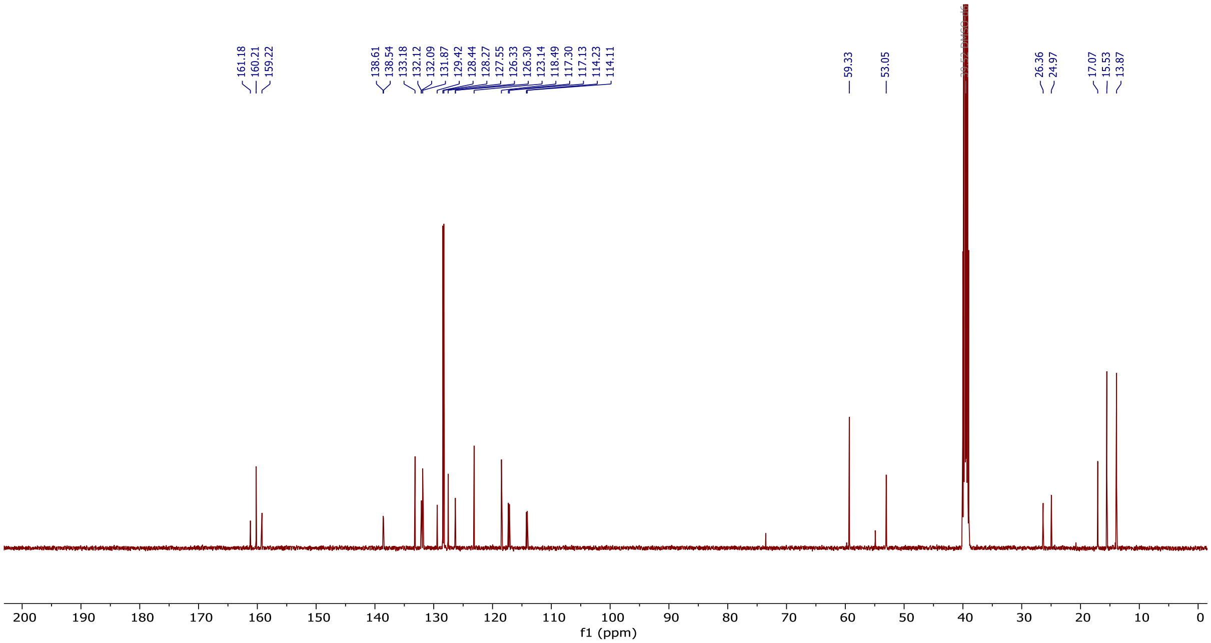

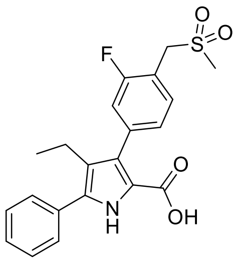

PyC 15

^{1}H NMR (600 MHz, DMSO-d_6)

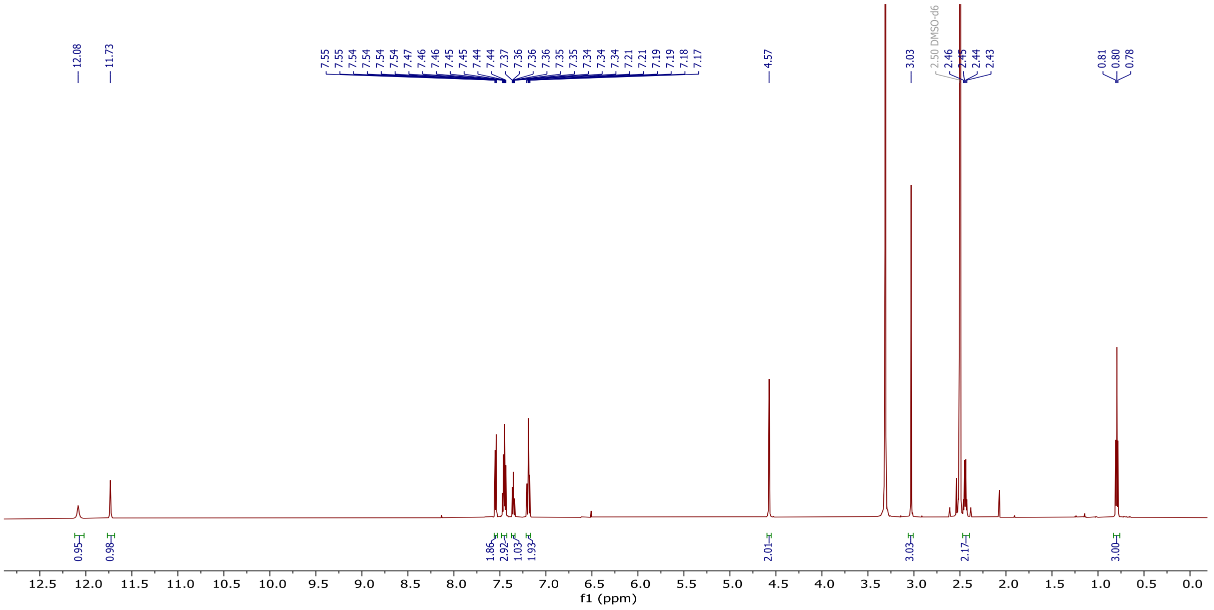

^{13}C NMR (151 MHz, DMSO-d_6)

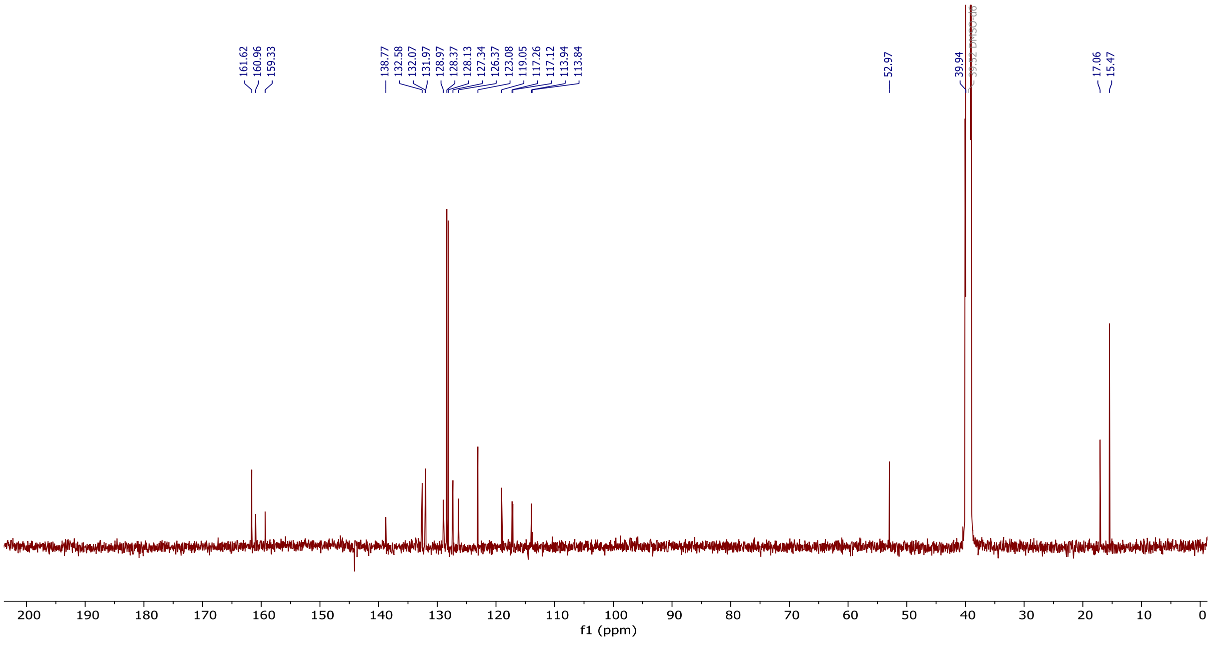

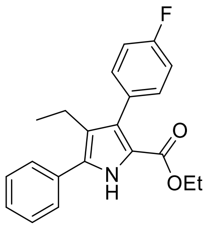

Compound **16a**

^{1}H NMR (400 MHz, DMSO-d_6)

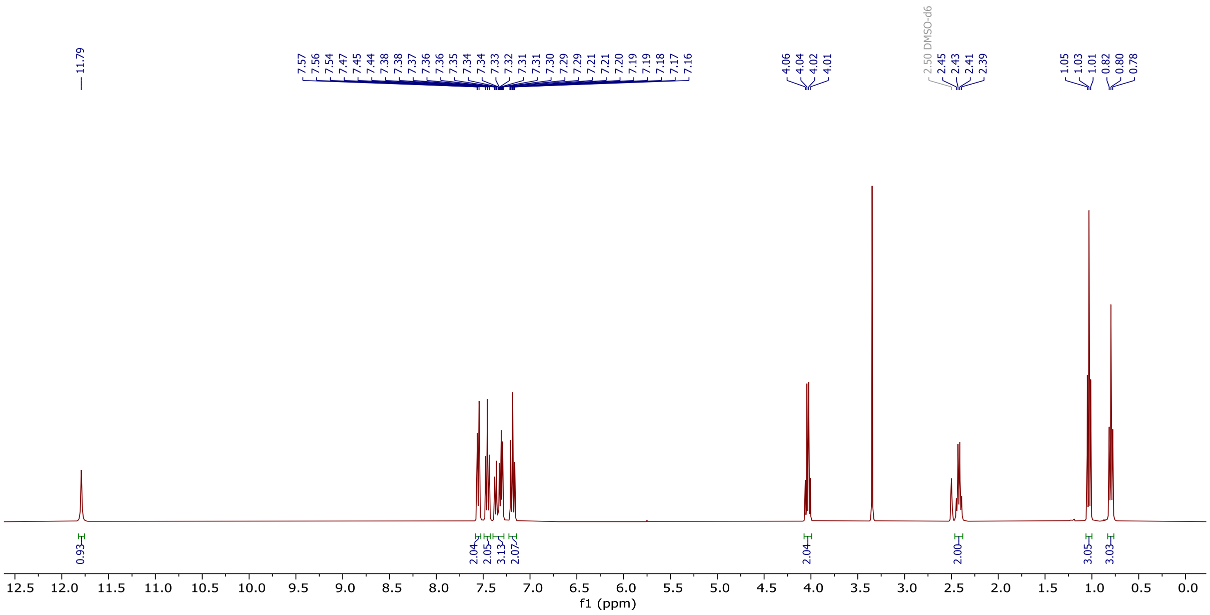

^{13}C NMR (101 MHz, DMSO-d_6)

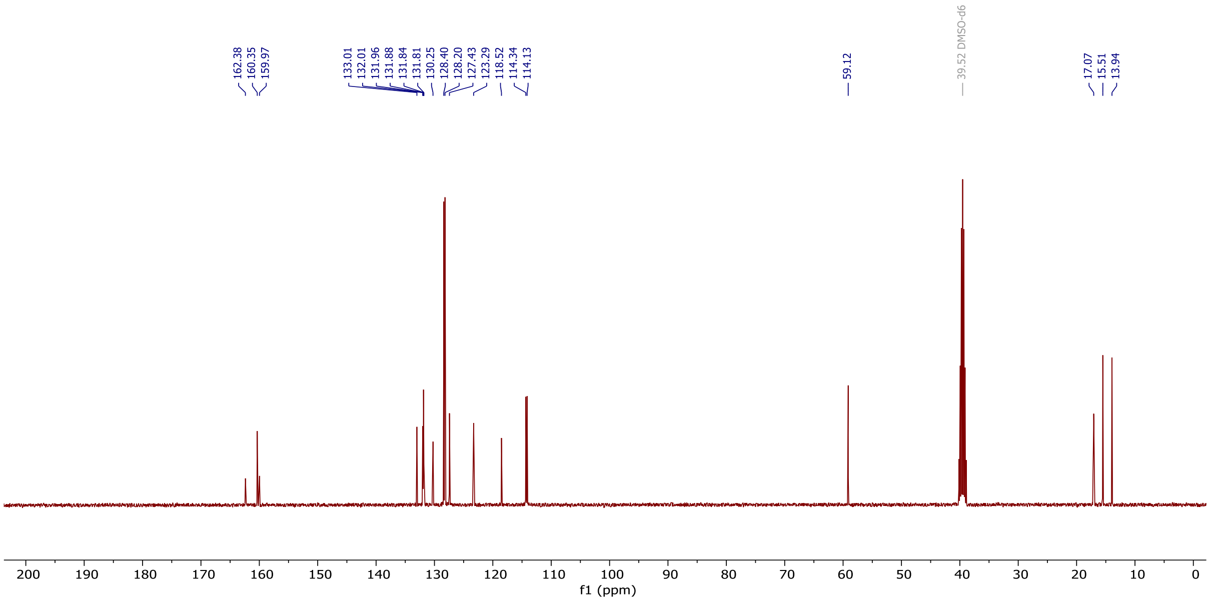

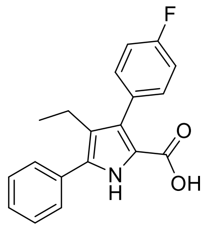

PyC 16

^{1}H NMR (600 MHz, DMSO-d_6)

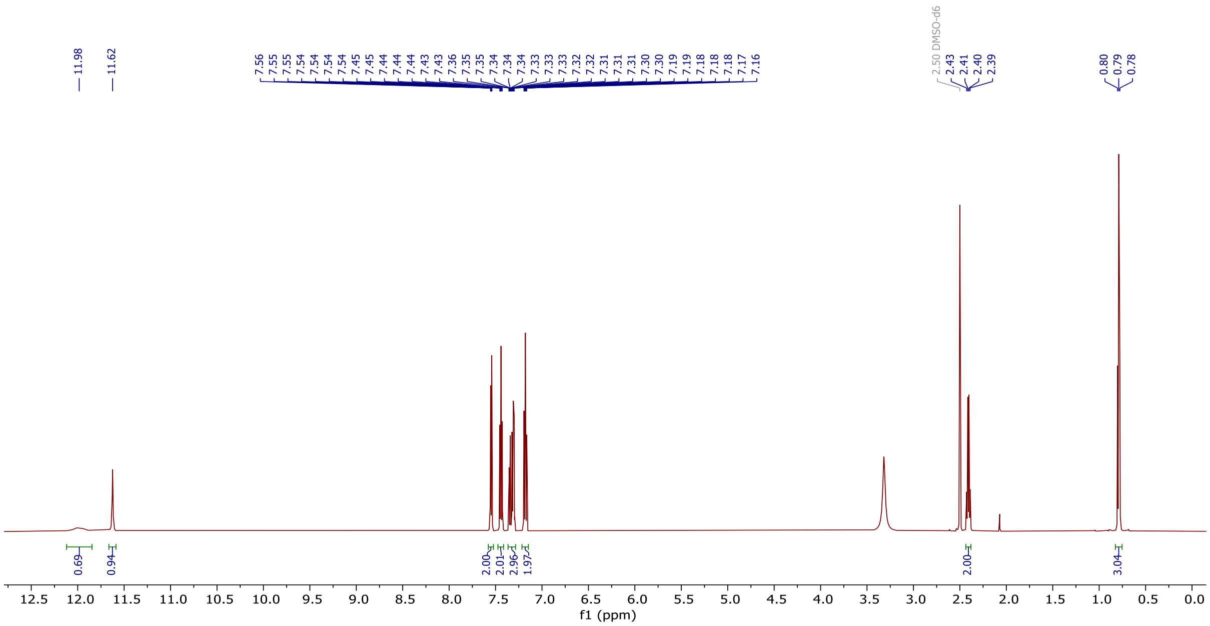

^{13}C NMR (151 MHz, DMSO-d_6)

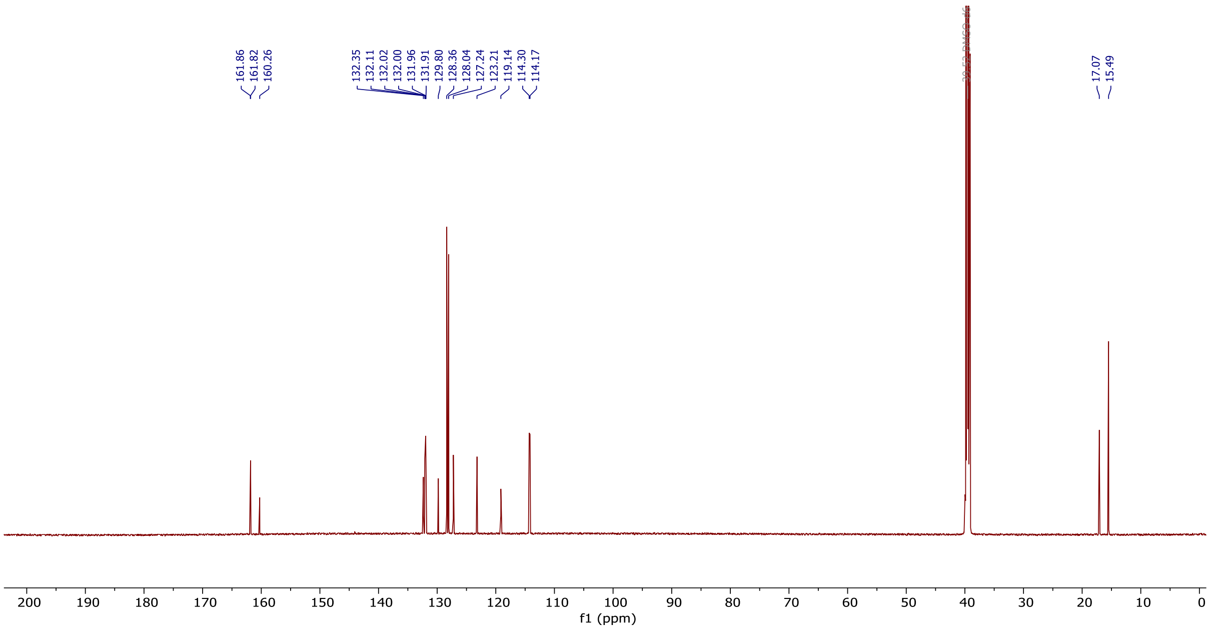

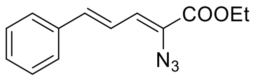

Compound **39c**

^{1}H NMR (500 MHz, $CDCl_3$)

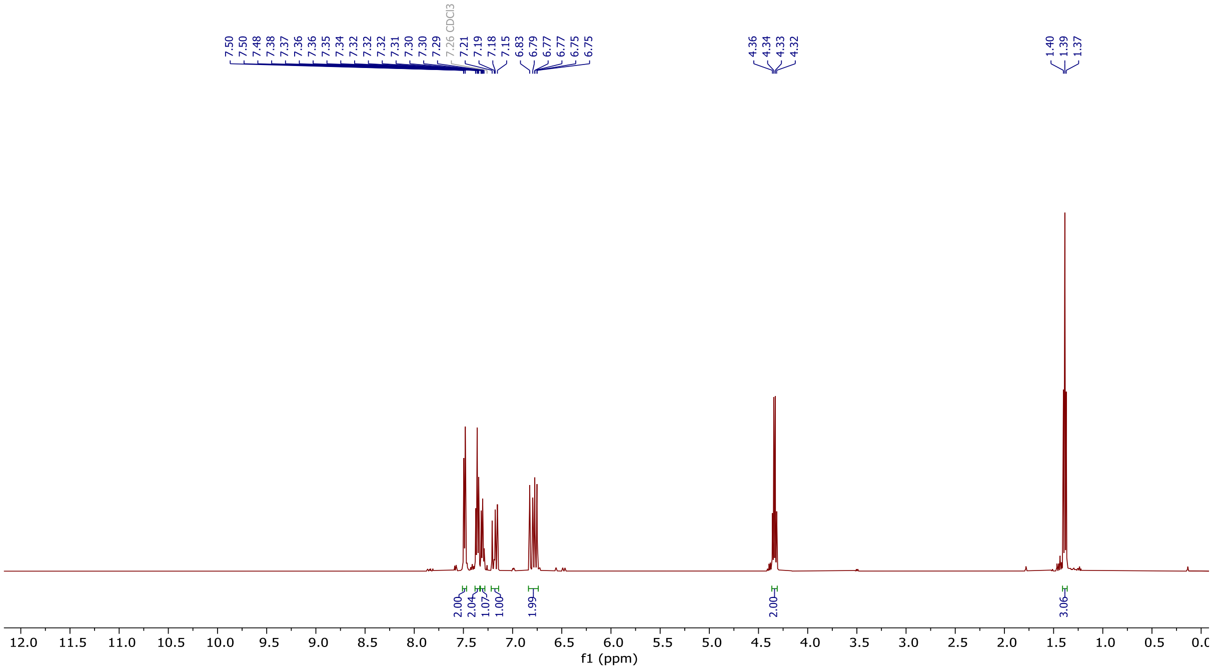

^{13}C NMR (126 MHz, $CDCl_3$)

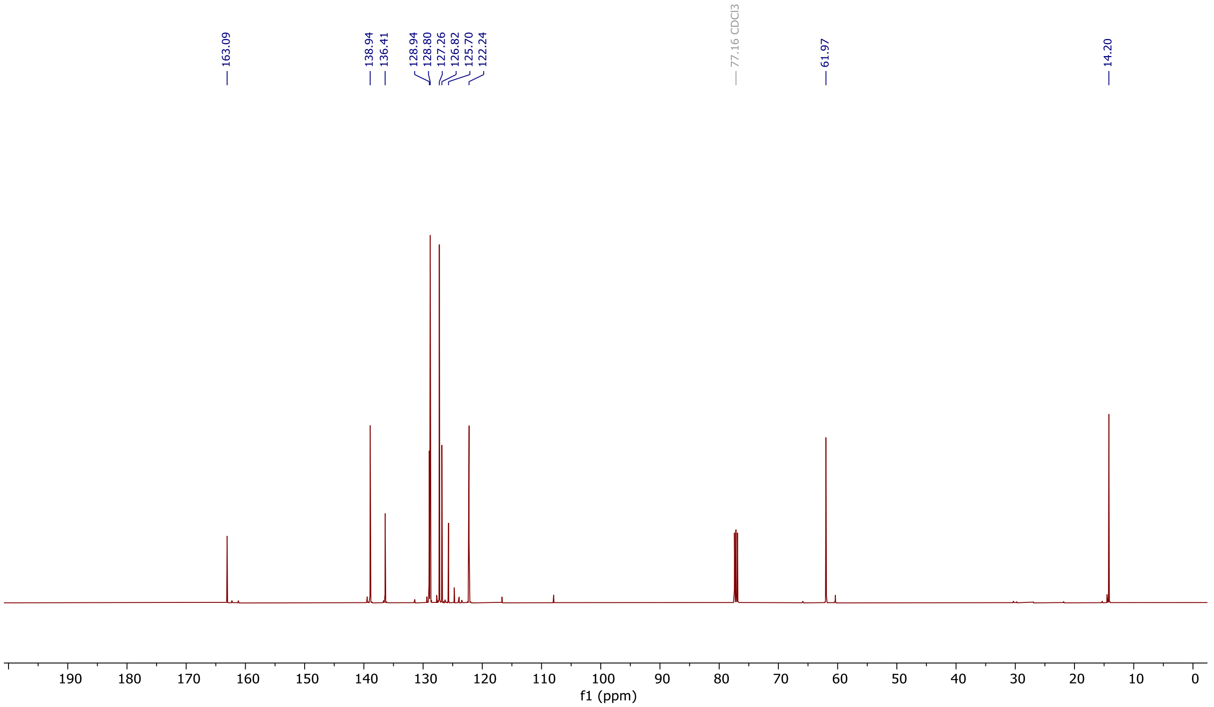

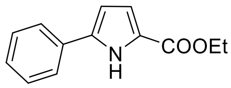

Compound **40d**

^{1}H NMR (600 MHz, $CDCl_3$)

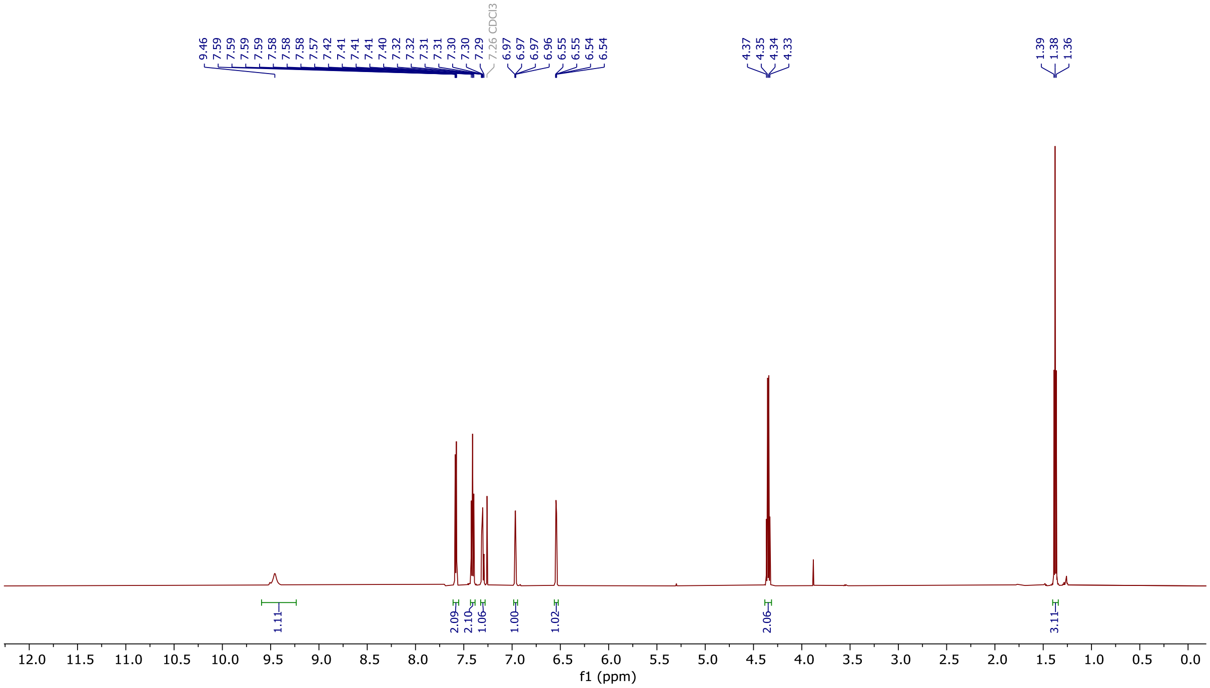

^{13}C NMR (151 MHz, $CDCl_3$)

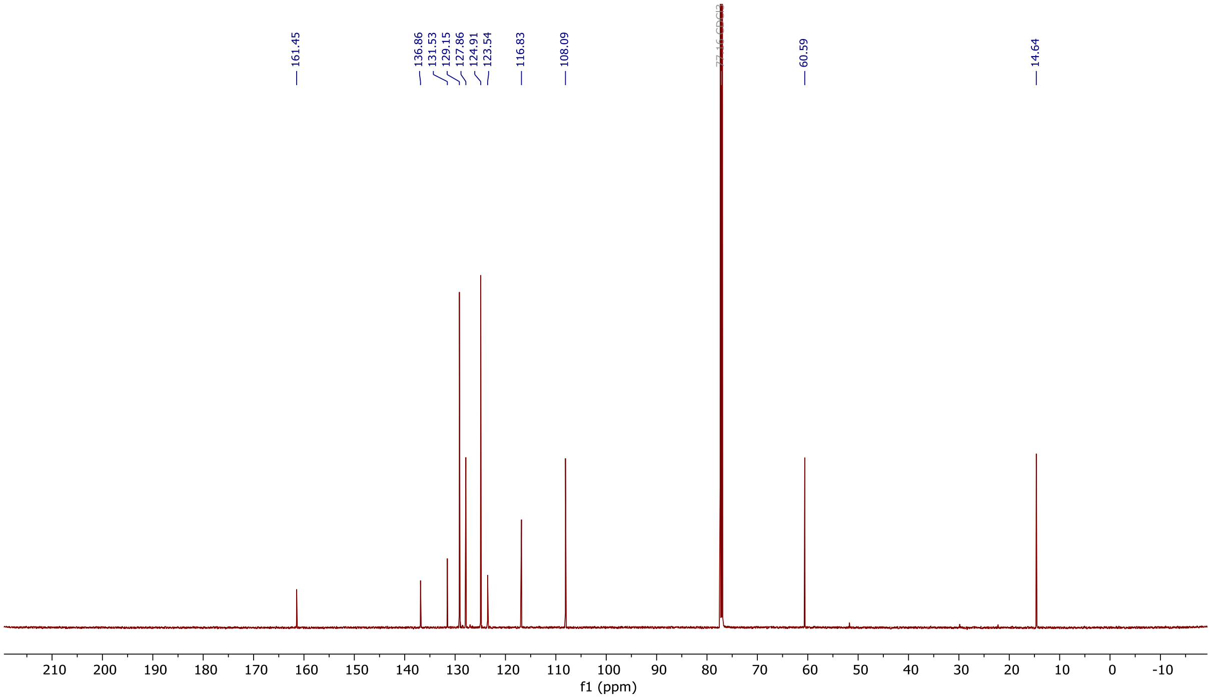

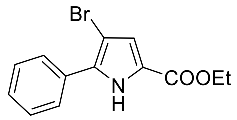

Compound **43′**

^{1}H NMR (600 MHz, $CDCl_3$)

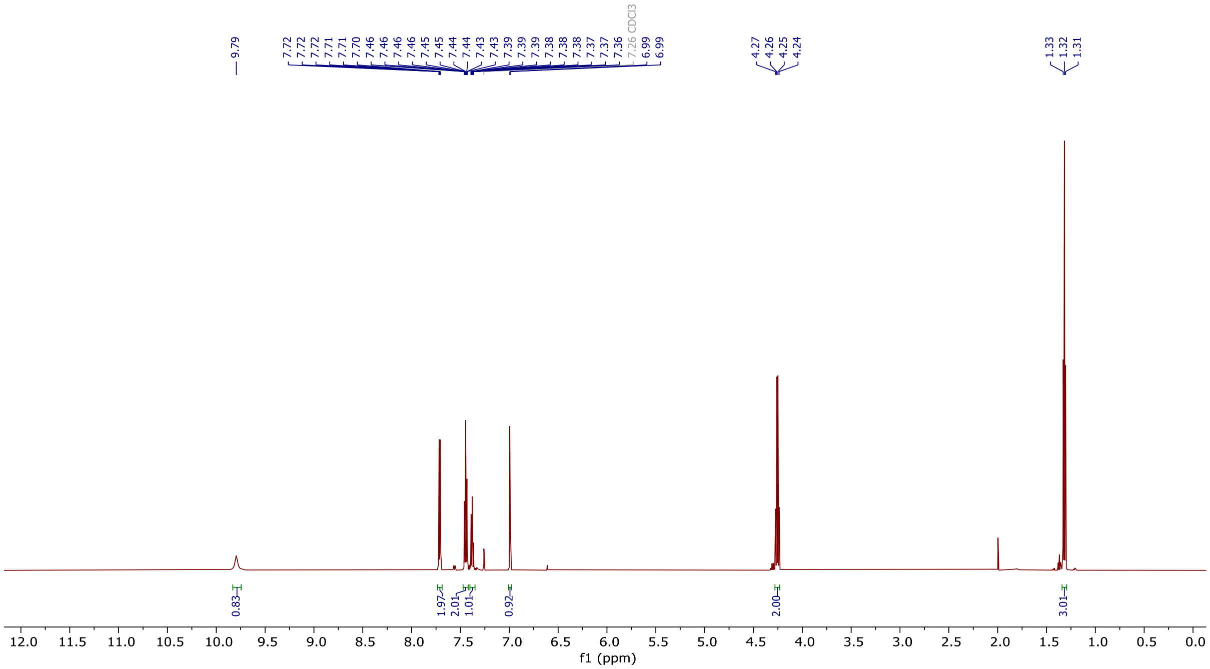

^{13}C NMR (151 MHz, $CDCl_3$)

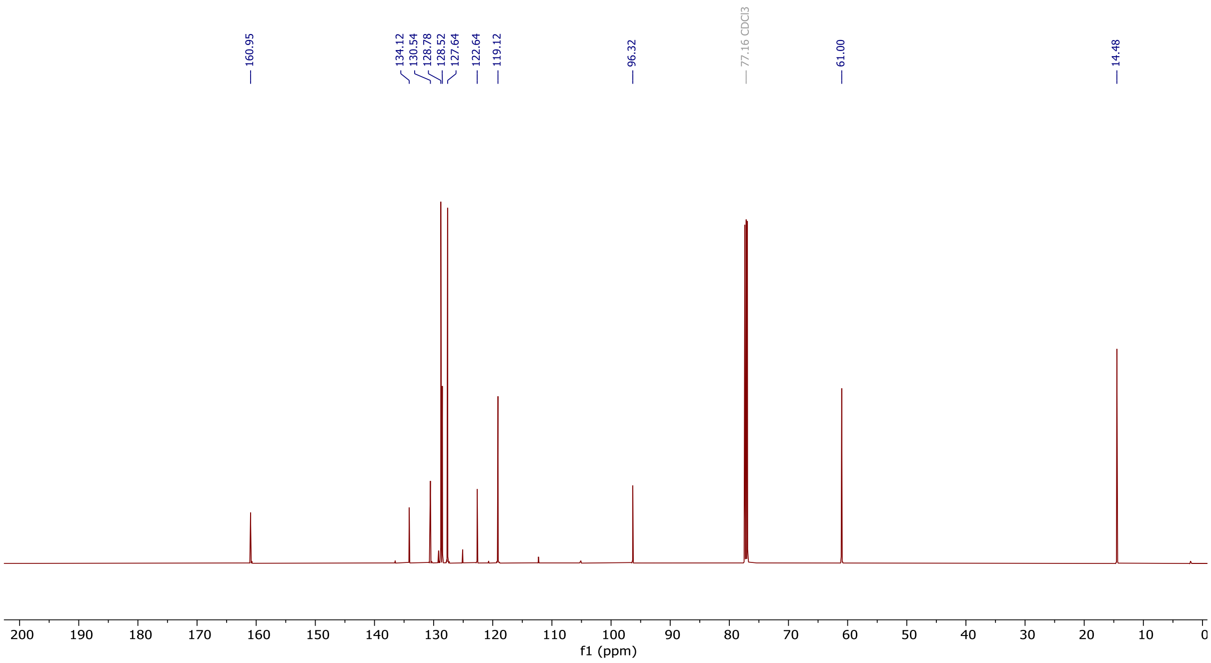

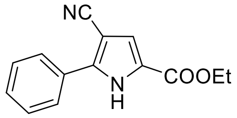

Compound **43**

^{1}H NMR (600 MHz, $CDCl_3$)

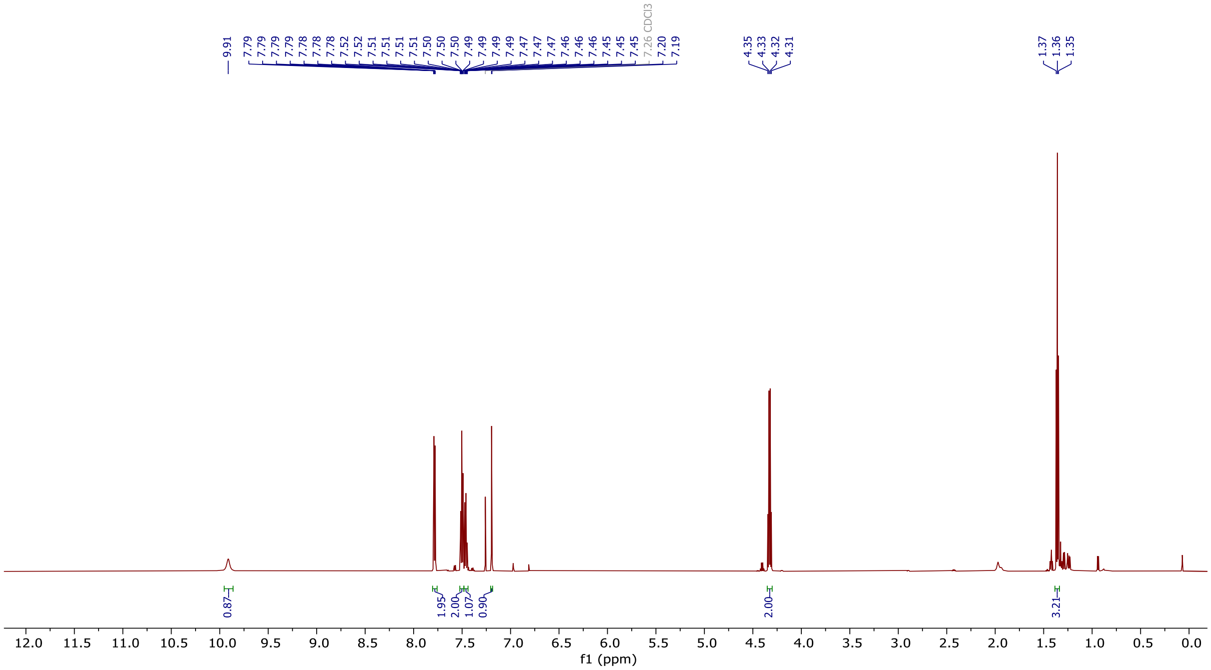

^{13}C NMR (151 MHz, $CDCl_3$)

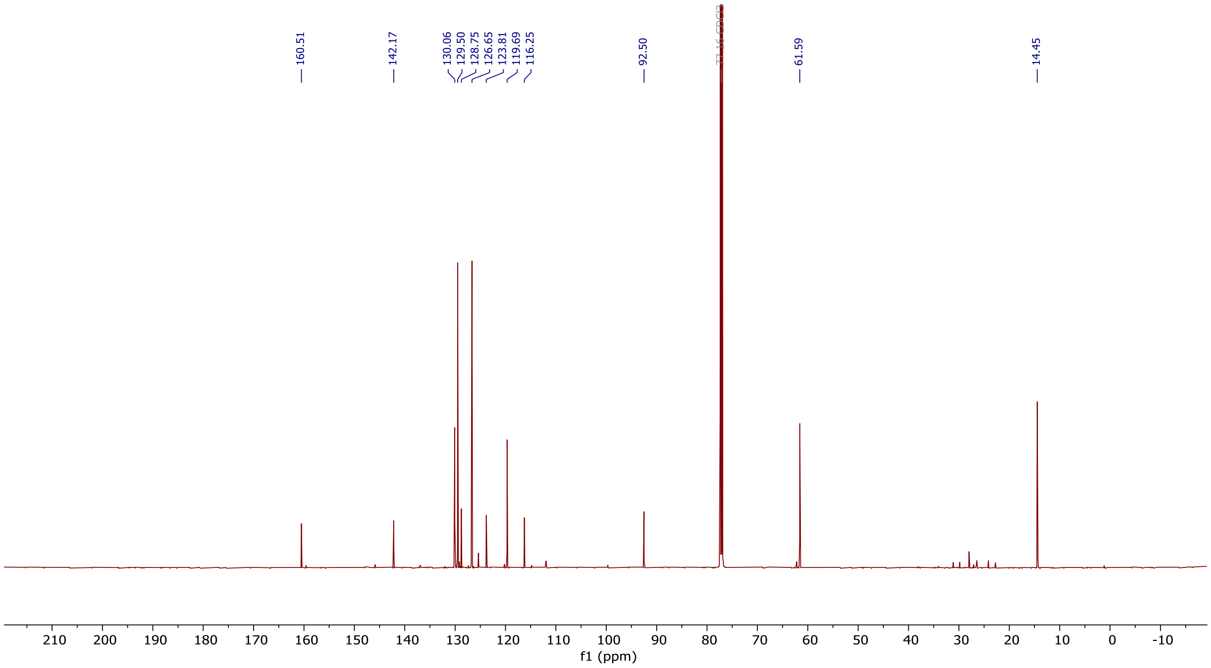

HMBC NMR_Compound **43**

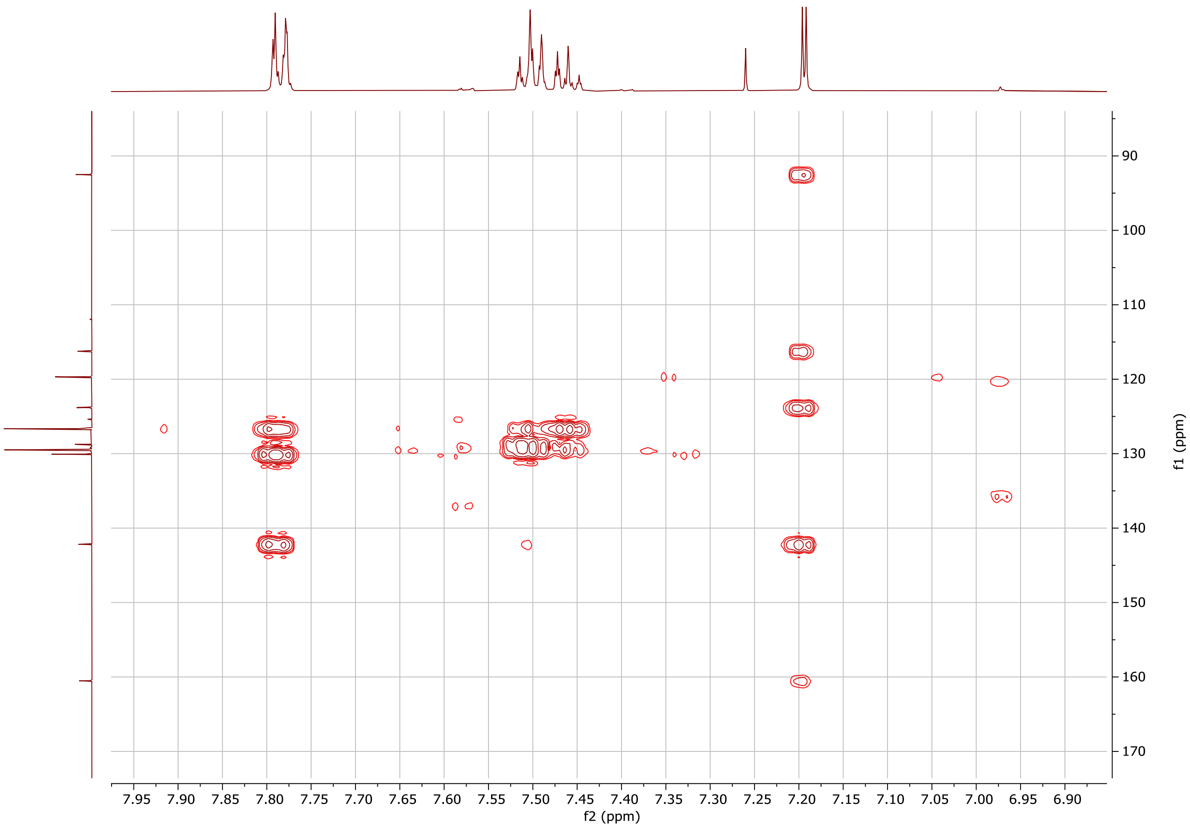

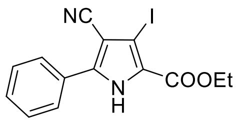

Compound **45**

^{1}H NMR (700 MHz, DMSO-d_6)

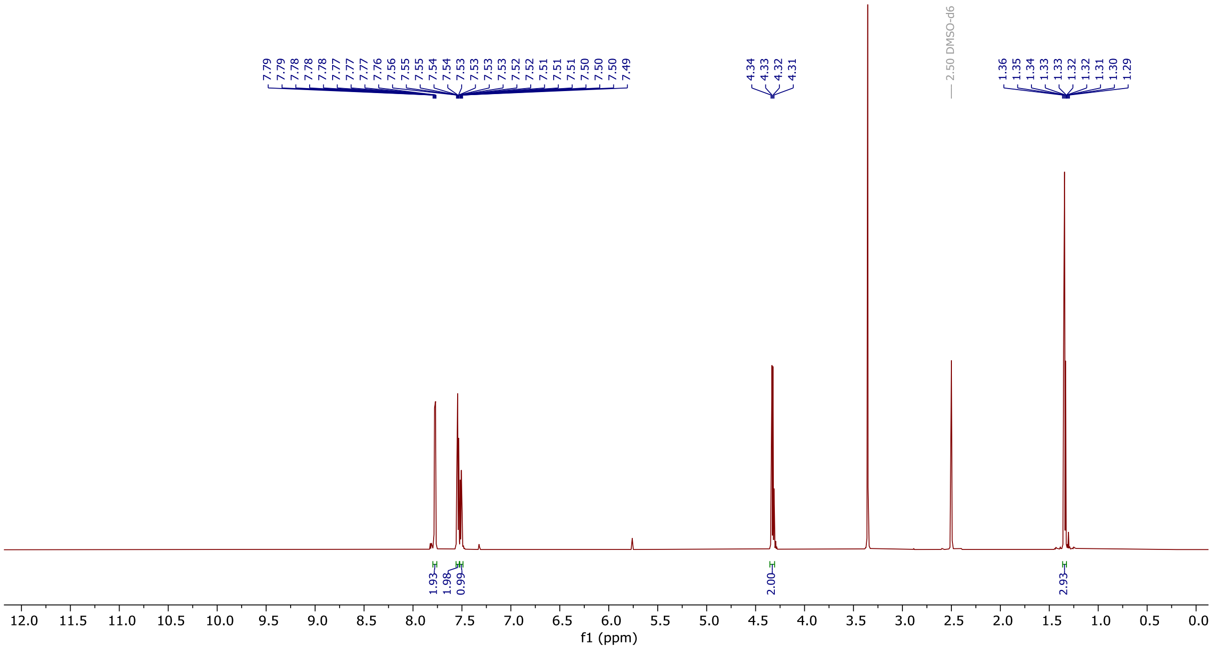

^{13}C NMR (176 MHz, DMSO-d_6)

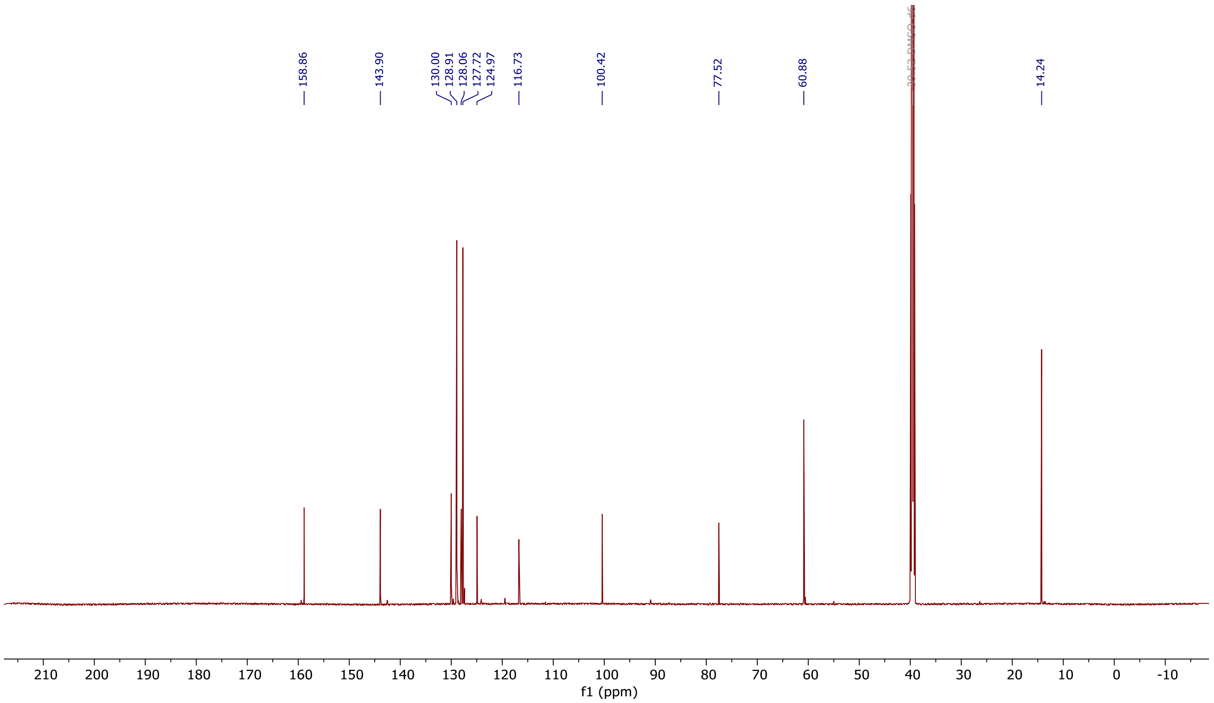

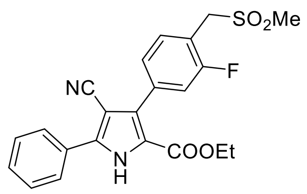

Compound **17a**

^{1}H NMR (600 MHz, DMSO-d_6)

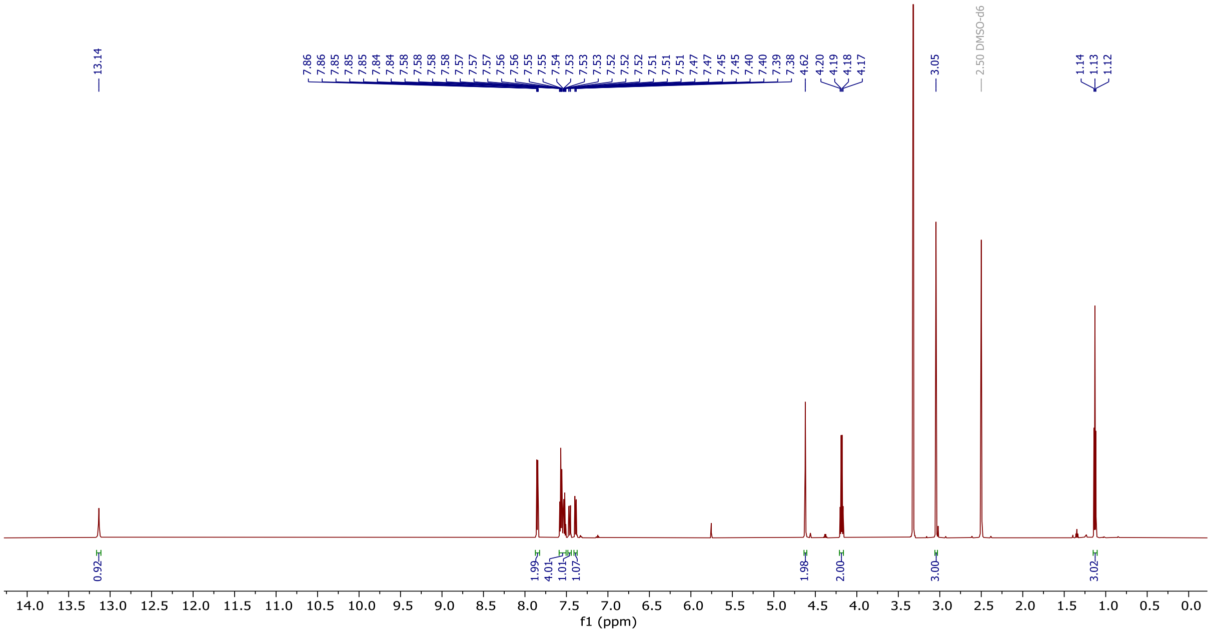

^{13}C NMR (151 MHz, DMSO-d_6)

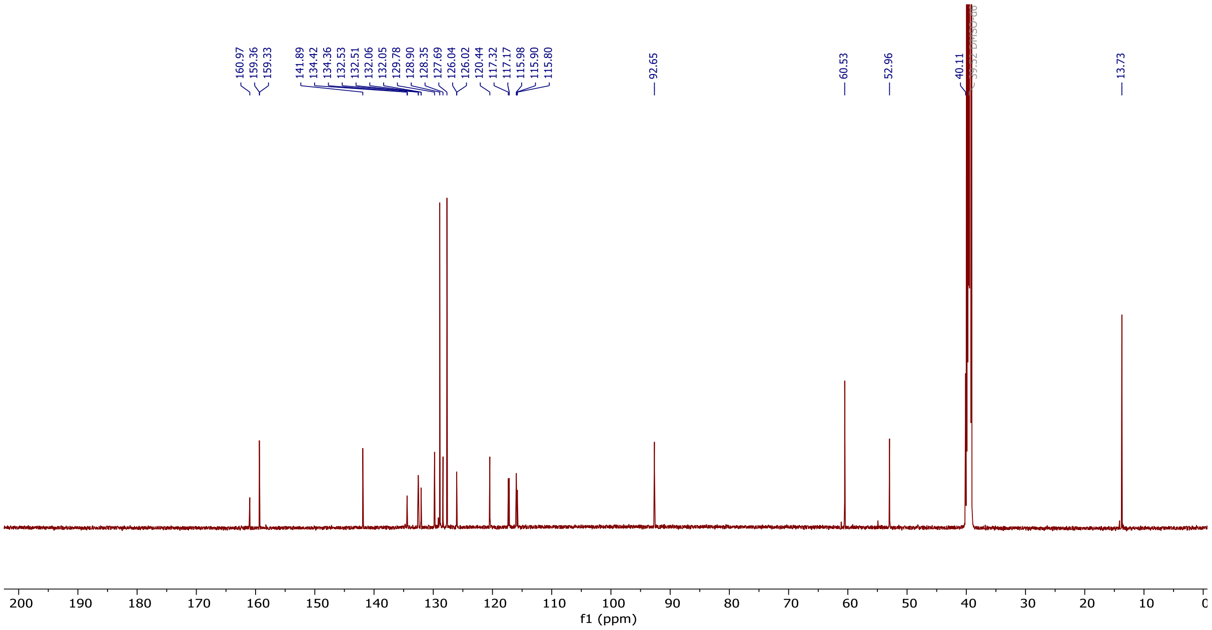

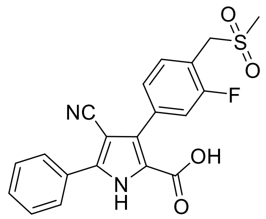

PyC 17

^{1}H NMR (600 MHz, MeOH-d_4)

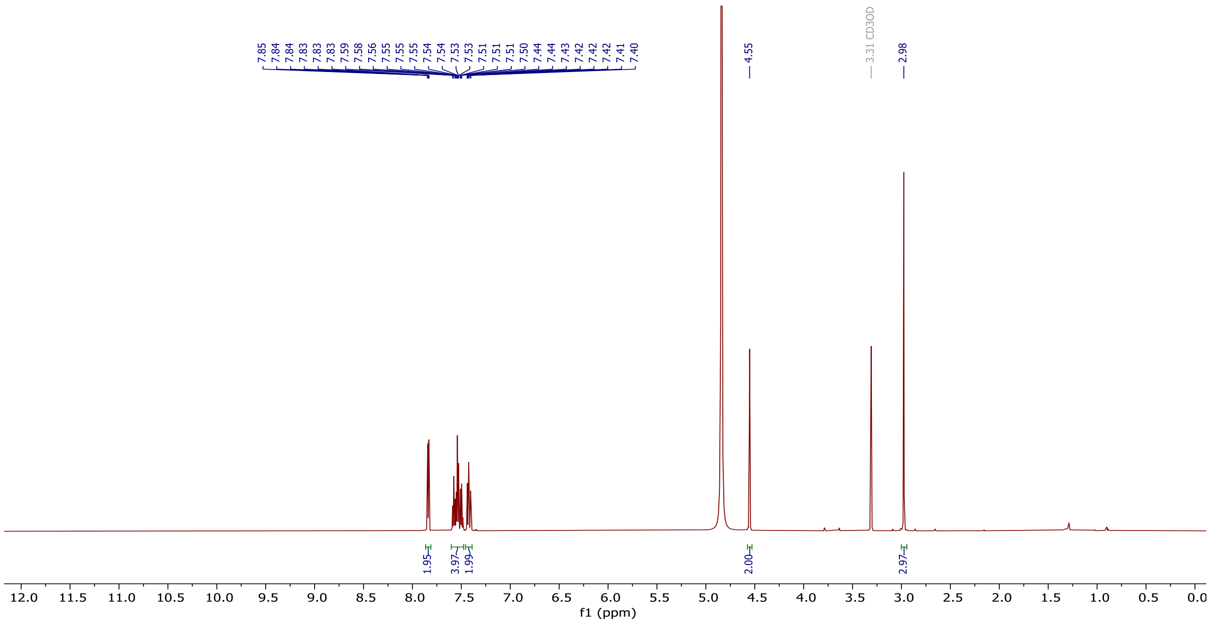

^{13}C NMR (151 MHz, MeOH-d_4)

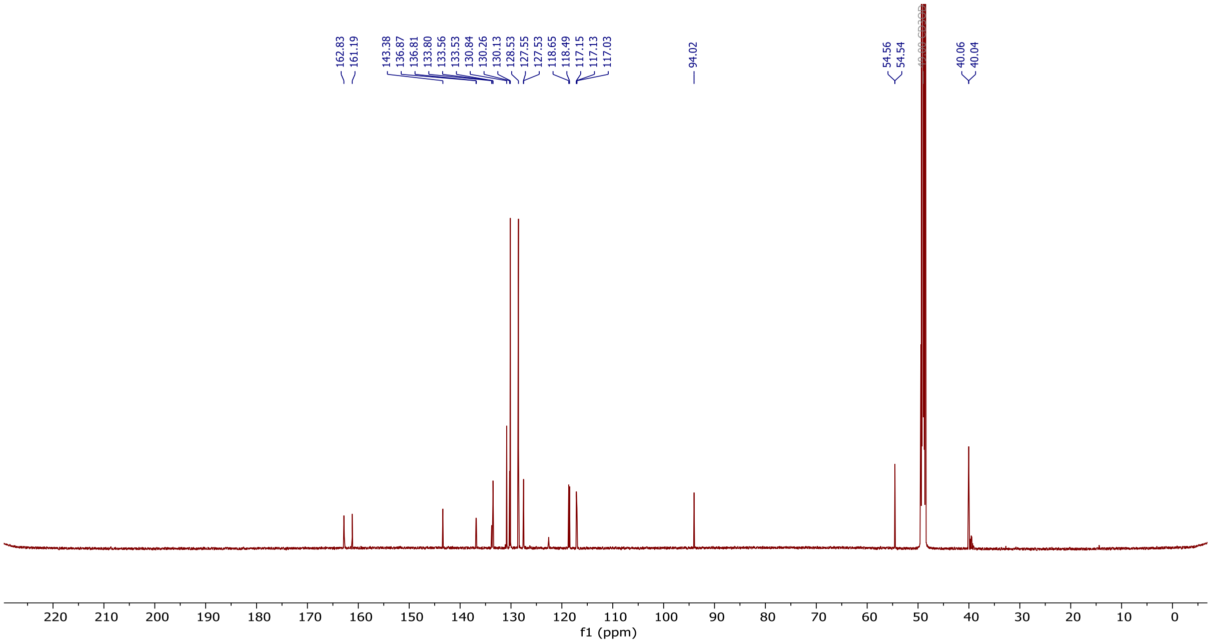

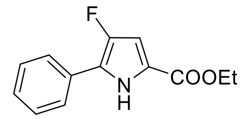

Compound **44**

^{1}H NMR (400 MHz, $CDCl_3$)

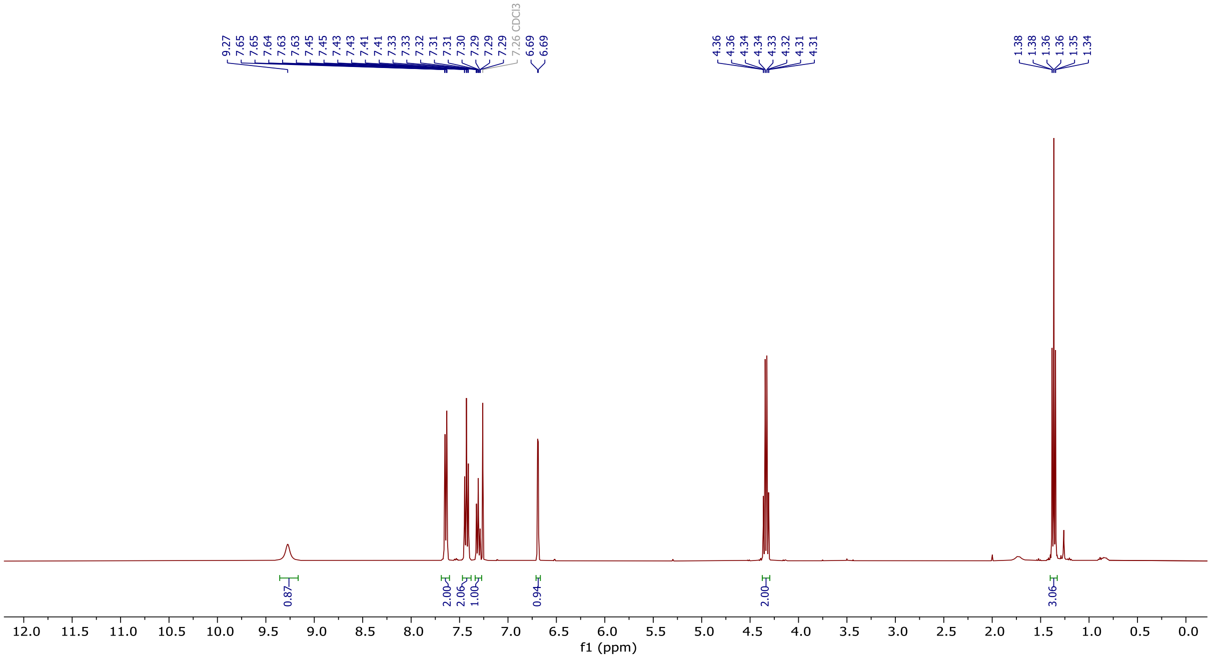

^{13}C NMR (101 MHz, $CDCl_3$)

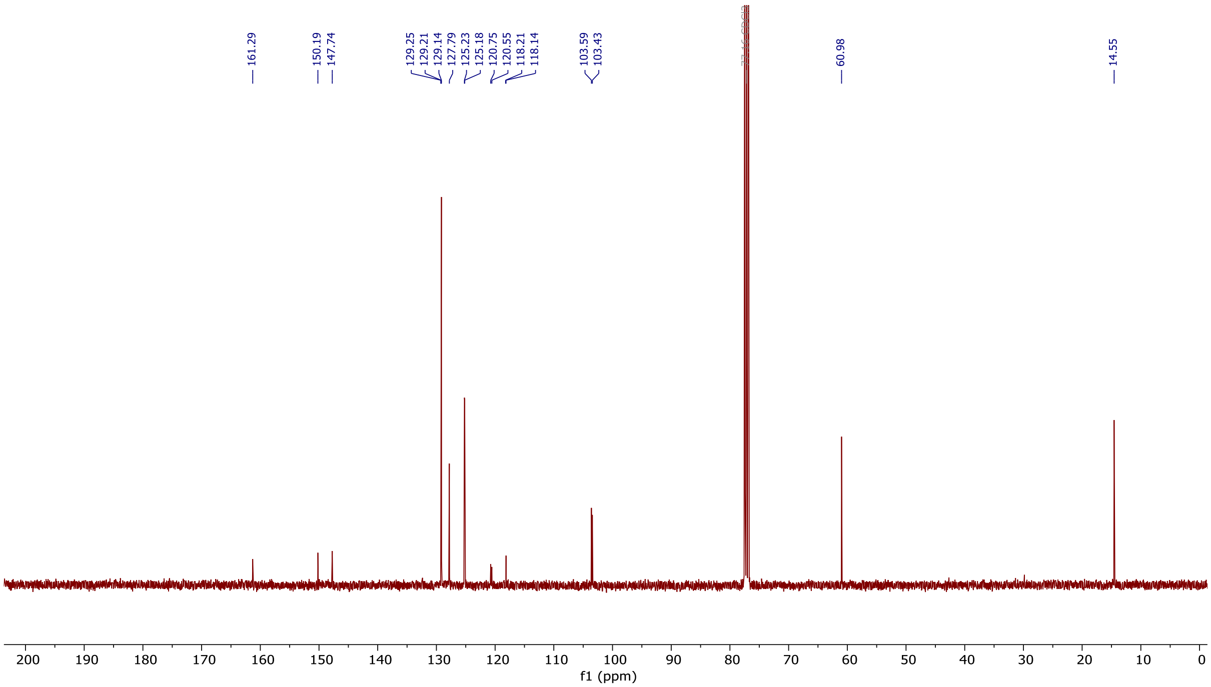

HMBC NMR_Compound **44**

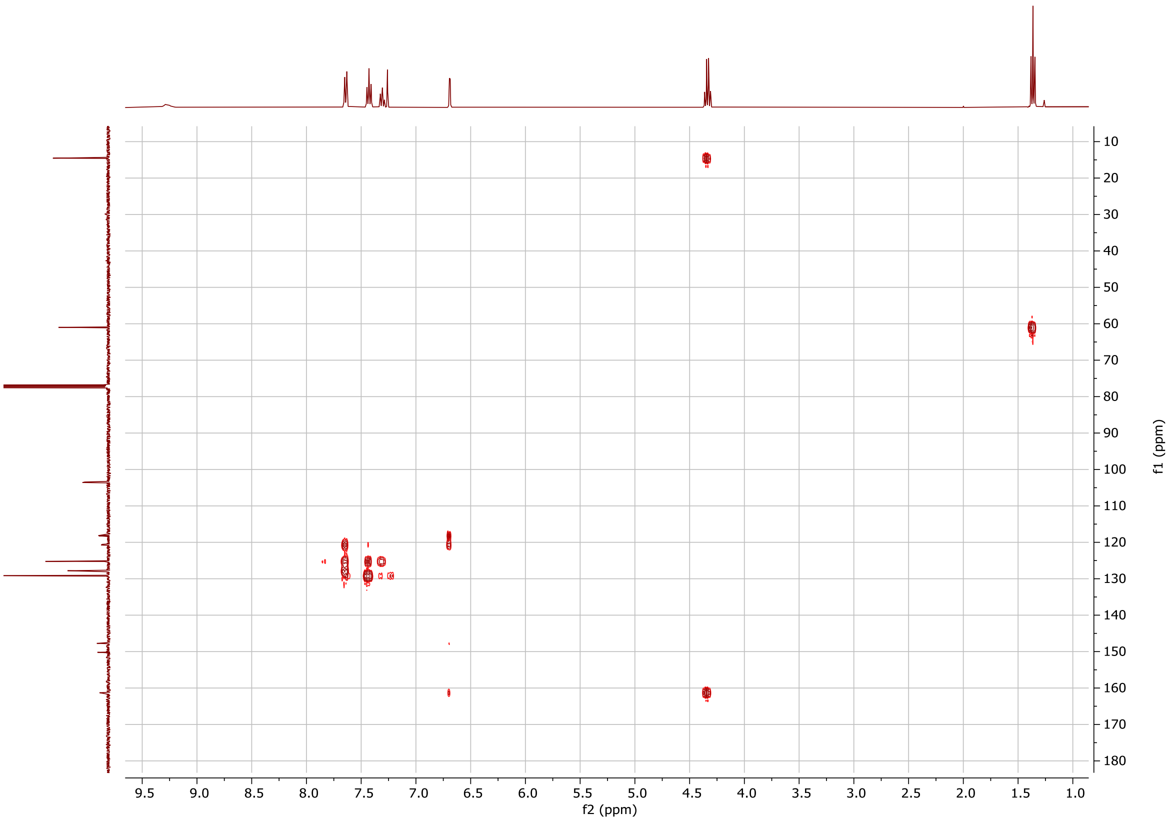

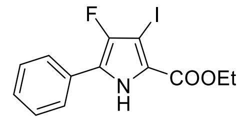

Compound **46**

^{1}H NMR (600 MHz, $CDCl_3$)

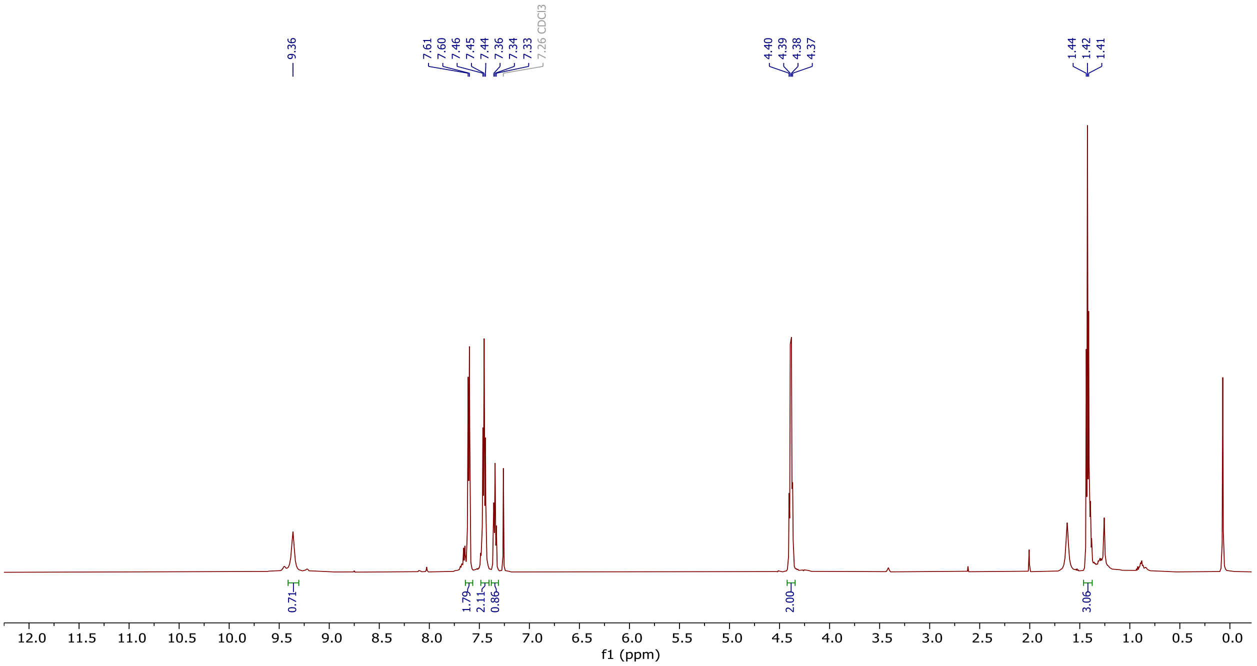

^{13}C NMR (151 MHz, $CDCl_3$)

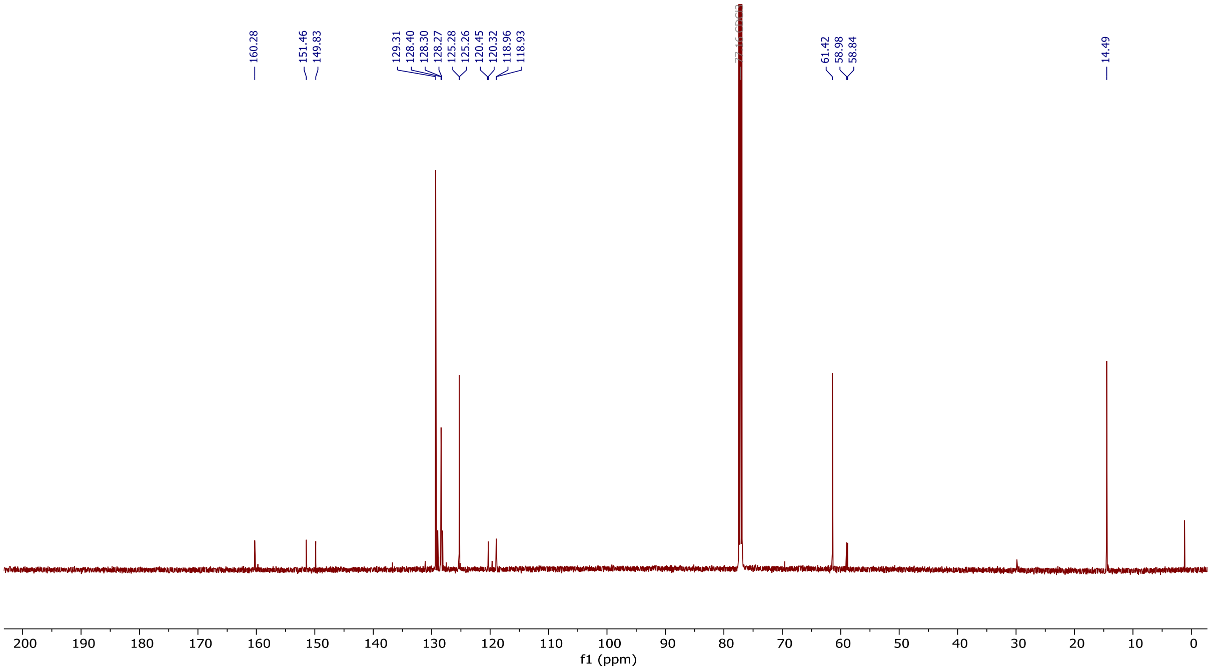

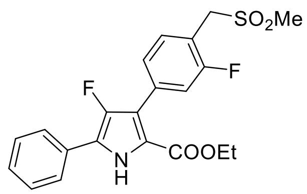

Compound **18a**

^{1}H NMR (700 MHz, DMSO-d_6)

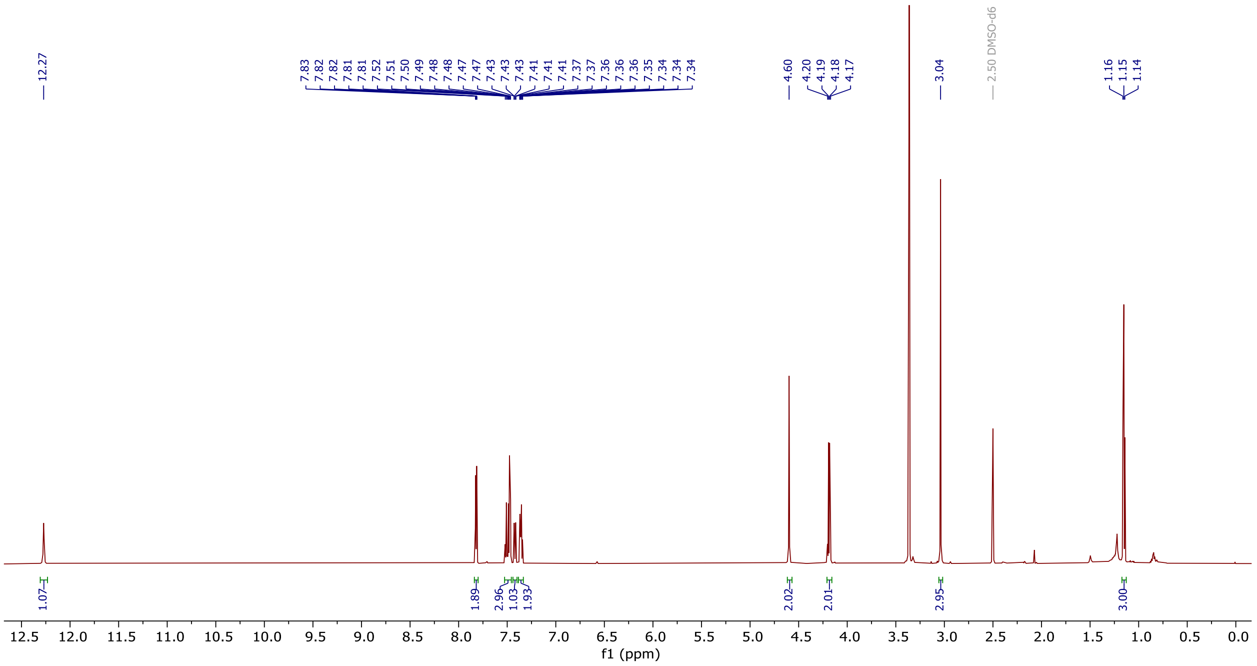

^{13}C NMR (176 MHz, DMSO-d_6)

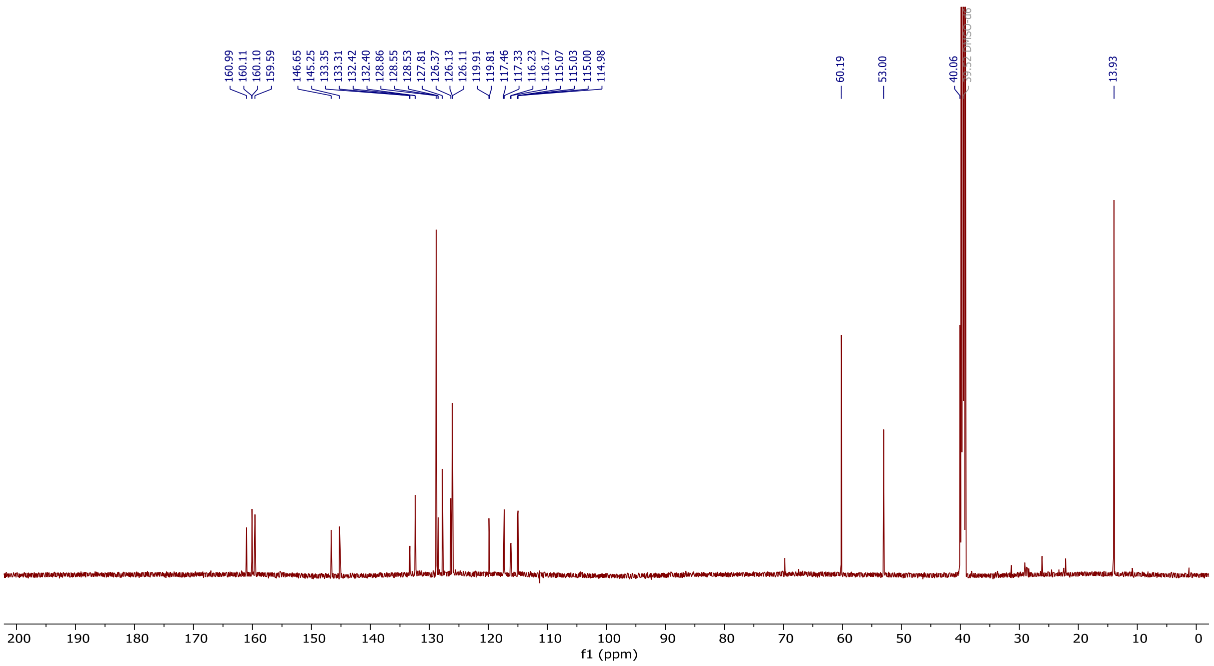

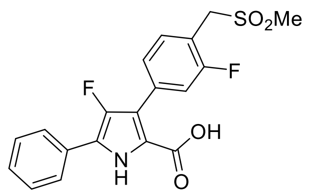

PyC 18

^{1}H NMR (600 MHz, MeOH-d_4)

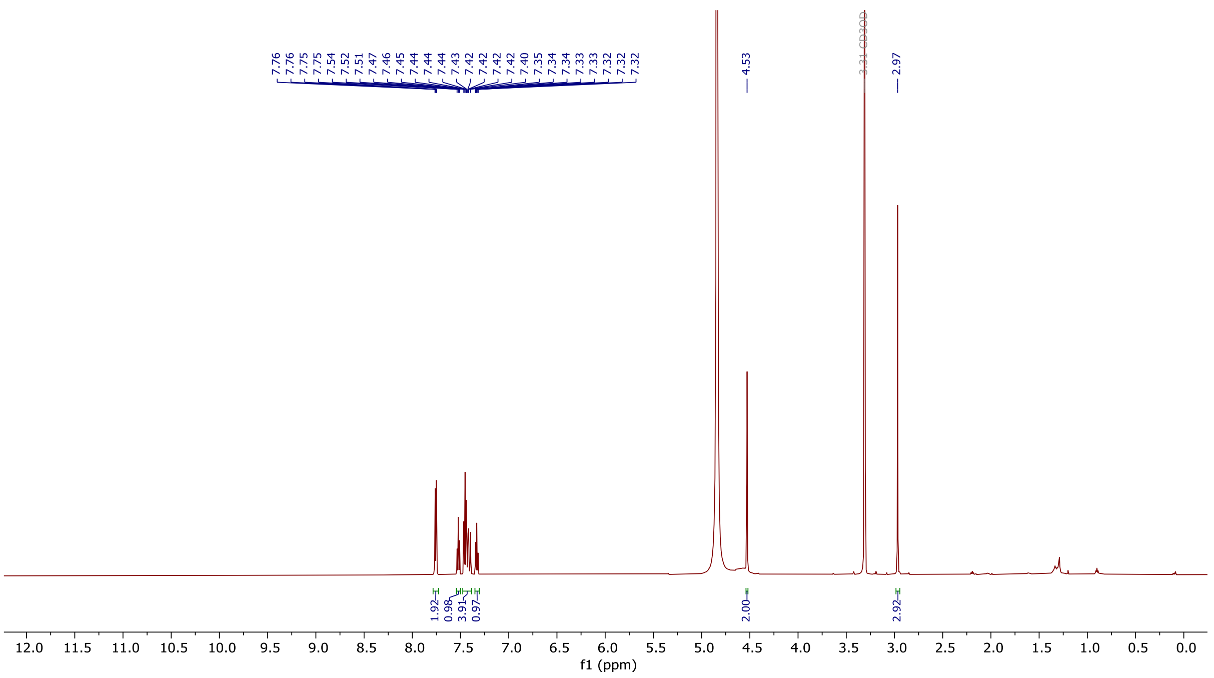

^{13}C NMR (151 MHz, MeOH-d_4)

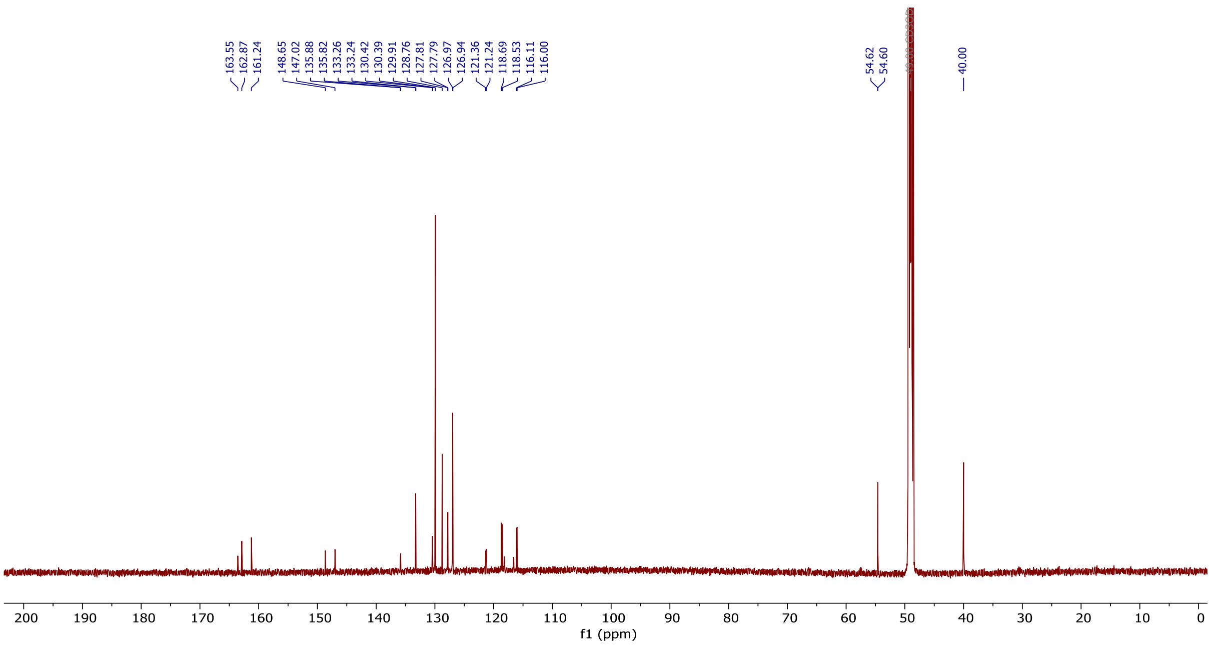

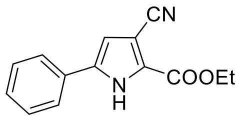

Compound **5a**

^{1}H NMR (500 MHz, $CDCl_3$)

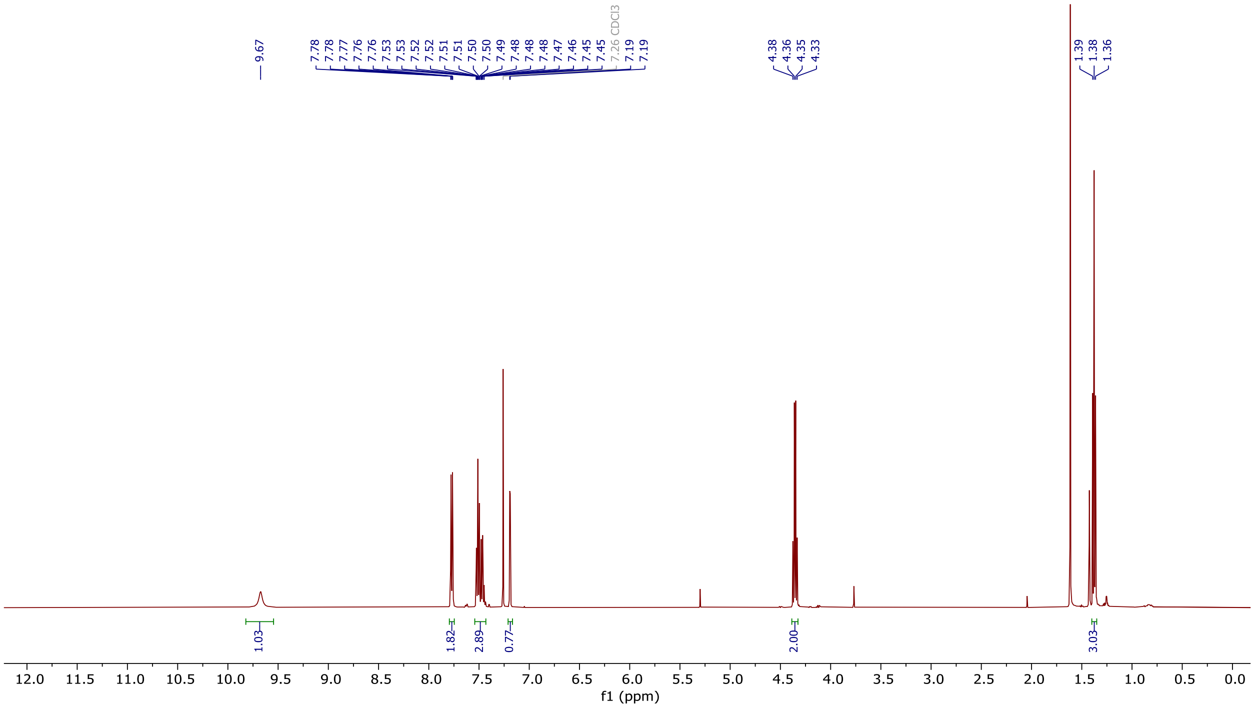

^{13}C NMR (126 MHz, $CDCl_3$)

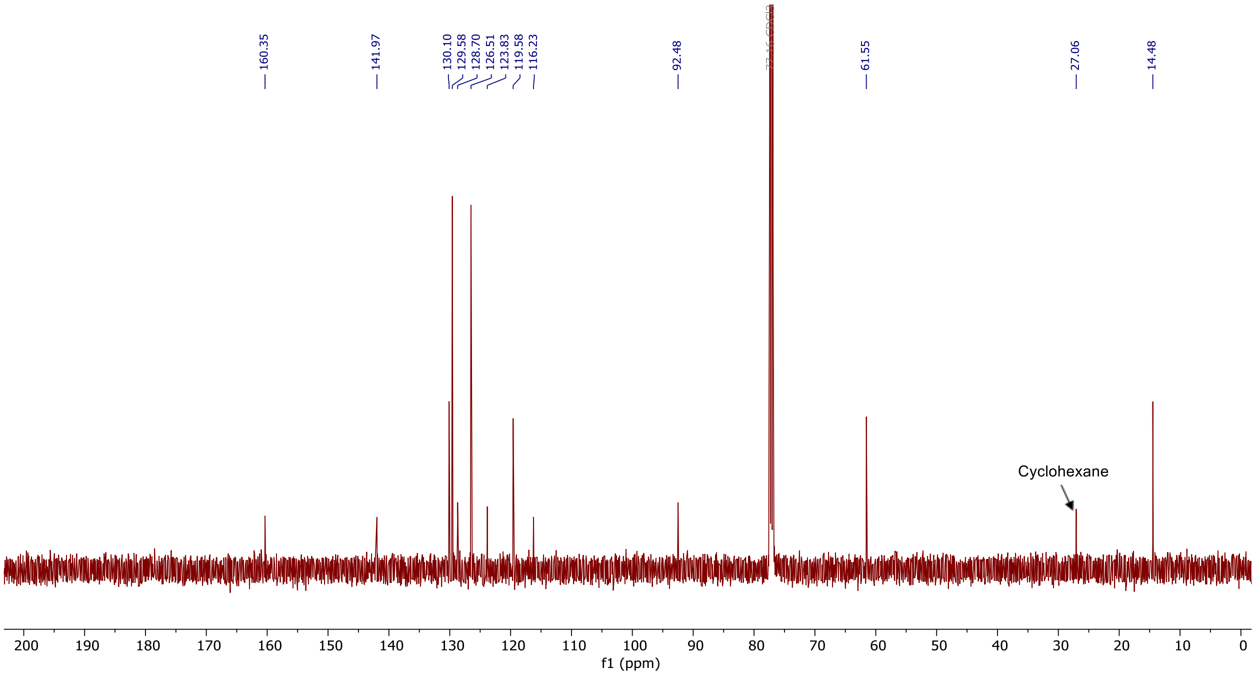

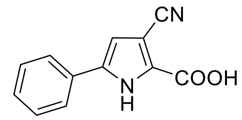

PyC 5

^{1}H NMR (600 MHz, MeOH-d_4)

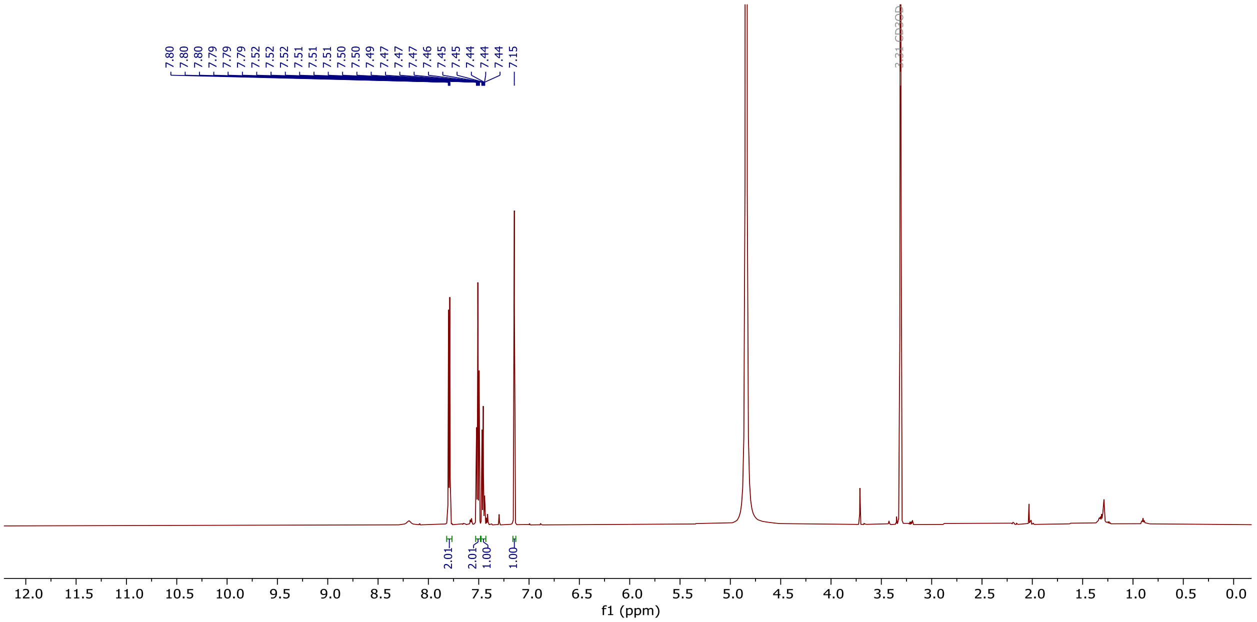

^{13}C NMR (151 MHz, MeOH-d_4)

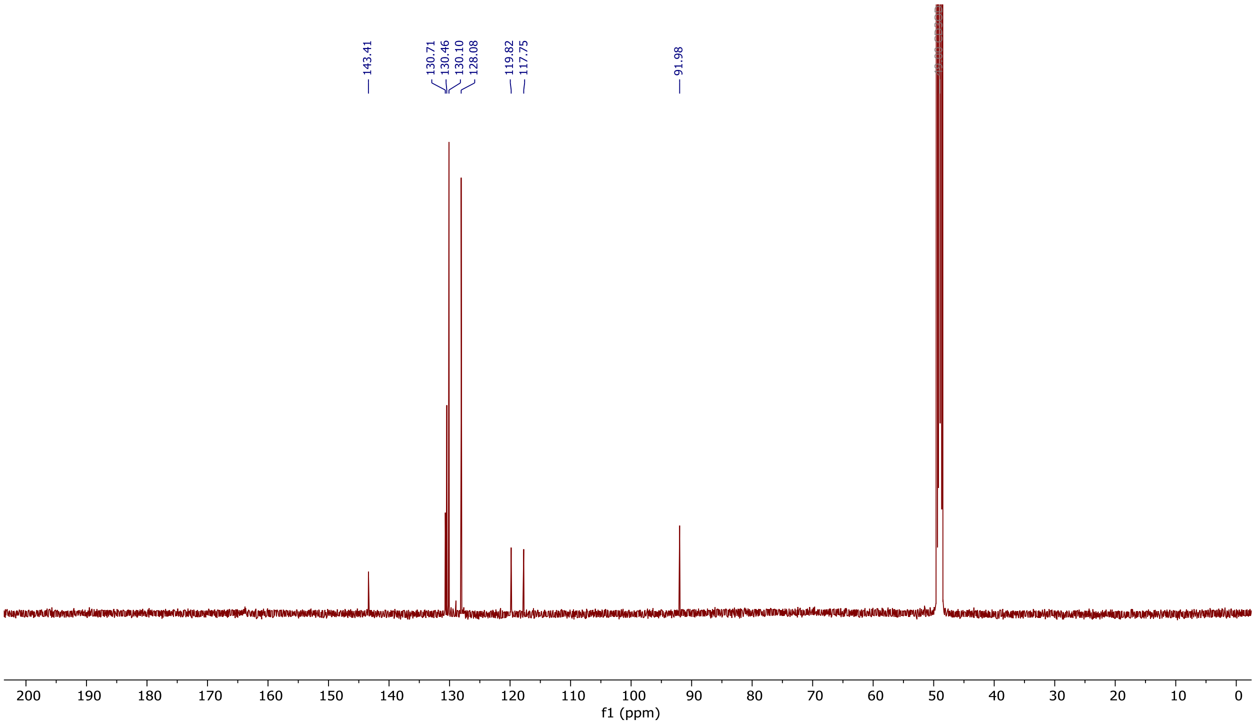

VII. UPLC and HRMS Traces of Final Compounds

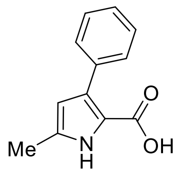

UPLC traces of PyC 1

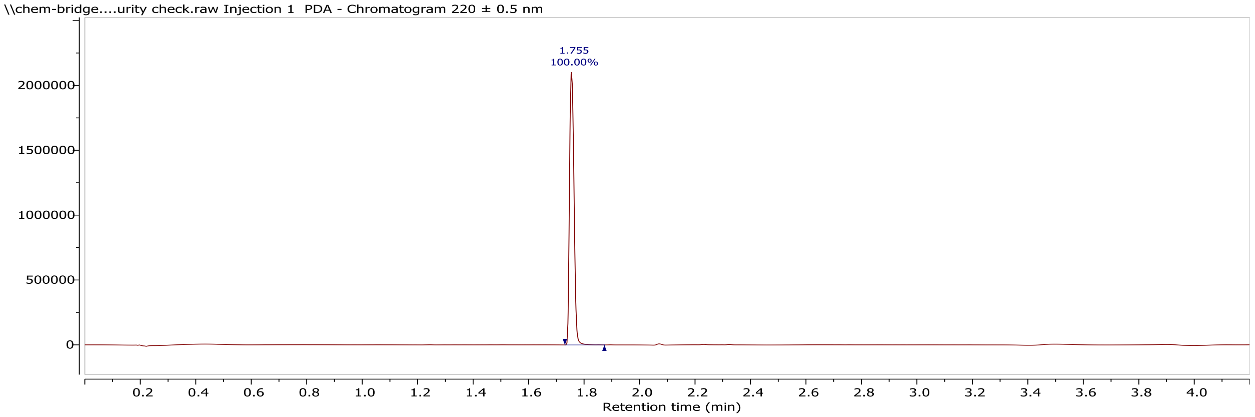

	RT	Scan	Type	Height	Area	Total Height %	Total Area %	Start time	End time
1	1.755	2105	BB	2103331.823	47410780.063	100.00	100.00	1.731	1.873

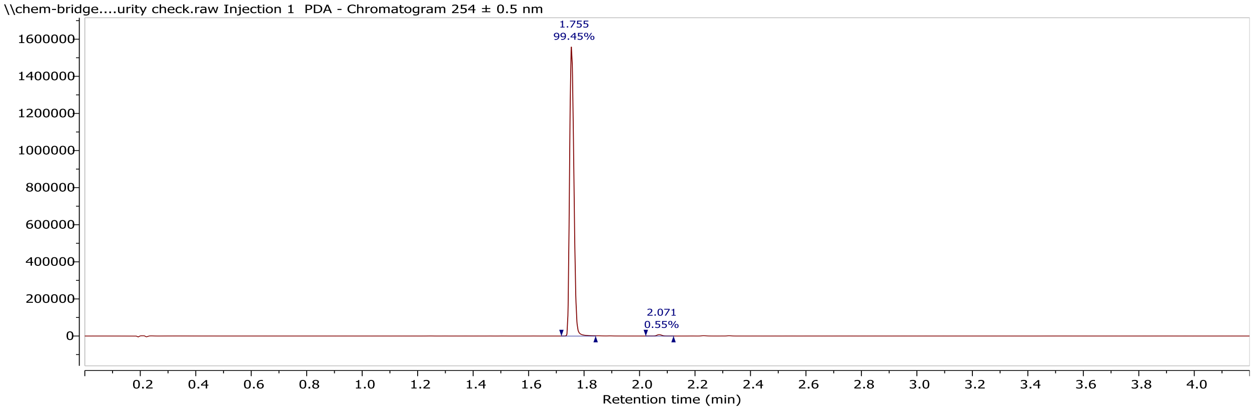

	RT	Scan	Type	Height	Area	Total Height %	Total Area %	Start time	End time
1	2.071	2485	BB	7728.865	185699.984	0.49	0.55	2.023	2.123
2	1.755	2105	BV	1558745.626	33509505.181	99.51	99.45	1.718	1.842

HRMS trace of PyC 1

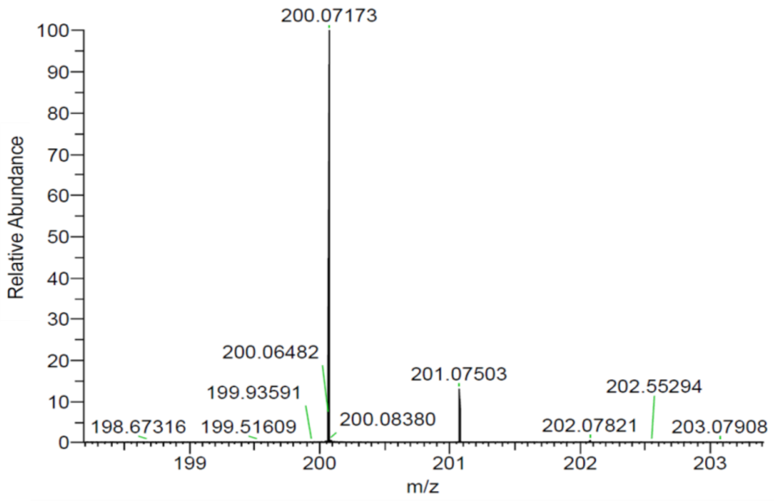

m/z	Formula	RDB	Delta ppm	Theo. Mass
200.07173	$C_{12}H_{10}O_2N$	8.5	0.15	200.07170

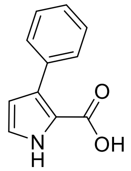

UPLC traces of PyC 2

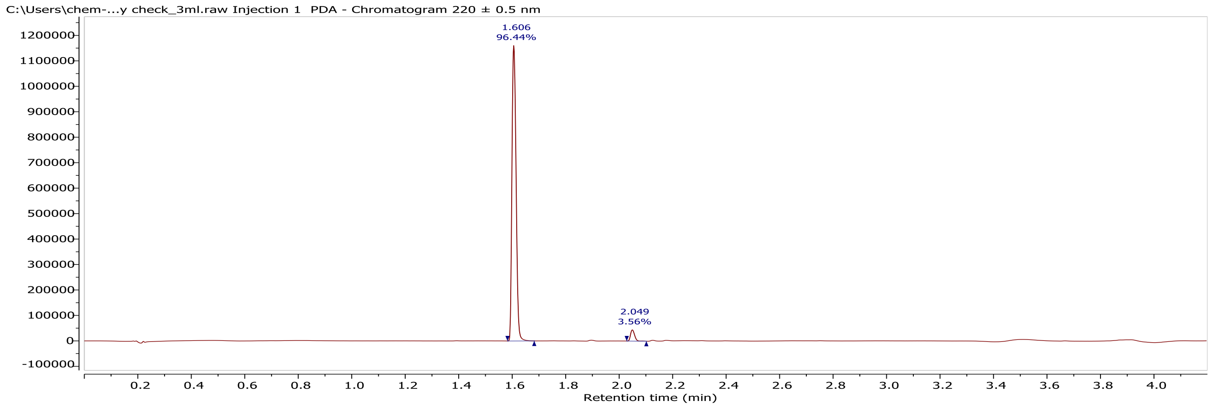

	RT	Scan	Type	Height	Area	Total Height %	Total Area %	Start time	End time
1	1.606	1927	BB	1160781.136	24943286.518	96.33	96.44	1.583	1.683
2	2.049	2459	BB	44225.343	920493.274	3.67	3.56	2.028	2.102

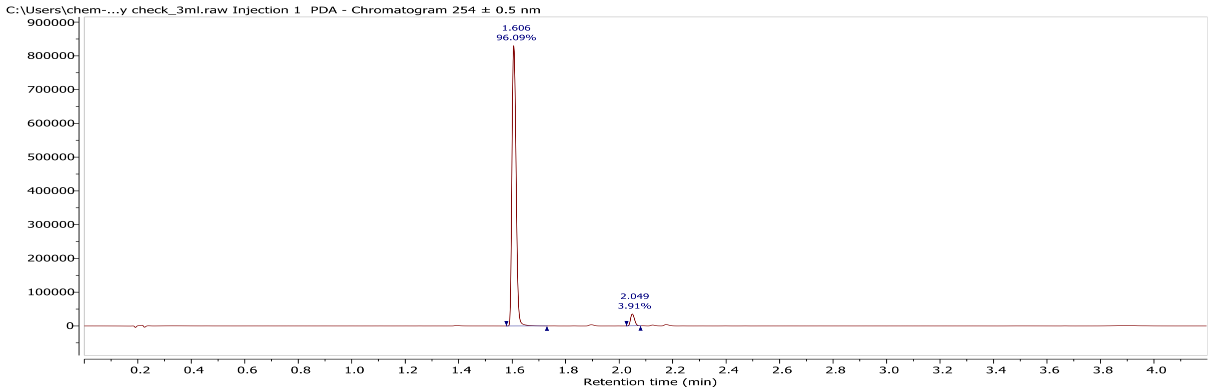

	RT	Scan	Type	Height	Area	Total Height %	Total Area %	Start time	End time
1	1.606	1927	BB	830723.324	17819887.073	95.91	96.09	1.578	1.730
2	2.049	2459	BB	35406.095	725809.000	4.09	3.91	2.028	2.080

HRMS trace of PyC 2

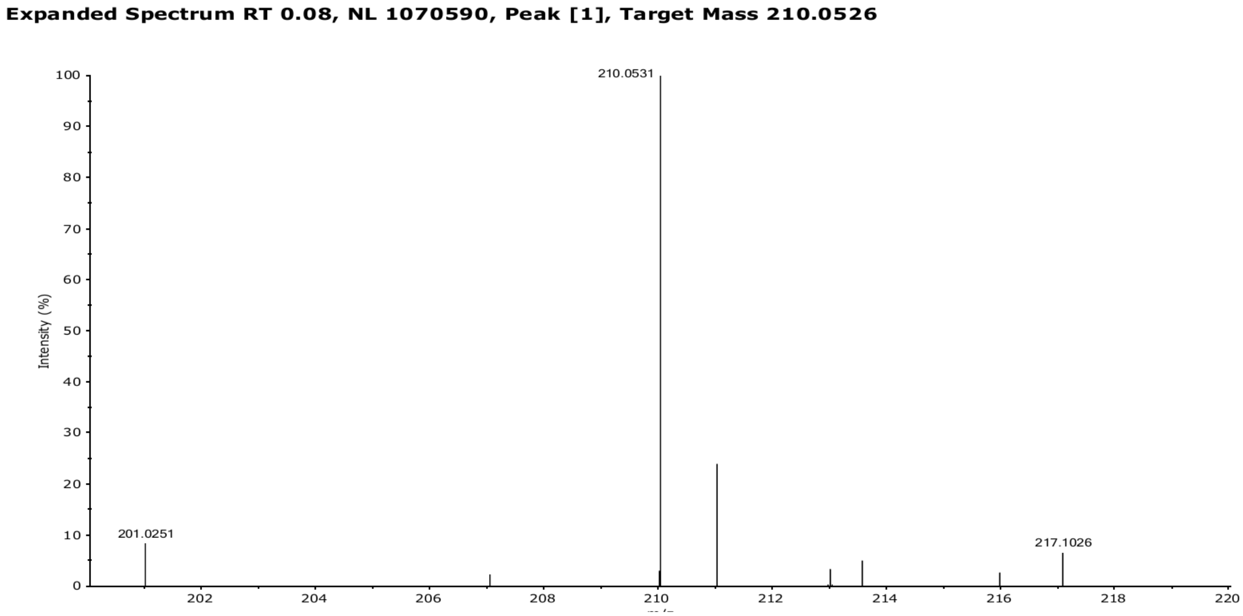

Measured Mass	Calculated Mass	Error (mDa)	Error (ppm)	Formula $[M+Na]^+$	Response
210.0531	210.0526	0.55	2.62	$C_{11}H_9NO_2Na$	28938

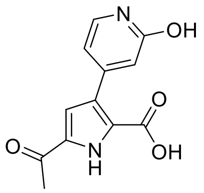

UPLC traces of PyC 3

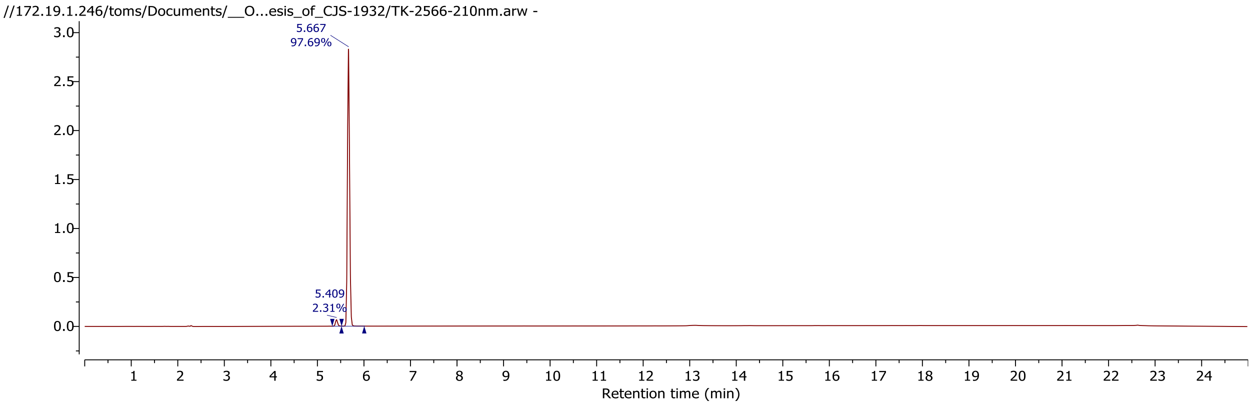

	RT	Height	Area	Total Height %	Total Area %	Start time	End time
1	5.667	2.832	0.153	97.69	97.69	5.520	6.007
2	5.409	0.067	0.004	2.31	2.31	5.320	5.518

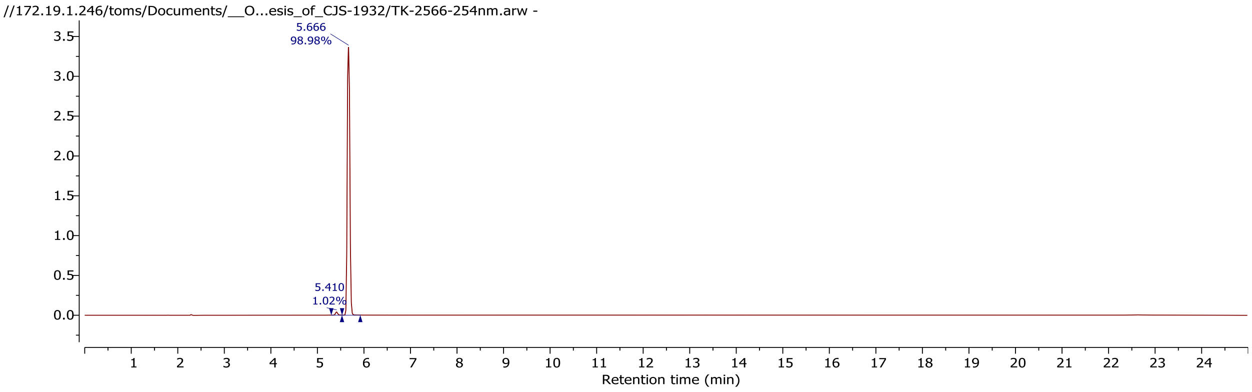

	RT	Height	Area	Total Height %	Total Area %	Start time	End time
1	5.666	3.364	0.216	98.82	98.98	5.530	5.922
2	5.410	0.040	0.002	1.18	1.02	5.298	5.528

HRMS trace of PyC 3

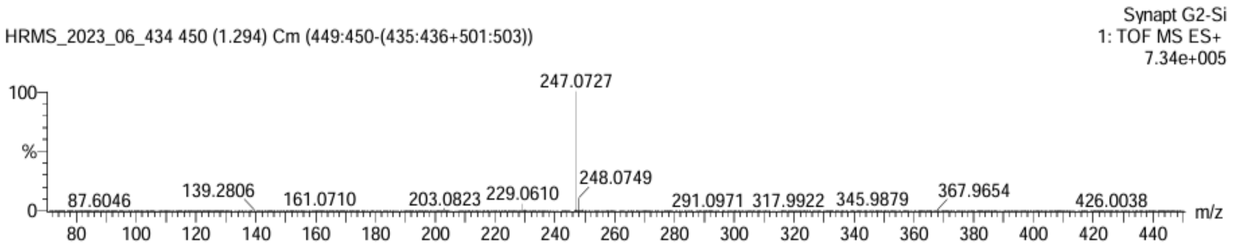

Measured Mass	Calculated Mass	mDa	PPM	DBE	i-FIT	Formula
247.0727	247.0719	0.8	3.2	8.5	335.9	$C_{12}H_{11}N_2O_4$

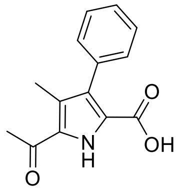

UPLC traces of PyC 4a

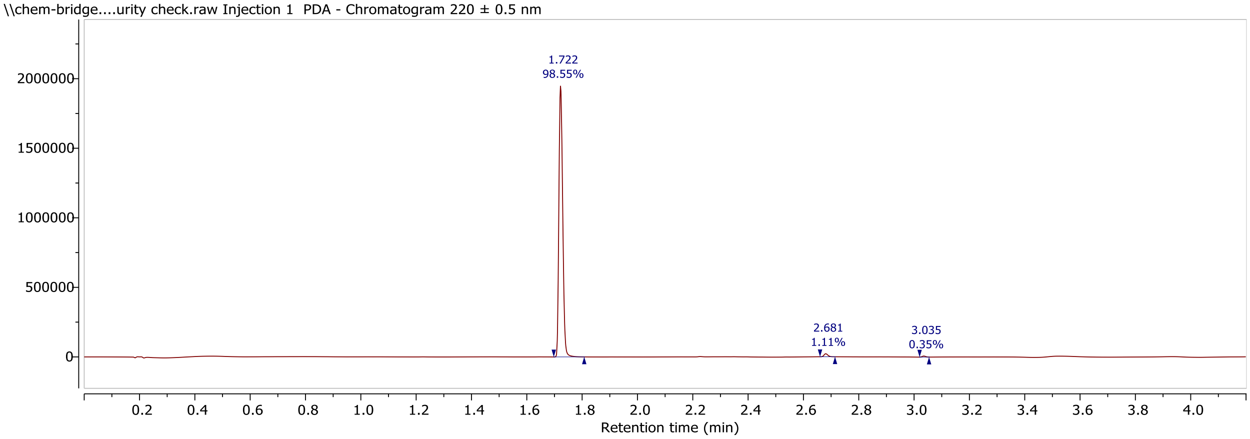

	RT	Scan	Type	Height	Area	Total Height %	Total Area %	Start time	End time
1	1.722	2066	BB	1948459.869	37401332.170	98.51	98.55	1.698	1.808
2	2.681	3217	BB	21959.136	420536.143	1.11	1.11	2.660	2.714
3	3.035	3642	BB	7528.832	131379.256	0.38	0.35	3.020	3.054

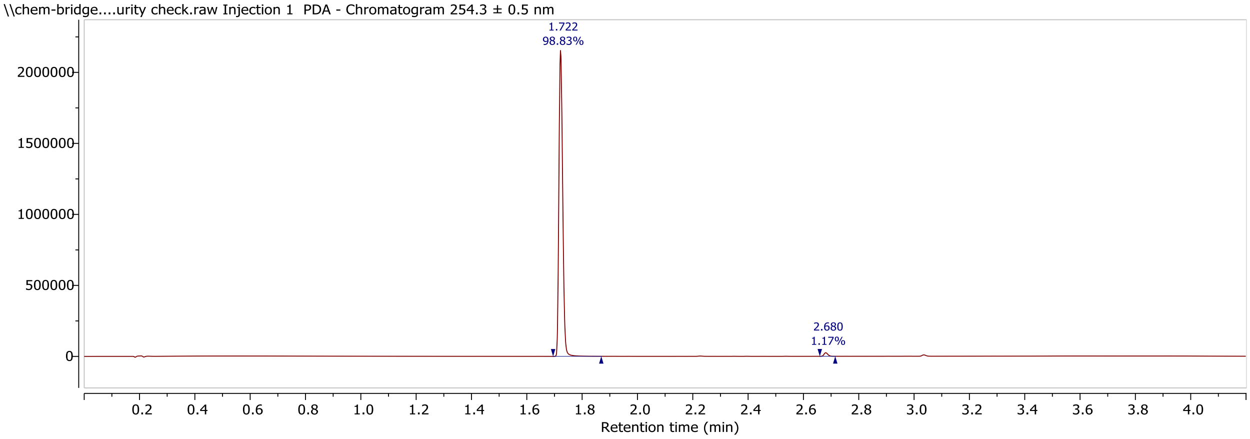

	RT	Scan	Type	Height	Area	Total Height %	Total Area %	Start time	End time
1	1.722	2066	BB	2154915.488	41104242.000	98.44	98.50	1.695	1.869
2	2.680	3217	BB	25656.701	485548.000	1.17	1.16	2.659	2.715
3	3.035	3642	BB	8584.463	142235.024	0.39	0.34	3.025	3.059

HRMS trace of PyC 4a

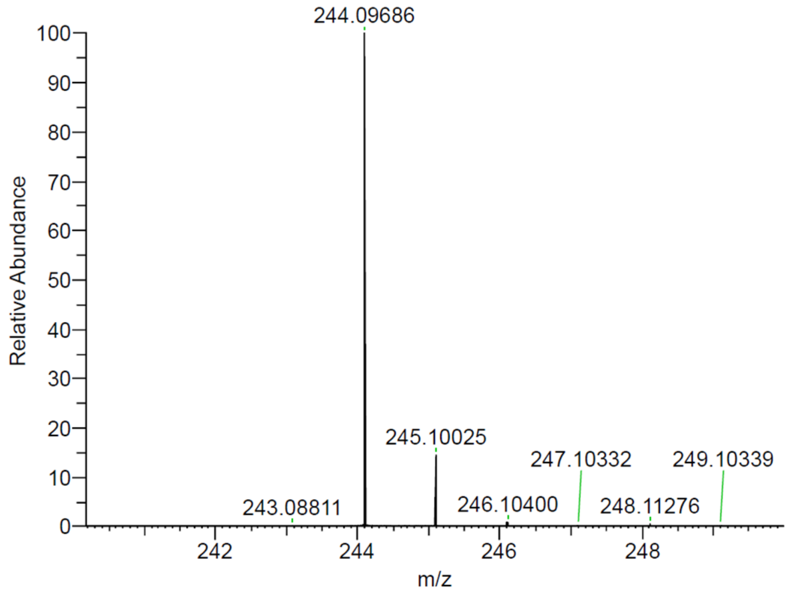

m/z	Formula	RDB	Delta ppm	Theo. Mass
244.09686	$C_{14}H_{14}O_3N$	8.5	0.18	244.09682

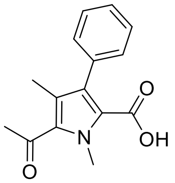

UPLC traces of PyC 4b

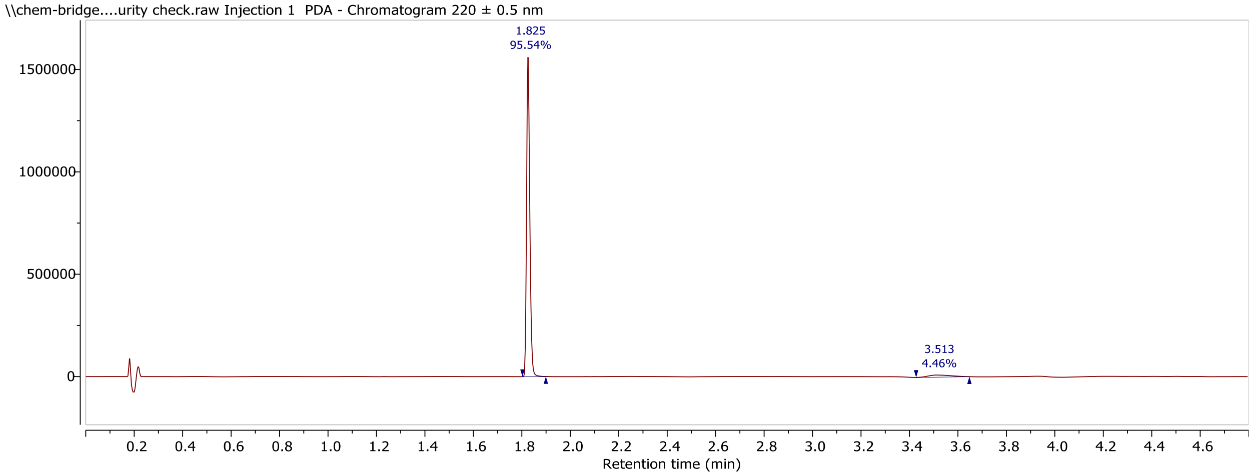

	RT	Scan	Type	Height	Area	Total Height %	Total Area %	Start time	End time
1	3.513	4215	BB	10261.149	1342487.448	0.65	4.46	3.427	3.647
2	1.825	2190	BB	1560325.123	28769400.953	99.35	95.54	1.803	1.899

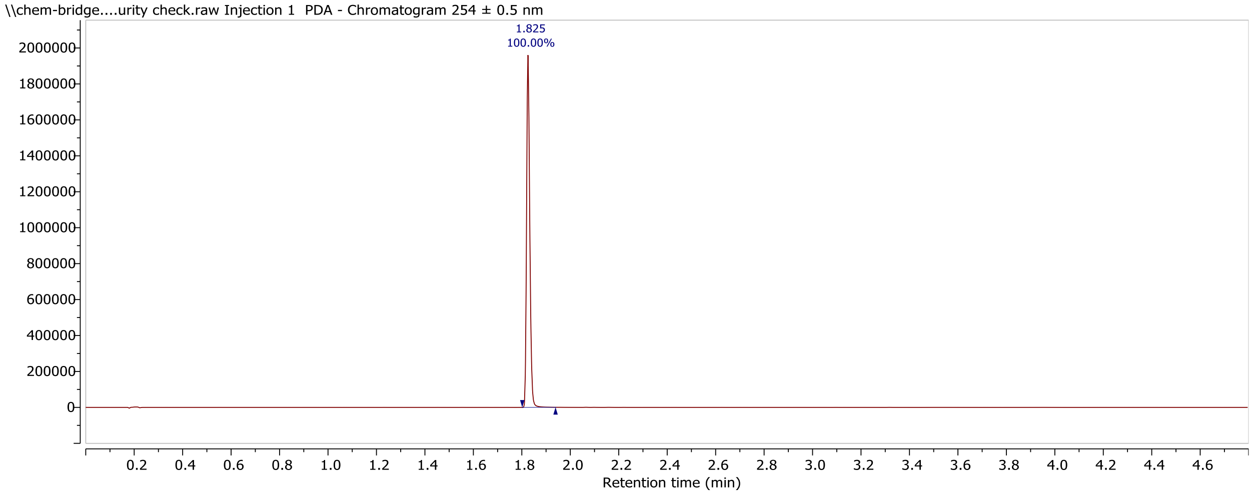

	RT	Scan	Type	Height	Area	Total Height %	Total Area %	Start time	End time
1	1.825	2190	BB	1960356.273	36152983.000	100.00	100.00	1.802	1.939

HRMS trace of PyC 4b

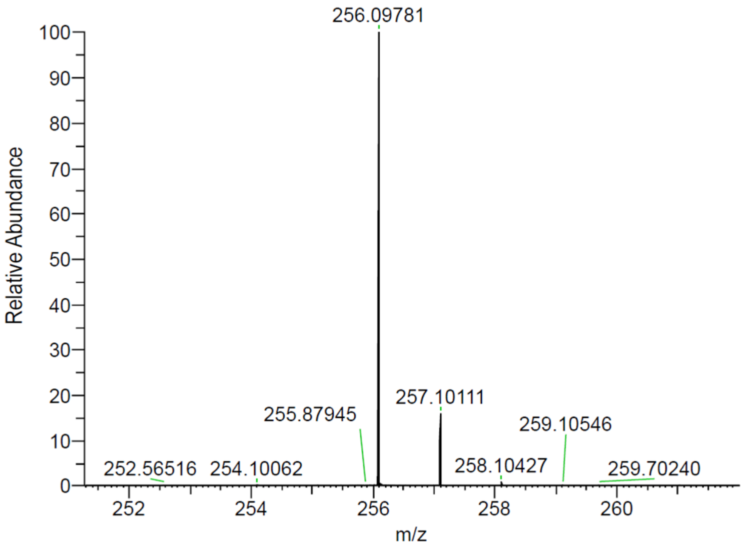

m/z	Formula	RDB	Delta ppm	Theo. Mass
256.09781	$C_{15}H_{14}O_3N$	9.5	-0.4	256.09792

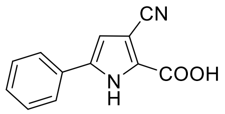

UPLC traces of PyC 5

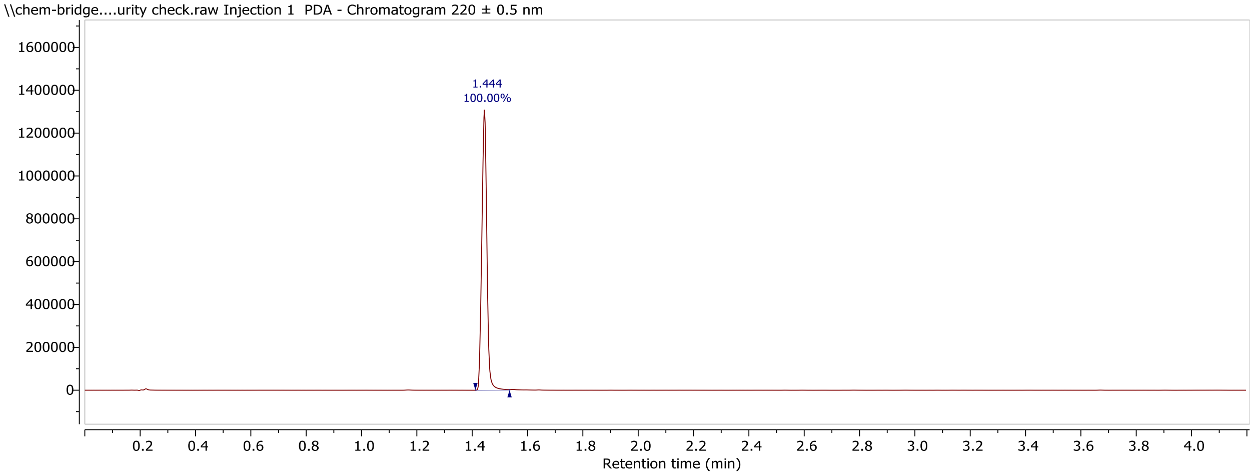

	RT	Scan	Type	Height	Area	Total Height %	Total Area %	Start time	End time
1	1.444	1733	BV	1308917.850	33030881.424	100.00	100.00	1.412	1.535

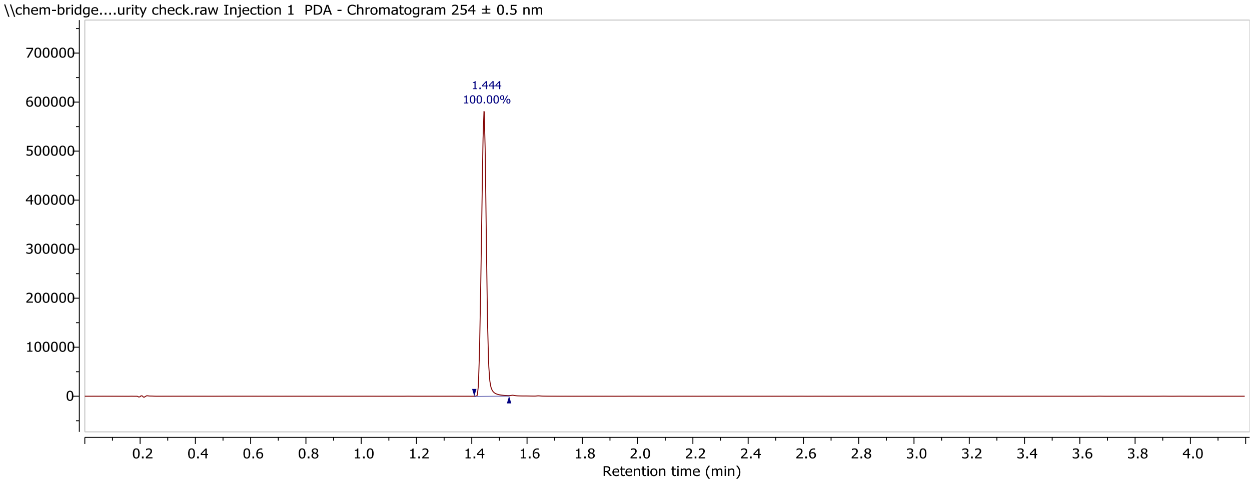

	RT	Scan	Type	Height	Area	Total Height %	Total Area %	Start time	End time
1	1.444	1733	BV	581081.519	14573795.366	100.00	100.00	1.409	1.535

HRMS trace of PyC 5

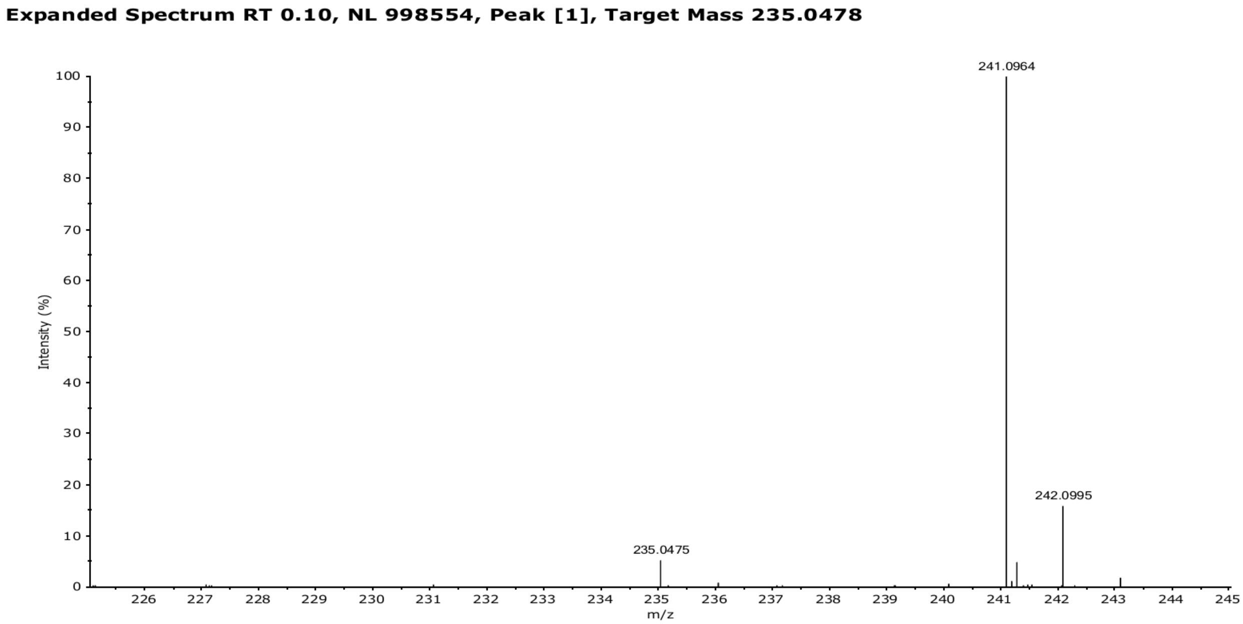

Measured Mass	Calculated Mass	Error (mDa)	Error (ppm)	Formula $[M+Na]^+$	Response
235.0475	235.0478	-0.30	-1.27	$C_{12}H_8N_2O_2Na$	156390

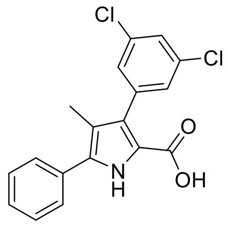

UPLC traces of PyC 6

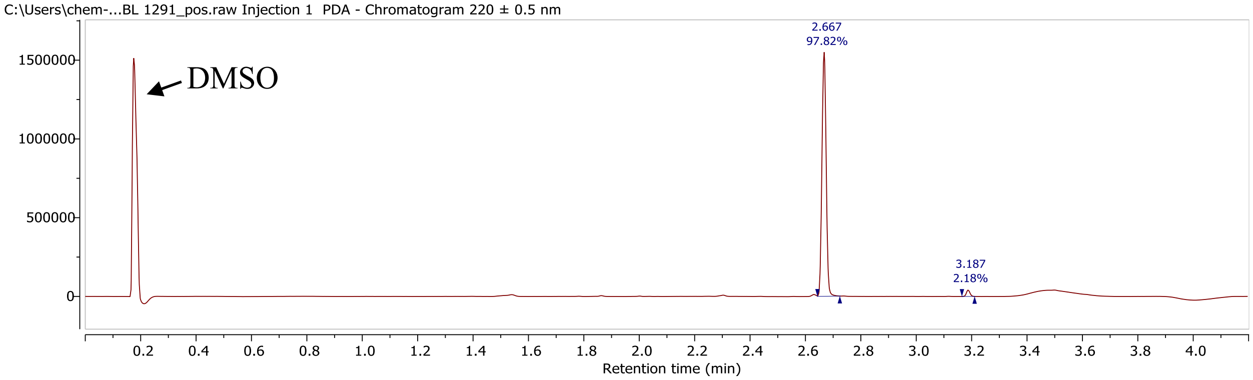

	RT	Scan	Type	Height	Area	Total Height %	Total Area %	Start time	End time
1	3.187	3825	BB	41017.236	730020.000	2.58	2.18	3.165	3.211
2	2.667	3201	VB	1549444.056	32822504.703	97.42	97.82	2.643	2.724

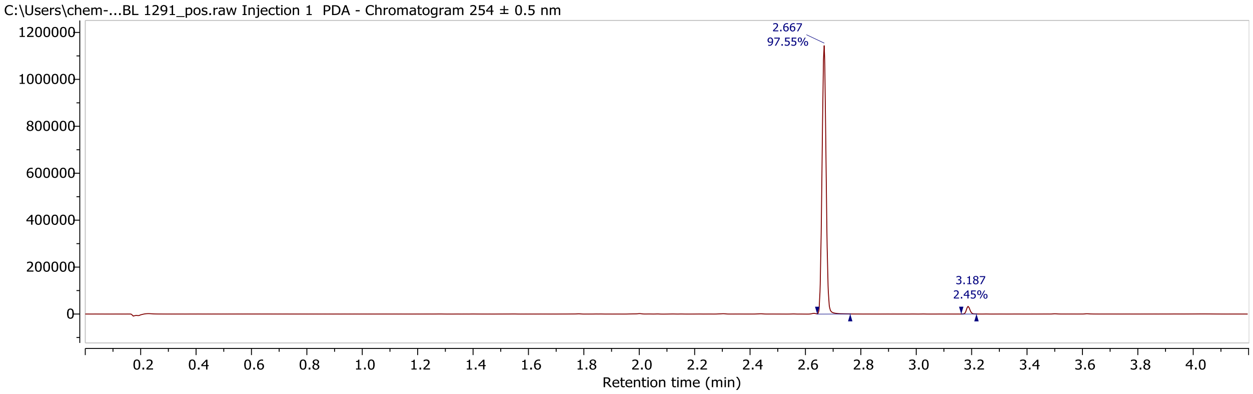

	RT	Scan	Type	Height	Area	Total Height %	Total Area %	Start time	End time
1	3.187	3825	BB	32477.727	582864.500	2.76	2.45	3.162	3.217
2	2.667	3201	VB	1144403.679	23249757.349	97.24	97.55	2.643	2.762

HRMS trace of PyC 6

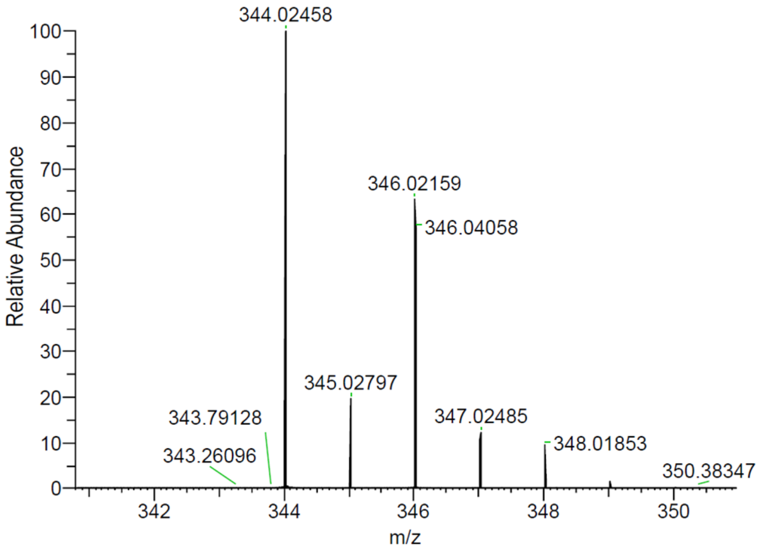

m/z	Formula	RDB	Delta ppm	Theo. Mass
344.02460	$C_{18}H_{12}O_2N^{35}Cl_2$	12.5	-1.34	344.02506

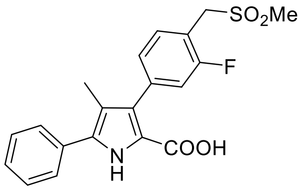

UPLC traces of PyC 7

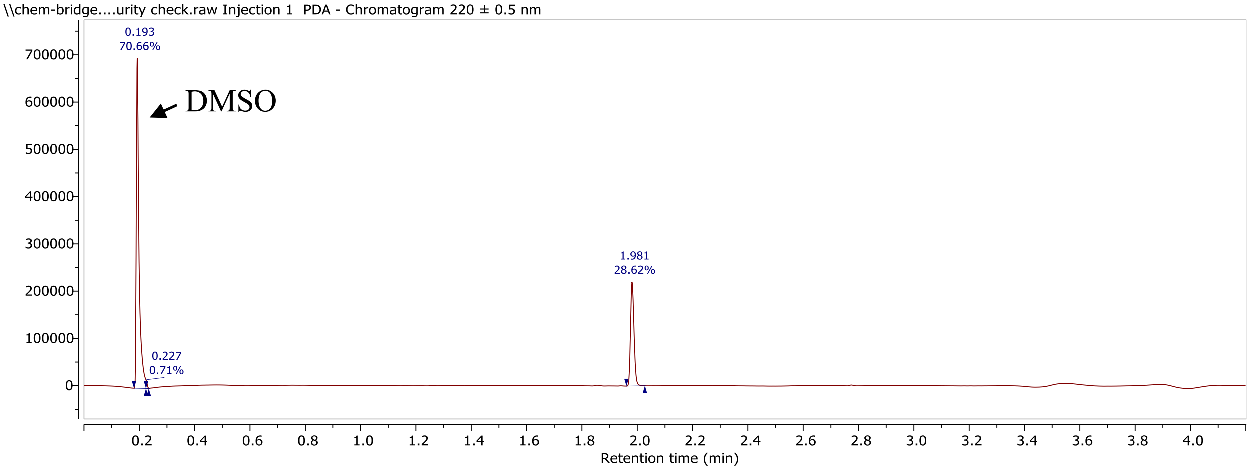

	RT	Scan	Type	Height	Area	Total Height %	Total Area %	Start time	End time
1	0.227	272	VB	13990.594	96127.750	1.50	0.71	0.224	0.234
2	0.193	231	BV	698975.188	9520532.250	74.90	70.66	0.181	0.224
3	1.981	2377	BB	220300.205	3856588.704	23.61	28.62	1.961	2.028

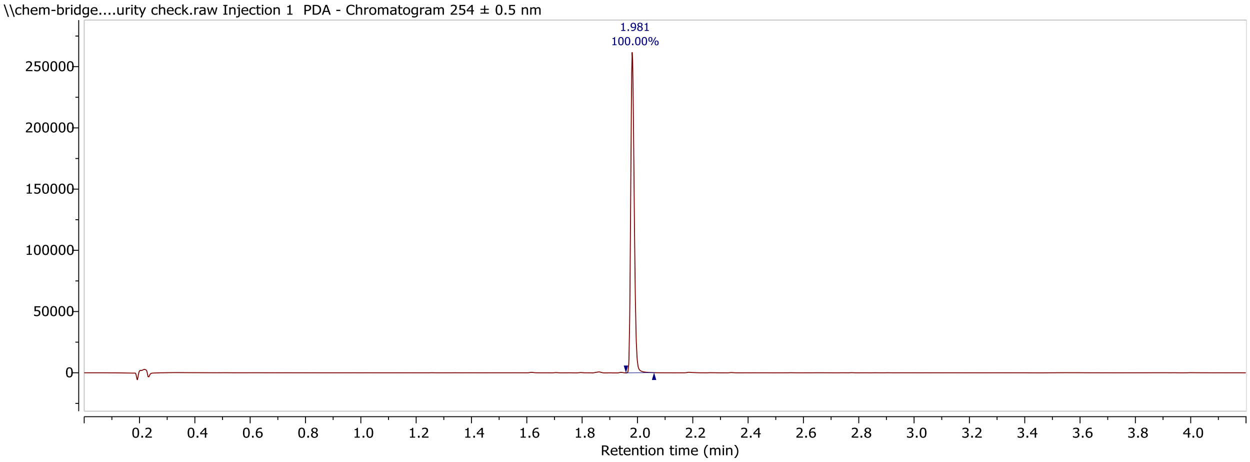

	RT	Scan	Type	Height	Area	Total Height %	Total Area %	Start time	End time
1	1.981	2377	BB	261739.246	4606380.000	100.00	100.00	1.958	2.060

HRMS trace of PyC 7

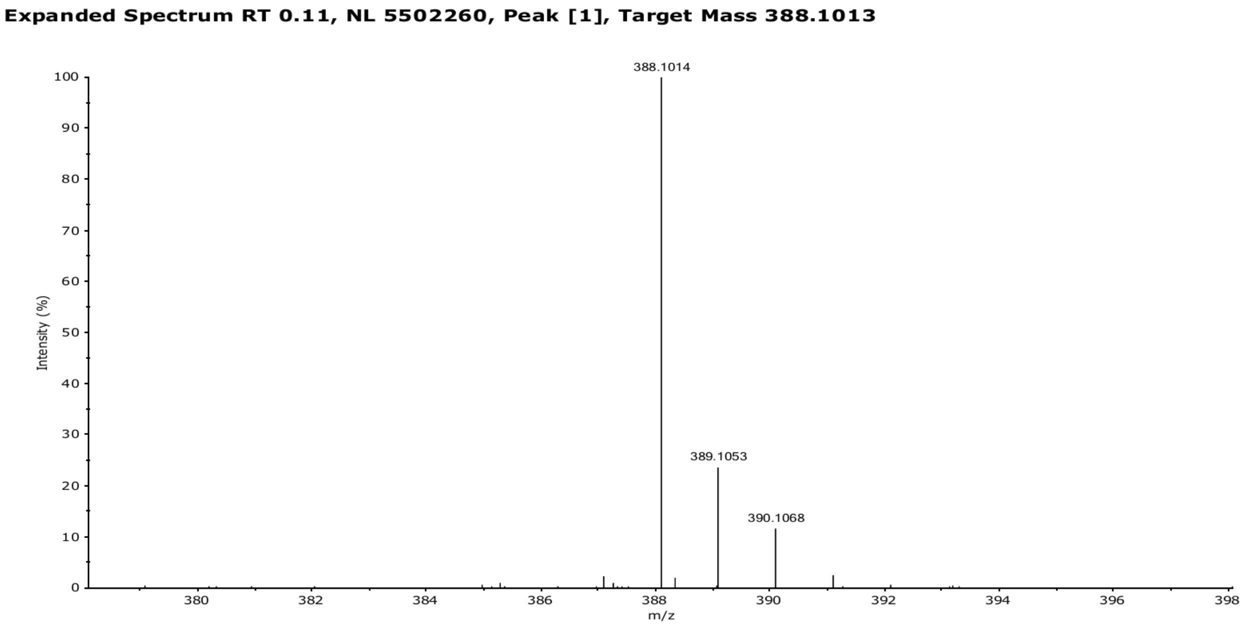

Measured Mass	Calculated Mass	Error (mDa)	Error (ppm)	Formula $[M+H]^+$	Response
388.1014	388.1013	0.06	0.17	$C_{20}H_{19}FNO_4S$	2858708

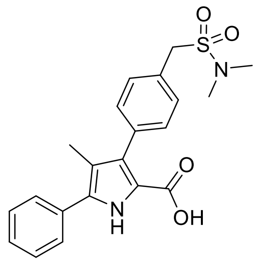

UPLC traces of PyC 8

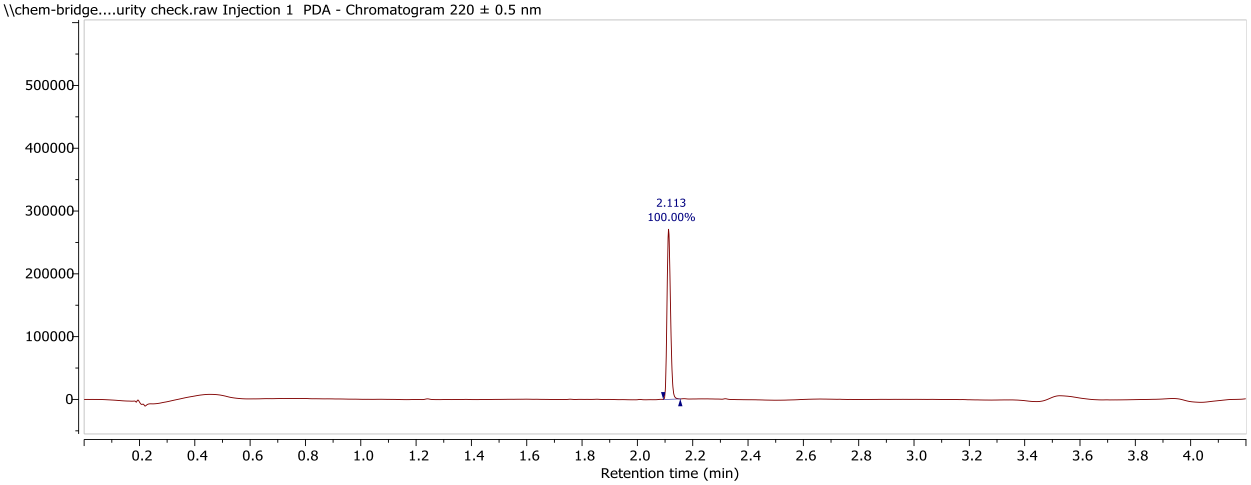

	RT	Scan	Type	Height	Area	Total Height %	Total Area %	Start time	End time
1	2.113	2535	BB	271166.250	4772633.939	100.00	100.00	2.093	2.155

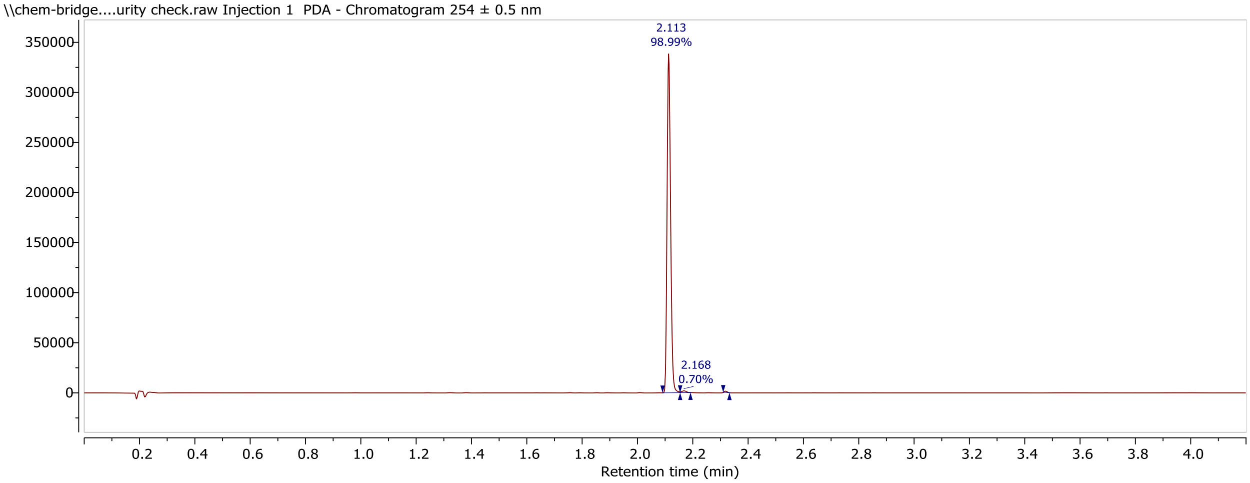

	RT	Scan	Type	Height	Area	Total Height %	Total Area %	Start time	End time
1	2.168	2602	VB	1793.295	42068.992	0.52	0.70	2.154	2.192
2	2.113	2535	BV	338457.841	5979460.164	99.08	98.99	2.091	2.154
3	2.318	2782	BB	1337.037	19092.000	0.39	0.32	2.310	2.333

HRMS trace of PyC 8

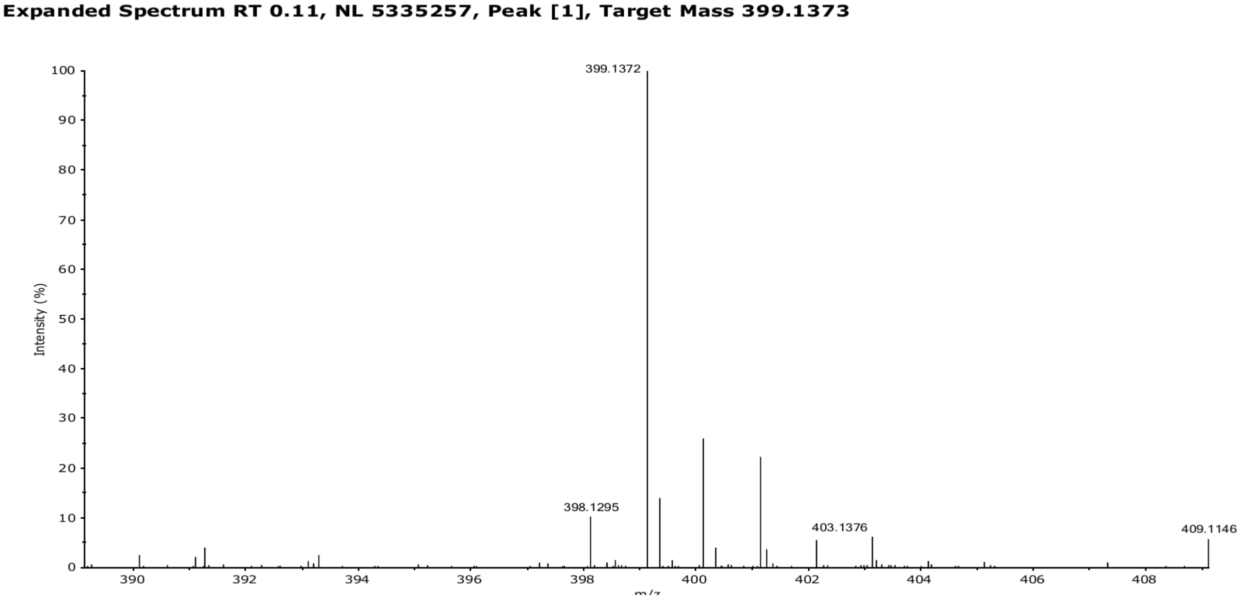

Measured Mass	Calculated Mass	Error (mDa)	Error (ppm)	Formula $[M+H]^+$	Response
399.1372	399.1373	-0.11	-0.27	$C_{21}H_{23}N_2O_4S$	587070

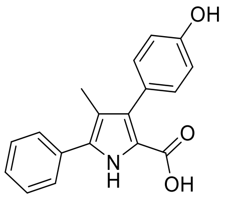

UPLC traces of PyC 9

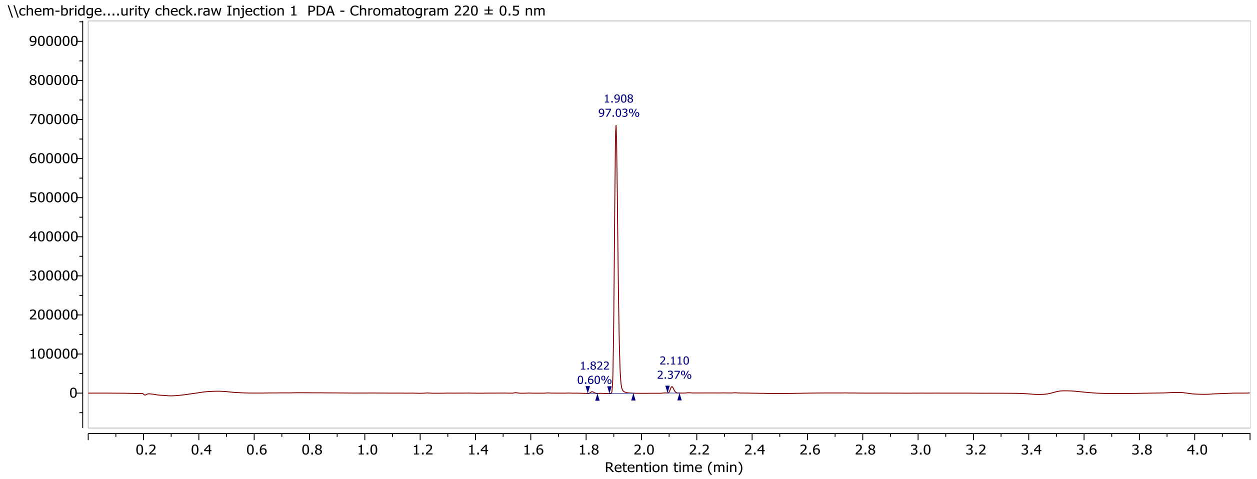

	RT	Scan	Type	Height	Area	Total Height %	Total Area %	Start time	End time
1	1.822	2186	BB	4368.992	74088.244	0.62	0.60	1.806	1.841
2	1.908	2290	BB	686068.265	12027375.319	97.06	97.03	1.884	1.971
3	2.110	2532	BB	16386.428	293485.686	2.32	2.37	2.094	2.138

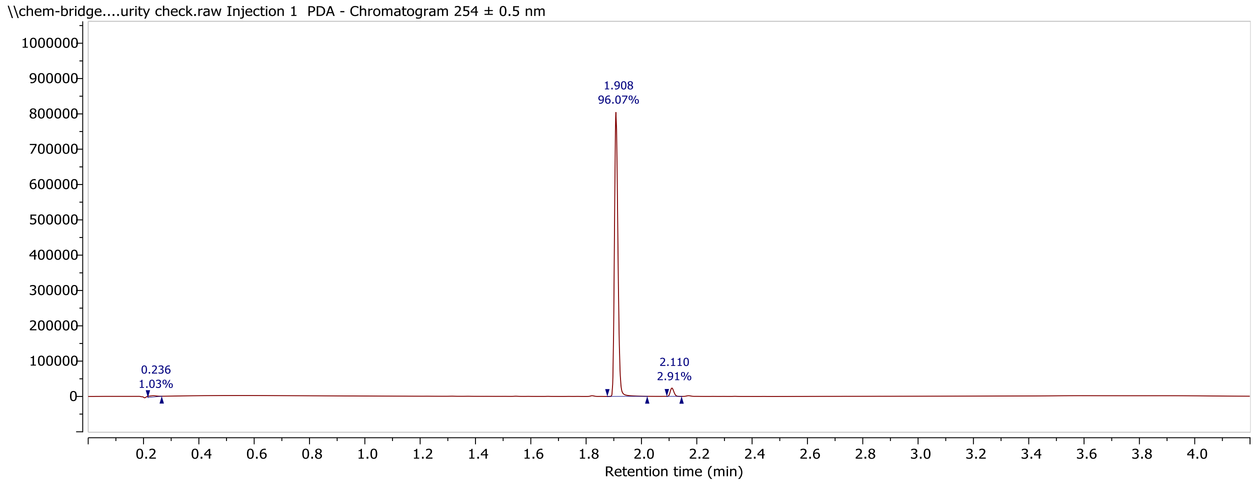

	RT	Scan	Type	Height	Area	Total Height %	Total Area %	Start time	End time
1	0.236	283	VB	3786.919	151029.446	0.46	1.03	0.216	0.266
2	1.908	2290	BB	804491.803	14151794.000	96.70	96.07	1.877	2.021
3	2.110	2532	BB	23643.500	428110.000	2.84	2.91	2.092	2.145

HRMS trace of PyC 9

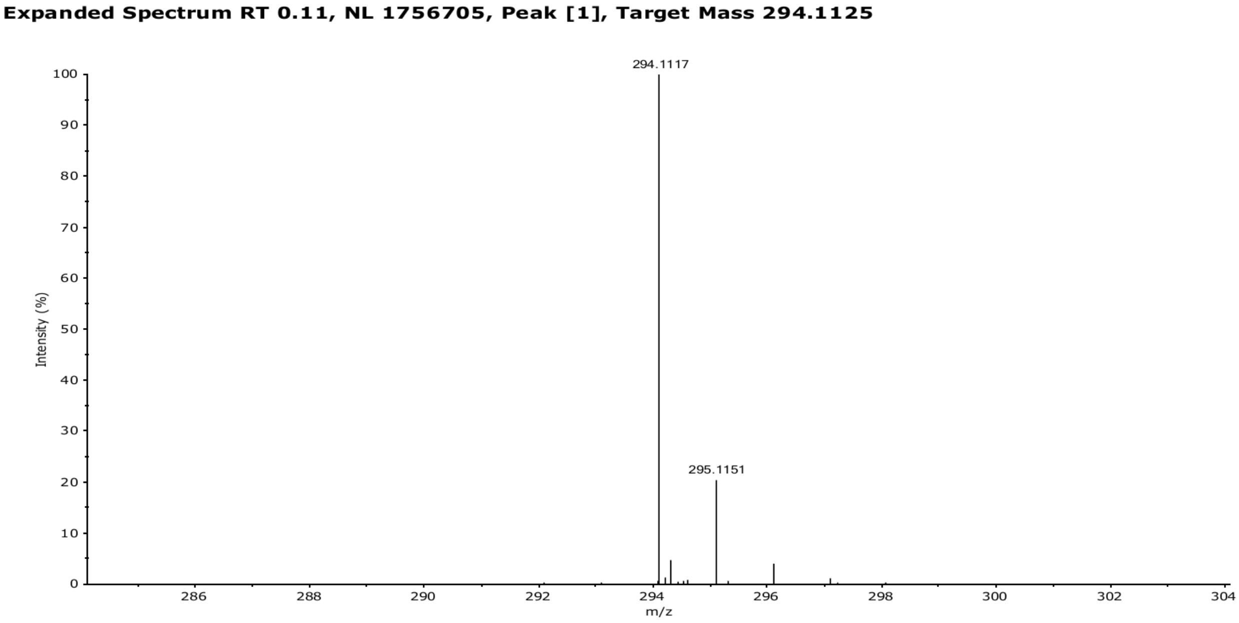

Measured Mass	Calculated Mass	Error (mDa)	Error (ppm)	Formula $[M+H]^+$	Response
294.1117	294.1125	-0.77	-2.62	$C_{18}H_{16}NO_3$	5363359

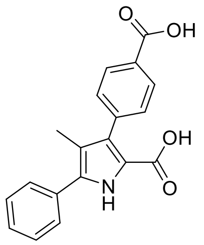

UPLC traces of PyC 10

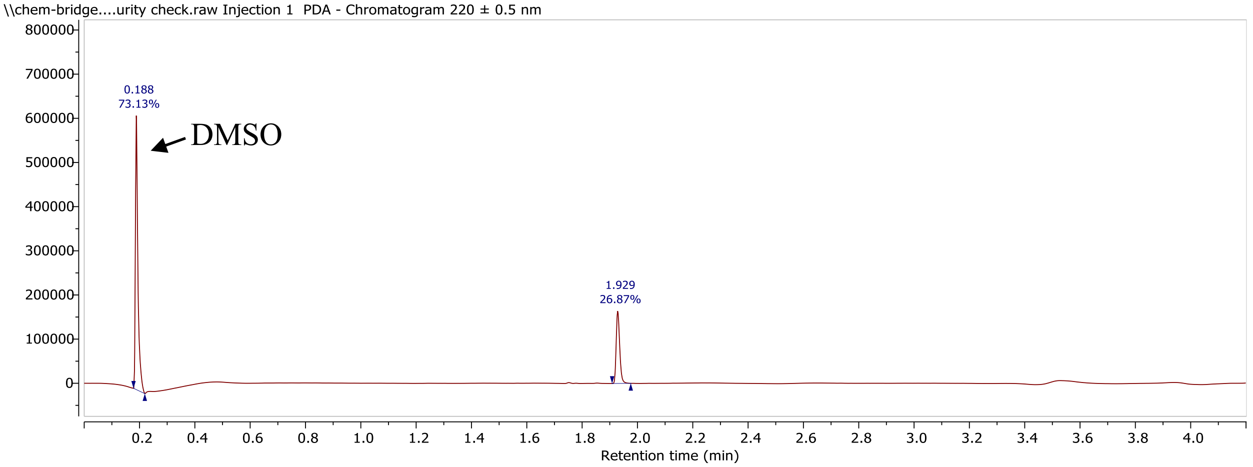

	RT	Scan	Type	Height	Area	Total Height %	Total Area %	Start time	End time
1	0.188	226	BB	620330.184	7838973.000	79.09	73.13	0.178	0.219
2	1.929	2314	BB	163956.054	2879838.674	20.91	26.87	1.908	1.976

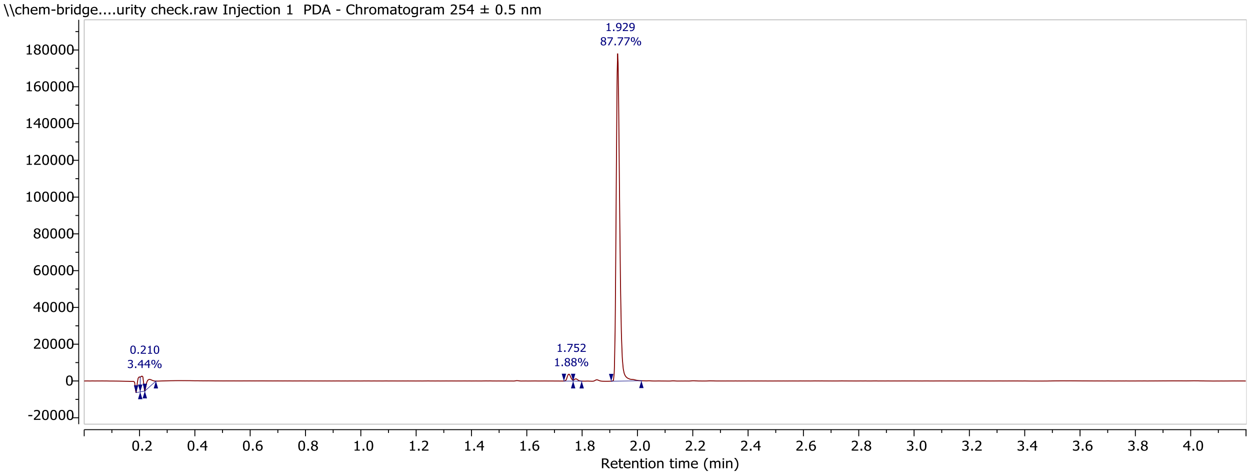

	RT	Scan	Type	Height	Area	Total Height %	Total Area %	Start time	End time
1	1.777	2132	VB	1226.948	21910.325	0.67	0.67	1.768	1.798
2	1.752	2102	BV	3829.273	67961.675	2.09	2.08	1.734	1.768
3	1.929	2314	BB	178150.153	3174257.000	97.24	97.25	1.905	2.014

HRMS trace of PyC 10

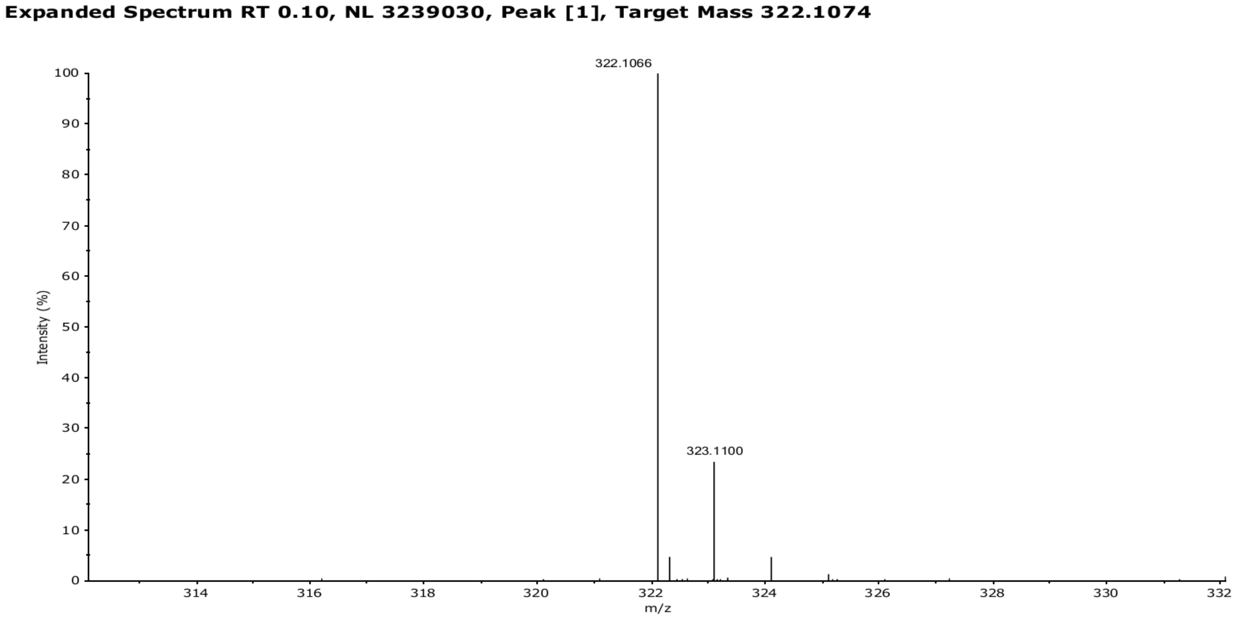

Measured Mass	Calculated Mass	Error (mDa)	Error (ppm)	Formula $[M+H]^+$	Response
322.1066	322.1074	-0.79	-2.44	$C_{19}H_{16}NO_4$	12237139

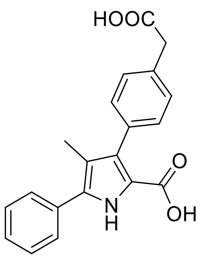

UPLC traces of PyC 11

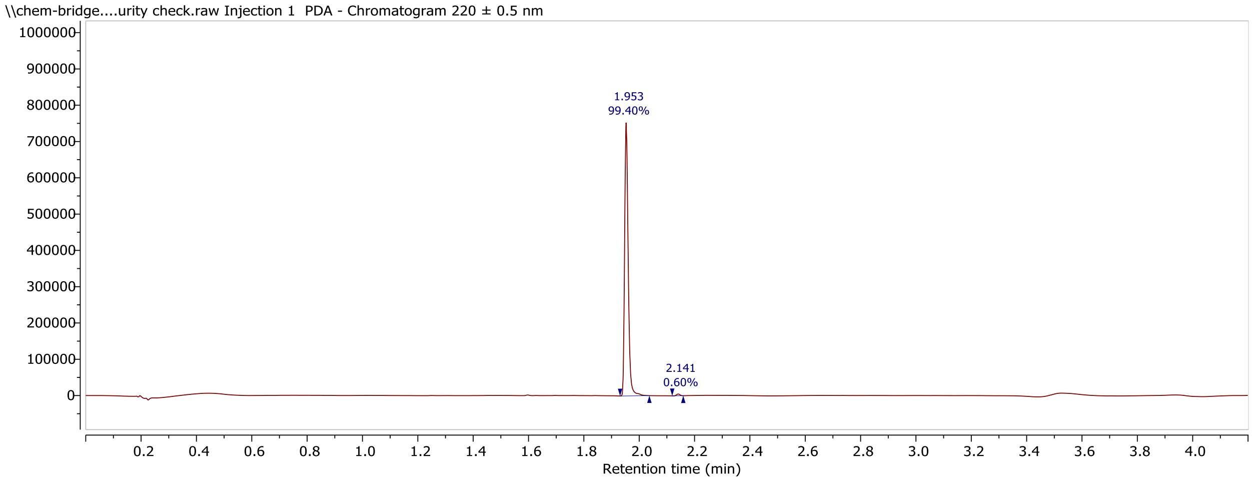

	RT	Scan	Type	Height	Area	Total Height %	Total Area %	Start time	End time
1	1.953	2343	BB	753019.074	13518738.541	99.36	99.40	1.931	2.037
2	2.141	2569	BB	4885.789	81380.575	0.64	0.60	2.119	2.159

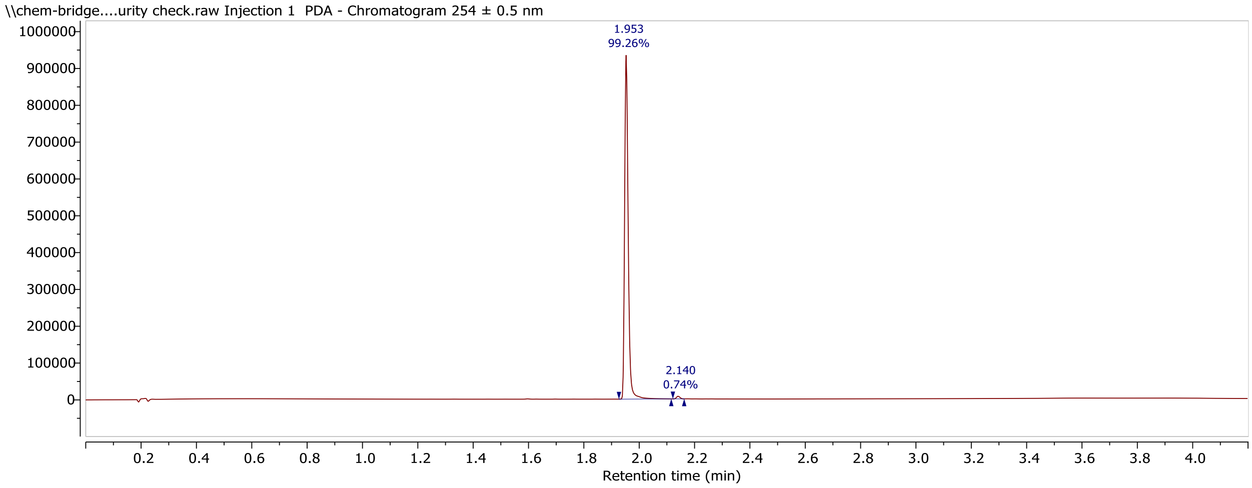

	RT	Scan	Type	Height	Area	Total Height %	Total Area %	Start time	End time
1	1.953	2343	BB	933864.894	16831000.000	99.23	99.26	1.927	2.116
2	2.140	2568	BV	7200.240	125756.460	0.77	0.74	2.122	2.163

HRMS trace of PyC 11

Expanded Spectrum RT 0.11, NL 10515305, Peak [1], Target Mass 336.1230

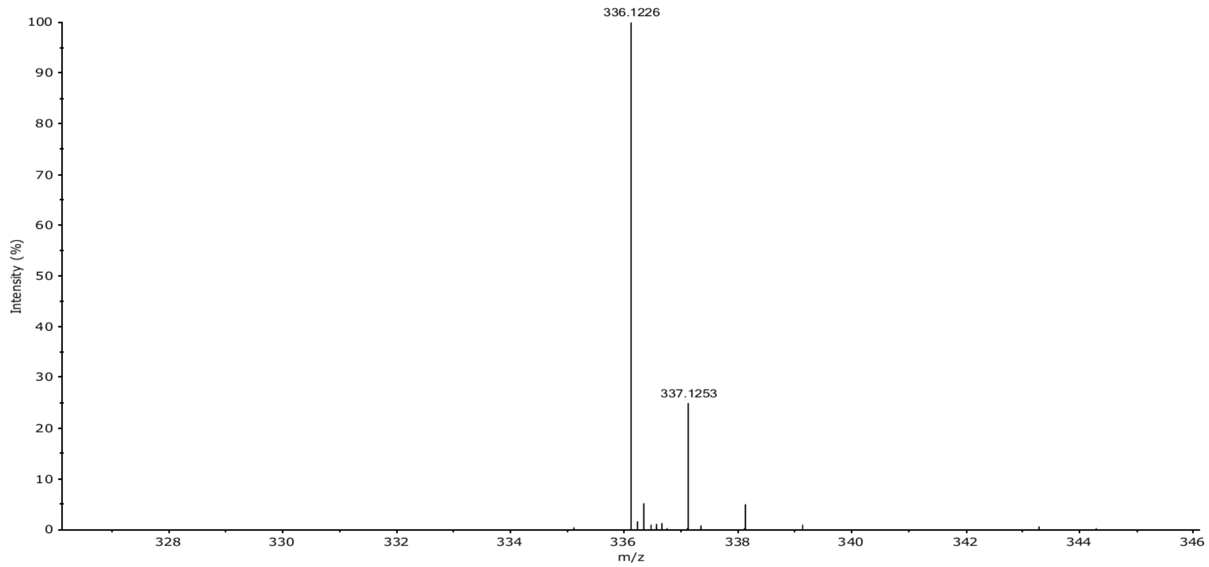

Measured Mass	Calculated Mass	Error (mDa)	Error (ppm)	Formula $[M+H]^+$	Response
336.1223	336.1230	-0.74	-2.19	$C_{20}H_{18}NO_4$	36868837

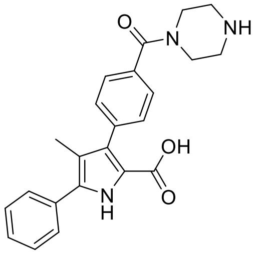

UPLC Traces of PyC 12

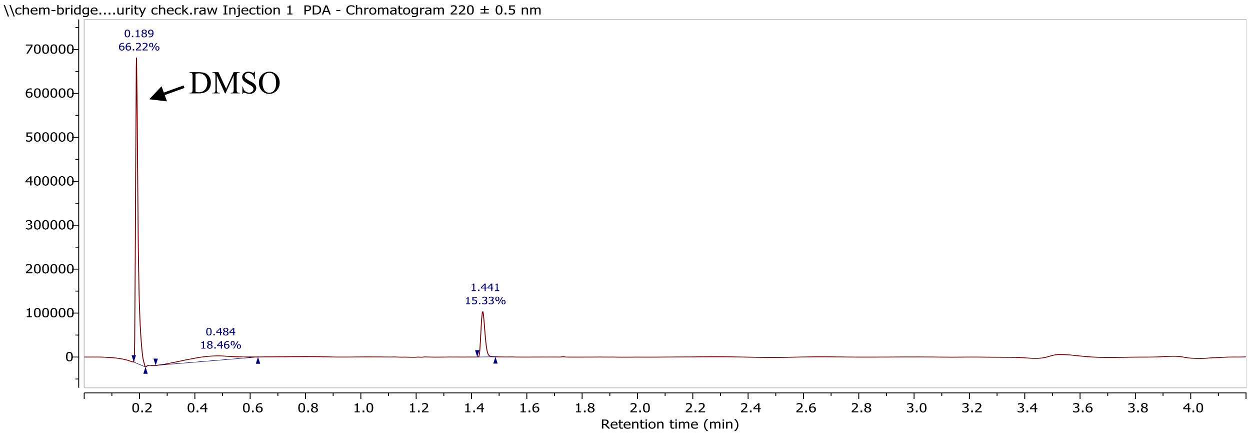

	RT	Scan	Type	Height	Area	Total Height %	Total Area %	Start time	End time
1	0.484	581	BB	9966.182	2491006.313	1.23	18.46	0.258	0.628
2	0.189	227	BB	695336.588	8937372.000	86.00	66.22	0.179	0.222
3	1.441	1729	BB	103246.796	2068644.656	12.77	15.33	1.421	1.487

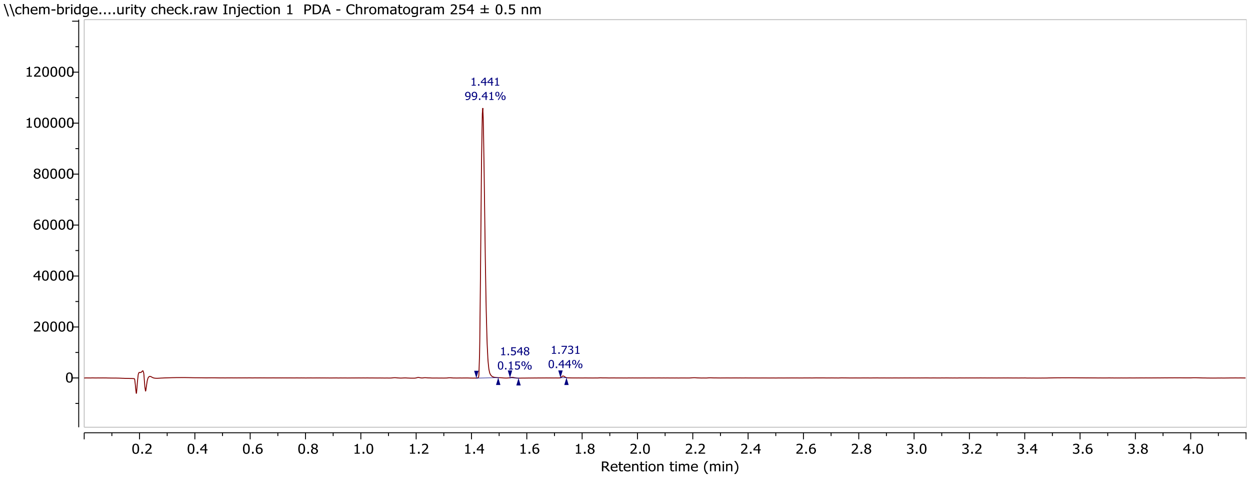

	RT	Scan	Type	Height	Area	Total Height %	Total Area %	Start time	End time
1	1.441	1729	BB	105923.842	2122994.000	99.16	99.41	1.418	1.497
2	1.548	1857	BB	205.865	3126.570	0.19	0.15	1.539	1.570
3	1.731	2077	BB	693.385	9458.000	0.65	0.44	1.722	1.743

HRMS Trace of PyC 12

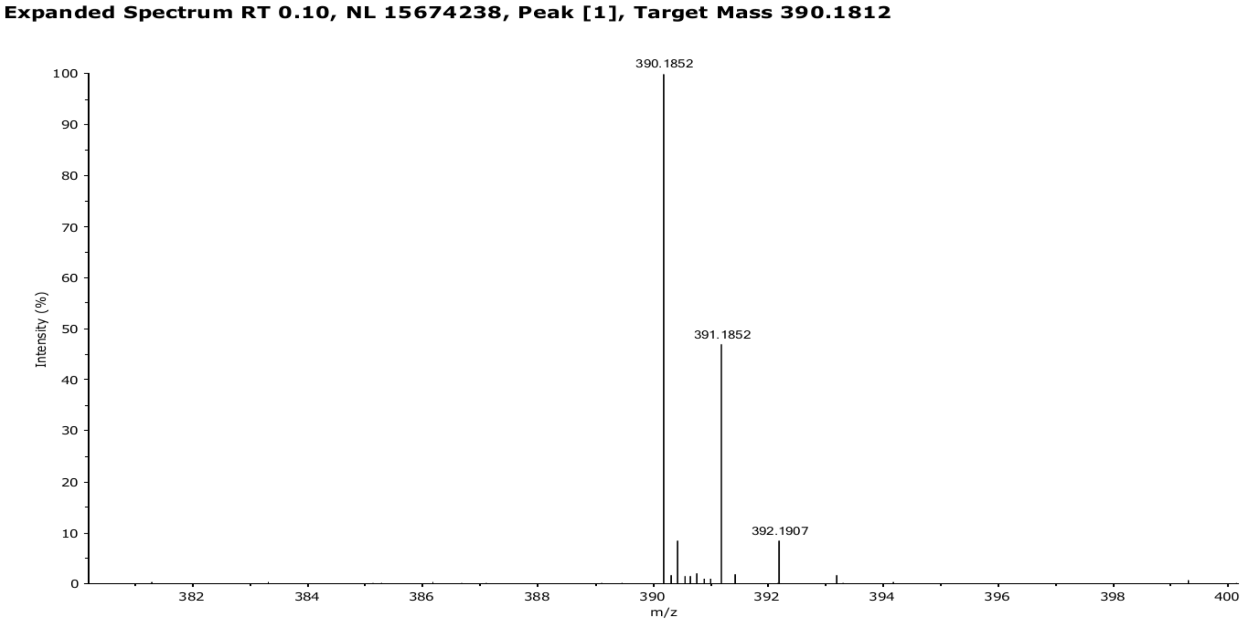

Measured Mass	Calculated Mass	Error (mDa)	Error (ppm)	Formula $[M+H]^+$	Response
390.1825	390.1812	1.28	3.28	$C_{23}H_{24}N_3O_3$	74117500

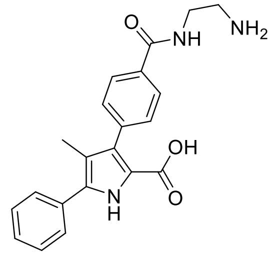

UPLC Traces of PyC 13

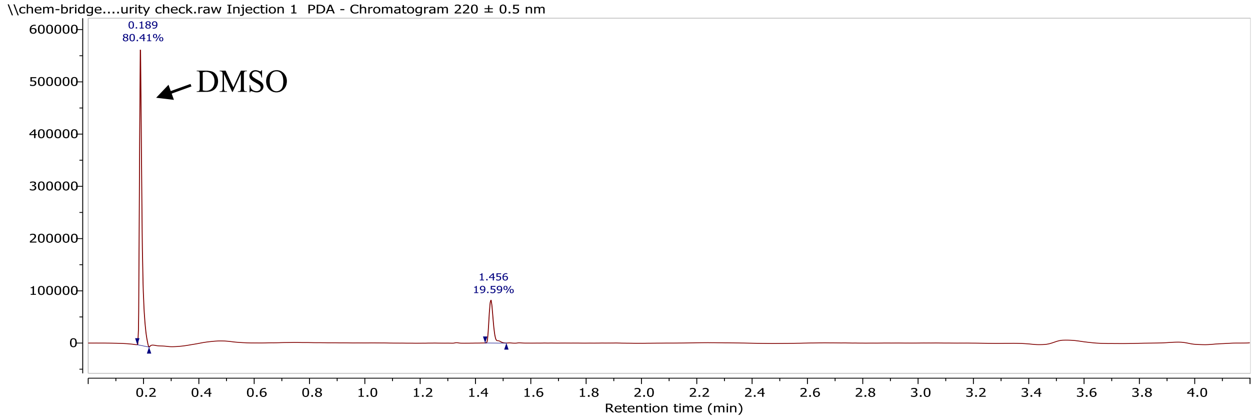

	RT	Scan	Type	Height	Area	Total Height %	Total Area %	Start time	End time
1	0.189	226	BB	565479.784	7271578.000	87.30	80.41	0.177	0.220
2	1.456	1747	BV	82261.708	1771513.422	12.70	19.59	1.435	1.512

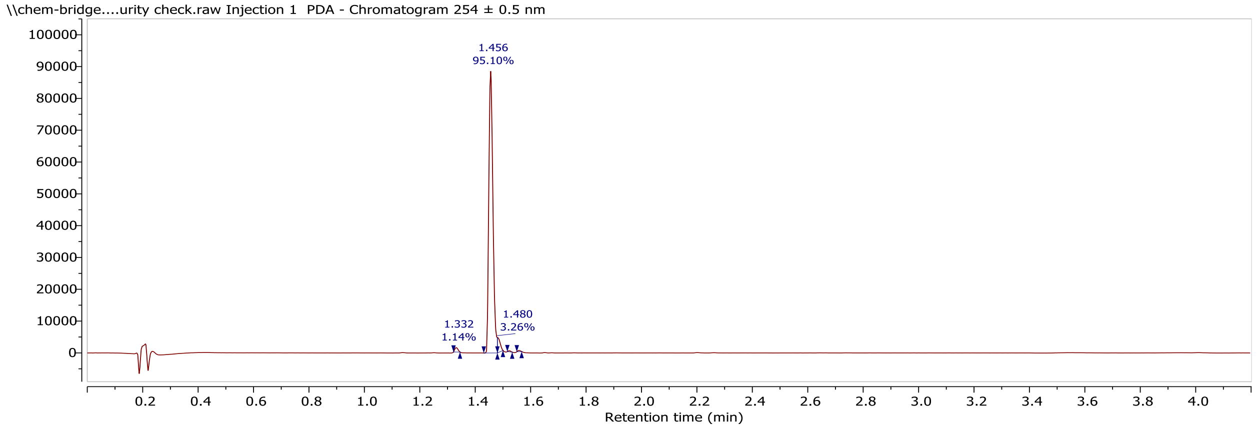

	RT	Scan	Type	Height	Area	Total Height %	Total Area %	Start time	End time
1	1.557	1868	BB	385.476	5006.000	0.40	0.26	1.550	1.568
2	1.523	1828	BB	367.857	4561.000	0.39	0.24	1.516	1.533
3	1.480	1776	BB	4821.270	62636.245	5.05	3.26	1.480	1.500
4	1.456	1747	BV	88561.392	1828505.476	92.71	95.10	1.431	1.480
5	1.332	1598	BB	1384.429	21911.500	1.45	1.14	1.322	1.345

HRMS Trace of PyC 13

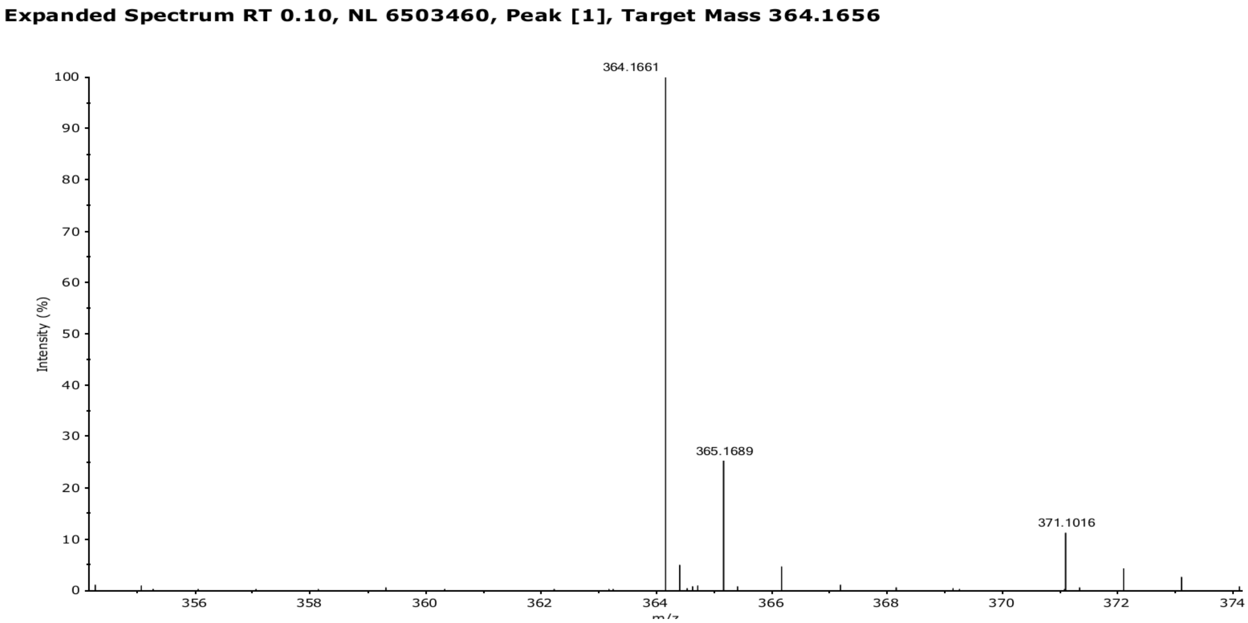

Measured Mass	Calculated Mass	Error (mDa)	Error (ppm)	Formula $[M+H]^+$	Response
364.1661	364.1656	0.53	1.46	$C_{21}H_{22}N_3O_3$	23110637

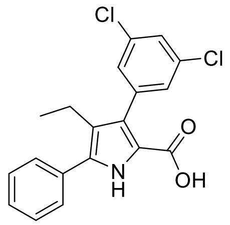

UPLC Traces of PyC 14

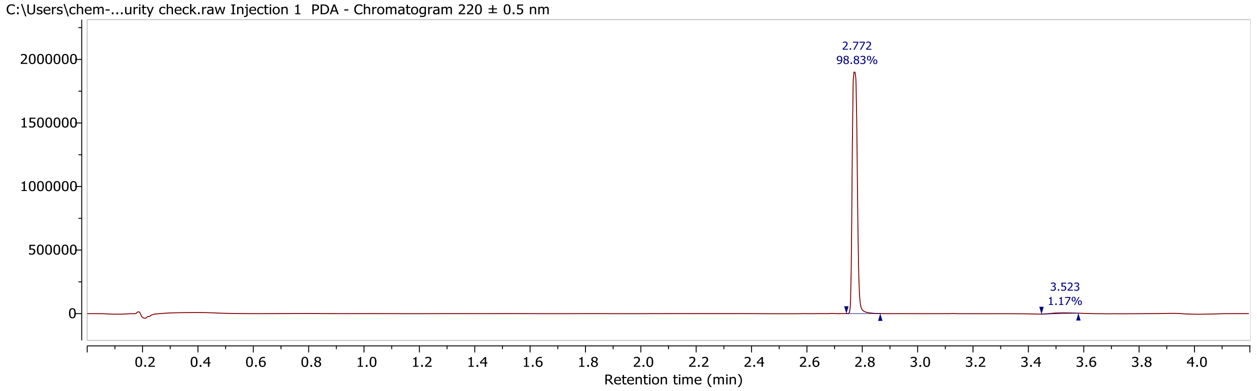

	RT	Scan	Type	Height	Area	Total Height %	Total Area %	Start time	End time
1	2.772	3326	BB	1901904.631	48199169.448	99.67	98.83	2.743	2.865
2	3.523	4228	BB	6235.479	571551.352	0.33	1.17	3.447	3.581

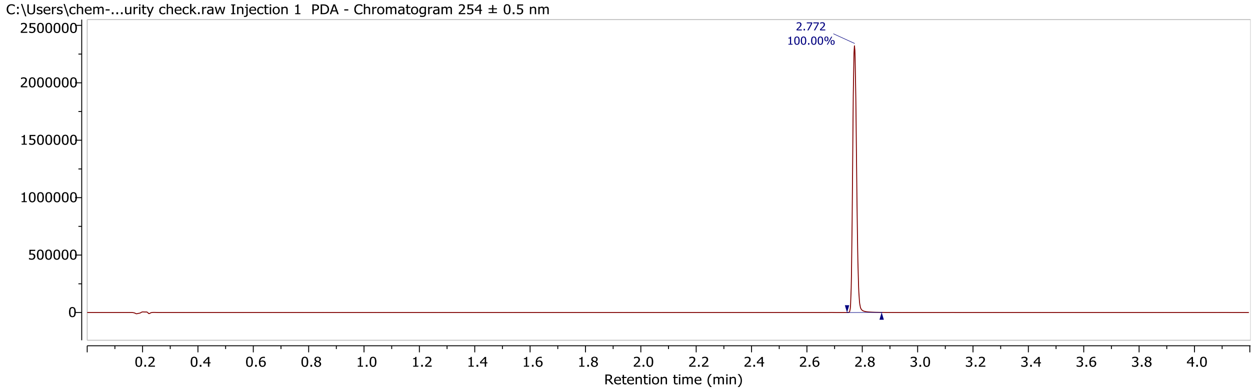

	RT	Scan	Type	Height	Area	Total Height %	Total Area %	Start time	End time
1	2.772	3326	BB	2323722.533	45246624.500	100.00	100.00	2.745	2.870

HRMS Trace of PyC 14

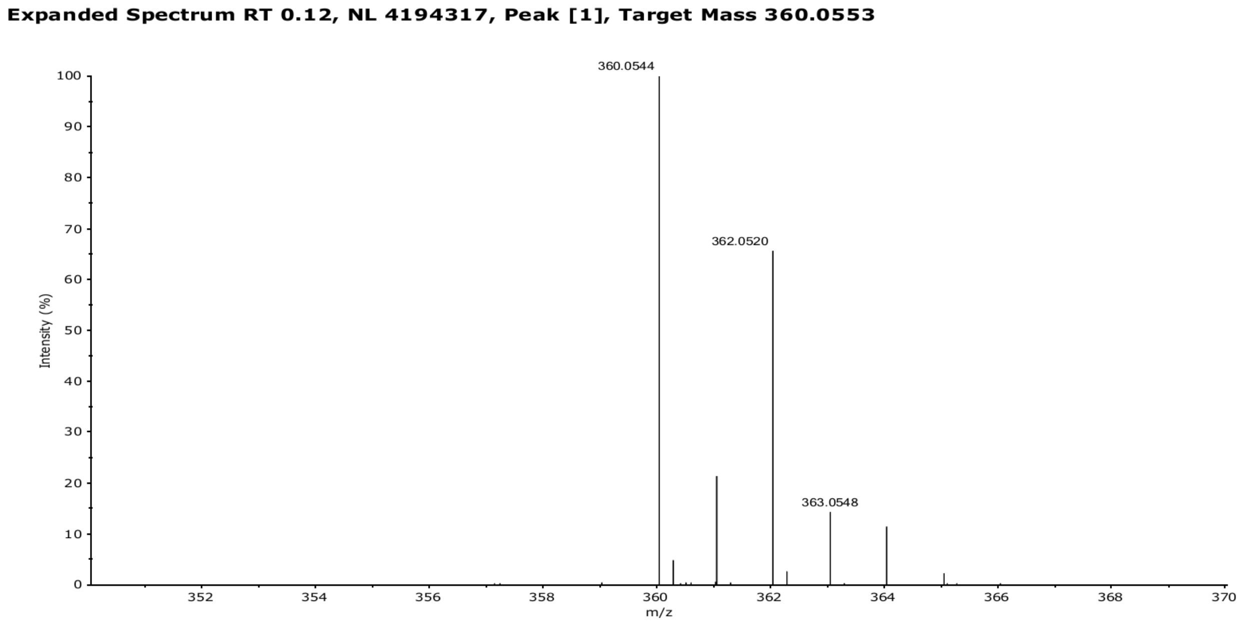

Measured Mass	Calculated Mass	Error (mDa)	Error (ppm)	Formula [M+H]$^+$	Response
360.0544	360.0553	-0.86	-2.39	$C_{19}H_{16}Cl_2NO_2$	21671067

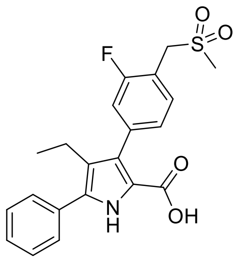

UPLC traces of PyC 15

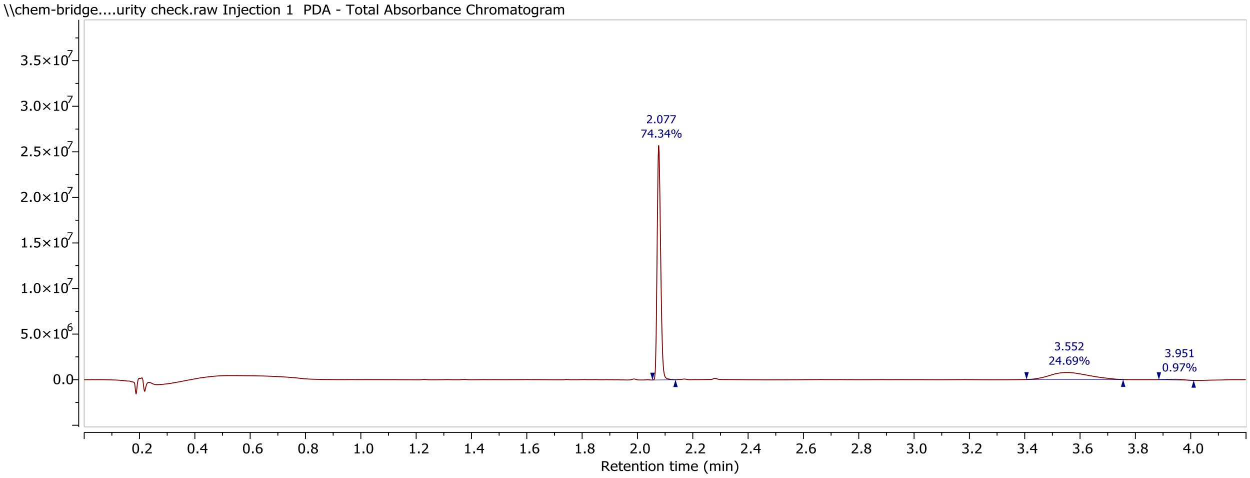

	RT	Scan	Type	Height	Area	Total Height %	Total Area %	Start time	End time
1	3.552	4262	BB	765562.975	151162101.655	2.88	24.69	3.407	3.756
2	3.951	4741	BB	78729.399	5951592.651	0.30	0.97	3.885	4.011
3	2.077	2492	BB	25729827.580	455160804.000	96.82	74.34	2.054	2.138

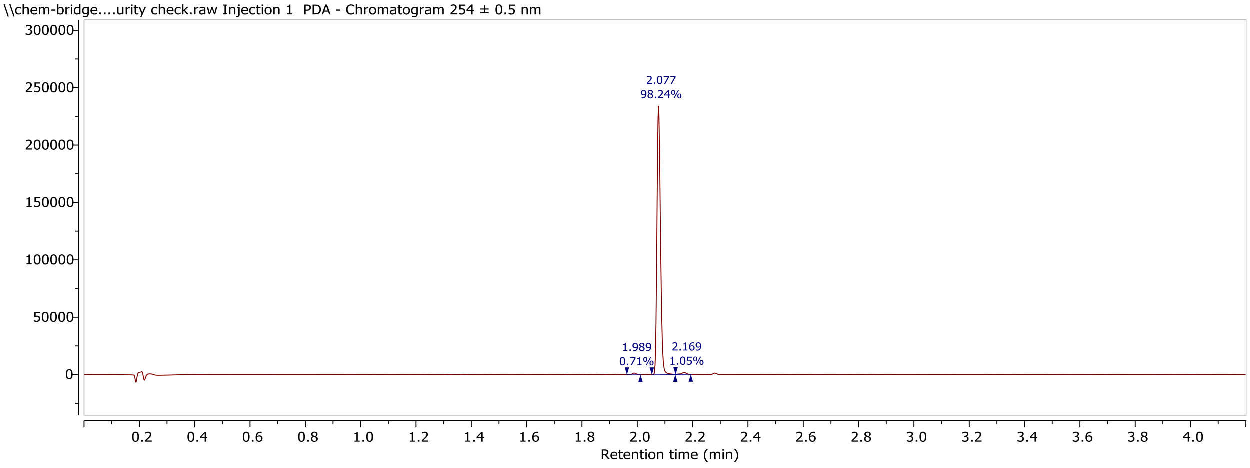

	RT	Scan	Type	Height	Area	Total Height %	Total Area %	Start time	End time
1	2.169	2603	VB	1642.853	44385.559	0.69	1.05	2.138	2.193
2	1.989	2386	BB	1432.390	29937.000	0.60	0.71	1.963	2.012
3	2.077	2492	BV	234037.854	4145478.000	98.70	98.24	2.053	2.138

HRMS Traces of PyC 15

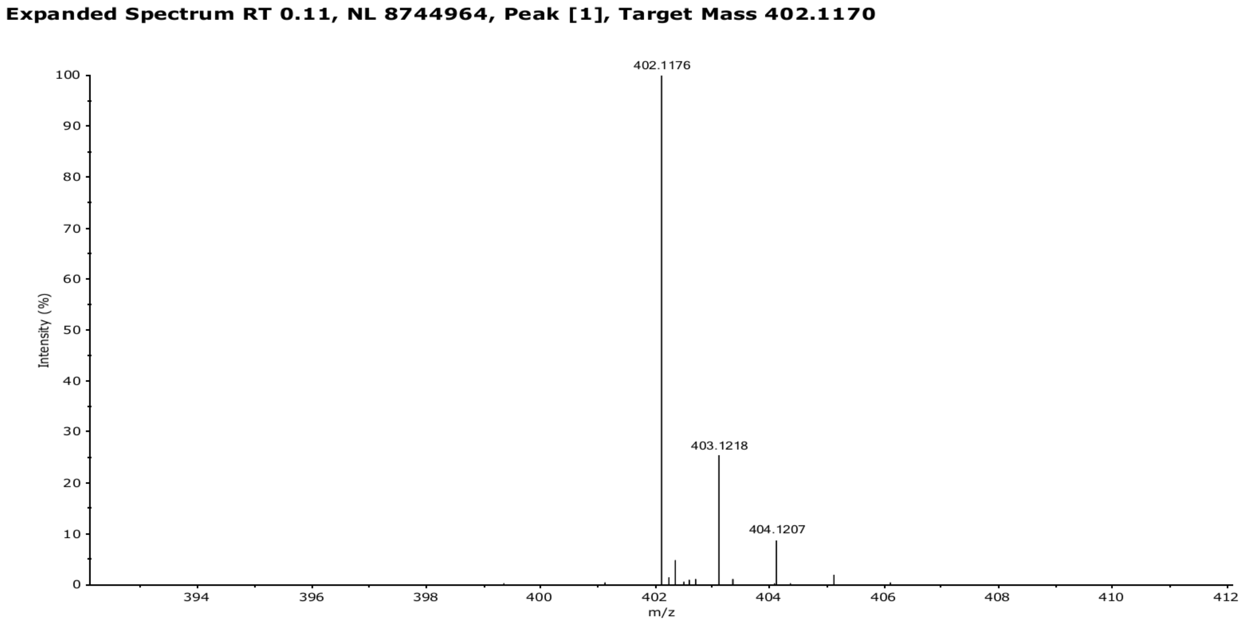

Measured Mass	Calculated Mass	Error (mDa)	Error (ppm)	Formula $[M+H]^+$	Response
402.1176	402.1170	0.61	1.53	$C_{21}H_{21}FNO_4S$	28609472

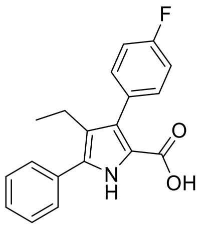

UPLC traces of PyC 16

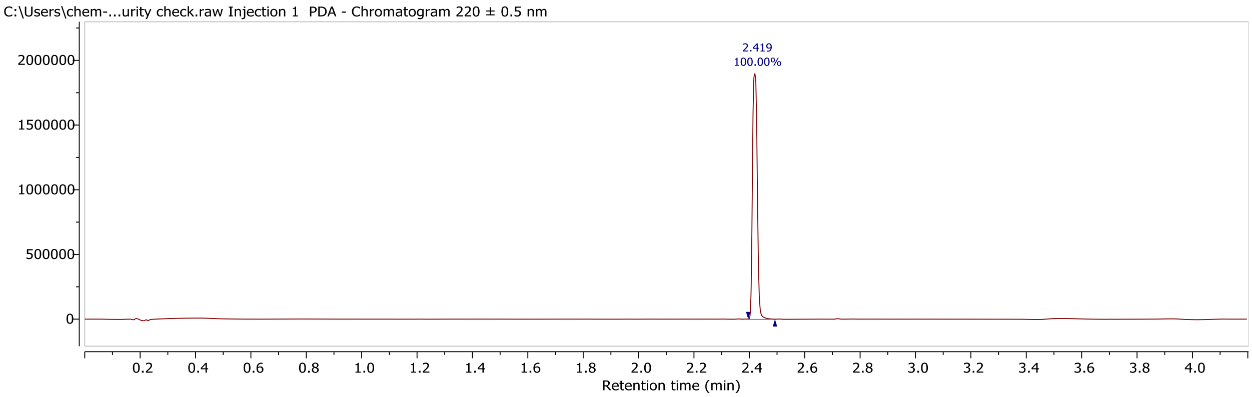

	RT	Scan	Type	Height	Area	Total Height %	Total Area %	Start time	End time
1	2.419	2903	VB	1897876.322	45509019.824	100.00	100.00	2.396	2.493

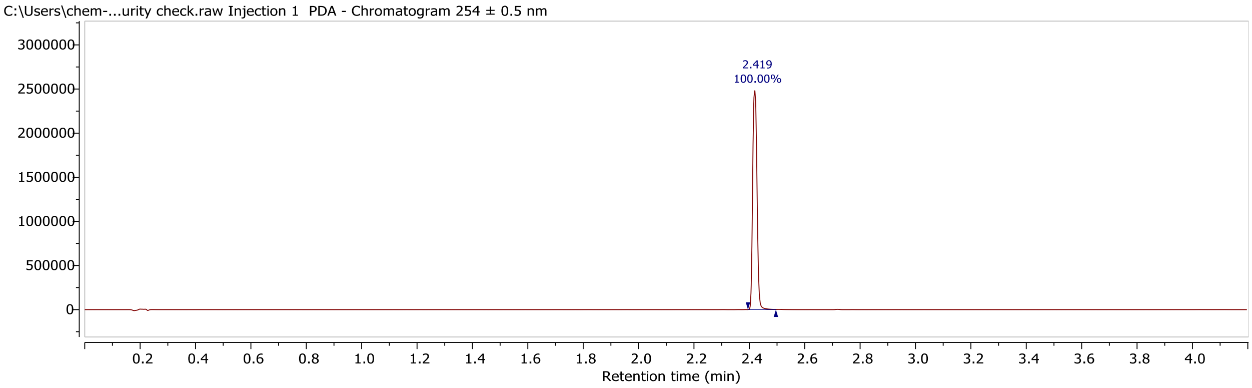

	RT	Scan	Type	Height	Area	Total Height %	Total Area %	Start time	End time
1	2.419	2903	VV	2482896.704	54137990.000	100.00	100.00	2.395	2.496

HRMS Trace of PyC 16

Expanded Spectrum RT 0.11, NL 5614383, Peak [1], Target Mass 332.1057

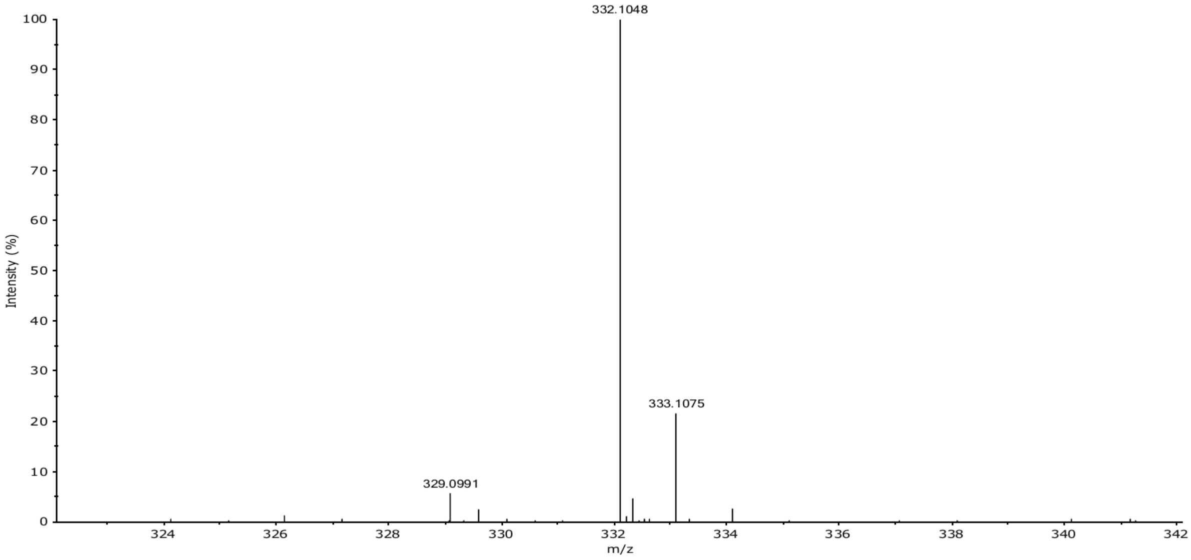

Measured Mass	Calculated Mass	Error (mDa)	Error (ppm)	Formula $[M+Na]^+$	Response
332.1048	332.1057	-0.93	-2.80	$C_{19}H_{16}FNO_2Na$	19344940

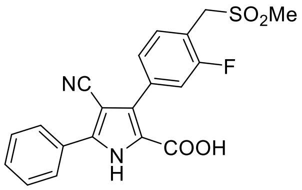

UPLC Traces of PyC 17

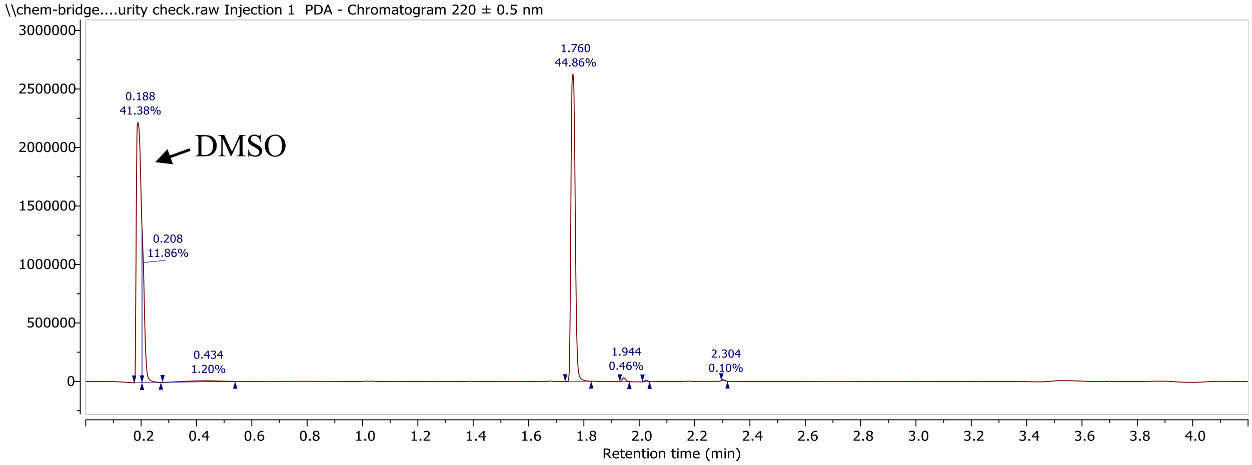

	RT	Scan	Type	Height	Area	Total Height %	Total Area %	Start time	End time
1	0.188	226	BV	2226496.690	51566294.414	37.59	41.38	0.175	0.203
2	0.208	250	VB	1008619.724	14780449.586	17.03	11.86	0.203	0.272
3	0.434	521	BB	8225.542	1492851.504	0.14	1.20	0.277	0.540
4	1.760	2112	BB	2625815.880	55907300.566	44.33	44.86	1.733	1.827
5	1.944	2333	BB	33886.829	578000.000	0.57	0.46	1.930	1.964
6	2.024	2429	BB	10688.220	164050.188	0.18	0.13	2.012	2.038
7	2.304	2765	BB	9477.333	127344.000	0.16	0.10	2.297	2.319

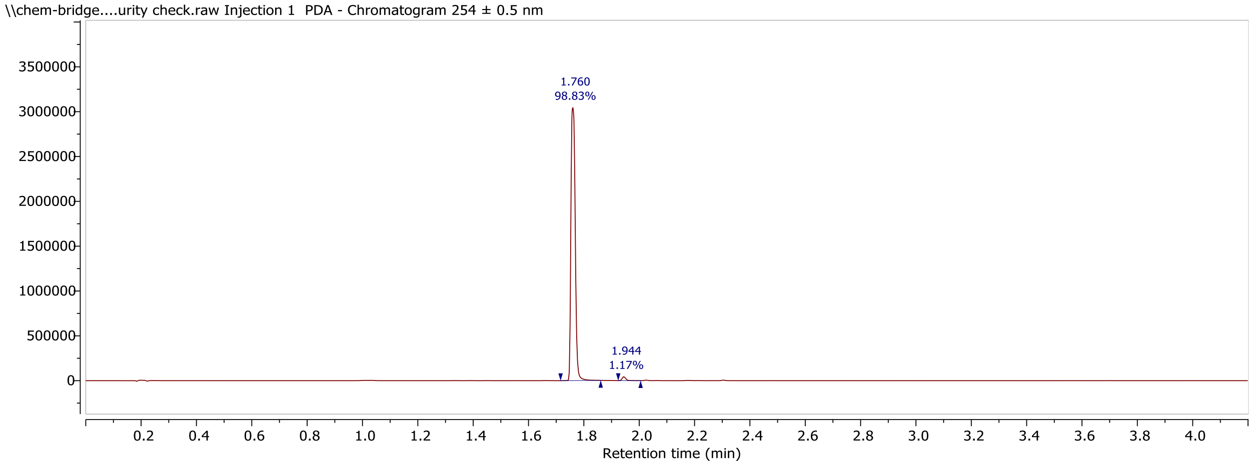

	RT	Scan	Type	Height	Area	Total Height %	Total Area %	Start time	End time
1	1.760	2111	BB	3043147.747	65856832.000	98.61	98.83	1.716	1.861
2	1.944	2333	BB	42981.969	780041.000	1.39	1.17	1.924	2.005

HRMS Trace of PyC 17

Expanded Spectrum RT 0.12, NL 340815, Peak [1], Target Mass 399.0809

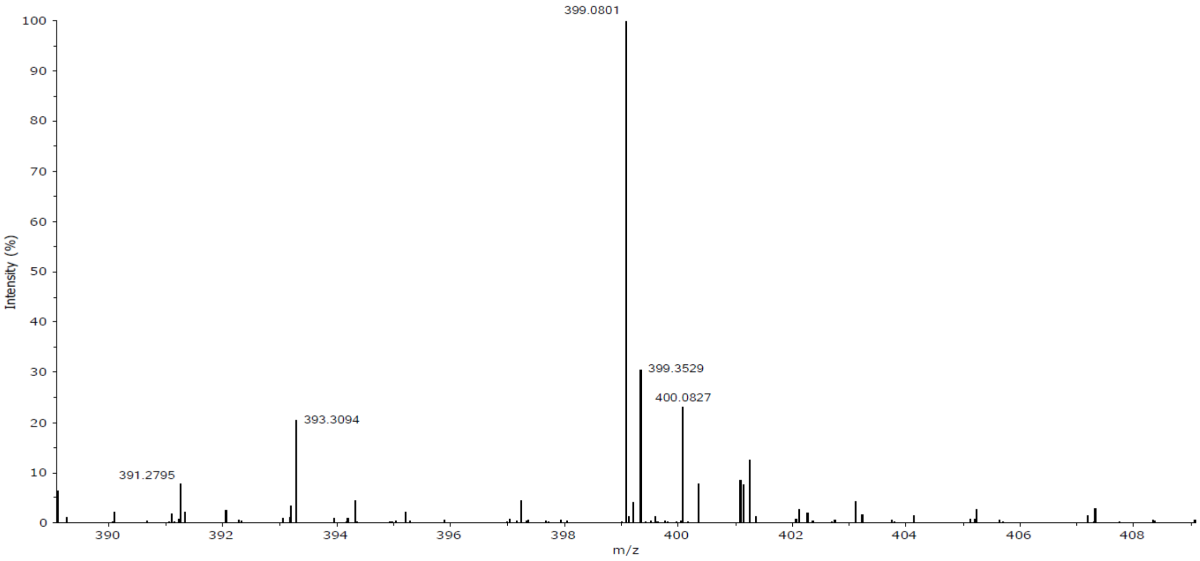

Measured Mass	Calculated Mass	Error (mDa)	Error (ppm)	Formula $[M+H]^+$	Response
399.0801	399.0809	-0.83	-2.09	$C_{20}H_{16}FN_2O_4S$	269959

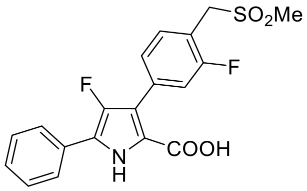

UPLC Traces of PyC 18

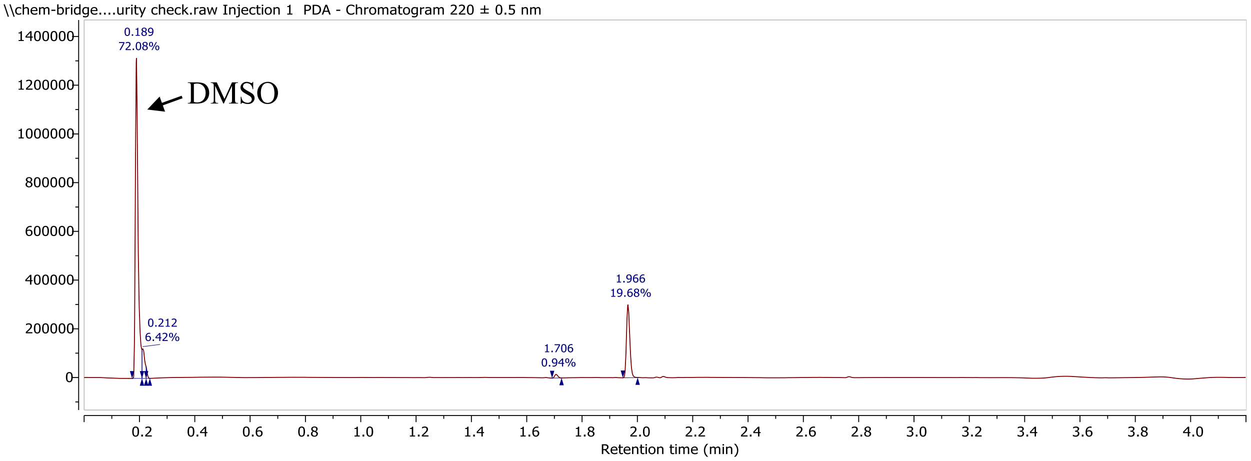

	RT	Scan	Type	Height	Area	Total Height %	Total Area %	Start time	End time
1	0.212	255	VV	121419.766	1651294.494	6.76	6.42	0.209	0.224
2	0.225	270	VB	43539.883	225505.403	2.43	0.88	0.224	0.237
3	0.189	227	BV	1314753.948	18535049.104	73.25	72.08	0.173	0.209
4	1.706	2047	BB	15065.950	240906.814	0.84	0.94	1.692	1.726
5	1.966	2359	BB	300050.000	5061296.000	16.72	19.68	1.948	2.001

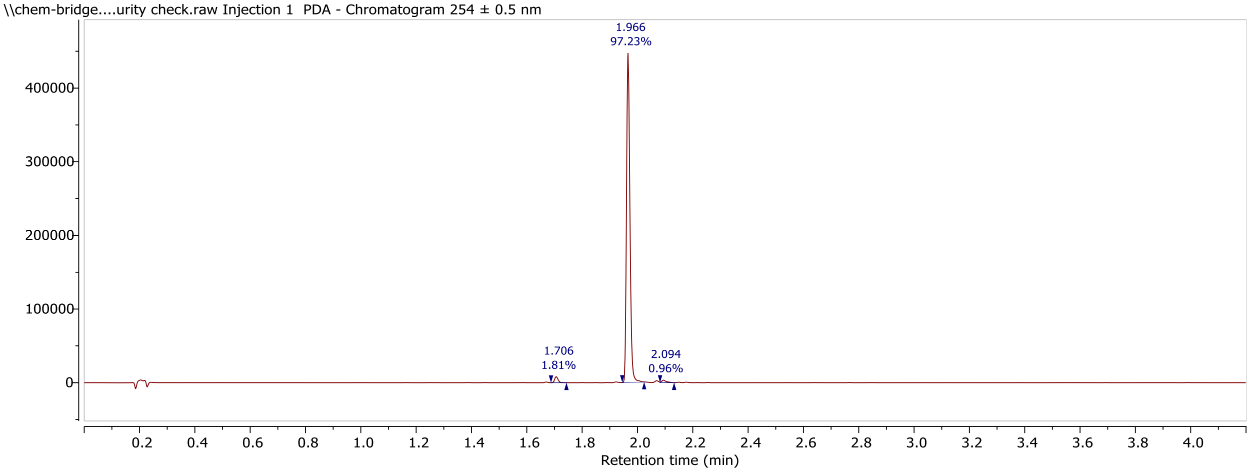

	RT	Scan	Type	Height	Area	Total Height %	Total Area %	Start time	End time
1	1.706	2047	VB	8182.818	142454.909	1.78	1.81	1.688	1.743
2	2.094	2512	VB	3368.765	75656.531	0.73	0.96	2.082	2.133
3	1.966	2359	BB	447117.105	7646032.000	97.48	97.23	1.945	2.024

HRMS Traces of PyC 18

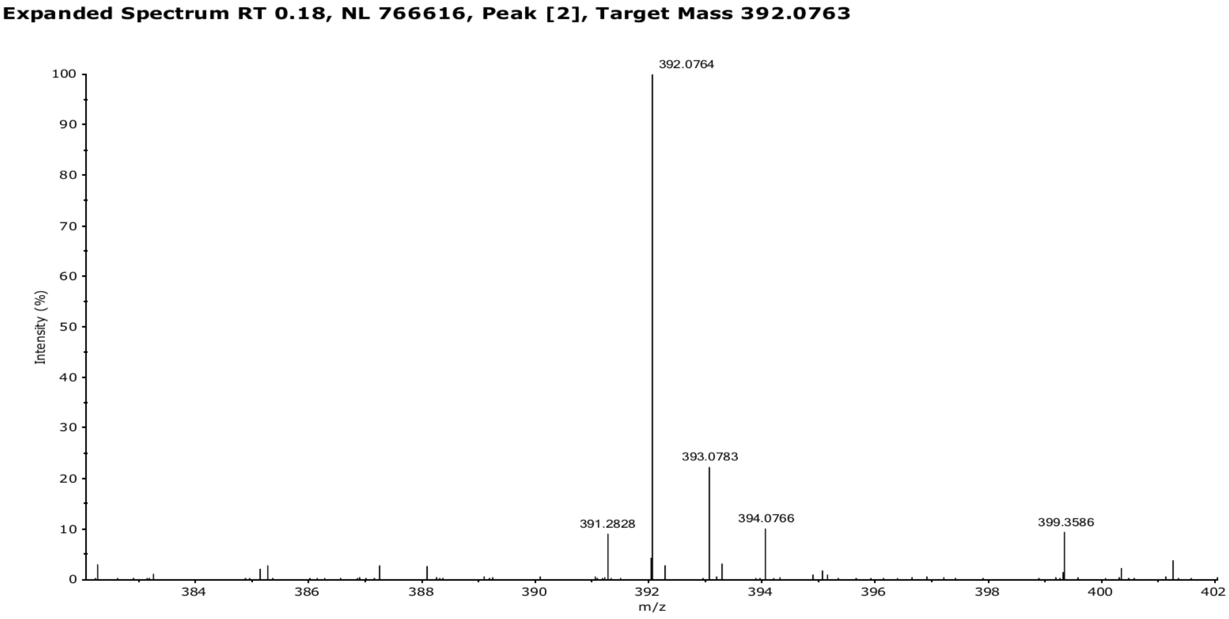

Measured Mass	Calculated Mass	Error (mDa)	Error (ppm)	Formula $[M+H]^+$	Response
392.0764	392.0763	0.14	0.35	$C_{19}H_{16}F_2NO_4S$	851938
